# Supplementary figures and images for: Lineage-specific gene duplication and expansion of DUF1216 gene family in Brassicaceae
Source: PLoS One. 2024 Apr 16;19(4):e0302292. doi: 10.1371/journal.pone.0302292 (PMC11020792; doi:10.1371/journal.pone.0302292)

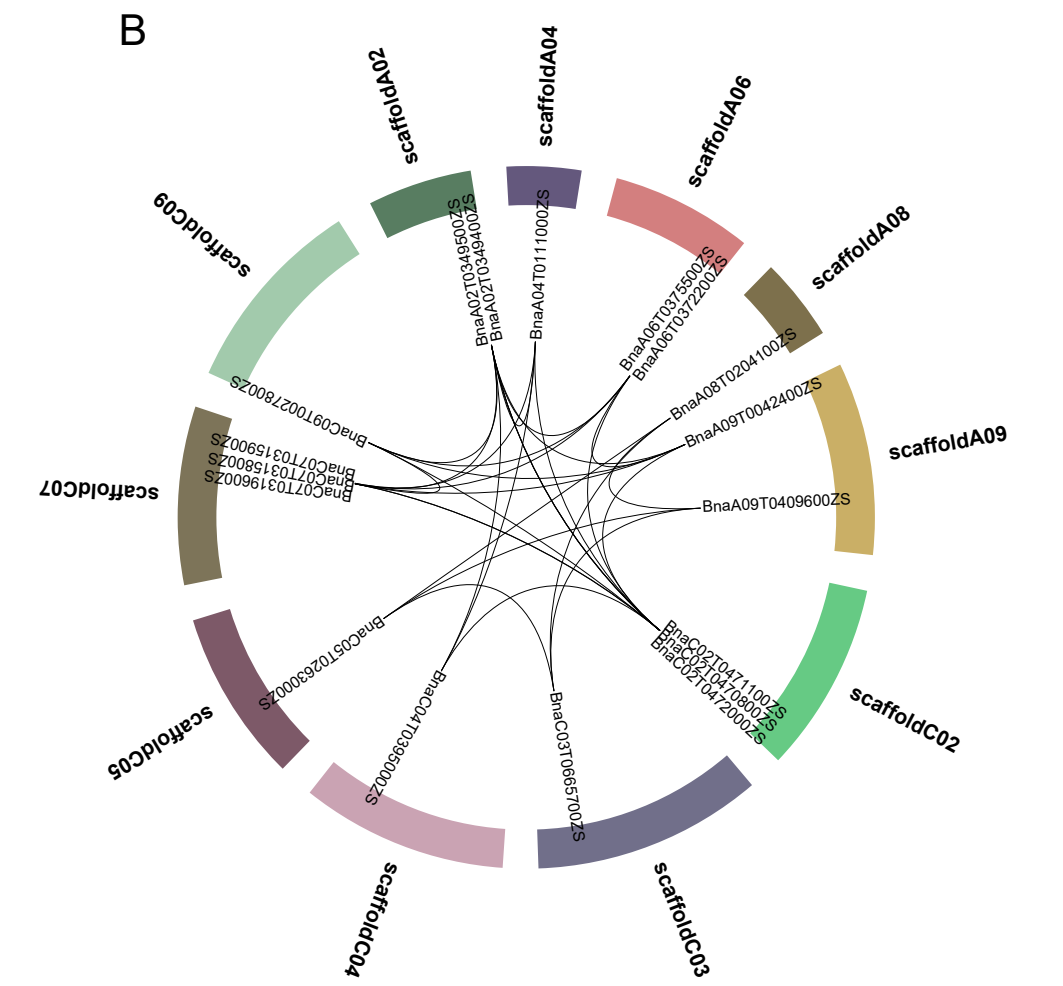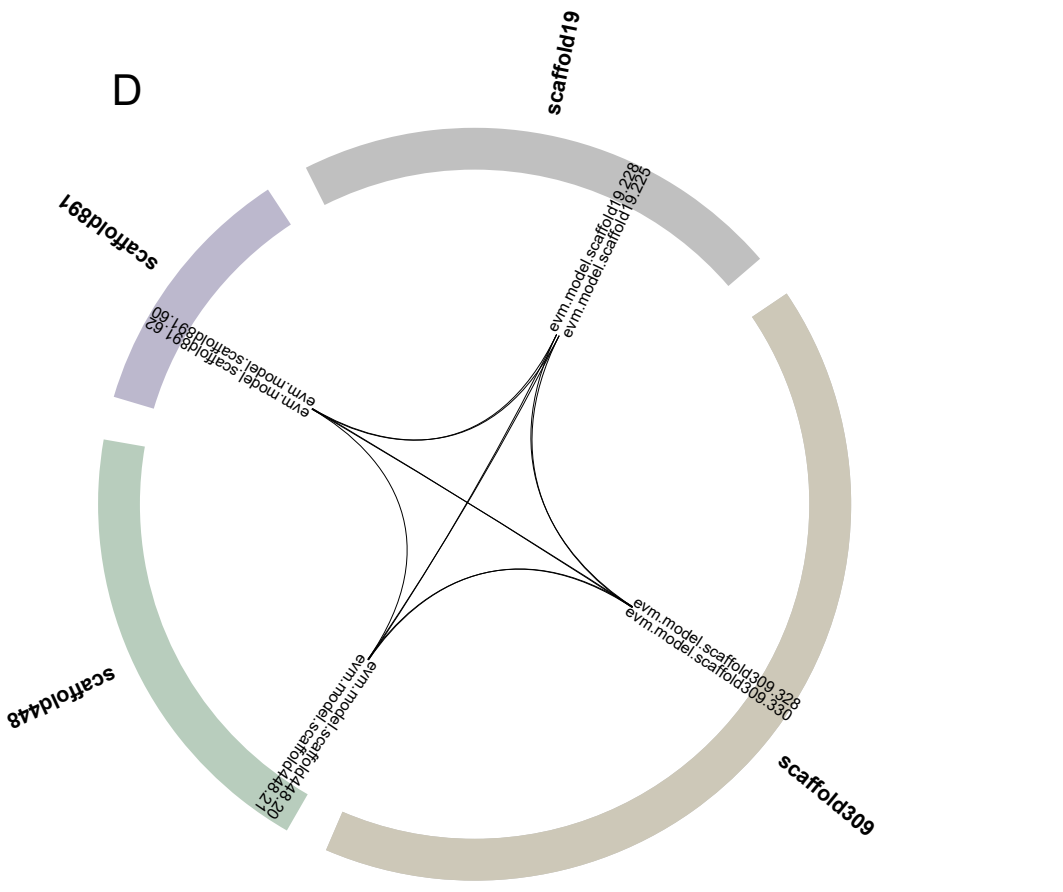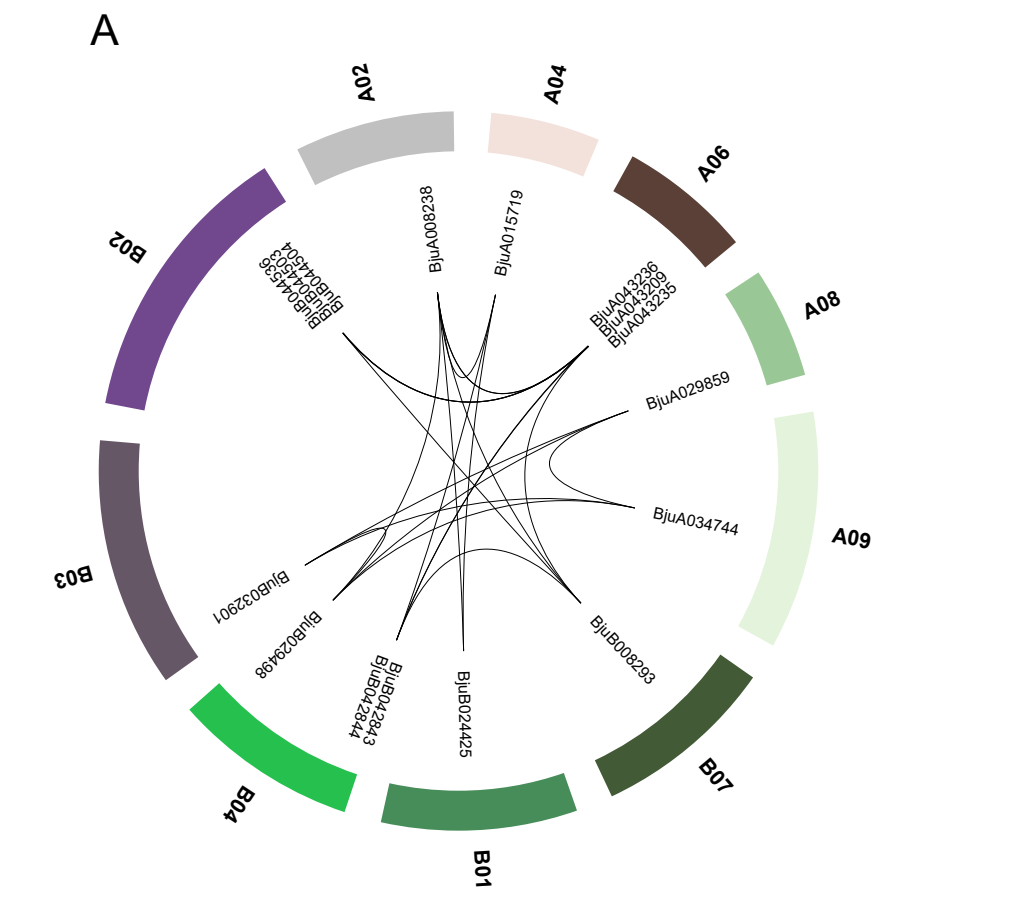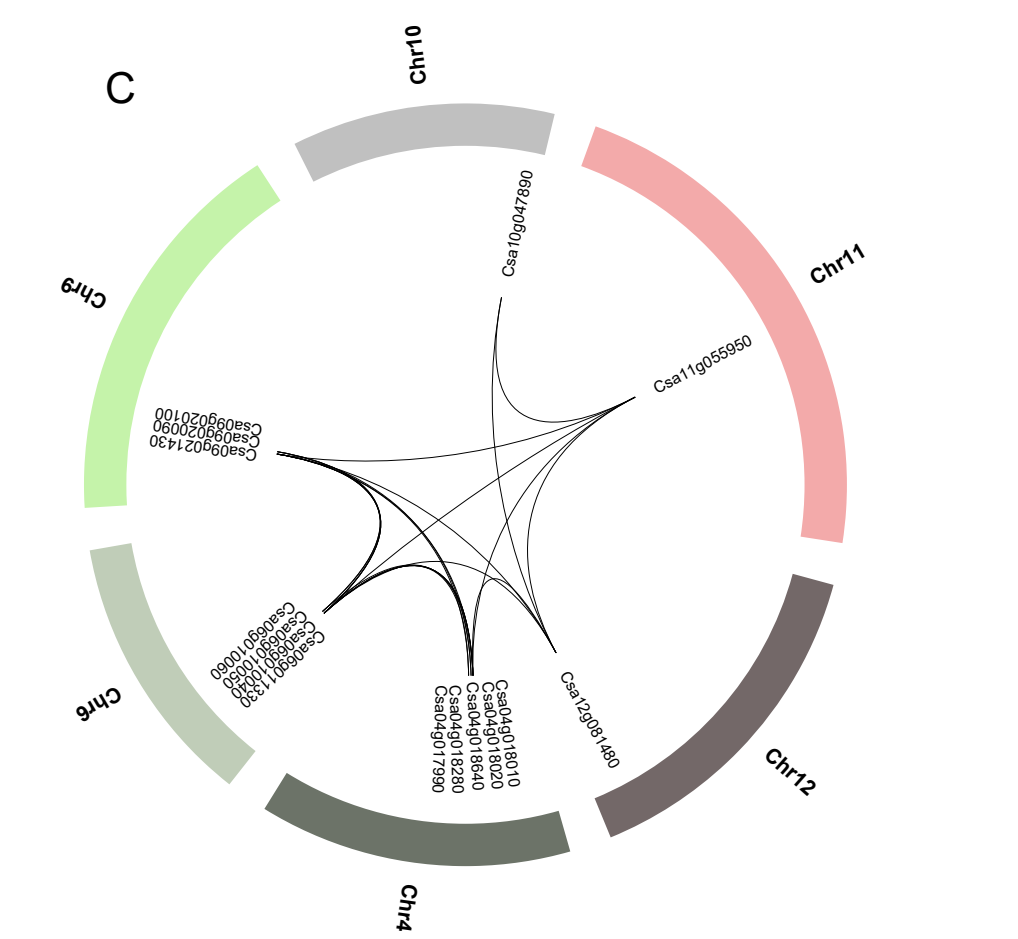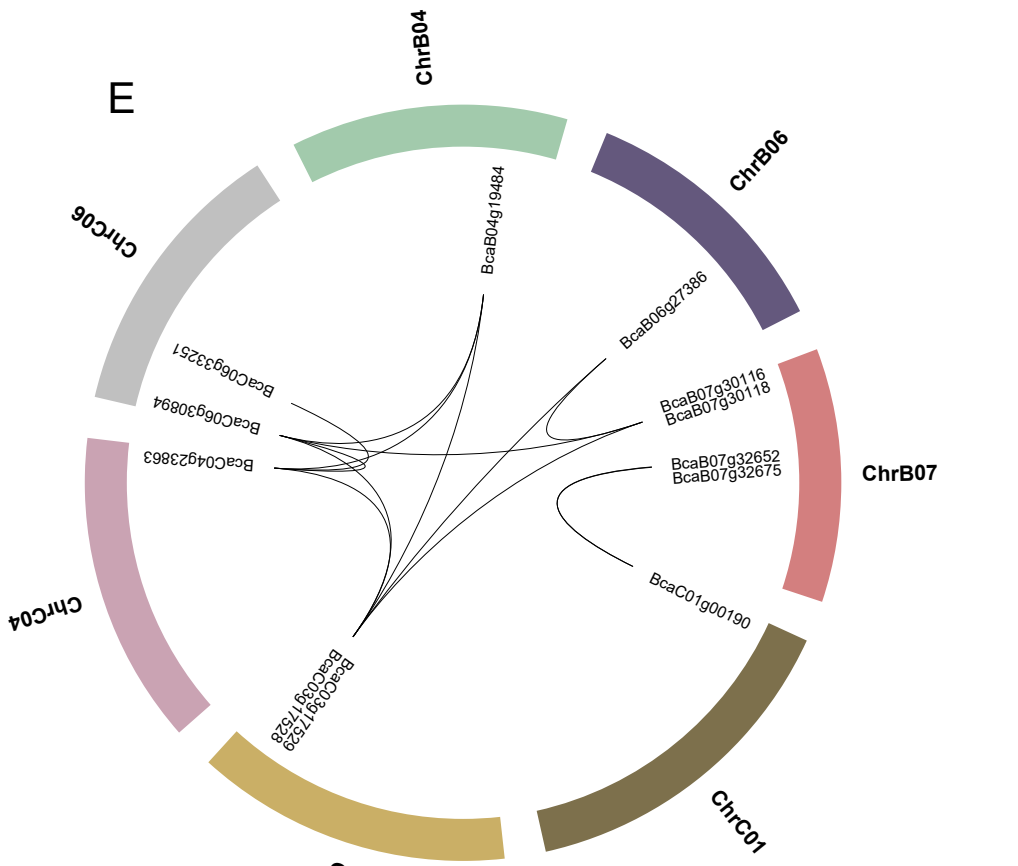

Supplement: S3 Fig — Synteny genes are linked by black lines. (PDF) [file pone.0302292.s003.pdf]
