# Supplementary material for: Lineage-specific gene duplication and expansion of DUF1216 gene family in Brassicaceae
Source: PLoS One. 2024 Apr 16;19(4):e0302292. doi: 10.1371/journal.pone.0302292 (PMC11020792; doi:10.1371/journal.pone.0302292)
Supplement: S4 Fig — (PDF) [file pone.0302292.s004.pdf]

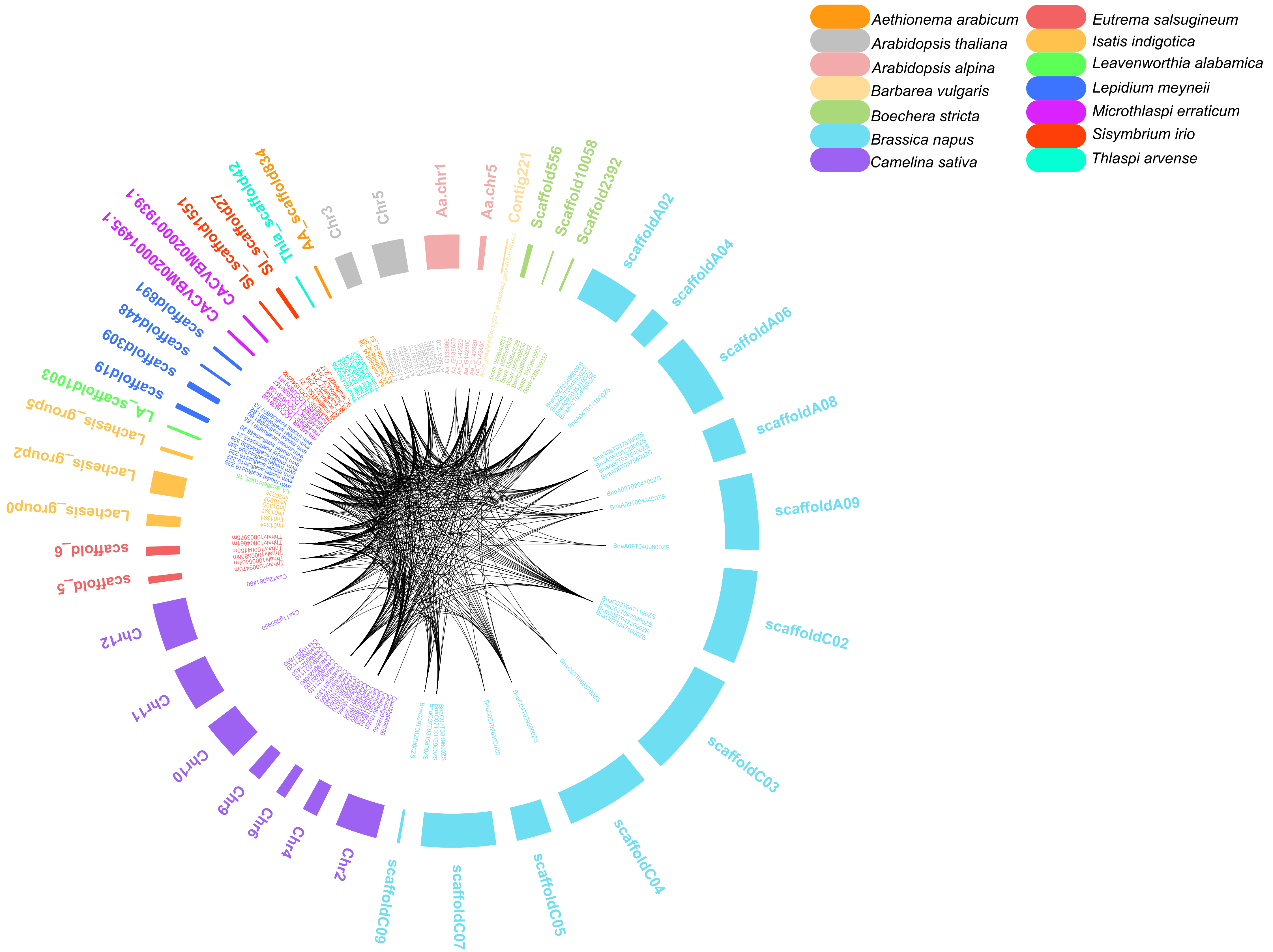
Aethionema arabicum
Arabidopsis thaliana
Arabidopsis alpina
Barbarea vulgaris
Boechera stricta
Brassica napus
Camelina sativa
Eutrema salsugineum
Isatis indigotica
Leavenworthia alabamica
Lepidium meyneii
Microthlaspi erraticum
Sisymbrium irio
Thlaspi arvense
AA_scaffold834
Sl_scaffold27
Sl_scaffold151
CACVBM020001939.1
CACVBM020001495.1
scaffold891
scaffold448
scaffold309
scaffold19
LA_scaffold1003
Lachesis_group5
Lachesis_group2
Lachesis_group0
scaffold_6
scaffold_5
Chr12
Chr11
Chr10
Chr9
Chr6
Chr4
Chr2
scaffoldC09
scaffoldC07
scaffoldC05
scaffoldC04
scaffoldC03
scaffoldC02
scaffoldA09
scaffoldA08
scaffoldA06
scaffoldA04
scaffoldA02
Scaffold2392
Scaffold10058
Scaffold556
Contig221
Aa.chr5
Aa.chr1
Chr5
Chr3
