# Supplementary material for: Lineage-specific gene duplication and expansion of DUF1216 gene family in Brassicaceae
Source: PLoS One. 2024 Apr 16;19(4):e0302292. doi: 10.1371/journal.pone.0302292 (PMC11020792; doi:10.1371/journal.pone.0302292)
Supplement: S7 Table — (DOCX) [file pone.0302292.s011.docx]

>AA_scaffold1851_2

MEKRSLAICLIFLITASTIYEAQGTFLLKMYLRKRFPGRGRDVGGFACKGMSTFMANLQGKCPVTSEFKQFFGSFNTFISFINSAKSASDESQVEGKVDAVCNSMGKIDSTTKSETRATLISMGRTLISQKLSGSNMMTVQQRKELVISLVKWFQVIGKFVKVAAEQRGKAIDLNSFGLDGIDANAVSSSSSSSSRSSSDSSSSDESSSTKTGSSSTKTPTPKTPSTPTETPKSSSSSGKSSSSGSASASGSVSSKKESSTSTTPTPKTPSTPTETPKSSSSGSTSSSGKSSSSGSASSSSKKESSTSTTPTPTPETPKASSSGSSGKSSASGSSSSSSSSSSKSSSSKSVSASASASASSAGTVKQVEDDCSNDVKSFISNLEKKYSSNSLLKGFFENVKSSMTSSTNQKDFFGGLISTGGKIAEGMSTVGSKVAKSATAQSSVKSYQAEVMKTLQELQSLNSKIQSENKGKQQESVKVSTTQQKEIKQTITRWEQVTTKFVETAAQSSSSSSSSSSSSSSSKSASAQSRKLLIAN

>AA_scaffold834_56

MARVQVLLCFTILFASATLLNVVSAHLKYEPALSTIEAPKTVKDVEPYTVKVVMTFVVNLEKECPKSKPFQLFFEKLRAYAKYVCPIVIVKKGADLKGYDSDRKAKIGSLLQAMSTFARGSIKKVLQEEKKDVLNTFNWMQGVAGKIMGGREENESEETKELTAEQQKEIKEGILKWEQVITKCVNTMVQSSSEKTEKSEKSSSKSSSSKSSSSKSSSFSSFQDTTGGSLGSPSESPASSPSGSSGSSFSSLVGSSESPASSSSGSTGSSFKDSTGGSLDTPAESPTRGASGSSSASGKVSMSGSGSQFQDTTGGSLGSSAESPNISPAASPLNSRSGSSSSKSASSITESSSAFKDTTGVTSLTSPAASPAGSPSGSITESGSSKSGSASSKSSSSGSASSKSSSSGSASSSKTETSSSSSTVEEVEKETSTQVMTFISNLEKKCPKNEEFNVFFEKLKGTMQVAFPGASKPSSPKSLISGMRAAAGKLSDAMAFVRSRIKGKTAEVKTSMEKYQEEVIKSLQELQDINSKIVRENQGKKQGSDLTVSTTQRTEIKQTITRWEQVTTQFVETAAQSESSSSSSSSSSSASSSSEKSSKSTAN

>AA_scaffold834_57

MERRSLAICLILLITASTVYEAQGHFLLKMYLRRKFSGRGRDFGGFACTGMAAFMANLQRSCPVNSEFKQFFGSFNTFISFIKSARSAKDESQVEGKADAICNSMGKMDSTTKSETRSTLISMGKTLISQKFSGSSMMTREQRREMVISLIKWFKVIGQFVKVAAEKSGKSIDLNSFGFDGIDASAASSSSSSSSGSSSDSSSSDESSSTKTGSSSTKTPTPETPSSSSSSGKSSSSGSASASGSVSSKKESSTSTTPTPETPSTPTETPTSDSASSSGKSSSSGSASASSKKESSTSTTPTPTPETPTATSSSGKSSSSGSASASGSVSSKKESSSSATPTPTPETPTASLSGAASGSASSSSSASSKSSSSKSASASASASSAGTVQQVENDCSNDVKSFISNLEKKYSSNTVLKGFFENVKSSIASSASNQKDFLGGLISAGGKIAEGMSSVGSQVAKSATAKSSVKSYQAEVMKTLQELQSLNSKIQSENQGKQQEAAQVSTTQQKEIKQTITRWEQVTTQFVETAAQSSSSSSSSSSSSSSSNSASAQSRKLLMAN

>AA_scaffold834_58

MTKTSSLGACLLLLVATSMVYEVQGTFLLKMYLKHFPKLSKDFEPFAFKGIQTFLDHLEKKCPMKPEFKVFFGKLRAYMLFINTAGSGSANFDAQLKVKAEELYESINGLGVKVGSDADTTALIENLISMGKILAEFKRSGSFKMSREQRRELIISIFKWAQVITKFVKAVAEKNGLNTSIDLSSLSLGDLDGILGGKDNDDDDDDDNDSKSSSGGGSSNTGSSSSGTGATGGSSANDSSSAGGATGGSSGTGSSSAGGATGGSSATDSTSAGGAKGGTSATDSSSAGGATGGSSATDSSSAGGATGGSSATDSSSAGGATGGSSATDSTSAGGAKGGTSATDSSSAGGATGGSSATDSSSAGGATSGSSATVSSSAGGATGGSSGTDSSSAGGAKGGSSATDSSSAGGATGGSSGATGGSSGTDSSSAGGAKGGSSATDSSSAGGATGGSSGTDSSSAGGATGGTSATDSSSAGGAKGGSSTTDSSSAGGATGGTSGSSDSSDMSASGAGSSASGSATGTGSGSAFSDSTGGASGGPSGSPTDASAGGSASSDTGASAGGSSSDSSTSGTSSMASKSASSGGSSSSGGSSSGGSISSVGGGSAETNSNGGPNSRTFKGKIGDISYEGAVSHEKKSTGEGTANHSTSETKSSHIKWNS

>AA_scaffold834_61

MSTRKANPNYTSTQRNIEKKVFTCSETKTMELFGGFDTRNRKQWQSHHPCFQNLLASLQTLNCVGSYGFSPITTRSHRFAQNRLNQTRFGSNLKKQFCDKTKLVLLFFESLSPNVVSGSGFPSAVNAPNTINDIQPYISNRAVGFVSNLENKCLIRDKLKSFFDKLKDLLRFESSVSDLIEKKESSSFNLELKSKADNLLKFMLMLRVRSGKFGLLSSSVRKEMFEVIKGLTQLHVAIGRVFLEKHITGGDGSMLLSGDQKSAIETAISTWELTITRVVKIVVEVISGSSSEVSTEESKTSDGSAVESGGLKSETTEEVNGENGGDSSTNESMENQGGNANSTEDKTENVEGGDNVSTGGTSTMENQGGNVNSTEEVNGNNNVSNENVTQENNVSGESASTVENQGGNVNSTEEVNGNNNNNVSTENVAQENNVSAESASTVESQGGNTNSTEVTDNVEPKEEIGEDDEKTKEAKLVEAQEIAKNMTEHESSENKESTNVTSEMGKEEDDEKAKEEKLVEAQEIAKNMTQHESSENKESTDGTSQVGKEEDDEKAKEEKLVEAQEIAKNMTQHESSENKESTNVNSKAETQEDENSKAETEEKDDEKAKEEKLVEAQEMAKKDENSKNEESKDVKSEVETKEKDDEKAKEEKLVGAQEMAKKDENSKNEESKDAETEEKDDEKAKEEKLVEAQEMAKKDESSKNKESNDAKSEAETEEKDDEKAKEEKLVEAQEMAKKDENSKNEESNDVQSEVKTEEKNDEKAKEEKLVEAQEMAKKDENSKNEESKDVKSEAETEEKDDEKAKEEKLVEAQEMAKKDENSKAEESNDVKSEAETEEKDDEKAKEEKLVEAQEMAKKDENSKNEESNDVKSEAETEEKDDEKAKEEKLVEAQEMAKKDENSKNEESKDSESKKDNNEKTHESNGEKSNKGKKECDGKKSANANTKKEDKKESKDNKKESKDDKKESEEDKSVEAKENKESSTEKKENKETNKETKEDKSQEEKEKEAKEKEEKEKEAKEKEEKEKKENAKKQKNKEAKEEKEKKERAKKEKKKEAKEEKEKKEKAKKEKKKEEKERKERAKKEKKEAKKEKKKEAKEEKEKKEKAKKEKKKEEKERKERAKKEKKECDKRKKEEEKKKENDEKNKKDSEKTEKKESKKETEENKESSTKKEQENVQGNGENSKSTGDKKEDKTKKEDSKENDKDSKKEKKKDSKEDDNSKNVKKEGDKKDSNDNKEKSQEKQEEGQGNSGESKKEEKKESKEEKSSKSKEKKECSSQKKDDKDEGKKEEKEDKSKKNEEEKKEKNEAKEEKSKENKKTENDQKENKESKSEETKKDNGDAEKQVNVEKSNEKKEDKESKEAKSKESEENEKESKKDNKEEKKDTKDSEKQKTSEKEKEEETQEKSEEAMEEKKEEENKDKDEKKVEAKVENENSTNNETTQMGDNNEESGKTEEAKVENENSANNETTQAGGSTEESGKTEEAKVENENAANNETTQAGGSTEEFEEAEVENGHSVNNETTQAGGSTEESGKTEEAKVENENSANNETTQAGGSTEESGKTEEAKLENEHSANNETTQAGGSTEESGKTEEAKVENENSANNETTQADGSTEESGKTEEPKVDNENSANNETMQADGSTEESGKTEEAKLENENSANNETTQASGSSEESETGKAEEAQIENGDSANNETTQASGSNEESTAGKTEEPKAENDNSANNETTQAGGSNEESTAGKTEEPKAENDNSANNETTQAGGSSEESANGETEETKLENGNSANNETMEAGESNANSTNTQTNEATSNSTTEIESTTSKEVKSFISNLEQKSPNTQEFQGFFQKLKDHMKFACPTSAKVEAKDSKSYMSEMVSTAKKLSESMAALQGTKSGSSEQMKTTLQGYQQEVMNNLQVMQSIMSKSMSEQENKNGGPLTLTPSAQQAIKDRVTKWEDGISQFVKVATESSSSSSSSNSMTSSSSSHTTGNENP

>g08612

MARVQLLLCFTILFASVTLLDLVSAHLKYKPSLPQIEDPKTVKDVEPYTVKVVMVFVADLEKECPKTSKFKAFFEKLRGFAKYVCPIRRRDQVDYDRDLKAKAGGVFKAISSFAIGKIREEIQEEKMEAINTFRFMKSVAAKIMGGRKKEESEETMKLTAEQQKEIKEGILKWETIITRITNTMVMSTTNSASGEESSVGKEVSSNNSKSSGSGSKTDSSSGEESNVGKEGSSNESKSSGSESKTKFSSGEESNVGKGGSSNNSKSSGKESETSAKGESETSAKGESETSAKGESETSETSAKGESETSAKGESETSAKGESETSAKGESETSSSKTAGGSSNVEASQSSSVTVTQVEEETSKDVSTFIMNLEKKCPQKEEFKVFFEQLKGTMIAPRKERKGLFSRIKSAAGKLSGAMAVMRSRIGSKSAEVKKNMEAYQEQVMTTLQELDTIHSQIVSQNKGKGSMTCTPAQQMQIKQTITKWEQVTTQFVEVAVQSESQSSASSSSSSSSSSGKLQAN

>g08653

MAKISLALGLLLLVALSEVYEVQGTFLFRHYLRKFPRRSRDFRPFACKGMLKFVDVLEVRCPLKPQYKSFFGNLRSYMNFINSASGSKNFDAELKGKAQGLLSAMSAMSGKGGASADSSKVMDTLLSMGKTLGNQQQSDSTVMSFGQRKEMIMSMAKWAQTIVQFVASAAAKSGNKIDISSLGIDGIDASAAAGAGDSTAAGSGTTTGGSSAAGSDTAAGGTTAAGGTTAAGAGTATGSGTVAGGYTATGSGTTTGAGTAAGDTTAAGTAAGGTTAAGTAAGGTATAGAGTAAGGTTAAGGTTAAGGTTAAGGTTAAGGVSGSYGTSGGSASNTNVGTNSCTKSACSNGGGASFKGAMNFQGKASSQSQQAASSQTSDGSS

>g08654

MLVVASSVIYEAQGTFLLKLYLKKNFPRKCNEFTPFANKGMMTLVTDLEGSSPATAEFKTFFSQFKSYMSFIETTSASTKNVDAEMTTKCDALFKAMSALSAGKGEKSAEAGGMKATLLSMGKTLVEQKKNTKIMTLKQKKELVIAMVKWTKMVATFVKSASEKKGKSINIASYGLDVDVNDSSIVGGAASSESSTKSGAVSTSGSVSTKTKESSSGSSANGSAATKSKESSGGSAARANFKDTTGKNLGSPSGSPKASPSGSVGGKSSSKESASAQGGASSQGSASAQGIVNGARAFSKNKETKTSSQRQSKSSSESSSSSTTTTTVKQVESETSKEVMSFITQLEKKYAAKSELKVFFESLKSSMQASASVGSKTAKDYVSASRAATGKLSEAMATVSSKNVKSAKMKSNLDTSKDELMKCVKQIQDINGKIVSGKTVSSAQQSELKQTITKWEKVTTQFVETAASSRSSSSSSSSSSSAASQQQGNAGMVKTN

>g08656

MAKVQLFLCFTILFASVTLLDFVSAHLKLKPTLPQIEPPQTVKDVEPYTVKVVMVFVSDLEKECPKTNKFKAFFEKLRAYAKYVCPIKRKDQVDYDRDMKAKAGGLFQTISSFAIGKIKKEIQEEKMEVINTFKFMRFLAAKIVGSRKKDESEESMKLTAEQQKEINEGILRWETIIARITNTMVMSTKNSSSSENSTTGKKASSDSSKKGSSSEDTKGANTNKGGSPSSSPSGSSSSAKGESETSSSKTTGGNSTTATKKEISASGNVGASQSSGVTVEQVEEETSKDVSTFIMNLEKKCPQKEEFKVFFEQLKGTMTAPAKERKGLFSRIKSAAGKLSGAMAVMRSRIGSKSAEVKKSMETYQEQVMKTLQELDTIHSQIVSQNKGKKEGSLICTPAQQTQIKQTITKWEQVTTQFVETAIQSETKSSSTTSSSVGKMNTN

>g08657

MAKNLLAICLVFMVVSSVVYEVQGTFLLKLYLRRKFPRRCVDFAPFASKGMLMLVSNLEGGCPATREFKQFFSTFKSYMSFISSASISSSKNIDVEMNGKCELLAKAMSALTGSRSSQSSELKITMLSMGKTLIEQKRQGSRIMSLKQKKELVVAMVKWTRIVVTFVKSVAEKRGKSIDESSYGLDVDVNASIGSGSESGGSSSSDNESSSNTKSQGSSSKSGSESKTEAGSTETKTGSVGSKTEAGSGSSANTYKDTTGSSSGPSPSGSPTPTPSTPTPSTPTPSTPTPSTPTPSSSTPAGGKTSEKGSESSSGSASTKKESKSKSESESAASKTKESSSGGTYKDTTGTSSGSPSGSPSGSPSPSTSTDGKTSSKGSASSGASANAEASASASAGANASAGESSQKKESNSKSSTSSSSTTSVKEVESQTSSEVSSFISNLEKKYTGNAELKVFFDKLKTSMSASSKLTASNAKEFVSGMRSAASKLSEAMMFVRSRFSKSEETKTSMESCQQQVMKSLQELQDINSQIVSGKTVTSTQQTELKQTITKWEQVTTQFVETAASSSSSSSQSSAGMAMKN

>g08658

MARFRIAICLMLILVASSTIYEAQAGFLLRHYMKKFPQNSQDFEPFAYKGMLSFVDNLESMAPGKGEYKDFFSKLKAFMGFINTAKGSSSDFQSQMKQQSEGLFKAISALGIKGGSSADTSKLIESLMSMGKTFAEFKRSGATTMTSEQRRELVTSMAKWAQVIGQFVKKVGEQNGDGKNIDLSSLLGGGSSGFGSGGDSGSPSSDSGSPSADTGSPTDSGSYGDSTGDTGSSASSPSYPSDGGSGSTAGGPSGSTTDDGSSAGGESSMGGDSSSAVGGAAGETASATDADSGGAAGGEAASGGAASGGSAETGGESASGGDASGGSAETGGESAGGGAASGGSAETGAESGGAASGASGGAA

>g32274

MAKISLAICLTLLVTLSTVYETQGTFSLPLYLKNFPKVGKDYEAFANKGISDFLGELEGMCPKTAEFKDFFEKLKDYMASFNSASPGSKDNLFEMSVKSEKLFRAMSAFNSSNGGTSEDSWKLVDGLLSMGKGLVEMKKSGSKEITFEQRRDLISSMVKWARAIGLFVKAASEDKGQSIDLASFGIDYDNHVESPFSKRAMYEKQGTFSLPHYLKNAPKMGKDIEPFAYNGMSDFLGSLESKCPATPEFQDFFVKLEDYMACFKLVSPESKDDMSVKSEKLFRAMILLDGTKGGTSVDSWRMLDGMLSMGKVLVEMKKSGSKEITFEQRRDLISSMVKWARAIGLFVKTASGNKGKPIDLAPFGIDYENNVGNGKGNYRTGPVLIDDGSEL

>AL5G18500

MFEVMKSLTELHAAIGRVIIEKHIKGDESMSLSLEQKNAVENSISEWEQTITRIVKIVVEVKSKSSSEASWKESSTTEHNNVSMNGNMVATNGENSESTQEKLDGVKGSNGGDVSMETQEGNKVDDLKEGNTVFENGETKEINGENAESNNEKAVEGQGESIGDSAIDKNLESKEDVKSEVEDKSDGSSMTENLEEVQRNNGVSTKDTNLENKGSEVESKDDKMVNATTNDEDNTKENREGTQENNGESLKDENLENKGSEGESKDDKMVNATTNDADHTKENKEETQENNGESLKDENLENNAGNNELKGNGSVEAKTNNEISMEAKREETQRSNEVYMNKETTKGENADFNKEKESNIQGESIGDSTKGNILEDKEDVKPEVDSNVSDGSSTKERHQEAQGNDKVSTKDKNLDNIGADEERKYDKSVKVMTNDEGHTKEMREETQGNNGESVKNENLENKENKKELKDDGSVDEKTNNESSLEEKGEQTQRGHDNSVNSKLVENKGGNAYSNKEKKVHVGDSTNDNNIESKAKSEAEVKKNDGSSMKEKGDEVQGNNIESMEDKNLENKESHTDLKDEKSIADKQNVRTTMKDKQEESQINVGESKDDKSVEAKGNTKESKDDKSVEAKGNTKESKESKWTKTNENKVRNKEENVHQGNKNESKKVEKGENKESKDAKLVEAKETKKLSRKENREEDNGGSTKIKTKKTTKEDRDSANNMDIDVQKGSGESVKYKKDGKKEGNKEEKKDTINTNSKKKEKDKKKKKGSQNSNMKKKQENEREKKEYEDSKLKKKEEDRKEKEEYVNNELKKKEDNKKKMKESESSNLKEKNKDNKEKKDSGDSKSKNRKEKKYEDNIKDDNKAKKKNSKDYKLKRKEEDKKKEYENTKSKKEEENKKEDKEYKSSKLKEKNKDNKEKNESKDSTSKNKEKKEYKDNKMKKEYEDSKSKTKEEAKKEKIYSKEYKSKRKEEDKNEKNKSEDKKREEKDSEDRKSKKAKEKKQEKEESKDLKGKKKENEKMEKKELENHKSKKKEKNVENKKEHEDNKSMKKEEDKKRKKEHEDNKSRTKEEDKKDIGKLEDHKSNENKEEHEDKISKKNIEEKKKKKKFQHGKSMKKESDKKKKKEKEEKNETKEIENSKSQNNEVDKKGKKSSKDQQKKKEKETKESEEKYPKKNEEDRKKQTSVEEHKSKTTGENKKQKETEEAKNKSKEDKKNTKTQSGGTNQVGNQSETENSMKRKEDKKESKDSDSKEANNQQKSHGQEKKVGVQVNSEKDTKKTKEDVNVSIDGKSKDHEKGTQTTTQGDSEESKNEMQADTQAKIGESLKHKKVKGEEDNGDEVGKEKGRRSKHGKEVQIEEGKDGSIEEGSKGVKTIENIGGKEDSENDKVIEHNKANEDSIEGGSKDGKTTEINASTKESMKKDLEDGKTVEINKGKNDSQGESSEDATTVETSGSKNHTEEGYKLDKTSDINARKEDYKKDGSKDGKIVEISRGTNSTGEFSKDGKTMENSGGKLDSMEESSKDSNIVETNGGKEGSEDGKTIETNGGKEVSTEESSKDGKIEEVNEGQENSTKKNYEDGKTNDIHGGKEVTMEEGSKVDKKIEINGGKEYAVEKNSEDGEITNLNGGTNSTGKDSKDSKSVEINGVKDDSLKEDSKNGNINEINNGKEDSVKDNVTEIQENGNILTNSTSMEPNGDKLDINEDSMKNKTMEAQGNSNGDSTNGETEETKESNISMNNQNMQDVGSNANSMNDQTAGDDVISTTKEETRTDIESNTSKEVTSFISNLEKKSPGTQEFQSFFQKLKDYMKYVCPVSSTFEAKDSRSYMSEMISMATKLSDAMAVLQAKKSGSGQMKTTLQGYQQEVMKTLTILQSVMGKAVSEQQSKNSGSLTLTLSQQQAIKGIVLKWEQVMSQFVKIATESEKQFSLEISTENGYHMKKSSNSSSSSSSSSSSSLDFKHNSENLKGVDMNG

>AL5G18520

MAKFPIAVCLMLILVASSTIYEAQAGFLLRHYMKKFPQNSQDFEPFAYKGMLSFVDNLESMAPGKGEYKDFFSKLKAFMGFINTAKGSSSDFQSQMKQQSEGLFKAISALGIKGGSSADTSKLIESLMSMGKTFAEFKRSGATTMTSEQRRELVTSMAKWAQVIGQFVKKVGDQTGDGKNIDLSSLLGGGSSGFGSGGDSGSPSSDSGSPSADTGSPTDGGSYGDSTGDTGSSASSPSYPSDGGSGSTAGGPSGSTTDDGSSAGGESSMGGDSSSAVGGAAGETASATDADSGGAAGGEAASGGASGGSAETGAESASGGDASGGSAETGGESAGGGAASGGAAETGGESGGATTGGSAETGAESGGAASGGSAETGDESGGAASGGSAEAGAESGGATSGGSAETGAESGGAASGGSAETGGENASGGAASGGSAETGAESGGAASGGASGGAASGGSAETGAESGGAASGGASGGAASGGSAETGAESGGAASGGASGGAASGGSAETGGESASGGATSGGSAETGGESASGGAASGGSTETGGGAAAGGASETTSESASGGSAAAGGASGTATETSNSQGSSMASGGTYTDSTGGSPAGSPSAGGPSGSATESSMEGGASGGQSMGGQAGSVSYQSANYQKTHSKSAGKSSFSHSSEEKSSGSANADS

>AL5G18530

MALELFKSGSESKTEAGSTETKTGSVGSKTEAGSGSSSSAKTKETSGGSSGNTYKDTTGSSSGASPSGSPTPTPSTPTPSTPTPSTPTPSSSTPSGGKTSEKGSESSSGSASTKKESKSKSESESAASKTKESSSGGTYKDTTGTSSGSPSGSPSGSPSLSTSTDGKTSSKGSASSGASANAEASAGANASAGESSQKKESNSKSSTSSSSTTSVKEVESQTSSEVSSFISNLEKKYTGNAELKVFFDKLKTSMSASSKLTASNAKEFVSGMRSAASKLSEAMMFVRSRFSKSEETKTSMESCQQQVMKSLQALQDINSQIVSGKTVTSTQQTELKQTITKWEQVTTQFVETAASSSSSSSSSSSSQSSAGMAMKN

>AL5G18540

MARVQLFLCFTILFASVTLLDVVSAHLKLKPTLPQIEPPQTVKDVEPYTVKVVMVFVSDLEKECPKTNKFKAFFEKLRAYAKYVCPIKRKDQVDYDRDMKAKAGGLFQTISSFAIGKIKKEIQEEKMEVINTFKFMRFLAAKIMGSRKKDESEESMKLTAEQQKEINEGILRWETIIARITNTMVMSTKNSSSSEDSTTGKEASSGSSKKGGSSEDTKGANTNKGGSPSSSPSGSSSSAKGESETSSSKTTGGSSTSATQKESSASGNVGASQSSGVTVAQVEEETSKDVSTFIMNLEKKCPQKEEFKVFFEQLKGTMTAPAKERKGLFSRIKSAAGKLSGAMAVMRSRIGSKSAEVKKSMETYQEQVMKTLQELDTIHSQIVSQNKGKKEGSLTCTPAQQTQIKQTITKWEQVTTQFVETAIQSETKSSSTTSSSVGKMNTN

>AL5G18560

MARISLGICLMLVVASSVIYEAQGTFLLKLYLKKNFPRKCNEFTPFANKGMMTLVTDLEGSSPATAEFKTFFTQFKSYMSFIETTSASTKNVDAEMTTKCDALFKAMSALSAGKGEKSAEAGGMKATLLSMGKTLVEQKKNTKIMTLKQKKELVISMVKWTKMVATFVKSASEKKGKSINIASYGLDVDVNDSSIVGGAASSESSTKSGAVSTSGSVSTKTKESSSGSSSSGSVDTKSKESSGGSAATKSKESSGGSAAGANFKDTTGKNSGSPSGSPKASPSGSVGGKSSSKESASAQGGASSQGSASAQGIVNGARAFSKNKETKTSSQRQSKSSSESSSSSTTTTTVKQVESETSKEVMSFITQLEKKYAAKSELKVFFESLKSSMQASASVGSKTAKDYVSASKAATGKLSEAMATVSSKNVKSAKMKSNLDTSKDELMKCVKQIQDINSKLVSGKTVSSTQQSELKQTITKWEKVTTQFVETAASSSSSSSSSSSSSSAASQQQGNAGMVKTN

>AL5G18590

MAKISLALGLLLLVALSEVYEVQGTFLFRHYLRKFPRRSRDFRPFACKGMLKFVDVLEVRCPLKPQYKSFFGNLRSYMNFINSASGSKNFDAELKGKAQGLLSAMSAMSGKGGASADSSKVMDTLLSMGKTLGNQQQSGSTVMSFGQRKEMIMSMVKWAQTIGQFVASAAAKSGNKIDISSLGIDGIDANAAAGAGDSTATGTGSSGATTGGSTTAGSDTAAGGTTAAGAGTAAGGYTATGSGTAAGTGTTTGAGTAAGSGTAAGGYTTTNSGTAAGAGTAAGGTTAAGPGTAAGGTTAAGAGTAAGGTTAAGAGTAAGGTTAAGSVSGSYGSSGGSASNTNVGTNSCTTNACSNGGGASFKGAMNFQGKASSRSQEAASTQTSDGSS

>AL5G18840

MARVQLLLCFTILFASVTLLDLVSAHLKYKPSLPQIEDPKTVKDVEPYTVKVVMVFVADLEKECPKTSKFKAFFEKLRGFAKYVCPIRRRDQVDYDRDLKAKAGGVFKAISSFAIGKIREEIQEEKMEAINTFRFMKSVAAKIMGGRKKEESEETMKLTAEQQKEIKEGILKWETIITRITNTMVMSTTNSASGEESSVGKETSSNNSKSSASGSKTGSSSGEESNVGQEASSNNNKSSGKESETSAKGESETSTKGKSETSAKGESETSAKGKSETSAKGESETSAKGESETSAKGESETSSSKTAGGSSNVEASQSSSVTVTQVEEETSKDVSTFIMNLEKKCPQKEEFKVFFEQLKGTMIAPRKERKGLFSRIKSAAGKLSGAMAVMRSRIGSKSAEVKKNMEAYQEQVMTTLQELDTIHSQIVSQNKGKGSMTCTPAQQMQIKQTITKWEKVTTQFVEVAVQSESQSSSSSSSSSSSSSGKLQAN

>AL7G50270

MPRFQLFFCFTILVATITFFNVASAHVKIKPALPQIEDPSTVKDVESYTIKVVTTFLVDLEKECPKTEKFKVFFEKLKAYSKYVCPISKAKGYESDMKAKAGSLFEAMSALGSVKNRSREGSVTKSLQRGKTEAMNTVKLLQSIGEKIAGGRNNKTEINGTAKLTIEQQKEIKDGILKWLQVITQIAKTTEEINSKSSLKSQTMQESREEKSSTQIKRGSQRENAQITALPRGSRVTKVGNN

>AL8G21560

MEIINTFKFTRSVAGKIIGGRKKEEGEESMKLTTQQQKEIKEGILKWETVITRITNTIVVSTTNSSSMENSTRRKRSSSGSISKSSGSFNSGNTLTDTTGKAVPYLGFETMVYGLSSFRIVN

>AL8G38440

MGKISLAVCLTLLVTLSTVYETQGTFSLPLYLKNFPKVGKDYEAFANKGISDFLGELEGMCPKTAEFKDFFEKLKDYMASFNSASPGSKDNLFEMSVKSEKLFKAMSAFNSSNGGTSEDSWKLVDGLLSMGKGLVEMKKSGSKEITFEQRRDLISSMVKWARAIGLFVKAASEDKGQSIDLASFGIDYDNHVESPFSKRAMYEKQGTFSLPHYLKNAPKMGKDIEPFAYNGMSDFLGSLESKCPATPEFQDFFVKLEDYMACFKLVSPESKDDMSVKSEKLFRAMILLDGTKGGTSVDSWRMLDGMLSMGKVLVEMKKSGSKEITFEQRRDLISSMVKWARAIGLFVKTASGNKGKPIDLAPFGIDYENNVGNGKGNYRTGPVLIDDGSEL

>AL8G38480

MGKISLAVCLTLLVTLSTVYETQGTFSLPLYLKNFPKVGKDYEAFANKGISDFLGELEGMCPKTAEFKDFFEKLKDYMASFNSASPGSKDNLFEMSVKSEKLFKAMSAFNSSNGGTSEDSWKLVDGLLSMGKGLVEMKKSGSKEITFEQRRDLISSMVKWARAIGLFVKAASEDKGQSIDLASFGIDYDNHVESPFSKRAMYEKQGTFSLPHYLKNAPKMGKDIEPFAYNGMSDFLGSLESKCPATPEFQDFFVKLEDYMACFKLVSPESKDDMSVKSEKLFRAMILLDGTKGGTSVDSWRMLDGMLSMGKVLVEMKKSGSKEITFEQRRDLISSMVKWARAIGLFVKTASGNKGKPIDLAPFGIDYENNVGNGKGNYRTGPVLIDDGSEL

>AT3G28780

MARFPIAICLMLILVASSTIYEAQGVFLLRHYMNKFPKNSQDFEPFAYKGMLSFVDNLENMAPEKGEYKDFFSKLKAFMS

FINTAKGSSSEFQSQMKQQAEGLFKAISALGVKGGSSADTSKLIESLMSMGKTFAEFKRSGATMMTSEQRRELVTSMAKW

AQVIGQFVKKVGDQNGDGAKIDLSSLLGGGSSGLGSSGDSGSPGSDSGSPSADTGSPTDGGSYGDTTGDSGSSAGSPSYP

SDDGSGSTAGGPSGSTTDGSAGGESSMGGDSSSAGAAGESGSAATADSGDAAGADSGGAAGADSGGAASADSGGAAAGET

ASGGAAAADTSGGSAETGGESASGGAASGAGAASGASAKTGGESGEAASGGSAETGGESASAGAASGGSAETGGESGSGG

AASGGESASGGATSGGSPETGGSAETGGESASGGAASGGESASGGAASSGSVESGGESTGATSGGSAETSDESASGGAAS

GGESASGGAASGGSAETGGESTSSGVASGGSTGSESASAGAASGGSTEANGGAAAGGSTEAGSGTSTETSSMGGGSAAAG

GVSESSSGGSTAAGGTSESASGGSATAGGASGGTYTDSTGGSPTGSPSAGGPSGSASESSMEGGTFGGQSMGGQAGSASY

QSTNYQKTHSKSAGKSSVSHSSEEKSSDSANADS

>AT3G28790

MAKNLLAICLVFMVASSVVYEVQGTFLLKLYLRRKFPRRCIDFAPYAGKGMLMLVSNLEGGCPATREFKQFFSTFKSYMS

FISSASISASKNIDVEMNGRCELLSKAMSALTGSKSSQSSELKMTMLSMGKTLVEQKRQGSRMMSLKQKKELVVAMVKWT

RMVITFVKSVAEKRGKSIDESSYGLDVDVNASIGSSSGSDGSSSSDNESSSNTKSQGTSSKSGSESTAGSIETNTGSKTE

AGSKSSSSAKTKEVSGGSSGNTYKDTTGSSSGASPSGSPTPTPSTPTPSTPTPSTPTPSTPTPSTPTPSTPAPSTPAAGK

TSEKGSESASMKKESNSKSESESAASGSVSKTKETNKGSSGDTYKDTTGTSSGSPSGSPSGSPTPSTSTDGKASSKGSAS

ASAGASASASAGASASAEESAASQKKESNSKSSSSSSSTTSVKEVETQTSSEVNSFISNLEKKYTGNSELKVFFEKLKTS

MSASAKLSTSNAKELVTGMRSAASKIAEAMMFVSSRFSKSEETKTSMASCQQEVMQSLKELQDINSQIVSGKTVTSTQQT

ELKQTITKWEQVTTQFVETAASSSSSSSSSSSSSSSSSQGSAKMAMKN

>AT3G28770

MRKVYLFFVCCIVATSLTLNVVLAHETVVNPPNTIKDIEPYISDRALGFVLKLENNCPIREQLRSFFEKLKDLLKLESSV

TPLIEDNEPKTFKFDLKSKSENLLQTMFMLGRGLLSSSIRKEMFEVMKSLTELHAAIGRVIIEKHIKGDEAMSLSLEQKN

AVETSITQWEQTITRIVKIVVEVKSKSSSEASSEESSSTEHNNVTTGSNMVETNGENSESTQEKGDGVEGSNGGDVSMEN

LQGNKVEDLKEGNNVVENGETKENNGENVESNNEKEVEGQGESIGDSAIEKNLESKEDVKSEVEAAKNDGSSMTENLGEA

QGNNGVSTIDNEKEVEGQGESIEDSDIEKNLESKEDVKSEVEAAKNAGSSMTGKLEEAQRNNGVSTNETMNSENKGSGES

TNDKMVNATTNDEDHKKENKEETHENNGESVKGENLENKAGNEESMKGENLENKVGNEELKGNASVEAKTNNESSKEEKR

EESQRSNEVYMNKETTKGENVNIQGESIGDSTKDNSLENKEDVKPKVDANESDGNSTKERHQEAQVNNGVSTEDKNLDNI

GADEQKKNDKSVEVTTNDGDHTKEKREETQGNNGESVKNENLENKEDKKELKDDESVGAKTNNETSLEEKREQTQKGHDN

SINSKIVDNKGGNADSNKEKEVHVGDSTNDNNMESKEDTKSEVEVKKNDGSSEKGEEGKENNKDSMEDKKLENKESQTDS

KDDKSVDDKQEEAQIYGGESKDDKSVEAKGKKKESKENKKTKTNENRVRNKEENVQGNKKESEKVEKGEKKESKDAKSVE

TKDNKKLSSTENRDEAKERSGEDNKEDKEESKDYQSVEAKEKNENGGVDTNVGNKEDSKDLKDDRSVEVKANKEESMKKK

REEVQRNDKSSTKEVRDFANNMDIDVQKGSGESVKYKKDEKKEGNKEENKDTINTSSKQKGKDKKKKKKESKNSNMKKKE

EDKKEYVNNELKKQEDNKKETTKSENSKLKEENKDNKEKKESEDSASKNREKKEYEEKKSKTKEEAKKEKKKSQDKKREE

KDSEERKSKKEKEESRDLKAKKKEEETKEKKESENHKSKKKEDKKEHEDNKSMKKEEDKKEKKKHEESKSRKKEEDKKDM

EKLEDQNSNKKKEDKNEKKKSQHVKLVKKESDKKEKKENEEKSETKEIESSKSQKNEVDKKEKKSSKDQQKKKEKEMKES

EEKKLKKNEEDRKKQTSVEENKKQKETKKEKNKPKDDKKNTTKQSGGKKESMESESKEAENQQKSQATTQADSDESKNEI

LMQADSQADSHSDSQADSDESKNEILMQADSQATTQRNNEEDRKKQTSVAENKKQKETKEEKNKPKDDKKNTTKQSGGKK

ESMESESKEAENQQKSQATTQADSDESKNEILMQADSQADSHSDSQADSDESKNEILMQADSQATTQRNNEEDRKKQTSV

AENKKQKETKEEKNKPKDDKKNTTEQSGGKKESMESESKEAENQQKSQATTQGESDESKNEILMQADSQADTHANSQGDS

DESKNEILMQADSQADSQTDSDESKNEILMQADSQADSQTDSDESKNEILMQADSQAKIGESLEDNKVKGKEDNGDEVGK

ENSKTIEVKGRHEESKDGKTNENGGKEVSTEEGSKDSNIVERNGGKEDSIKEGSEDGKTVEINGGEELSTEEGSKDGKIE

EGKEGKENSTKEGSKDDKIEEGMEGKENSTKESSKDGKINEIHGDKEATMEEGSKDGGTNSTGKDSKDSKSVEINGVKDD

SLKDDSKNGDINEINNGKEDSVKDNVTEIQGNDNSLTNSTSSEPNGDKLDTNKDSMKNNTMEAQGGSNGDSTNGETEETK

ESNVSMNNQNMQDVGSNENSMNNQTTGTGDDIISTTTDTESNTSKEVTSFISNLEEKSPGTQEFQSFFQKLKDYMKYLCP

VSSTFEAKDSRSYMSEMISMATKLSDAMAVLQAKKSGSGQMKTTLQGYQQEVMKTLTILQSVMGKAVTEQQSKDSGSLTL

TLSQQQAIKEIVLKWEQVMSQFVKIATESEKQFSLEISTENGYHMKKSYNSSSSSSSSSSSSSRSDLKLNGEHLKGMGMN

G

>AT3G28840

MAKLSLALGLLLLLALSEVYEVQGSFLLRHYMRKFPRRSRDFRPFACRGMLKFVDVLELKCPLKPQYKSFFGNLRSYMNF

INSASGSKNFDAELKGKAQGLFSAMSAMSGKGAASADSSKVMDTLLSMGKTLGTQQQSGSSVMSLGQRKEMIMSMAKWAQ

TIGQFVVSAAAKSGNKIDISSLGIDGIDASAAGAGDSTASGGVSATGTGSYGAGAGGSSASGSDTAAGGTTATGGTTAAG

GSTAAGGTTASGAGTAGYGATAGGATASGAGTAAGGTTASDAGTAAGTTASGAGTAAGGTTAAGAGAAAGAGAAAGAGAA

AGGTTAAGGVSGSYGSSGGSASNTNGGTSSCGTSACSNGGGASFKGAMNFQGKASSQSQQAASSQTSNGSS

>AT3G28830

MARISLGICLMLVVASSVIYEAQGTFLLNHYLKKNFPKKCNEFTPYANKGMITLVTDLEGSSPATTEFKTFFTQFKSYMS

FIETTSASTKNVDAEMTAKCDGLFKAMSALSASKGVKSADAGSMKMTMLSMGKTLVEQKKNTKIMTLKEKKELVIDMVKW

TKMVATFVKSASEQKGKSINIASYGLDVDVNDSSIVGGAASSESSSTKSGSVSSSGSVSTKSKESSSSGSSASGSVATKS

KESSGGSAATKSKESSGGSAATKSKESSGGSATTGKTSGSPSGSPKASPSGSVSGKSSSKGSASAQGSASAQGSASAQGS

ASAQGSASAQRRESGAMAMSKSRETKTSSQRQSKSSSESSSSSTTTTTVKQVESETSKEVMSFIMQLEKKYAAKAELKVF

FESLKSSMQASASVGSKTAKDYVSASKAATGKLSEAMASVSSKNVKSAKMKSNLDTSKDEMLKCVKQIQDINGKMVSGKT

VSSTQQSELKQTITKWEKVTTQFVETAASSSSSSSSSSSSSSSSAASQQQGNAAMVKTN

>AT3G28820

MARVQLLLCFTILFASVNLLDVVSAHLKLKPTLPQIEPPQTLKDVEPYTVKVVMVFVSDLEKECPKTNKFKTFFEKLRAV

AKYVCPIQRKDQVDYDKDMKAKAGGLVRAISSFAIGKIKKEIQEDKMEVINTFKFMRFLATKIVGSRKKEESEESMKLTA

EQQKEIKEGILRWETIITRITNTMVSSTTNSASSNEESSVGKEASSENSKSSGKESESSAKGESETSAKGESKTSAKGES

ETSSSKSAGGSSTSATKEESSASQSSGVTVTQVEEETSKDVSTFIMNLEKKCPQKEEYKVFFEQLKGTMIAPPKERKGLF

SRIKSAAGKLSGAMGVIRSRIGSKSAEVKKNMEAYQEQVMKTLEELDTIHSQIVSQTKGKQEGSLTCTPEQQTQIKTTIT

KWEQVTTQFVETAIQSETQSSSTTSSSVGKMNTN

>AT3G28980

MARVQLLLCFTILFASVTLLDLVSAHLKFKPSLPQIEDPKTVKDVEPYTVKVVMVFVADLEKECPKTNKFKAFFEKLRGF

AKYVCPIRRIGQVDYDRDMKAKAGGIFKAISSFAIGKIREEIQEEKQEAIDTFRFMKSVAAKIMGGRKKEENEETMTLTA

EQQKEIKEGILKWETIITRITNTMVSSTSTSASSSEESSVGKESETSAKGESETSAKNESKTSAKGESETSAKGESKTSA

KGESETSAKGESETSAKGESETSSSKTAEASQSSSLTVTQVEEETSKDVSTFIMNLEKKCPQKEEFKVFFEQLKGTMIAP

RKERKGLFSRIKTAAGKISGAMQVMRSRIGSKSAEVKKNMEAYQEQVMKTLEELDTIHAQIVSQNKGKASLTCTPTQQVQ

IKQTITKWEQVTTQFVEVAAQSESSASSSSSSSSSSSSSGKMQAK

>AT3G28810

MARVQLFLCFTILFASVTLLDIVSAHLKLKPTLPQIEPPQTLKDVEPYTVKVVIVFVSDLEKECPKTNKFKAFFEKLRAF

AKYVCPIKRKDQVDYDRDMKAKAGGLFQVISSFAIGKIKKEIQEDKMEVINTFKFMRFLATKIVGSRKKEESEESMKLTA

EQQKEIKEGILRWETIITRITNTMVSSTTNSASSNEESSVGKEASSENSKSSGKESESSAKGESETSAKGESKTSAKGES

ETSSSKSAGGSSTSATKEESSASQSSGVTVTQVEEETSKDVSTFIMNLEKKCPQKEEYKVFFEQLKGTMIAPPKERKGLF

SRIKSAAGKLSGAMGVMRSRIGSKSAEVKKSMETYQEQVMKTLQELDTIHSQIVSQNQGKKEGSLTCTPAQQTQIKTTIT

KWEQVTTQFVETAIQSETKSSSTTSSSVGKMNTN

>AT5G48575

MIFIINLEKKCPPKEEYEVFFEKLKGIMAARSKNSPKSKKSFFSTIKYAAGKLSDDMTFMRSRIGTKSAKVKQSMETYQK

EVKTIKELETIHSRIVS

>AT5G39870

MPRFQHFLCFTILVATITFFKVASAHVKIKPALPQIEDPLTVKDVESYTIKVVTTFLVELEKECPKTEKFKVFFEKLKAY

SKYLCPVSKAKGYKSDMKAKAGSLFEAMSALISVKHRSKEGSVNKSLQREKMEAMNTINLLQSVGEKIAGGRSNKTETNG

VAKLTVEQQKEMKDGILKWLQVITRIVKTNVEINLKSSSKSQTTQESKEEKSSTQTQIKRRSEREYAQITALPRGSRVTN

KGNINGILKSARNSPKEIDDHGNKRRKPKKQYKFKEGDEPKQIIKKKFQN

>AT5G61720

MGKISLAICLTLLVTLSTVYETQGTFSLPLYLKNFPKLGKDYEAFANKGISDFLGDLEGMCPKTAEFKDFFENLKDYMAF

FNSAAPGSKDNQFEMFVKSEKLFKALSAFNSSKGGTSEDSWKLVDGLLSMGKGLVEMKKSGSKEITFEERRDLISSMVKW

ARAIGLFVKAASEDKGQSIDLASFGIDYDNNVESPFSKRAMYETQGTFSLPHYVKNTPKMGKAIEPFAYKGMSDFLGSLE

SKCPATPEFKDFFVKLEDYMACFKLVSPESKFEMAVKSDKLFTAMISLDGTKGGSVDSWRMLDGMLSMGKVLVDIKKSGS

KEITFEQRRDLISSMVKWARAIGLFVKAASERKGKSIDLAPFGIDYENNVGNGKGNFRTGPVLIDDGSEL

>Aa_G138650

MKAVEELDTIYAQIVSQNKGKKEGTLTCTPEQQKQIKLTITKWEQVTTQFVETAVQTESSSSSSSSSS

>Aa_G138660

MARIQLFLCFTILFASTALLDVVSAHLKIKAALPQIEDPKTLNDVEPYTVKVVMVFVSDLEKQCPKTSKFKAFFEKLRACAKYICPIKRNNQNEDYDRDMKAKAGSLFQTIASFAIGKIKEEIQEEKMEAIETFRKMKSLAGRIIGSRKKDENEESMNLTVEQQKEIKEGLLKWETVITRIANTMWKSIYQQIWXXXXNSGSSFKDTTGSSSDVGSPSGSPSTSKSGSISFKDTTGGNSISSSGSPTSSPSSSTPGTSENSISPTASSTSSTKSESAEGSQNSALTVTEVEAETSKEVMTFIMNLEKKCPPKEEYKSFFEKLKGTMTAKATASVKAKRSFFAGIKSGAQKISGAMALLRSRIGSKSAEVKSSMENYQTEVMKAVEELDTIYAQIVSQNKGKKEGTLTCTPEQQKQIKLTITKWEQVTTQFVETAVQTESSSSSSSS

>Aa_G142480

MFTIGRGLLSSSVRKEMFQVTKSLTELHAAIKKVIMERHITGDGSMSLSLEQKNAIENAVSQWEVTITRIVKIVVEIKSGGSSETSGEESNTSGDSTIDKSLESKEDVKSEVEANGGSSTTEEGLEKAQGNHEVSTNDNNLENKESEEEVQDIKTAQAPTNDGNVETKTNHEMSMEEKREEANHTFSMNENLENKESEEKAQENGESEKNENLENKESEEEAQDVKTAEAPTNDVNGTIEKPEETSENNGEAVKNENLENKESKEEAQNVKTAEAPTNDVNEKPEETSENNGEAVKNENLENKESKEEAQNVKTAEAPTNDVNGTMEKPVETPESNGESSMKNEKLENNVENKELKEHGSVETKTNHEMSMEEKREHANHTFSMNGEAIDTKGGDSNKEKEVEAQGKSIGDSTKDKKLENNDVKQEVEAKENDASITEEEKKEEAQGTDEASSKDIGANTETKDNKAVETMKNEGADTKEKTQENNGESVNDENLEKIEEKNEMKEGSVETNTNNKVSMDKKRDGAQGNNEVSVNNNSNKENHIEVQGGNIEDSTKDKSLKSKETDKAKVEAKEISGNSTNEVSMNDKNLEKEGGEKELKDDKLVEAKTGKETSTKVKEEESQGTRGNSTEVETKGGKEESKDDTSAEEKENARSSMNEKQEEAQDGGDKKESKDGKSVEAMEKNKESKDNKRSEVNENQGSSTEEGQPEVQGNNKESKKVENKGDNKLVEVKENKESSMKENQEETQGSNSGDLGKVNKKEGEEEPKDYKSVEAKEDKKSSAKENRKEGQGNGEVDKKNSKDKKMVEAKENEKSSTKKKQEEAQGSSRDTTKVDGSDSKDTKLSENTEKDKGTTENMATEVQNGSGESMQDNKEEKKDSTDGKSGELEKETQVEAQTKNGESVEDKKGEGKEDDDKYEGGKENDEDSTEESGESIKNDKVEENKEEDNEDSSMKKNEELQEAAAASNMVEQKHIDAAQVNVGDSTKVETKESNGDSMNGKSVAVQEESENSKDGQTVETNQGKNDSMEEGSKDGKTVGNNGEENKAEDSSMKKNEELQEAAAASNMGEKKDQKDSIDDKSKETKESDGNSVEQKHIDAAQVNVGDSTKEETKENNGDSMNGKTVAIQEENENSKDGQTVETNQGKNDSMEEASKDGKTTEIKRGKEDSIEEKSKDSKTVENSGGKEDSKDVKENEINQGKEDSKDVKANEINQGKEDSMEEGSKDSKIVEHNGTKEDTMEQGSKDGKTVENSGGKEDSKDAKANEINQGKEDSMEEGSKDNKIVEHNGTKEDIMEQGLEDGKTVENNEGKEDATEKDFEDGKKVENNERKEDSMEEGSKEGKIDEISGGKNSTEEGSKDGKIAEMNGGNEDFKDGKTVETNEGKNSTEEMSKDVQASEGIEGKDGSVEEGSKDGNVVENSQKKEHSLEEGSKDSKSVEVKEGQEESMEKDFKGDKIDINADSINNNTAEAQGGIIGDLTNGKTGETKESNDSMNNQIVQDGGSNENSTNNKLTGEAVNTTTEQTTTTNIESSTSKEVTSFISDLEKKSPGTQEFQSFFQKLKDYMKFACPVTSTFEAKDSRSYMSEMMNMATKLSDAMGVLQAKKSGSGLMKTTLQGYQQEVMKTLTTLQSVMSKVVATESEKQFSSEISTEHNYHMEKSSSSSSSSSSSSSPDLDMKHDDETSNMEDVNG

>Aa_G142490

MAKYPLSICLLFMLVSSTVYEAQGTFLLRHYLRKFPRDAKEFEPFAFKGMLSFLDNLESMCPLKGEYRGFFGKLKAFMAFVNSASGSSSEFESQLKTQSEELFKAISALGIKGGSSAETSKLVESLVSMGKTLAEYKRSSSQTMTSEQRIQLVKSMARWALMIGQFVKTVGEKSGDGVNIDLGSLLGGINGDSGGIGGGSSDSGNSDSPSTETGGTGDSGSSSDSSGQGVSSMGSGSPSDSSAGGSPSDSSGGGSPSDSSSGGSPSDSSGGGSPSDSSAGGSPSDSSGGGSPSDSSTGGSPSDSSGGGSSSGSSGQGVSSMGGGNSNTGGESGSGNPADAGSGSPSSETPSSGTPAAGPSGSPSDSSGQGSSSMGGGNSDTTGESGGGSPADARSGSPSSGTPAAGPSGSPSDSSSSGESSMANGSSGSGSSDETENTTAVGGGSPSGEAPTTGPSGGPNGSQSGSSSSTGNAQSAEDATQSAEAAQSTTGANTESASGGTPATGPSGSPSGGSTSTANVQSAEAATESAEAAGKQSTTTANTETASGGTPATGPSGSPSGSSSSTENAQSAEGGSASGETVGTGSATKTNSMSGGSSTENSESASGESQTSASGESQVNASGQTFSDSTGGESPASSPSGSASASGESSGMSASGENSVSGGYSAKQAAGSSTQGNSFGAEGKASIGGSESFKGKMEAANYESSMNYQKSHSKSSSKSSSTHSTEEKKSGDADSDS

>Aa_G142500

MVASSVVYEVQGSFLLKLYLKRKLFPRRCIDFTPYACKGMLMLVSNLEGGCPATREFKSFFSSFKAYLSFLSSVSVSSSSNVDVELQGKTKSLFESCALLNAIKGRRSSSSMSSFQTSMMSMGKILIEQKRSGSRFMSFRQRRQLIVAMVQWTRVVATFVATASEQMGKSIDVSSYGLDVDVNQAVKSTSDSSSSSSEDESSPTPKNSGSSPTPTDSGSSPTPTDSGSSPTPTDSGSSPTPKNSGSSPKPTNSGSSPVDQSDSSSKTKSTGSKSKESSGSETSTKSSGAAYTDTTGTNSASTPSGSPTTTSSGPSTTTGDSSSTTSGSSKQTSGKTSSSSPTPSSSTPTPSTSTPTPSSSTPTPSTSTPTPSSTTPTTSSSGTESETKGSGSASSEKSESQSSSASTSVKQVETETSSEVMSFIKNLEKKYTGNAELKVFFDKLKTSMDASSKISATNGQDLISVMKSAAGRLSEAMMFVRSRFSKSEETKSSMESCQEQVMKSVKDLQELNSEIASAKTVTSTQKTELKQTITKWEQVTTKFVETAASSSSSSSSSQQSSSSQKKTSASSQES

>Aa_G142510

MARISLGICLMLVVASSVIYEAQGSFLLKHYLRKKFPRTCNEFTPYANKGMIMLVTNLESGCPATPEFKTFFSNFKSYMTFIETTSATSKNVDVEITTKADELFKSMTALSGGKGQKSADAGSLKVTMISMAKTLVEQKKNAAIMTMAQKKRLIVSMVKWTRMLATFVKTASEKKGKTINIASYGLDVNVNDKTLISTSSSSSSKSESASSTRTESTASTKTKEAGSTKTKEASGTGNSSTKTKEASGTGNASTKTKEAGSTKTESAKGGAKESSKGGSATTFKDTTGGKLITPSGSPSASPSGSTSSSGKTNTKGSAGAKGSADAKVSGGATATASYETKDSKSGSQTSSSTQSVKQVETETSKDAMTFIMELEKKYSGKAEFKAFFEKLKASMTAFASVSSKTAKDYVSVTKSASAKLSEAMALVGSRNVKSAKMKSNMEDCEQQLMRTLQELIDINSKIVNAKTVTSTQQSEIKQTITKWEKVTTQFVETAASSSSSSSSSSSSSKSEQSSGPHVKKD

>Aa_G142520

MARSSLAVCLLLLVTLSAVYEVQGTFLLRHYMRKFPRRSRMFRPFACRGMLKFCDLLELKCPFKPEYKTFFGRLKSYVGFLNSAASVSKNFDVELKGQAEGLHSAMSALTGKGGSSVDSTKVIDVLMSMGKTLGDNQRSDSILMRLAQRRELARSMAKWAQVITQFVVSAAAQSGTSIDISSLGIDGIDAEISSTNTGSADTTPESPTGSTNTDTTNTDTTNTGTTSTSCTTNKCSSGGGANVKGSLNFQSSGQKTSEQTDSGSS

>maker-Contig139-snap-gene-0.35-mRNA-1

MAKFPLAICLMFILVASSTIYEAQGTFLLEHYMRKFPKMSQDFEPFAYKGMISFVDNLQSKCPANGEYQQFFTKLKSFMSFINTASGSSSEFQSHMKTKSEELFKSISALGVKGGSSADTSKLIDNLMSMGKTFHEFKRSGSTTMTSEQRREMVTSMAKWAQGIGQFVKNVSDKSGDSANIDLSSLIGGANGGSSGFGSSSSPTGDGSTSDSPSSTGDSGSPSYGSGSPSGSPSDSGSPNDSGSSSDT

>snap_masked-Contig221-processed-gene-0.6-mRNA-1

MARVQLLLCFTLLFSAVSLLDIVSAHLKIKPTDMPKIEDPKTVKDVEPYTVKVVMVFVSDLEKECPKTNKFKAFFEKLRAYAKYVCPIKNKEEADYDKDMKAKAGGLLRTISSFAVGKYI

>snap_masked-Contig221-processed-gene-0.7-mRNA-1

MDGDKKEENKETMQLTAEQQKEIKEGILRWKTIMTKITNTMVVSSSSSSSSSSSSSSSSSSEDSKSDKESSKGKSKSSGSSSSSEDSKSDKESSKGKSKSSGSSSSSEDSKSDKESSKGKSKSSGETSYKDTTGSSGSSYMGAPSGSPSPSPSDSTKGSGVMMSPSGSMTIPKGSPMSSPSGSSSNSPTQSGSSTKGSVESSQNSTMTVTEVEAETSKEVMTFIMNLEKKCPQKEEYKSFFEKLKATMTAPPKAAAKGLKGFLSGIKSAGGKLSEAMTFMGARIGSKSAEVKKSMETYQGEVIKTIKELEAIHSQIESQNKDKKDGSLTVTTEQRTEIKKTITKWEQVTTQFVETAIQSETQSSSPTLGKALGNINLPNAA

>maker-Contig599-snap-gene-0.18-mRNA-1

MAKNLLAICLMFLVVSSVVHEVQGTFILKLYLLRKIPMFCAAFVPFAIKGIMMLVKNLESICPATIVFKHFFSKIESYMSYINSVSPTSTNVDSELNGRCESLAKTMSSLTGSKSVDTNELKNTMYSMGKTLVEQKKQGSKRMPYQQRKQLIISMVQWTRVIITFVKTAAETVGTSIDESSYGLDVDVNKSVRNENDKTPSTSTSQTTPSSSTPSNTPSTSTPSNTPSTSTPSDTPSTSTPSDTPSTSTPSDTPSTSTPSDTPSTSTPSTTPSDSIPPNTPSSSTSPTTPSSPTSSGGQTSQKGSSNDTEKESSSNKQKSTSENKQYSSGTSRGSYQDVKGKNSDSPNESPPESPSSTTSGKENETSGKENETSGKENETSGKENETSEKENETSTKSGSSMNQEASSGSSKRSSSNTEVKKVESETSSELMSFVSNLEKKYAEKADLKVFFDKLKTSMAASSKLSSKNQQEFVSGVQSATGKLSQAMTSVRSKIRKSEESRRTEGG

>maker-Contig599-snap-gene-0.20-mRNA-1

MARISLGICLMFIIASSMIYEAQGHFLLKHFLRKRFPQKTNEFTPFANKGMLMFVTDLESSCPPTTEFKTFFTQYKSYMTFIESASTSSKNVDAEMTEKCDGLYKAMSALSSSNDAKSASAGSMKATILSMGKTLFAMKKKAVIMTLKQKKELVLSMVKWTRMLATFVKGASEKKGKTINIALYGLDVDVNDKSIIGDSTESTTTTETENRSTTKTGGNSESPSTPTTNSPSTPTTSSPSTPTTNSPSTPTTNSPTTSGGSSESLSKSQSSSSLSKSQSSSSSTVTMDQVEAETSKNVMTFIMNLEKKCPQKEEYKGFFERLKSTMTFPSRASPRKRKGLFSRIKSAAGKVSDAMAFIRSRIGSKSAAAKKSMENYQGEVVKTMEELDAIYSKIVSQNKGKTEGALTLTAEQQKEIQVTITKWEQVTTQFVETAVQTETQSSVIYEAQGHFLLKNFLRNKFPSKSNEFTPFANKGMLMFATDLESSCPPTTEFKTFFTQYKSYMTFIESASTSSKNVDAEMTAKCDGLFKAMSTLSSSKGAKSADAGSMKATILSMGKTLLAQKKKAVVMTLTQKKELVMSMVKWTRMLATFVKSASEKKGKTINIASYGLDVDVNDKSIFSESSSSTKTATKSEAISGTKNQRYSSGPYKEKIGLNSENKASPSGPTSVGGKTSPKGSESPNGNVNGGSNLATRKSSRSSSQTRQSSRSSYNMAYSSSVKKVESETSKEAMSFISQLEKKYAAKTDLKVFFEKLKTSMQASSSITSKSAKDYVSASKSATSKLSDAMTLVGSRYSKSAKMKSNMETCQDEMMRTLKQLQDINTNIATRKKVTSTQQTELKQTISKFEKVTTQFVETASSSSSSSSSSQQGMKSRMVKNN

>maker-Contig2688-snap-gene-0.4-mRNA-1

MPRFQVLFCFTILVATITFFNVASAHVKIKPVLPQIEDPKTVKDVESYTIKAVMSFLVDLEKECPKTKKFKVFFEKLKAYSKYVCPVSKARGYESDMKAKAGSLFKAISALNSVKNRSKERSVNKSLQREKTEAMNTMKFLQSIGEKIAGRRNKKQEINETMKLTLKQQKEVKHGILKWLHVITRIAKTTEEINSKSSSRSFSTQTKTKSSQRDSQKATIHVLPRGTRRD

>BOERETT00014479

MPRFQFLFCFTILAATIIFFKVASAHVKIKPVLPQIEDPTTVKDVESYTIKVVTNFLVDLEKECPKTEKFKAFYEKLKAYSKYVFPVSKARGYEYDMKAKAGSLFEAMSALSSVKNRFRGGSVKQHKEIKDGILKWIQVITRISKTSKEINSSKYQTTQERNEEKSSTQTPTRRRSHRQKAQITALPRGSRVAKCLKSLKSVVGLLQVETEKVLSFWPKEMNRDVCHGHLNLAHGKIIVCPEDKAFDRFDEDLAMG

>BOERETT00028842

MRKVSLLFFCCIAATSLSLNVVLAHEMVVNPPNTIKDIEPYISNRALGFVLKLETSCPIREQLRSFFQQLKDLLKLESSVTPLIENNEPKTFNFDLKSKSENLLQTMFTLGRGLLSSSVRKEMLEVMKSLTELHVSIGRVIIEKHIKGDESMSLSLEQKNAVENAITEWEGTITRIVKIVVEVKSKGSNEAALEESSTTGHNNVSMDSNMVDTNEENSESTQENADVVKGSNGEDVSMETQEGNKGEDVRDGNIVSETGERKEIKGESVDSNNKKEVERQGEVKSEVEAKNDGNSMTGKLEEAQKDNGVSTMDMNLENKGSDEEAKDDKMVNTTTDYEDHMKEKREETQENNGDSVKDENLENKELMGNGSVETKTNNEMSMEEKREATQGSKEVYMNKETTKGENADFNKDKETKFQGESTGDSTKDTILKNKEDVNSEVNSNESDGSTTKERHQEAQANDGVLTKDKNLENIGADEEKIYDQSVEDTKEKRGETQGNNGESIKNENLENKEDKKELKDDGSVEAKTNNEISMEEKGEQTQGDHDISMNSKTMENKGGNAYSNKEKEVHVGDSTNDNNMESGAKSEVGVKKNDGSSKKEKGAEGQGTNGGSMENKNLENKESQTDLKDDKSVEDKENVRSSIKEKQEEAQRNGGNNKETKDDKSVEAKENKKESKESKWTKTNKNKVKSKEYYVQGNNKESKKVKKGERKESKDAKLVEAKENKKKFMKENREESQGSSSGGLSKVDNKEEKKESKDYQSADTKEKNESSMKEKQQKAQKNGGVYTKIGNKEDSKDLKGDKTVEAKANKESFMKKKREEAQRNEIASTKVKKNKSKDAKKITEEDKGSADNMDIDVQKRRGGSMQYKKEEKKDTIDANSKKKGKDKKEKNESRNSNLKKKQEDKKEKKESGDSKSKKKERKESEDNKLKIKDDSKTEKTNSKDYKLKRKEEDKNNKKEYEGNTSKKKQEDKKENNENESNKLKKKNEDNKEKKESGDSKSKNKEKKEYEDNKSKIKEDNKNEKTDSKDYKSKRKEEDKNEKKKSEDNKKEKKDFEDRKSEKMKEEKQEKKESENHKSKKKEKDKNESDDTRKEKNEEKKKKHEDNDLMKNGEDKKEKKKYEDNKSKIKEEDKKMEKFEDTKSKKKEEFEKGKKEHEDHKLKKKEEEKKEKKEHEEKNPKKKKEDKNEKRKSQDNKSKIKETGEEEEEKEQKKKKKESEKDKSKNTKKQSGSSKQVGFQSKTKNSMKRKEDKKESKDSKSKEAEEQQKNQEQEKKVGVQVNTEDSRTDKKKTKEDVKVSKHGNEKEMPVTTQADSGESKNEMQADAQGKIEESLKDTKGESNKDNEDEVHEENDEDDRKETGNIIKDKKASDKMIEEIKSKRKQEDNKDSSRKKHEEVQEANVNSSINEKKDQNESIYETSKEINESDETSMKQKKIEEGQVNIGNSKMKTKGETKENNGDLVNGNEAKKDSMEGGSKDGNKTEINEGKEDYMKKDSENGKTIETNRDKNGSVEEGPKDAKIVETSGGKNYTEEGSKDDKTTNINEGKADSMKEGSKDGKIVEISKGTYSTEESSRDGNIMETSGGKLDSMEESSKDSNIAETNGGKEDSIKEGSEDGKEIETNGGKEVSTKENSKDGKIEEMNEGQEDSTKKGSEDGKTNEIHGGKVVTMEEGSKDDKTIEINGGKEDSMEDNSKDGKTADLNGDTYSMEKSYEDSKTVEINGVEEDSKNGNINEIRKGKEDSMKDSVMENQGNDNTSASNTSMEPKGDNLDINEDSMKNKTMETQEGSNGDSTNGETEETKESNEFMNNQNVQDGGSNANIMNNETTGDGGNSTTKTKITTDIESNSSKEVTNFISNLEKKSLGTQEFQSFFQKLRDYMNYVFPVSSTFEAKDSRSYMSEMINMATKLSDAMAVLQAKKTGSEQMKTTLEGYQQEVMKTLNILQSVMGKAISEQQSNNGGSFTLTLSQQQAIKEIVLKWEQVMSQFVKIATESEKQFSVDFSTENGYHMKKSSNSSSSSSSSSNLDLKLDGETLKGLDMNG

>BOERETT00028843

MARFPLGICLMLIIVASSTIYEAQGGFLLRHYMRKFPEMSQDVEPFAYKGMTSFVDNLQSMSPAKGEYKDFFSKLKSFMAFINTAKGSSSEFQSQMKSQSEGLSNAISALGVKGVSSADTSKLIESLMSMGKTFVEFKRSGATTMTSEQRRELVTAMAKWAQVIGQFVKKVSGDGANIDLSSLIGGGSSGFGSTGGSPSSTGYSGSPSYGSYSDSTGDSGSPASSPSYTGYGSMGDAGSGRPAGGPSGSTTDSSSGGLGSMGAGGGAAGETESATDADSGSASGGGDAGGSTETGGDSVSGGGIFGGSTETGGESASGGGAAGGSTETSGDSASGGGAAGGSTETGGESASGGGVSGGSTETGGESASSGGAAGGSTKAGGESTNGETAAGGSTEANGESTSGETAAGGSTEAGGDSTSGGTTASGSTKAGSENTSGETATGGESTSGGGAVGGSTEAGGESTSGGTAVSGSSEAGGESTSGKIVVGGSTEADGESTSGETTAGGSTKASGESTSGGTAAGGSTEPGSESTSGETATGGESTSGGGAAGGSTEAGSESTNGGIAAGGSTEAGGESTSGKTVAGGSTEAGGESTSGGTAAGGSTEVGGGTSTETSSMGGGSAASSDAGGGASSMGGESSGSQTSSATGGASQSSENGESSSDDSAAAGGTTQRTGSGGSSNGGSNAAGGATQSTENGESSSDESAVAGGATESTRSSESSSGGSAAAGGASETASESSKNQASSMAGGGTYTDSTGGSPAGSPSAGGPSGSTTESSMEGVSGGSANEASSTSQQSASAARGGAAEESSGEGGASGGQSMEGKADTVSYKTAKYEKRHSKSSGKISFSHSSEEKSSGSANADS

>BOERETT00028844

MAKNLLAICLVFMVASSVVYEVQGTFLLKLYLMRRFPRRCMAFAPFAAKGMLMLVRNLEGGCPATREFKEFFTRFRSYVSFISSASSSSRNVESEMNGRCELLAKAMSALSGSNSEQSSELKNTMLSMGKTLVDQKRQGSKIMTMKQRKELVISMVKWTRVVVTTIKSVAEKRGKSIDESSYGLDVDVNKSIGSRSESGGSSSSDSDSSSNTRSQGWSSKSGSASNTEAGSTETGSSTKTGSFGRSGSDSSNTKESRAESSGNTYKDTTGSNSDSPSGSPTTSRSSSTSADDKTSEKGSASSSGSASMRREYKSGSATSGKVSSKTKESSSGSSGNTYKDTTGTTSGSPRRSPTGSPSGSTSTGGETSVQESASARGSASESDSGSESDSGSASSEKKESSRKSSRSSSSSTSVKEVESQTSSEVKSFISNLEKKYTGNAELKVFFDNLKTSMTASSKLSSSNKQDFLSGMKSAATKLSEAMMFVRSRFSKSEETKNSMENSQEQVMKTLKELQDINSQIVSGKKVSSTQQTELKQTITKWEQVTTQFVETAASSSSSSSSSSSSSQQQGSARMAQNN

>BOERETT00028845

MQRGDEMQLQHYVICSDMALDNVLFRTSADLFPILERIKAMAPDSFPAADLILENKLFLCLGGAIIVKKSMETYQEQVMKTLQELQTIHSQIVSQNKGKKGGSLTCTPAQQTQIKLTITKWEQVTTQFVETAIQSETQSSSTTSSSLKKMMPN

>BOERETT00028846

MARISLGICLMLVVASSVIYEAQGTFLLNHYLKKIPKKCNEFTPFANKGMLTFVTNLEGSTPATTEFKNFFTQFKSYMSFIETASASSKNVDAEMTAKCDELFKAMSAMSSGKGVKSVDAGGLKATMLSMGKTLVEQKKNTNIMTSTQKKELVTDMMKWTKTVGTFVKSASEKKGKSIDIASYGLDVDVNDSSIVGGTASSSESSSSSTKTGSVSSKTKEMSRGSSSSGSVSSKTKEMRGGSSASGSASSKTKESSGGSSGGAYKDTTGKPSGGPSGSPKANPSGSAPAGGKGTLKGSASAAGSVSGGKTFSTNTATKSSSERQSKSSSQSSSSTSVKQVESETSKEVMSFIMELEKKYSSKADLKIFFEKLKASMQASSSIASKSSKDYVSSTAATTGKLNEAMAVVGARNVKSAKMKSNMDTSKDELIKSLKQLQDINSKIVSGKKVSSTQESELKQTINKWEKVTSQFVETAASSSSSSSSSSSASNQQGSARMVKNN

>BOERETT00028847

MARISLALGLLLLVALSSVYEVQGTFLLRHYLRKIPRRSRDFQPFACKGMLNFVDVLQNKSPMTPQYKSFFGNLKSYMNFINSASGSKNYNSELKGKAQGLHSAITSISGKSGSSADSNKVMETLMSMGQTLGQQKQSGSTVMSFGQRKEMITSMAKWAQTIGQFVASSAAKNGKKIDVSSLGVDGIDASAAAESGDSTATGGTSSTVSGGSSSTSSGSGTATGGSNFYGSDTATGGSTATSSDTASGGSTATGSGTATGGSTVTGSGTATGGSSETGSVNGSYGSGGGSTQSSTNGGSQTSSCTTSKCSNGGGSSFKGSMNFKGQASSQSQHKSSQSQQGSSQQSASFDGSS

>BOERETT00028871

MARVQLFICFTILFASVTLLDVVSAQLKLKPSLPQIEDPKTVKDVEAYTVKVVMVFVSDLEKECPKTSKFKAFFEKLRAYAKYVLPIKRKDQVDYDRDMKAKAGGLFQTISSFAIGKIKGKIQEEKMEAVNNFRSMRSIAAKILGGRQKEESEETMKLTAEQQKEIKEGILRWETIITRITNTMVMSTTNSSSSETSSSVTSTTEKGGSSGNSKSSGSGSSTFNSKGSFEDTTGSSSYLGSPSGSPSSSPSGSSSSAASGSGTSSTGGSSVSGNVASSQSSSVTVQQVEAETSKDISTFIMNLEKNCPPKEEFKAFFVQLKGTMIAPPKQRKGLFSRIKSATGKLSGAMAFIRSRIGNKSAEVKKSMEDYQEQVMKTMQELQTIHSQIVSQNKGKKGVSLTCTPAQQTQIKQTITKWEQVTTQFVEIAIQSETQSSSTTSSSLKKMMPN

>BOERETT00057824

MARVQLLLCFTILFASVSLLDIVSAHLKFKPSLPQIEDPKTVKDVEPYTVKVVMVFVSDLEKECPKTSKFKAFFEKLRGYAKYVCPIKRKNQVDYDRDMKAKVGSLFQAISSFGVGKIKKEIQEQKMEVMNTFKFMRSVAGKIIGSRKKEESEESMKLTAQQQKEIKEGILKWETVITQITNTIVISTTNSSSTENSTSGKGSSSGSSKSSGSFNSGSTFTDTTGLGSNLGSPSGSPTISPSSSNSFAPSGRRSLLSTSGSPTSSPSGSSSFGAGGRGTVFTDTSGISISPTPTQSGSVGASQKNTKTMTQVEAETSKQVMVFLINLEKKCPQKEEFKVFFEKLKSTMAAPAKSSSKTKKGFFSGIKSAAGKLSDAMTFMRSRIGSKSAKVKQSMETYQEEVMKTISELESIHSKIVSQNKGKKDGILTCTAAQQKEIKQTITKWEQVTTQFVETAIQSETHSSSTPSSTLGKIIGKIKPT

>Bostr.0556s0528

MRKVSLLFFCCIAATSLSLNVVLAHEMVVNPPNTIKDIEPYISNRALGFVLKLENICPIREQLRSFFQQLKDLLKLESSVTPLIENNEPKTFNFDLKSKSENLLQTMFTLGRGLLSSSVRKEMLEVMKSLTELHVSIGRVIIEKHIKGDESMSLSLEQKNAVENSITEWEGTITRIVKIVVEVKSKGSNEAALEESSTTGHNNVSMDSNMVDTSGENSESTQENADVVKGSNGEDVSMETQEGNKGEDVREGNIVSETGERKEIKGESVDSNNKKEVEGQGEVKSEVEAKNDGNSMTGKLEEAQKDNGVSTMDMNLENKGSDEEAKNDKMVNATTDDEDHMKEKREETQENNGDSVKDENLENKELMGNGSVETKTNNEMSMEEKREATQGSNEVYMNKETTKGENADFNKDKETKFQGESTGDSTKDTTLKNKEDVNSEVNSNESDGSTTKERHQEAQANDGVLTKDKNLENIGADEEKNYDQSVEDTKEKRGETQGNNGESIKNENLENKEDKKELKEDGSVEAKTNNEISMEEKGEQTQGGHDISMNSKTMENKGGNAYSNKEKEVHVGDSTNDNNMESGAKSEVGVKKNDGSSKKEKGEEGQGTNGGSMENKNLENKESQTDLKDDKSFEDKKNVRSSIKEKQEEAQRNGGNKKETKDDKSVEAKENKKESKESKWTKTNKNKVKSKEYYVQGNNKESTKVKKGERKESKDAKLLEAKENKKKFMKENREESQGSSSGGLSKVDNKEENKESKDYQSADTKEKNESSMKEKQQKAQKNGGVYTKVGNIEDSKDLKGDKTVEAKANKESFMKKKREETQRNEIASTKVKKNNSKDAKKSTEEDKGSADNMDIYVQKRRGGSMQYKKKEKKDTIGANSKKKGKDKKEKKESRNSNLKKKQEDKKEKKESGDSKSKKKERKESEANKLKIKDDSKTEKTNSKDYKLKRKEEEKNNKKEYEGNKSKKKQEDKKEKNENESNKLKKKNEDNKEKKESGDSKSKNKEKKEYEDNKSKIKEDNKNEKTDSKDYKSKRKEEDKNEKKKSEDNKKEKKDFED

>Bostr.0556s0529

MKIKSKKKEEEKKEKKEHEEKKPKKKKEDKNEKRKSQDNKSKIKETEEEEEKEQKKKKKESEKDKSKNTKKQSGISKQVGFQSKTENSMKRKEDKKESKDSKSKEAEKQQKNQEQEKKVGVQVNTEDSRTDKKKTKEDVKVSKHGNEKEMPLTTQADSGESKNEMQADAQGKIEESLKDKKGESNKDNEDEVHEENDEDDRKETGNIIKDKKASDKMIEEIKSKRKQEDNKDSSRKKHEEVQEANVNSSINEKKDQNESIDDTSKDGKIVEISRGTNSTEESSRDGNTMETSGGKLDSMEESSKDSNIAETNGGKEYSIKEGSIEDGKEIETNGGKEVSTEENSKDGKIEEMNEGQEDSTKKGSEDGKTNEIHGGKVVTKEEDSKDDKTIEINGGKEDSMEDNSKDGKTADLNGDTYSTEKSSEDSKTVEINGVEEDSKNGNINEIRKGKEDSMKDSVMENQGNDNTSASNTSMEPKGDNLDINEDSMKNKTMETQEGSNGDSTNGETEETKESNEFMNNQNVQDGGSNANIMDNETTGDGGNSTTKTKITTDIESNTSKEVTNFISNLEKKSLGTQEFQSFFQKLRDYMNYVFPVSSTFEAKDSRSYMSEMINMATKLSDAMAVLQAKKTGSGQMKTTLEGYQQEVMKTLNILQSVMGKAISEQQSNNGGSFTLTLSQQQAIKEIVLKWEQVMSQFVKIATESEKQFSVEFSTENGYHMKKSSNSSSSSSSSSSSNLDLKLDGETLKGLDMNG

>Bostr.0556s0530

MARFPLGICLMLIVVASSTIYEAQGGFLLRHYMRKFPKMSQEVKPFAYKGMTSFVDNLQSMSPAKGEYKDFFSKLKSFMAFINTAKGSSSEFQSQMKSQSEGLSNAISALGVKGVSSADTSKLIESLMSMGKTFVEFKRSGATTMTSEQRRELVTAMAKWAQVIGQFVKNVSGDGANIDLSSLIGGGSSGFGSTGGSPSSTGDSGSPSYSGSYSDSTGGSGSPASSPSYTGYGSMGDAGSGSPAGGPSGSTTDSSSGGESSMGAGGGAAGETESATDADSGSASGGSAAGGSTETGGGSASSGGVSGGSTDTGGGSASSGGFSGGSTDTGGGSASSGGVSGGSTDTGGESASGGGVSGGSIEIGGESASGGGAAGGSTETGGESASGGGVSGVSTETGGESTSGGGVSGGSTNTGGESASGGGVSGGSTETSGESASGGGVSGGSTDTGGESTGGGDVSGGSTETGGESASGGGTAGGSTEASGESTSGGTAGGGSTEAGGESTSGGGAAGGSTEADGESASGGTAAGGSTEAGGGTATETSSMGGGSAAGDSSDAGGGASSMGGESSGSQTSSATGGASQSTENGESSSDESAAAGGATQSTGSGGSTAAGGATQSTGSGGSSSGGSAAAGGATESTGSSESPSGGSAAAGGASETASESSKNQASSMAGGGTYTDSTGGSPAGSPSAGGPSGSTTESSMEGVAGGSADEASSTSQQSASAAGGGAAEESSGEGGASGGQSMEGKADTVSYKTAKYEKRHSKSSGKSSFSHSSEEKSSGSANADS

>Bostr.0556s0531

MAKNLLAICLVFMVASSVVYEVQGTFLLKLYLMRRFPRRCMAFAPFAAKGMLMLVRNLEGGCPATREFKEFFTRFRSYVSFISSASSSSRNVESEMNGRCELLAKAMSALSGSKSEQSSELKNTMLSMGKTLVDQKRQGSKIMTMKQRKELVISMVKWTRVVVTTIKSVAEKRGKSIDESSYGLDVDVNKSIGSRSESGGSSSSDSDSSSNTRSQGWSSKSGSASNTEAGSTETGSSTKTGSFGRSGSDSSKTKESRDESSGNTYKDTTGSTSDSPSGSPTTSRSSSTSADDKTSEKGSASSNGSASMRREYKSGSATSGSVSSKTKESSSGSSGNTYKDTTGTTSGSPRRSPTGSPSGSTSTGGETSVQESASARGSASESGSESDSGSESDSGSASSQKKESSSKSSRSSSSSTSVKEVESQISSEVKSFILNLEKKYTGNAELKVFFDKLKTSMTASSKLSSSNKQDFVSGMKSAATKLSEAMMFVRSRFSKSEETKNSMENSQEQVMKTLKELQDINSQIVSGKKVSSTQQTELKQTITKWEQVTTQFVETAASSSSSSSSSSSSSQQQGSARMAQNN

>Bostr.0556s0532

MARISLGICLMLVVASSVIYEAQGTFLLNHYLKKIPKKCNEFTPFANKGMLTFVTNLEDSTPATTEFKNFFTQFKSYMSFIETASASSKNVDAEMTAKCDELFKAMSAMSSGKGVKSVDAGGLKATMLSMGKTLVEQKKNTNIMTSTQKKELVTDMVKWTKTVGTFVKSASEKKGKSIDIASYGLDVDVNDSSIVGGTASSSEGSSSSTKTGSVSSKTKEMSRGSSSSGSVSSKTKEMRGGSSASGSASSKTKESSGGSSGGAYKDTTGKTSGGPSGSPKANPSGSAPAGGKATLKGSASATGGASAAGSVSGAKTFSTNTATKSSSERQRKSSSQSSSSTSVKQVESETSKEVMSFIMELEKKYSSKADLKIFFEELKASMQASSSIASKSSKDYVSSTAATTGKLNEAMAVVGARNVKSAKMKSNMDTSKDELIKSLKQLQDINSKIVSGKKVSSTQESELKQTINKWEKVTSQFVETAASSSSSSSSSSSASNQQGSARMVKNN

>Bostr.0556s0533

MARISLALGLLLLIALSSVYEVQGTFLLRHYLRKIPRRSRDFQPFACKGMLNFVDVLQNKSPMTPQYKSFFGNLKSYMNFINSASGSKNYNSELKGKAQGLHSAITSISGKSGSSADSNKVMETLMSMGQTLGQQKQSGSTVMSFGQRKEMITSMAKWAQTIGQFVASSAAKNGKIIDVSSLGVDGIDASAAAGSGDSTATGGTSSTVSGGSSSTSSGSGTATGSSNSYGSGTATGGSTATSSDTASGGSTATGSGTATGGSTVTGSGTATGGSSETGSVSGSYGSGGGSTQSSTNGGSQTSSCTTSKCSNGGGSSFKGSMNFKGQASSQSQHKSSQSQQGSSQQSASFDGSS

>Bostr.10058s0007

MPRFQFLFCFTILAATITFFKVASAHVKIKPVLPQIEDPTTVKDVESYTIKVVTNFLVDLEKECPKTEKFKAFYEKLKAYSKYVFPVSKARGYEYDMKAKAGSLFEAMSALSSVKNRFRGGSVIQHKEIKDGILKWIQVITRISKTSKEINSSKYQTVQESNEEKSSTQTQTKRRSHRQKAQITALPRGSRVAKCLKSLKSVVGLLQVETEKVLSFWPKEMNRDVCHGRLNLAHGKIIVCPEDKAFDCFDEDLAMG

>Bostr.1693s0001

MARVQLFLCFTILFASVTLLDVVSAQLKLKPSLPQIEDPKTVKDVEAYTVKVVMVFVSDLEKECPKTSKFKAFFEKLRAYAKYVLPIKRKDQVDYDRDMKAKAGGLFQTISSFAIGKIKGKIQEEKMEAVNNFRSMRSIAAKILGGRKKEENEANSRTTKRNQRRDLTMGNDYYSNNKHNGNEHNKYFFFFYETSTTETSSGNSKSSGSGSSTFNSKGSFKDTTGSNSYSGSPSGSPSSSPSGSSSSAASGSGTSSMNTTSGSSVSGNVASSQSSSVTVQQGTMIAPPRQRKRLFSRIKSAAGKLSGAMAFIRSRIGNKSAEVKKSMENYQEQVMKTLQELQTIHSQIVSQNKGKKG

>Bostr.2392s0027

MARVQLLLCFTILFASVSLLDIVSAHLKFKPSLPQIEDPKTVKDVEPYTVKVVMVFVSDLEKECPKTSKFKAFFEKLRGYAKYVCPIKRKNQVDYDRDMKAKAGSLFQAISSFGVGKIKKEIQEQKMEVMNTFKFMRSVAGKIIGSRKKEESEESMKLTAQQQKEIKEGILKWETVITQITNTIVISTTNSSSTENSTSGKGSSSGSSKSSGSFNSGSTFTDTTGLGSNLGSPSGSPTISPSSSNSFAPSGRRSLLITSGSPTSSPSGSSSFGAGGRGTVFTDTSGISISPTPTQSGSVGASQKNTKTMTQVEAETSKEVMVFIINLEKKCPQKEEFKVFFEKLKSTMAAPAKSSSKTKKGFFSGIKSAAGKLSDAITFMRSRIGSKSAKVKQSMETYQKEVMKTISELESIHSKIVSQNKGKKDGTLTCTAAQQTEIKQTITKWEQVTTQFVETAIQSETQSSSTPSSTLGKIIGKIKPT

>BcaC01g00190

MARVHLLLCFTLLFASVTLLDPSLPQIKDPKTVGEVEGYTVEVVTIFVGNLEKECPKTICPLKIFGKKDGADMKAKEAGILKTIASFAIGREKQEAIETFKFMKSLAGRILGGRKKEEKETTTLTVEQLKEIKDGILKWQTVIVKVANTMVVSTTTNNEGSAGSNPGAGTPSTDTNNQSQGAPSTDTNNQSQGTTEGSSSPNSGSATGSPSNKPSAGSNPGAGTPSTDTNNQSQGTTNTASSRSATTSQTTEVTVTEVETQTSEQVMTFLMNLEKKCPPKEEYKQFFEKLKSTMAGSAKVSSPKKKGGLFAMIKGAVGKIGDAMQFIRSRIGNKSAEVKKSMETYQAEVIKNMEELNAIYAKIVGKGGAMTCTPEQQAEIKTTITKWEQVTTQFVKVAIKSETSTATSTSTSSSTGTAQAN

>BcaC03g17528

MKKVYLLCLCCITILATILSFNVVSAQGIAVNAPNTIKDIESYISNRAIGFVLKLEDECPIREKLRSFFEKLKDLLKLESSVTPMIEENEPKTFMSHMKSKADNLLQTMLIIGRGLLSSSVRKEMFKVVKSLTELHAAIGKVIMEKHIKGDGSMSLSLEQKNAVENAVSQWEVTITRIVKIVVEVKSKSSSAALGEESSTTDQNNTSVNETMLESNKGNFESMQENADDVSESNEDSKNDVAMKAKEGNRVEDVKGGGTEVSMNGETMHTKNGNVDSNKEKEVEVQGERSIGDSQINKNLDTKEDIKSEVDARNDGSFMNEKSEAQGNDEVSTKNTNLENKGKVQEGSIGDSHINKNLDTKENVKSEVDARNDGSSMTELEDTKGNGQLFTNDTTLENKESEARTYDEDHTKENGEDTQENNGELVKDANLENNKDNKEVKDDGFVETKTNHENSTEEKREHTQRGTEVSMNGDVMPTKGGHVNSNKESVLKFQGEIIGDSTKDINLENKDVKSEVETNKNDESSMKEKQGYSHGNDRVSTKDQNLESVGAHEETMNHKTVDAMKNNGDHLKKKREENQQNNKESTKDENSENKEDKKEFKDNGLVETKKNHETSMEENRKHTQGGTEVSMNGKMMHTKNGNVDSNKENEVEVQGGSIGDSHINKNLDTKEDIKSEVDARNDGSSITKNLEEAKGNGGVLTNDNNLENNKDNKEVKDDGFVETKKNHETSMEEKKEKDHVDNDEFTYGGNKNESKDDKLDEIKKNKKEHMDKKWREAKGNEKNIMRGHPKEVSIDNKGYKKEIKDGKPLEVKENKEKKEETQRSSSGQYTNVDNKEGKDDKSVEVKENKEKRKKKRKRSRRALRTHIQKKEEEHLSKKKEEKQRIELEKNKLKKEEEKKKQKEHEEHISKKKEEEKRKEHEKNKSEKNEKEKKKQKEHGEYISRKKEKDKRIEHERNKLKNEEEKKKHKEHEEHKSKKKEEEKKNEHERNKSRKNEEEKKKQKEQEEHLSKKKDEEKRKEHERNKLRKNEEEKKKQKEHEEHISQKKEEDKRKEHEEHTSKKKEEEKRKEHEENKAKKIEEEKRKEHEKNKSKKNEDKKSEHEQYKSKKTHKENESNENEEEKMKKREYEETKSKKNEEQKKREHEKNKSNENEEEKKKEHEENKFKKEEEEKKMKRDYDNNKSKKNKEDGKEKTKENVKITAEINGGKEDSIEEGSKDSTAEINGGKEDSIEEGSKDSTAEINGGKEDSIEEGSKDSTAEINGGKEDSIEEGSKDSKTVEVNEGQDKSMEKGNGSQDVTMEEASKDGNTTELNGSKDNSMEEGSKDGKTLENNNNSTENSVEEGSKDSTAEINGGKEDSIEESSKDGDIDEINSDKEESMKEGSENDKTADIDGIKEVPIEEGSKDSTAEINGGKEDSIEEGSKDSTAEINGGKEDSIEEGSKDGKTLENNNNSTENSVEEGSKDSTAEINGGKEDSIEEGSKDGKTLENNNNSTENSVEEGSKDSKTVEVNEGQDKSMEKGNGSQDVTMEEASKDGNTTELNGSKDNSMEEGSKDSKTVEVNEGQDKSMEKGNGSQDVTMEEASKDGNTTELNGSKDNSMEEGSKDSKTVEVNEGQDKSMEKGNGSQDVTMEEASKDGNTTELNGSKDNSMEEGSKDSKTVEVNEGQDKSMEKGNGSQDVTMEEASKDGNTTELNGSKDNSMEEGSKDSTAEINGGKEDSIEESSKDGDIDEINSDKEESMKEEETAESNDSMNNQNVQHVEGDNIDINEDSTKNKTMEAQGGGAGDSTNGETEETTESNDTMNNQNVQHGENNANSINNQTSESSSTDKKVTTDIESSTSKEVTSFISNLEHKSPGTQEFQSFFQKLKDYMKYAWPVSSTFEATDSRSYMSEMTNMATKVSDAMAVLQAKKLGSGLMKTTLQGYQQEVMKTLTILQSVLSKAVSGQQSQNPGSLTLTLSQQQAIKEITLKWEQVMSQFVRVATESEKQFSMETSTGNGFHMKKSFSSSSSSSSSSSPGLDFNLNSESPEMVDVNS

>BcaC03g17529

MQVAINQLCMSATRPHGHELFDEPDHQRMNLELDRNGTRAGCFLSSRERKMTRFPLAICLMCIIVASSTVYEAQGGFLLRHYLRKLPRLANEFEPFAFKIMIRFIDNLESLCSSKVEYKEFFSKLKAFLIFINSTAGKSSSSEFESQLKAHSEGLFKAITALGVKASADTSKLIESLMSMGKVMAEYKRSGSLTMTSEQRRVLITSMMKWAQVIGQFVKTVREKTGDGDIDLPSLGIGGSGESAGGETSGAGGSSGNTSNMSTEESSSMGGGNSSEDSKIATGGSASAESTSGGASGGSTKTTAAGESSMNNGDSYSDSTGGNTMGSPTGSPSGSGGSSFTGSETGSSSYQAGGASAGGPSGSTTDSSAAGASSKNSGYSAKGSSTTSAQGSTQGGSGLAEGASEGKSFKGKTGSTRYEGSSNYQKTHSKSSDKSSFSHSSEEKSSGNV

>BcaC03g17532

MANKNVLGLYLMFLVVSSVVYEAQGTFLLNMYLKRKFLRNTRNFTPFACKGMLLLMSRLKRGCPATEGFKTFFSLFTSYVKFIKTAPTTSNTDTQLTTKVDALTNAISVLTGTKSGSNSNFRETMLSMGKTLIEQKRSTSQRMTHQQKKVLVAAMVQWTKTVATLVKTAVETAGKSIDVSNLGLDVDVNSIVGSGSDESPESGSPGSDSPTTTSSGSNTDSGSIIYEDTTGGGSSSPSGSPADSPSSTMTTQSAGSTPGSQSGTTTESPNDTTTESPSGTTTESPSGCDSPSGSPTDSPSDTTTESPSGTTTESPSDTTTGIPSGSPTDSPSGATTESPSDTTTDSPSDTTTGSPSGSPTNSPSGATTDSPSGATTDSQSDTTTESPTDTTTGSPSGSPTDSPSGATTDSPSGATTESQSDTNTESPTDTTTGSPSGSPTDSPSGATTESPSGATTDSPSGVTTGSPSGSPTDSPSGATTDSPSGATTDSLSGTTTESPNGSPTGSPSGTTTDSPSGSPTDSPSGSPTDSPSGSPMDSESSQSSTSSSSTTSAKEVEIQTSKEARSFIHALEKKYAGTVQLDTFFEKLKTSMSASTKISNTDEKRFVSKMSSAVSAVSEAATTVSSKLAKSPEAKSRMESSKEKLMKTYKELEDVNSKIVSENKGKTVSSTQKSELKQTLSKWEQVTTQFVENLVSSSSSSSSSSSSSQSQQSQQSQKSHQSQQSQQGSTMKTETN

>BcaC03g17568

MSFIMNLEKKCPPKEEYKSFEKLKDTMVASATGYSRKEERFLLWKVVKALQELETIHSKIISATQGKKDESVTVTAEQRTEIKQTITKLNGNKSQPSSWRLRLLQILQ

>BcaC04g23863

MANKNVLALCLMFILVSSVIYEAQGTFLLKMYLRRKFFKKAMQFTPFACKGMTFLLNRLKGGCPATKGFKTFFSLFISYVNFIKTATVSKTTDSQLTTKADGLANAVSVLTGARKDVSNNFKETILSMGKTLIEQKKAGTQPVTYKQRKVLIVALVQWTKTVVTVVKTAVETAGKTIDTSNLGLDVDVNDVIGGEKDDDSPTPTTPTTPTTPATPTTPSTPTTPSTPTTPTTPTTPTTPTPSTPTTPTPTTPTTPTPSTPTTPTPSTPSTPTPSTPTTPTTPTPSTPRTSTTPTPNTPTTPTPSTPTTPTTPTPTTRTPTTPSTPTTPTGTTTNNAATNTNSQTSQSTTSTASAKQVETQTSQEVMSFISDLEKKYASKTELNTFFETLKSTMTATSKIASTDEKTFVSGVKAAAGTLSEAAETVTEKLGTSAESKQKIESSQQQLMTTFKELEDVKTKIVSESKGKTVSSTQQTELKQTLTKWEQVTTQFVETAASSSSSSSSTQSQQIQQSHQSQQSQQSQKTQQTQQTQQIQQGSILRAQTQT

>BcaC06g30894

MARISLGLCLMLVVASSVIYEAQGHFLLKNYLKTQFPSKGNEFTPYVNRGLTELLTDLERFCPPTPEFKGFFTEFKSYMSFIETSSSSSSKNIDMEMTKKADGLFRAMSAISGGAGQKSAEAGSFKSTMISMGKTLVEQKKSTTTITSTEKKTLVTSMVQWTKTIATVVKTASEKKGKKIDINSFGLDVDVKSIVSVSGSSHSSSSSTKTSSESSSKSSSYNARAETGSSAKAKETTEKGEKKDNAEKKPEKKEEKKPEKKEEKDSSSAKTKETSSTKAGASGSTKTGSSVKDTTGGSAGSPRGSPTAAKDKTSGSPTSDKGRTSEKESGNAGSVKQQSNNAASRGSASSMTQQQSKKSESASSTTSVTEIEKETSQETSSFISGLEKRFSQKAELRPFFEKLKASMTASSRVSSTKSAQEFTSTAKSTTGKLSDAMTFVGSRFSKSAEMKSNIETTQEKLMKSLKEFQDLNSRIVGEQKVSTTKETQIKQTMSKIEQVTTQFVETAASSSSQQSQQSQKQRTATSQQQTAASSQQQTAASSQQQNEQQNGMGRLKTN

>BcaC06g30895

MFMITTSTIYEVQGHFLLDHYLKKIPTISSEFEPFASKGILSFIDHLEGLSPPKEEYKEFFTKLKEFMAFINSASGSSSEFHSQLKTKSEELFKAITKMGGKAGSSAHTSKLIESLMSMGKTFAEYKRSGSQTLTSEQRTELVTSMSKWAQVIGQFVKTATEKSGDGNIDLKSLGCGGGTSGSISSSSTETGSSASGSSSMENALSTGGSGSPSEATGSPSAETGSPASGDGPGDSGGPSDSGSPSNETPAEGGSSSPSGSPSDSSSGPGSTGVESPAEAGSAAGAGSGTGDGETPGAAGPSGSPTEPGESSKDESSKDESSEDESSKDESSKDESSKDESSKDEKATESGESSKDASGANSNEESSTETQSETGAESGSTDGGASAGGPSGSPTESSGPGDVGAGGPSGEPSGEAGAGGETSGEAGAGGETSGEAGAGGETSGDAGAGGPSGETSGAGGETSGAGGPSGSTSDMSAGGASTSGGASGTSTQTSAEAESTMNNGGSFADTTGGSSGMEGPASSPSGSASGGSSETSSFSEMSSHQAGGSSAGAPSGSTNDGSAEGETSTKGKMSYERSASYRKSHSQTSGHSSFKHSSEEKNTGSS

>BcaC06g33251

MAKTSLTIYLSLLVALSTVYETQGTFSLPLYLKNFPKVGHDFESFAYKGMMDFMGDLEGKCPQTTEFKDFFLKLKDYMACYSSTAPGSKDLQVELSIKSETLFRAMSDFSGTKGGTSEDSWTLVDGLLSMGKSLVEMKKSGSKEITFEQRKEIIQSMVKWTRGIGLFVKKVSESKGQSIDLSSFGIDYDNNVSSPSERALYETQGTFSLPHYVKDFPKMSKDFEPFAYKGMSGFLGALESKCPATAEFKDLFVKVADYMACFKSGIEVEMQEKSVKLFRAISVLDGTNGGTSVDSWRMVDGMLSMGILVTEMKKNVSQEITFEQRKELIGGMVKWARAIGLLVKTASEKKGKSIDLASFGVDYSPHVPSPFKGASGEL

>BcaC06g33642

MARISLALCLMLVVTSSVIYEARGHFLLKDYLTTKFPSKSSEFTPYVNTGLTEFLTDLERFCPPTPEFKSFFTEFKSFFSSIETSSSTSQNIDMEKKGDGLFKAVSAITGGAGQKSAEAGSFKSTMISMAKTLVEQKKSTTAITSTEKKTLVTSMVQWTKTIATTVKTACEKKGKKIDINSFGLNVDVNSVMTVSESRQSSSSSSKSSSESSSKSSSYAARAETAASAKAKETTGARAETGASAKAKETTGASAEKGEKKAEKKAEKNEQKAATSSAKTKEASSTKAGASSSTKTGTIFRDTTGGYAGSPRGSPTAAKDKTAGSPRGSPTAAKEKTSVKGTGKAAASVNQQSNAGSSRGSAAASTNQQQSNAASSRGSAASTNQESASSKSSSTTSVTEIEKETSQETTSFISGLEKRFAQKAELKPFFEKLKASMTASSRVSSTKSEQEYTNTAKSTTGKLSDAMSFVGSRFSKSAEMKSNIQTTQQQLIKNLQQFQNLNSQIVGEQKVSSTKETEIKKTMSKIEQVTTQFVETAASSSSSSSKQETAASQQSQKKTMASSKKETAASSQQQTQQQTQQENGMGRLKTN

>BcaB04g19484

MANKNVLALCLLFLVTSSVIYEAQGTFLLKWYLRRKMFKKAMEFTPFACKGMLFLLNRLKEGCPATKGFKTFFSLFTSYVNFIKTAKVSKTTDTELTTKADGLAKATSVLTGAKTVSTDFRETLLSMGKTLIEQKKSGAEPVSRKQRKVLVVALVQWTKTVVTIVQTAVQTAGKTIDTSNLGLDVDVNDVVGDDSDDDDDDSPKKGTPSGSDSASPSSGTPTPTTPTTPTTPTTPSTPTSSTPTPTTPTTPTTPSTPTSSTPTPTTPTTPTTPSTPTGSTPTPTTPSTPTTSTPTPTTPTTPSTPTGSTPTPTTPTTPSTPTRSTPTPSTPTSSTPTPTTPSTPTTPTTSTGGETTSTGSTTNTGSTNTKGAGSTKTTQEATETDTGATKGSPSGTPTDSPSGETPTNTNSQTSESTSSTTSAKQVETQTSQEVMSFISGLEKKYAGKAELNTFFEKLKSTMSATSKIATTDEKTFVSGVKSAAGKLSEAAETVTEKVGKSAESKQKIESSQEQLMKTFKELEDVKTKIVSESKGKTVSSTQQTELKQTLTKWEQVTTQFVETAASSSSASSSSQSSLSQKSQQSQQSHQSHQSQHTQQTQQTQQGSTMKAQTQTN

>BcaB06g27386

MSRIQNLLCFTILLATVTFFNVASAHVKIKFALPQTGGPISVGDVEPYTVKVVSTFVADLEKECAKTEKFRHFFENVNAFSKCVCSVSKVEDHESHMKAKAGSLFQAISALGSDENGSKGGKVNKLQKEKTEAMETVKILQSIGEKITGGKNNKTEINGTLKVTTKQQKEIKDGILKWLDVITRIAKKADEISMESSSKSVGPRILVETKSSTKSNENSDKGSDNKKKESDNKKKKSDDKKKESDNKKNKKGESKKKKNGGKEGRHGSAKSKGGSDDDKSSKNKRKGSEIADKNNQTKYGKVETNKGGVEQPRNKTAERLRAEAADELD

>BcaB06g27388

MSRFQHLLCFTIILATVTFFNVASAHVKIKPALPQSGDPIIIGDIEPYTVKVVSTFVADLEKECAKTEKFRYFFEKINAFSKYVCSVSKGKDHESHMKAKAGSLFQAIAALGTDESVSKGGEVNKLQKEKTEAMETVKMLQSIGEKITGGRNNKTEINEILQLTKKQQKEIKDGILKWLNVISLIAEKADEISMKSSSKSETKEEKSVEPRKLVATKSSTKSNKNSDKESDNKKKESDNKKNKKGESKKKKNGGKEGRHGSAKSKGGSDDDKSSKNKRKSSENADKNHRTKYGTVETKKGGVEQSRNKTAERLRAEAADELD

>BcaB07g30115

MPDFSSSIKCGVRSARNVPAKDVLEEEHVSEGKGLYTIRMQSNASATSKSTDAQITSQVDGLSNAMSELTAVKGRAPSNTDFRDAMLSMGKTLIEQKKSGSQSMTFQQRKKLVVEMVQWTKNTTREESEHRSIKCGVRSARNVPAKDVLEEEHVSEGKGLYTIRMQSNASATSKSTDAQITSQVDGLSNAMSELTAVKGRAPSNTDFRDAMLSMGKTLIEQKKSGSQIVSSVVYEAQGTFLLKMYLKRKFLRKAKDFTPFACKGMLLLMKRLKRECPATEGFKTFFSLFTSYVKFIKTASTTSESTDTQLTTKVDALTNAISELTGAKSGSNSNFRETMLSMGKTLIEQKRSSSQLMTHQQKKVLVVAMVQWTKTLVTLVKTAVETAGKSIDVSNLGLDVDVNSVVGNGSDESPKSDSPSDSDSPTATSSGSVTYEDTTGGGSGSPSGSPTDSPSGTTTDSPSGTTTDSPNDTTTDSPSESPTDSPSGTTTDSPSGTTTDSLSGTTTDSPSGSPTDSPSGTTTDSPSDTTTDSPSGTTTDSPSGSPTDSPSGTTTDSPSGSPTDSPSGSPTDSPSDTTTDSPSGSPTDSPSDTTTDSPSGSPTDSPSDTTTDSPSGTTTDSPSGTFTDSTGGTTTDSPSGSPTDSPSGTTTDSMSDTTTESPSGTTMDSLSGSPTDSPSGSPTDSPSGITTDSPSGTTTDSPNSPSESPTDSPSGSKSASGETSVKESGSDSEASAPNTNSESSESSTSSSSTTSAKEVEIQTSKEARSFIHGLEKKYAGTVQLDTFFEKLKTSMSASTKISNTDEKRFVSKMNSAVSSVSEAATTVSSKLAKSPEAKNRAESSQEKLMKTYKELEDVNSKIVSENKGKTVSSTQQSELRKTLSKWEQVTTQFVENLASSSSSSSSSSQSQQSQQSQQSHQSQQSQQGSAMKTQTD

>BcaB07g30116

MARFPLAICLMCIIVASTTVYEAQGSFLLRHYLRKLPRLANESEPFAFKIMLRFIDNLESLCSSKVEYKEFFSKLKAFLIFINSAAGKSSSSEFESQLKAHSEGLFKAITALGVKSSADTSKLIESLMSMGKVMAEYKRSGSLTMTSEQRRVLITSVMKWAQVIGQFVKTVREKTGEGDIDLPSLGIGGGGDDDAGRAGGGSSGSGSSSMGDGSAGGGSSGGGASSLGAGSAGGGSSGSGTSSMGAGSAGDGSSGSGSPYPGAGGGSSGSGTSSMGAGNTGMGDGSTGGSGSPSSESGTPSSETGNPADGGSGSPGGSPTDSSTAAGGSSMGGGGSSGATTGAAGGSSGDTGSTTGAGGGSNGDTGSTTGAVGGSSGATGSTTGAAGGSSGDTGSTTGAGGGSSNGGGSVDTQSMTGAGSGSGSGGTSAGGPSGIPTDDGSGSGESAGGETSGAGAGAGAGAGGSSGNTSNMSSKDSKITTGGSASEESTSGGASGGSTDTTATGESSMNNRDSYSDSTGGKTMGSPTGSPSGSTSGSGGSSFTGSETGTSSYQAGGASAGGPSGSITDSSAAGASSMNSGSSKTQTSAQGSTQGGSDLAEGASEGKSFKGKTGSMGYEGSSNYQKAHSKSSDKSSFSHSSEEKSSGNV

>BcaB07g30117

MARFPLAICLMCIIVASTTVYEAQGSFLLRHYLRKLPRLANESEPFAFKIMLRFIDNLESLCSSKVEYKEFFSKLKAFLIFINSAAGKSSSSEFESQLKAHSEGLFKAITALGVKSSADTSKLIESLMSMGKVMAEYKRSGSLTMTSEQRRVLITSVMKWAQVIGQFVKTVREKTGEGDIDLPSLGIGGGGSGTSSMGAGNTGMGDGSTGGSGSPSSESGTPSSETGNPADGGSGSPGGSPTDSSTAAGGSSMGGGGSSGATTGAAGGSSGDTGSTTGAGGGSNGDTGSTTGAVGGSSGATGSTTGAAGGSSGDTGSTTGAGGGSSNGGGSVDTQSMTGAGSGSGSGGTSAGGPSGIPTDDGSGSGESAGGETSGAGAGAGAGAGGSSGNTSNMSSKDSKITTGGSASEESTSGGASGGSTDTTATGESSMNNRDSYSDSTGGKPWGAPPGVPLVQLLVVVGALSPEVKLELHLTKLEEHPQVGRVEV

>BcaB07g30118

MKKVSLLCLCCITILATSLSLNVVSAHETAVNAPNTIKDIEPYISNRAIGFVLKLENDCPIREKLRSFFEKLKDLLKLESSVTPMIEDNEPKTFMSHMKSKADDLLQTMLMIGRGLLSSSVRKEMFKVIKSLTELHAAIGKVIMEKHIKGDGSMSLSLEQKNAVENAVSQWEVTITRIVKIVVEVKSESSSTVSGEESSTAEQNSTSVNETMLESNKGNLESMQENGNGINESNEDSKNNVAIEAKEDNRVEDVKGDANNEKQLEVQGGNIDTSLNTKEDVNSEVDAKPDGSSMTKNSEEAQGNDGVSIKDHNLENIGSEAGINDGDHTKDNGEETQENNGELVMNENSENNKDNKEVKNDGLVQTKTNHEISMKEKREHTQERSEVSMNGETMHSKSGGVQGESIGDSTKDVNLENKENLKGDANSKKPLEVQGGSIGDSHIDKNLDTKEDVKSEVDARKEMLSNSEEKSEESEKEMQVKTPTRSEESHKKIKAEVPATSDESEKEMQAETLTKNEELEKKMQAEAPTKSGESDKEMQAKAPSRSDESEKEMQARLQQGVRSQKRKYKQRLQQGMRNQVTKYKQRLHQGVTNLKKKSKQRLQQRVRNQRKTCKQRLQQGVRSQKRKYKQRLQQGVRNLKKKIQAETPTRSDKSEKEMQAETPTKTETLTKNEELEKKMQAEAPTKSGESDKEMQAKAPSRSDESEKEMQAETPTKSEESEKEMQAEAPTRNEESGNEIQAEAPSRSDESEKEKQAETPTKSEESEKDMQAEAPARSEESEKKIQAEAPTRSEESEKEIQAETPTRSDKSEKEMQAKTPAKSEESDKEMQAKAPAMSDESEKEMQAETPTKSEESEEEMQEEAPARSEESEKEIQAEAPTRSEESGKEMQAETPTRSDESEKEMQAETPAKSDESNKEMQANAHTESEESEKEMQAEAQARSEKFEKEIQAEAPARSKESEKEMQTKTHIESEESEKKMQAETPTKSEELEKEMQAKAPAKSGESDNEMHANAHTESEESEKEMQAETQARSENFEKEMQAEAHTKSAESEGKKQVEAQAGNEDSSMKKQEEFQETNVGEKKNQTVPKEEGSTDSKITEIKEGSKDGETVEKNGNIAETNRDKEDYKKEGSENGERAEIDGRKEVTVEEGSKDGKTLENNSSTEISMKKGSKDSKTVEVNEINKSKEKEAEKKKQKEHEENIFKKKEEEKRKERERNKLKNEEEKKKQKEHEEHISKKKEEEEKKKEHERHKLKKKEEEKKKQKENEEHTSKNKEEEKERAEKKQIEEKRRREKKQREEKKQKEHEEHISKKKEEEEKKKEHERHKLKKKEEEKKKQKENEEHTSKNKEEEKRKKHEKNKLKNEEEKKKQREYGENISKMKEEERMKEHERNKLKNEEEKKKQNKNREHISKMKEEEKRKEHERNKLKKNEEEKKKQKQHEEHISKKKEEEKRKEHEKDKLKNEEEKKKQKEHEEHISKRKEEEKKKEHERNKLKKNEEEKRRKKKRKGKSMKNTYPKERRREKERIENEEEKKKKKEHEEHIYKMKEEEKRKEHKKHISKKKDEEKIKEHERDKWKNEEEKKKQKEYEEHISEKKEEKKRKEHEKNKVKKKKEEKKKQKEHEEHISKKKEEKKRKKHERNKLKNEERGKKQKEYDEHIFKKKEEEKRKEHERNKLKNEEEKKKKKEHEEHIFKMKEEEKRKEHERNKSKKNEEQKTKQKEYEEPISKTNEEEKRKEHKRNKLKKNEEEKKKQKEHEEHIFKKKEEEKRKEHERNKLKVNEKDKQKLKEHEEHKSKKKEEEKRKEHEKNKFKKGEKDKKMKREYENNKSKKRKEDAKEKLKENVNISSDYKSKDHEKEMLANSEEKSEESEKEMQAKTPTRKRSEESEKEMQAETPTKSEESEEEMQEEAPARSEESEKEIQAEAPARSEESGKEMQAETPTRSEESEKEMQAETPAKTKSGETDKEMQANAHTESEESEKEMQAEAQARSEKFDKEIQAEAHTKSAESEKKKQGEAQAGNEDSSMKKHEEIQETNVGDKKDQSVSKEKDSTDSKTTEIKEGSEEDSKNGETVENNGNIAEINKDKEDYMKEDSENSEGAEIDGGKEVPVEEGSKDGQTLENNSSTENSMEEGSKDSKAVEVREGQDNSMEKSNESQEVTMEEGSKDGNTTKLNGSKDNSMEEGTVETSGVKEDSMKDKATEVQGNNNSSTSDTSMATEHVEGDNIDVNEDSTKNKTMEAQSGSAGGSTNSTTQETTESNDSMSNQNVQHGGNNANSMNNQTSESSTTEQKVTTDIESSTSKEVSSFISNLENKSPGTQAFQSFFQKLKDYMKYAWPVSSTFEATDLRSYMSEMTNMATKLSDAMSVLQARKLGSGLMKTTLQGYQQEVMKTLTLLQSVLSKAMSGQQSQNSSSLTLTLSQQQAIKEITSKWEHVMSQFVRVATESEKQFSMETSTGNGFHTKKSSSSTSSSSSSSSSSSSPGLDFNLLNGESPGMVDVNN

>BcaB07g32652

MARVHLLLCFAVLFASVTLFDVASAFLKLKPSLPKIEDPKTVGEVEEYTVKVVMVFVGDLEKECPKTSKFKTFFDKLRGFAKYVCPLKIFGKKDDADMKAKEAGILKTIASFAIGRIKSEIQEEKQEAIETFKFMKSLAGRILGGRKKEEKETTTLTPEMVKEIKDGILKWQTVIHKNNEGSDSTTTTPTTGGDSSSPNSGNSTGSPSNKPSAGSNPGAGTPSKDTNNQPEATTNTASSGSESTSQTKEVAVTEIETQTSEQVMTFLMNLEKKCPPKEEYKQFFEKLKSTMTGSAKASSPKKKGGLFAMIKGAVGKIGDAMQFIRSRIGNKSAEVKKSMETYQSEVIKNMEQLDAIYAKIITQNQSKKGGAMTCTPEQQAEIKQTITKWEQVTTQFVEVAIKSETSTSTSTSTSSSTGTAQAN

>BcaB07g32675

MARVHLLLCFTLLFASVTLFDAASAFVKLKPSLPQIEDPKTVGDVEGYTVQVVMVFVGDLEKECPKTSKFKMFFDKLRGFAKYVCPLKIFGKKDDADMKAKEAGILKTIASFAIGRIKREIQEEKQEAIETFKFMKSLAGRILGGRKKEEKATTTLTPEQLKEIKDGILKWQTVIVKITNTMVVSTTNSEGSAGSNPGAGIPSTDTDNQSQGTPSADTNNESQGTTGGSSSPNSGSATGSPSNKPSAGSNPGAGTPSTDTNNQSQGTPSADTNNESQGTTGSSSSPNSGSATGSPSNKPSAGSNPGAGTPSTDTNNQSQGTTNTASSGSATTSQTTEVTVTEVETQTSEQVMTFLMNLEKKCPPKEEYKQFFEKLKSTMTGSAKVSSPKKKGGLFGMIKGAVGKIGDAMQFIRSRIGNKSAEVKKSMETYQAEVIKNMEELNAIYAKIVSQNQSKKGGAMTCTPEQQAEIKTTITKWEQVTTQFVEVAIKSETSTTTSTSTSASTGTAQAN

>BcaNung06844

MARVHLLLCFTLLFASVTLLDPSLPQIKDPKTVGEVEGYTVEVVTIFVGNLEKECPKTICPLKIFGKKDGADMKAKEAGILKTIASFAIGRVSEREEKQEAIETFKFMKSLAGRILGGRKKEEKETTTLTVEQLKEIKDGILKWQTVIVKVANTMVVSTTTNNEGSAGTTEGSSSPNSGSATGSPSNKPSAGSNPGAGTPSTDTNNQSQGTTNTASSRSATTSQTTEVTVTEVETQTSEQVMTFLMNLEKKCPPKEEYKQFFEKLKSTMAGSAKVSSPKKKGGLFAMIKGAVGKIGDAMQFIRSRIGNKSAEVKKSMETYQAEVIKNMEELNAIYAKIVG

>BjuA008238

MKKVSLLCLCCITILATSLSFNVVSAHEIAVNAPNTIKDIESYISNRAIGFVLKLEDECPIREKLRSFFEKLKDLLKLESSVTPMIEENEPKTFMSHMKSKADNLLQTMLMIGRGLLSSSVRKEMFKVMKSLTELHAAIGKVITEKHIKGDESMSLSLEQKNAVENAVSQWEVTITRIVEIVVEVKSKSSSAASGEESSNTGQNSTSVNETTLESNKGNSESMQENSDNVSESNEDSKNDVAMKAKEGNRVEDVKGDVNNEKQLEVQGGSIDSNTNTSLDAKEDINSEVKANTDGSSITKNLEEAKGNSGVLTNDNNLENKGSEVTLNDGDHTKENGEETQENNGEVVMNENLENNKDNKEVKDDGLVETKKNHETSMEENREHTQRGTEVSMNEENMHTKSGNADSNKEKEVEVQGENIGDSTKDVNLENKEDFKGDANSEKKLEVQGGNNGDSQINKNLDTKEDVKSEVDARNDGSSMTEESETQGNDEVSTKNTNLENKGSEATLNDEYHTKENGKNTQENNAELVNDENLEKNKDNKEVKDNGLVETNTDHENSTEKKREHIQGGTEVSINEKKMHTNSGNADSNKEKEVEVQGESIGNTTKDLNLENEEDLKGDANSEKPLEVQEGSIGDSHINKNLDTKNGVKSEVDARNDGSSMTESEDTKGNGQFFTKDTTLENKESEARINDEDHTKENGEETKENNGELVKDENLENNKDNKEVKDDGFVETKTNHENSTEEKREHTQGGTEVSMNGEVMPTKGGNVDSNKESEVEFQGESIGNSTKDINLKDKEDVKSKVETNKNDESSMKEKQRYSQGNDRVSTKDQNLESIGAHKETMNHKTVEAMKNNGDQMKDKREENQQRESMKDENSENKEDKNELKDNELVETKTSNESSVKENKEEAQGSNEVSMNGQMDSLGGNVDSNNKEAVEFKKRHVEDSNKDSKPKVEAIKNEGNSMEEKGEVAEGNNGVSTEDKNLENIESEQELKNNIPAETKTNKETTMEENKEKDHVNNDESTYDGNKNESKDDKLDKTKKNKKDHMDKKWREAKGNEKSFMREHQKEVSVDNKGDKKEIKDGKPLEVKENKENKEETQRNSSGQYTNVDNKEGKDDKSKEAKENKEGSNNKIQEKVQGSGRDTTKVDRKKDRSSIDNMDIEVQKGSEESMKEAERKEHEKNKSTKIEKEEKKQKEHEEHKSKKKEEEKRKEHEKNKLKENDDDKKKQKEHSGYKSKKKEKEKRKEHKEHKSKRNEEERKDHERNKLKNEEEKKKQKEHEEHISKKKEEEKRKEYERNKSKKNEEEKKKQKEHEEHISKKKEEEKRKEHERNKLKNEEDKKKQKEHEERISKKKEDEKRKEYEKNKSKKNEEDRKKHKEHKEHISKKKAEAKRKEQEKNKSMKSEEEQKRQKEYEEHISKKKEENKRIEHERNKLMNGEEKKKQKEHEEHISKKKEEEKENEHEKNKSKKNEEEKNKQKEHQEHISRKKEEEKRIEHERNHLKNEEDKKKQKEHEEHISKKKKEEKMKEQENNKSKKNEEEKKKQKEHEEHTSKKNEEKKKKKKKKEHEKNKSKKNEEEKKKQKEHNEHLSKKKEEEHRIEHERNKLKNEEEKKKQKEHEEHISKNMEEKKRKEHEQNKSKKNEEEKNKQKENDEHMSRKKEEEKRIEHERNKLKNEEEKKKHKEHEEHKSKKKEEEKKKEHERNKLRKNEEENKKQKEHEKHISQKKEEDKRKEHEEHTSKKKEEEKRKEHEKNKAKKNEEEKMKEHEKNKSKKNEDKKSEHEQYKSKKTHKENKSNNNEEEETKKGEYEETKSKKNEEQKKREREEEKKKEHEENKFKKEEEEKKMKRDYDNNKSKQNKEDEKEKTKEDVKMSNDDKSKDHEKEMLANAKEKSEESEKEMQAETLSRSEESDKEMQADAPTKSEESVKEMQTKTHARSEESEKKIQAEAQARSEEHEKNMQAEAQAKSEVSEKETQTKDQAKNEEPEKEMQANAHTENEESEKEMQAEAHARSEESEKEMQEEAQVKSVESEKRKHTKAQAENEDSSMKKQEEIQETNVGEKKDQGDLKEEGSADGKTTEIKEGSIEEDSKDGETVENNGVNEEAMEEHSKDGKVVETNGGKEDSMEEGPKDEKLIKDNENTTEISGGKNSTVGDSSSDDGKIVENNGGKEDSLEEGEDGKTVEMHGGKNSTEEVSKDSTTEINGGKEDSIEEISKDGDIDEINRDKEDSMKEGSENGKTAEIDGRKEVPIEEGSKDGKTLENKNSTESSVEEGSKDSQTVEVNEGQDKSMEKGNGSQEVTMEEGSKDGNTTELNGSKDNSMEEGSTDGNTVETDGVKEDSMKDKATEVQGNNNSSTSDTSMATEVQGHNNSSTSDTSTDVKGDSAYINEDSTKNTTLEAQGGSAGGSTNGTIGETTKSNDSMSNQNVQHVEGDSIDINEDSTKNKTMEAQGGGVGDSTNGEKEETTESNDTMNNQNVQHGENNANSTNNQTSESSSTEKKVTTDIESSTSKEVTSFISNLEHKSPGTQEFQSFFQKLKDYMKYAWPVSSTFEATDSRSYMSEMTNMATKVSDAMAVLQAKKLGSGLMKTTLQGYQQEVMKTLTILQSVLSKAVSGQQSQNSGSLTLTLSQQQAIKEITLKWEQVMSQFVRVATESEKQFSMETSTGNGFHMKKSFSSSSSSSSSSSSSPGLDFNLNSESPEMVDVNS

>BjuA008242

MANKNVLGLCLMFLVVSSVVYEAQGTFLLKMYLKRNFLRNARNFTPFACKGMLLLISRLKRGCPATEGFKTFFSLFTSYVKFIKTASTTSNTDTQLTTKVDALTNAISVLTGAKSGSNSNFRETMLSMGKTLIEQKRSTSQRMTHQQKKMLVAAMVQWTKTVVTLVKTAVETAGKSIDVSNLGLDVDVNSIVGSGSDESPESGSGSNTNSGSIIYEDTTGGGSGSPSGSPGCSPSSTTTTQSDGSTMGSQSGTATESPSGTTTESPSGTTTDSPSGSPTDTPSGTTTETPSGTGTGSPSGSPTDSPSGATTDSPSETTTESPTRWQPQQPRVAQPQQPSGATTTARVYTKGPSGSPTDSPSGETTDSPTGATTESPSGTTTESPNGSPTDSPSGTATDSPSSTTTGGPSGSPTDSPSGSPTDSPSGSPTDSPSGSPTASEDATSNTNSQSSQSSTSSSSTTSAKEVEIQTSKEARSFIHGLEKKYAGTVQLDTFFEKLKTSMSASTKISNTDEKRFVSKMSSAVSSVSEAATTVTSKLAKSPEAKNRMESSQEKLMNTYKELEEVNSKIVSENKGKTVSSAQKSELKQILSKWEQVTTQFVENLVSSSSSSSSSSQSQQSQQSQKSHQSEQSQQGSTMKTETN

>BjuA015719

MSRFQNLLCFTILLATVTFFNVASAHVKIKLALPQTGDPISVGDVEPYTVKIVSTFVADLEKECAKTEKFRHFFEKVNAFSKCVCSVSMDHESHMKAKAGSLFQAISALGSDENGSKGGMVNKLQKEKTEAMETVKMLQSIGEKITGRQNNKTEINGTLKLTTKQQKEIKDGILKWVKVITQIAKQADEISMESETKKESKEENYVGPRILVQTKSSTKSDKNSDKKSGNKKNNKGESKKKKRGGKEGRQGSAKSKGGSNDDKSSKNKGKSSENAKNQQSKYGKVEKKKGGVDQSRNKTAERLRAEAADELD

>BjuA043209

MEHVSSATTATTTSSTTVAELEAETSKEVMEFIMKLEKKCPPKEEYKSFFEKLKATMVASAKVTQEKKKGFFSTAAGKISDAVSFIGSKFTGKSPEVKKSMETYQQEVAKSLQELEAIHKKIIEANQGKVEGSVAVTAEQKTEIKQTITRWETVTTQFVETAIQTEAASNTTVGVDKVKLP

>BjuA043235

MAKFPLGICLMFMITTSTIYEVQGHFLLDHYMKKIPKISSEFEPFAFKGILSFIDHLEGLCPLKVEYKEFFTKLKDFMAFINSASGSSAEFHTQLKTKSEELFKAITKMGGTAGASAHTTKLIESLVSMGKTLAEYKRSGSQTLTSEQRTELVTSMSRWAQTIGQFVKSVTETNGGGNIDLKSLGCGGATGISTSMETGSTATGSTSMETGSTGGSGSPSGDTGSPSAETGSPAGSESGSPSNETPAEGGSGSPSGSPSDSSSGAGSTGSETSAEAESTAGAGSGVGAGETPGAAGPSGSPTDSPSESEESSKDESSKEESSKDESSKDESSKDE

>BjuA043236

YNARAETGSSAKAKETSEDEKAEKKDEKKAEKNEQKAEKKAEKNEQKAEKNEQKAATSSAKTKEASSTKAGAAFRDTTGGSAGSPRGSPTAGKDKTAGSPRGSPTAAKEKTSVKGTGNAAGSVNQQQSNAGSSRGSGSAAASTNQQQSNAASRGSAASTNQESKKTASSKSSSTTSVTEIEKETSQETMSFISGLEKRFAQKAELKPFFEKLKASMTASSRVSSTKSEQEYTNTAKSTTGKLSDAMTFVGSRFSKSAEMKNTIQTTQQQLIKNLQQFQNLNSQIVGEQKVSSTKETEIKKTMSKIEQVTTQFVETAASSSSSSSKQETAASQQSQKQTMASSKKETAASSQQQTQQQTQQQTQQQTQQENGMGRLKTN

>BjuA029859

MARVNLLLCFTLVFASVTLLDVTSAFLKLKPSLPQIEDPKTVADVEGYTVNVVMVFVGDLEKECPKTSKFKMFFDKLRGFAKYVCPLKIFGKKDDTDMKAKEAGILKSIASFAIGRIKSEIQEEKQEAIETFKFMKSLAGRILGGRKKEEKATTTLTPEQLKEIKDGILKWQTVIVKITNTMVVSTTNTEGSAGSNPGAGTPSTDTNNESQGTPSADKNNKSQGTPSTDTNNESQGTTGGSSSPNSGSATGSPSNKPSTGSNPGAGTPSTDTNNQSQGTKNTASSGSTTTSQTTEVTVTEVETQTSEQVMTFLMNLEKKCPPKEEYKQFFEKLKSTMAGSAKVSSPKKKGGLFGMIKGAVGKIGDAMQFIRSRIGNKSAEVKKSMETYQAEVIKNMEELNAIYAKIVTQNQSKKGGAMTCTPEQQAEIKTTITKWEQVTTQFVEVAIKSETSTTTSTSSSTGTAQPN

>BjuA034744

MARVHLLLCFTLLFASVTLFDAASAFLKLKPSLPQIEDPKTVGDVEGYTVQVVMVFVGDLEKECPKTSKFKMFFDKLRGFAKYVCPLKTSGKKDDDDMKAKEAGILKTIASFAIGRIKREIQEEKQEAIETFKFMKSLAGRILGGRKKEEKATTTLTPEQLKEIKDGILKWQTVIVKITNTMVVSTTNTEGSAGSNPGAGTPSTDTNNESQGTPSADKNNKSQGTPSTDTNNESQGTTGGSSSPNSGSATGSPSNKPSTGSNPGAGTPSTDTNNQSQGTKNTASSGSTTTSQTTEVTVTEVETQTSEQVMTFLMNLEKKCPPKEEYKQFFEKLKSTMAGSAKVSSPKKKGGLFFMIKGAVGKIGDAMQFIRSRIGNKSAEVKKSMETYQTEVIKNMEELNAIYAKIVSQNQSKKGGAMTCTPEQQAEIKTTITKWEQVTTQFVEVAIKSETSTTTSTSTSTSDSRGTAQAN

>BjuB008293

TTPSTPTTSTPTPTTPTTPSTPTRSTPTPSTPTSSTPTPTTPSTPTTPTTSTGATETDTGATKGSPSGTPTDSPSGETPTNTNSQTSESTSSTTSAKQVETQTSQEVMSFISGLEKKYAGKAELNTFFEKLKSTMSATSKIATTDEKTFVSGVKSAAGKLSEAAETVTEKVGKSAESKQKIESSQEQLMKTFKELEDVKTKIVSESKGKTVSSTQQTELKQTLTKWEQVTTQFVETAASSSSASSSSQSSQSHQSQQSQQSHQSHQSQHTQQTQQTQQGSTMKAQTQT

>BjuB024425

MSRIQNLLCFTILLATVTFFNVASAHVKIKFALPQTGGPISVGDVEPYTVKVVSTFVADLEKECAKTEKFRHFFENVNAFSKCVCSVSKVEDHESHMKAKAGSLFQAISALGSDENGSKGGKVNKLQKEKTEAMETVKILQSIGEKITGGKNNKTEINGTLKVTTKQQKEIKDGILKWLDVITRIAKKADEISMESSSKSVGPRILVETKSSTKSNENSDKGSDNKKKESDNKKKKSDDKKKESDNKKNKKGESKKKKNGGKEGRHGSAKSKGGSDDDKSSKNKRKGSENADKNNQTKYGKVETNKGGVEQPRNKTAERLRAEAADELD

>BjuB029498

MARVHLLLCFAVLFASVTLFDVASAFLKLKPSLPKIEDPKTVGEVEEYTVKVVMVFVGDLEKECPKTSKFKTFFDKLRGFAKYVCPLKIFGKKDDADMKAKEAGILKTIASFAIGRIKSEIQEEKQEAIETFKFMKSLAGRILGGRKKEEKETTTLTPEMVKEIKDGILKWQTVIVKITNTMVVSSTKNNEGSDSTTTTPTTGGDSSSPNSGNSTGSPSNKPSAGSNPGAGTPSKDTNNQPEATTNTASSGSESTSQTKEVAVTEIETQTSEQVMTFLMNLEKKCPPKEEYKQFFEKLKSTMTGSAKASSPKKKGGLFAMIKGAVGKIGDAMQFIRSRIGNKSAEVKKSMETYQSEVIKNMEQLDAIYAKIITQNQSKKGGAMTCTPEQQAEIKQTITKWEQVTTQFVEVAIKSETSTSTSTSTSSSTGTAQAN

>BjuB042842

MKKVSLLCLCCITILATSLSLNVVSAHETAVNAPNTIKDIEPYISNRAIGFVLKLENDCPIREKLRSFFEKLKDLLKLESSVTPMIEDNEPKTFMSHMKSKADDLLQTMLMIGRGLLSSSVRKEMFKVIKSLTELHAAIGKVIMEKHIKGDGSMSLSLEQKNAVENAVSQWEVTITRIVKIVVEVKSESSSAVSGEDSSTAEQNSTSVNETMLESNKGNLESMQENGNGINESNEDSKNNVAMEAKEDNRVEDVKGDANNEKQLEVQGGNIDTSLNTKEDVNSEVDAKPDGSSMTKNSEEAQGNDGVSIKDHNLENIGSEAGINDGDHTKDNGEETQENNGELVMNENSENNKDNKEVKNDGLVQTKTNHEISMKEKREHTQERSEVSMNGETMHSKSGGVQGESIGDSTKDVNLENKENLKGDANSKKPLEVQGGSIGDSHIDKNLDTKEDVKSEVDARKEMLSNSEEKSEESEKEMQVKTPTRSEESHKEIKAEVPAMSDESEKEMQAETLTKSEELEKEMQAEAPANSGESDKEMQAEAPARSEESEKEMQAEAPTKSEESEEEIQAEAPTRSEESGNKIQAETPERSEESEKEMQAETPTKSEESEEEMQEEAPARSEESEKEIQAEAPARSEESEKEMQAEAPTKSEESEEEIQAEAPTRSEESGNKIQAETPERSEETEKEMQAETPTKSEESEEEMQEEAPARSEKSEKEIQAEAPARSEESGKEMQAETPTRSEESEKEMQAETSAKSDESDKEMQAKAPAMSDESEKEMQTEAPTKSEESKKEMQAEAPARIEKSDKEIQAEVPGRSDESEKEMQAETPIKNDELEKEMQAEAPSKSGESNKEMQANAHTESEESEKEMQAEAQTRSEKFEKEIQAEAPARSKESEKEMQTKTHIESEESEKKMQAETPTKSEELEKEMQAKAPAKSGESDNEMHANAHTESEESEKEMQAETQARSEKFEKEMQAESHTKSAESEGKKQVEAQAGNEDSSMKKQEEFQETNVGEKKNQTVPKEEGSTDSKITEIKEGSKDGETVEKNGNIAETNRDKEDYKKEGSENGERAEIDGRKEVTVEEGSKDDHEKEMLANSEEKSEESEKEMQAKTPTRSEESHKEIKAEAPARSDESEKEMQAETPIKSEELEKEMQAEAPANSGESDKEMQAEAPARSEESEKEMQAETPTKSEESEEEMQEEAPARSEESEKEIQAEAPARSEESEKEMQAEAPTKSEESEEEIQAEAPTRSEESGNKIQAETPERSEETEKEMQAETPTKSEESEEEMQEEAPARSEKSEKEIQAEAPARSEESGKEMQAETPTRSEESEKEMQAETSAKSDESDKEMQAKAPAMSDESEKEMQTEAPTKSEESKKEMQAEAPARIEKSDKEIQAEVPGRSDESEKEMQAETPIKNDELEKEMQAEAPSKSGESNKEMQANAHTEIEESEKEMQAEAQTRSEKFEKEIQAEAPARSKESEKELQTKTHIESEESAKKMQAETPTKSEELEKEMQAKAPAKSGETDKEMQANAHTESEESEKEMQAEAQARSEKFDKEIQAEAHTKSAESEKKMQGEAQAGNEDSSMKKHEEIQETNVGDKKDQSVSKEKDSTDSKTTEIKEGSEEDSKNGETVENNGNIAEINKDKEDYMKEDSENSEGAEIDGGKEVPVEEGSKDGQTLENNSSTENSMEEGSKDSKAVEVREGQDNSMEKSNESQEVTMEEGSKDGNTTKLNGSKDNSMEEGTVETSGVKEDSMKDKATEVQGNNNSSTSDTSMATEVQGHNNSSTSDTTTDVKGGSININEDSTKNKTMEAQGGSTGGSTNGTTQETTESNDSMNNQNMQHVEGDNIDVNEDSTKNKTMEAQSGSAGGSTNSTTQETTESNDSMRNQNVQHGGNNANSMNNQTSESSTTEQKVTTDIESSTSKEVISFISNLENKSPGTQAFQSFFQKLKDYMKYAWPVSSTFEATDLRSYMSEMTNMATKLSDAMSVLQARKLGSGQMKTTLQGYQQEVMKTLTLLQSVLSKAMSGQQSQNSSSLTLTLSQQQAIKEITSKWEQVMSQFVRVATESEKQFSMETSTGNGFHTKKSSSSSSSSSSSSSSNSSPGLDFNLLNGESPGMVDVNN

>BjuB042843

MARFPLAICLICIIVASSTVYEAQGSFLLRHYLRKLPRLANESEPFAFKIMLRFIDNLESLCSSRVEYKEFFSKLKAFLIFINSAAGKSSSSEFESQLKAHSEGLFKAITALGVKSSADTSKLIESLMSMGKVMAEYKRSGSLTMTSEQRRVLITSMMKWAQVIGQFVKTVREKTGEGDIDLPSLGIGGGGDDDAGRAGGGSSGSGSSSMGDGSAGGGSPGGGASSLGAGSAGGGSSGSGTSSMGAGSAGDGSSGSGSPYPGAGGGSSGSGTSSMGGGNTGMGDGSTGGSGSPSSDSGTPSSETGNPADGGSGSPGGSPTDSSTAAGGSSMGGGGSSGDTGTTTGAGGGSSGDTGGTTGAGGGSNGDTGSTTGAVGGSSGATGSTTGAAGGSSGDTGSTTGAGGGSSNGGGSVDTQSMTGAGSGSGSGGTSAGGPSGIPTDDGSGSGESAGAGGSGGNTSNMSTEESSSMGGGKSSKDSKITTGGSASEESTSGGASGGSTDTTATGESSMNNRDSYSDSTGGNTMGSPTGSPSGSTSGSGGSSFTGSETGTSSYQAGGASAGGPSGSTTDSSAAGASSMNSGYSAEGASEGKSFKGKTGSMGYEGSSNYQKAHSKSSDKSSFSHSSEEKSSGNV

>BjuB042844

KVHRQSTSGTTTDSPSGTTRTALNGTTTESPSGTTTDSPSGSPTDTPSGTTTETPSGTGTDSPSGSPTDSPSGTTTDSMSDTTTESPSGTTMDSLSGSPTDSPSGSPTDSPSGITTDSPSGTTTESPNSPSESPADSPSGSKSASGETSVKESGSDSEASAPNTNSESSESSTSSSSTTSAKEVEIQTSKEARSFIHGLEKKYAGTVQLDTFFEKLKTSMSASTKISNTDEKRFVSKMNSAVSSVSEAATTVSSKLAKSPEAKNRAESSQEKLMKTYKELEDVNSKIVSENKGKTVSSTQQSELRKTLSKWEQVTTQFVENLASSSSSSSSSSQSQQSQQSQQSQQSQQSQQGSTVKTQTN

>BjuB032901

MARVHLLLCFTVLFASVTLFDVASAFLKLKPSLPQIEDPKTVGDVEGYTVKVVMVFVGDLEKECPKTSKFKMFFDKLRGFAKYVCPIKIFGKKDDDDMKAKEAGILKTIASFAIGRIKSEIQEEKQEAIETFKFMKSLAGRILGGRKKEEKETTTLTPEQLKEIKDGILKWQTVIVKITNTMVVSTTTNKEGSDSTTTTTPTGGSSSPNSGSSAGSPSNKPSAGTNSGAGTPSKDTNNQPEDTKNSASSGTGSTSQSTKEVTVTEVETQTSEQVMTFLMNLEKKCPPKEEYKQFFEKLKSTMTGSPKVSSPKKKGGLFTMIKGAVGKIGDAMQFIRSRIGNKSAEVKKSMETYQTEVIKNMEELNAIYAKIVSQNQSKKGGALTCTPEQQAEIKQTITKWEQVTTQFVEVAIKSETTTTSTSSSSSSSTEKAQAN

>BjuB044503

MFMITTSTIYEVQGHFLLDHYLKKIPTISSEFEPFASKGILSFIDHLEGLSPPKEEYKEFFTKLKEFMAFINSASGSSSEFHSQLKTKSEELFKAITKMGGKAGSSAHTSKLIESLVSMGKTFAEYKRSGSQTLTSEQRTELVTSMSKWAQVIGQFVKTATEKSGDGNIDLKSLGCGGGTSGSISSSSTETGSSASGSSSMETAGSTGGSGSPSEETGSPSAETGSPASGDGPGDSGGPSDSGSPSNETPAEGGSSSPSGSPSDSSSGPGSTGVESPAEAGSAAGAGSGTGDGETPGAAGPSGSPTESGESSKDESSKDESSKDEKATESGESSKDASGANSNEESSTETQSETGAESGSTDGGASAGGPSGSPTESSGPGDVGAGGPSGETSGEAGAGGETSGEAGAGGETSGDAGAGGPSGETSGAGGETSGDAGAGGPSGETSGAGGETSGDAGAGGETSGAGGPSGSTSDMSAGGASTSGGASGTSTQTSAEAESTMNSGGSFADTTGGSSGMEGPASSPSGSASGGSSETSSFSEMSSHEAGGSSAGAPSGSAIGGSAEGETSTKGKMSYERSASYRKSHSQTSGHSSFKHSSEEKNTGSS

>BjuB044504

MARISLGLCLMLVVASSVIYEAQGHFLLKNYLKTQFPSKGNEFTPYVNRGLTELLTDLERFCPPTPEFKGFFTEFKSYMSFIETSSSSSSKNIDMEMTKKADGLFRAMSAISGGAGQKSAEAGSFKSTMISMGKTLVEQKRSTTTITSTEKKTLVTSMVQWTKTIATVVKTASEKKGKKIDINSFGLDVDVKSIVSVSGSSHSSSSSTKTSSESSSKSSSYNARAETGSSAKAKETTEKGEKKDNAEKKPEKKEEKKPEKKEEKPEKKEEKDSSSAKTKETSSTKAGASGSTKTGSSFKDTTGGSAGSPRGSPTAAKDKTSGSPTSDKGRTSEKESGNASSVKQQSNNAASRGSASSMNQQQSKKSESASSSSTTSVTEIEKETSQETSSFISGLEKRFSQKAELRPFFEKLKASMTASSRVSSTKSAQEFTSTAKSTTGKLSDAMTFVGSRFSKSAEMKSNIETTQEKLMKSLKEFQDLNSRIVGEQKVSTTKETQIKQTMSKIEQVTTQFVETAASSSSQQSQQSQKQRTATSQQQTAASSQQQTAASSQQQNEQQNGMGRLKTN

>BjuB044536

MEHVSSATTASSSKSTTVSELEVETSKEVMEFIMKLEKKCPPKEEYKSFFEKLKSTMVGSTKVTLEKKKGFFSAAAGKISDAMSFIGSKLSGKSAEVKKSMENYQQEVTKSLQELETLHKKIIEANQGKADGSATVTAEQKTEIKQTITRWETVTTQFVETAIQTENASNTTVGVDKVKLP

>BnaA02T0349400ZS

MKKVSLLCLCCITILATSLSFNVVSAHEIAVNAPNTIKDIESYISNRAIGFVLKLEDECPIREKLRSFFEKLKDLLKLESSVTPMIEENEPKTFMSHMKSKADNLLQTMLMIGRGLLSSSVRKEMFKVMKSLTELHAAIGKVITEKHIKGDESMSLSLEQKNAVENAVSQWEVTITRIVEIVVEVKSKSSSAASGEESSNTGQNSTSVNETTLESNKGNSESMQENSDNVSESNEDSKNDVAMKAKEGNRVEDVKGDVNNEKQLEVQGGSIDSNTNTSLDAKEDINSEVKANTDGSSITKNLEEAKGNSGVLTNDNNLENKGSEVTLNDGDHTKENGEETQENNGEVVMNENLENNKDNKEVKDDGLVETKKNHETSMEENREHTQRGTEVSMNEENMHTKSGNADSNKEKEVEVQGENIGDSTKDVNLENKEDFKGDANSEKKLEVQGGNNGDSQINKNLDTKEDVKSEVDARNDGSSMTEESETQGNDEVSTKNTNLENKGSEATLNDEYHTKENGKNTQENNAELVNDENLEKNKDNKEVKDNGLVETNTDHENSTEKKREHIQGGTEVSINEKKMHTNSGNADSNKEKEVEVQGESIGNTTKDLNLENEEDLKGDANSEKPLEVQEGSIGDSHINKNLDTKNGVKSEVDARNDGSSMTESEDTKGNGQFFTKDTTLENKESEARINDEDHTKENGEETKENNGELVKDENLENNKDNKEVKDDGFVETKTNHENSTEEKREHTQGGTEVSMNGEVMPTKGGNVDSNKESEVEFQGESIGNSTKDINLKDKEDVKSKVETNKNDESSMKEKQRYSQGNDRVSTKDQNLESIGAHKETMNHKTVEAMKNNGDQMKDKREENQQRESMKDENSENKEDKNELKDNELVETKTSNESSVKENKEEAQGSNEVSMNGQMDSLGANVDSNNKEAVEFKKRHVEDSNKDSKPKVEAIKNEGNSMEEKGEVAEGNNGVSTEDKNLENIESEQELKNNIPAETKTNKETTMEENKEKDHVNNDESTYDGNKNESKDDKLDKTKKNKKDHMDKKWREAKGNEKSFMREHQKEVSVDNKGDKKEIKDGKPLEVKENKENKEETQRNSSGQYTNVDNKEGKDDKSKEAKENKESSNNKIQEKVQGSGRDTTKVDRKKDRSSIDNMDIEVQKGSEESMKEAERKEHEKNKSTKIEKEEKKQKEHEEHKSKKKEEEKRKEHEKNKLKENDDDKKKQKEHSGYKSKKKEKEKRKEHKEHKSKRNEEERKDHERNKLKNEEEKKKQKEHEEHISKKKEEEKRKEYERNKSKKNEEEKKKQKEHEEHISKKKEEEKRKEHERNKLKNEEDKKKQKEHEERISKKKEDEKRKEYEKNKSKKNEEDRKKHKEHISKKKAEAKRKEQEKNKSMKSEEEQKRQKEYEEHISKKKEENKRIEHERNKLMNGEEKKKQKEHEEHISKKKEEEKENEHEKNKSKKNEEEKNKQKEHQEHISRKKEEEKRIEHERNHLKNEEDKKKQKEHEEHISKKKKEEKMKEQENNKSKKNEEEKKKQKEHEEHTSKKNEEKKKKKKKKEHEKNKSKKNEEEKKKQKEHNEHLSKKKEEEHRIEHERNKLKNEEEKKKQKEHEEHISKNMEEKKRKEHEQNKSKKNEEEKNKQKENDEHMSRKKEEEKRIEHERNKLKNEEEKKKHKEHEEHKSKKKEEEKKKEHERNKLRKNEEENKKQKEHEKHISQKKEEDKRKEHEEHTSKKKEEEKRKEHEKNKAKKNEEEKMKEHEKNKSKKNEDKKSEHEQYKSKKTHKENKSNNNEEEETKKGEYEETKSKKNEEQKKREREEEKKKEHEENKFKKEEEEKKMKRDYDNNKSKQNKEDEKEKTKEDVKMSNDDKSKDHEKEMLANAKEKSEESEKEMQAETLSRSEESDKEMQADAPTKSEESVKEMQTKTHARSEESEKKIQAEAQARSEEHEKNMQAEAQAKSEVSEKETQTKDQAKNEEPEKEMQANAHTENEESEKEMQAEAHARSEESEKEMQEEAQVKSVESEKRKHTKAQAENEDSSMKKQEEIQETNVGEKKDQGDLKEEGSADGKTTEIKEGSIEEDSKDGETVENNGVNEEAMEEHSKDGKVVETNGGKEDSMEEGPKDEKLIKDNENTTEISGGKNSTVGDSSSDDGKIVENNGGKEDSLEEGEDGKTVEMHGGKNSTEEVSKDSTTEINGGKEDSIEEISKDGDIDEINRDKEDSMKEGSENGKTAEIDGRKEVPIEEGSKDGKTLENKNSTESSVEEGSKDSQTVEVNEGQDKSMEKGNGSQEVTMEEGSKDGNTTELNGSKDNSMEEGSTDGNTVETDGVKEDSMKDKATEVQGNNNSSTSDTSMATEVQGHNNSSTSDTSTDVKGDSAYINEDSTKNTTLEAQGGSAGGSTNGTIGETTKSNDSMSNQNVQHVEGDSIDINEDSTKNKTMEAQGGGVGDSTNGEKEETTESNDTMNNQNVQHGENNANSTNNQTSESSSTEKKVTTDIESSTSKEVTSFISNLEHKSPGTQEFQSFFQKLKDYMKYAWPVSSTFEATDSRSYMSEMTNMATKVSDAMAVLQAKKLGSGLMKTTLQGYQQEVMKTLTILQSVLSKAVSGQQSQNSGSLTLTLSQQQAIKEITLKWEQVMSQFVRVATESEKQFSMETSTGNGFHMKKSFSSSSSSSSSSSSSPGLDFNLNSESPEMVDVNS

>BnaA02T0349500ZS

MTRFPLAICLMCIIVASSTVYEAQGGFLLRHYLSKLPRLANEFEPFAFKIMIRFIDNLESLCSSKVEYKEFFSKLKAFLIFINSTAGKSSSSEFESQLKAHSEGLFKAITALGVKASADTSKLIESLMSMGKVMAEYKRSGSLTMTSEQRRVLITSMMKWAQVIGQFVKTVREKTGDGDIDLPSLGIGGGDDDAGSAGGGSSGSGSSSMGAVSAGGGSSGGGASSLGAGSAGGGSSGGGTSSMGAGSAGDGSSGSGSPYPGAGGTGGGSSGSGSSTMGAGNTGMGDGSTGGSGNPSSDSGTPSSETGSHAGGGGGSPGGSPTDSSTAAGGSSMGGGGSSSDTGSTTGAGGGSSGDTGSTTGAAGGSSGDAGSITGAAGGSSGDTGSTTGAGGGSSGDTGSTTGAAGGSSGDTGSTTGAAGGSSGDTGSTTGAAGGSSGDTGSTTGATGGSSGDTGSTTGVGGGSSGDTGSTTGAGGGSSGDTGSTTGATGGSSGDTGSTMGAEGGSSSDTGSTTGVGGGSSGAGGGSSGDTRSTTGAAGGSSGDTGSTMGAEGGSSGDTGSTTGAGGGSSGDTGSTTGAGGRSSGDTGSTTGAGGGSSGDTKSTTGAAGGSSGDTGSTTGAGGGSSGDTGSTTGAGGGSGSGGTSAGGASGSPTDDSSGSGESAGGETSGAGGSSGNTSNMSTEESSSMGGGKSSKDSKIATGGNASEESTSGGASGGSTKTTAAGERSMNNGDSYSDSTGGNTMGSPTGSPSGRGGSSFTGSETGSSSYQAGGASAGGPSGSTTDSSAAGANSMNSGYSAKGSSTTSAQGSTQGGSGLAEGASEGKSFKGKTGSTRYEGSSNYQKTHSKSLDKSSFSHSSEDKSSGNV

>BnaA02T0349600ZS

MANKNVLGLCLMFLVVSSVVYEAQGTFLLKMYLKRNFLRNARNFTPFACKGMLLLISRLKRGCPATEGFKTFFSLFTSYVKFIKTASTTSNTDTQLTTKVDALTNAISVLTGAKSGSNSNFRETMLSMGKTLIEQKRSTSQRMTHQQKKMLVAAMVQWTKTVVTLVKTAVETAGKSIDVSNLGLDVDVNSIVGSGSDESPESGSGSNTNSGSIIYEDTTGGGSGSPSGSPGCSPSSTTTTQSDGSTMGSQSGTATESPSGTTTESPSGTTTDSPSGSPTDTPSGTTTETPSGTGTGSPSGSPTDSPSGATTDSPSETTTESPSDTTMGSPSGSPTDSPSGETTDSPSETTMGSPSGSPTDSPSGETTDSPSETTMGSPSGSPTDSPSGATTESQSDTTTESPSDTNTGSPSGSPTDGPSGATTESQSDTTTESPSGSPTDSPSGATTDSPSGTTTDSPSGATTDSPSDTTKGSPSGSPTDSPSGETTDSPTGATTESPSGTTTESPNGSPTDSPSGTATDSPSSTTTGGPSGSPTDSPSGSPTDSPSGSPTDSPSGSPTASEDATSNTNSQSSQSSTSSSSTTSAKEVEIQTSKEARSFIHGLEKKYAGTVQLDTFFEKLKTSMSASTKISNTDEKRFVSKMSSAVSSVSEAATTVTSKLAKSPEAKNRMESSQEKLMNTYKELEEVNSKIVSENKGKTVSSAQKSELKQILSKWEQVTTQFVENLVSSSSSSSSSSQSQQSQQSQKSHQSEQSQQGSTMKTETN

>BnaA02T0353100ZS

MSFIMNLEKKCPPKEEYKSFEKLKDTMVASAAEKKKGFFSGVKGKISDAMSFIKTSRNTYGSISMEKYHQEVVKALQELETIHSKIISATQGKKDESL

>BnaA04T0111000ZS

MSRFQNLLCFTILLATVTFFNVASAHVKIKLALPQTGDPISVGDVEPYTVKIVSTFVADLEKECAKTEKFRHFFEKVNAFSKCVCSVSMDHESHMKAKAGSLFQAISALGSDENGSKGGMVNKLQKEKTEAMETVKMLQSIGEKITGRQNNKTEINGTLKLTTKQQKEIKDGILKWVKVITQIAKQADEISMESETKKESKEENYVGPRILVQTKSSTKSDKNSDKKSGNKKNNKGESKKKKRGGKEGRQGSAKSKGGSNDDKSSKNKGKSSENAKNQQSKYGKVEKKKGGVDQSRNKTAERLRAEAADELD

>BnaA06T0372200ZS

MEHVSSATTATTTSSTTVAELEAETSKEVMEFIMKLEKKCPPKEEYKSFFEKLKATMVASAKVTQEKKKGFFSTAAGKISDAVSFIGSKFTGKSPEVKKSMETYQQEVAKSLQELEAIHKKIIEANQGKVEGSVAVTAEQKTEIKQTITRWETVTTQFVETAIQTEAASNTTVGVDKVKLP

>BnaA06T0375400ZS

MARISLALCLMLVVTSSVIYEARGHFLLKDYLKTKFPSKSSEFTPYVNRGLTEFLTDLERFCPPTPEFKSFFTEFKSFFSSIETSSSTSQNVDVEKKGDGLFKAVSAITGGAGQQSAEAGSFKSTMISMAKTLVEQKKSTTTITSTEKKTLVTSMVQWTKTIATTVKTACEKKGKKIDINSFGLNVDVNSVMTVSESRQSSSSSSKSSHESSSKSSSYAARAETAASAKAKETSGARAETGATAKAKETTGASAEKDEKAEKKDEKKAEKAEKKAEKNEQKAEKNEQKAATSSAKTKEASSTKAGAAFRDTTGGSAGSPRGSPTAGKDKTAGSPRGSPTAAKEKTSVKGTGNAAGSVNQQSNAGSSRGSGSAAASTNQQQSNAASRGSAASTNQESKKTASSKSSSTTSVTEIEKETSQETMSFISGLEKRFAQKAELKPFFEKLKASMTASSRVSSTKSEQEYTNTAKSTTGKLSDAMTFVGSRFSKSAEMKSTIQTTQQQLIKNLQQFQNLNSQIVGEQKVSSTKETEIKKTMSKIEQVTTQFVETAASSSSSSSKQETAASQQSQKQTMASSKKETAASSQQQTQQQTQQQTQQQTQQENGMGRLKTN

>BnaA06T0375500ZS

MAKFPLGICLMFMITTSTIYEVQGHFLLDHYMKKIPKISSEFEPFAFKGILSFIDHLEGLCPLKVEYKEFFTKLKDFMAFINSASGSSAEFHTQLKTKSEELFKAITKMGGTAGASAHTTKLIESLVSMGKTLAEYKRSGSQTLTSEQRTELVTSMSRWAQTIGQFVKSVTETNGGGNIDLKSLGCGGATGISTSMETGSTATGSTSMETGSTGGSGSPSGDTGSPSAETGSPAGSESGSPSNETPAEGGSGSPSGSPSDSSSGAGSTGGSESSAEAESTAGAGSGAGAGETPGAAGPSGSPTDSPSESEESSKDESSKDESSKDESSKDESSKDESSKDESSKDDSSKDESSKDENSKDESSKDESSKDESSKDDSSKDESSKDESSKDESSKDESSKDESSKDESSKDESSKDESSKDESSKDESSKGESSAESGESSKEASGGSSTETQSETGAESGSTAGGPSGEPSGDAGAGAPSGSTSDMSAGGASASGGASETSTQTSAEGESSMNSGGSYADTTGGSAEGSASSPSGSASGSSETSSITGSENSSYQAGGSSAGGPSGSTTEAAGSTEGGSGASVNQSVKGKVGATSYEGSSSYKKSHSQTSESGQSSFKTEKKAGSS

>BnaA08T0204100ZS

MARVHLLLCFTLVFASVTLLDVTSAFLKLKPSLPQIEDPKTVGDVEGYTVNVVMVFVGDLEKECPKTSKFKMFFDKLRGFAKYVCPLKIFGKKDDTDMKAKEAGILKSIASFAIGRIKSEIQEEKQEAIETFKFMKSLAGRILGGRKKEEKETTTLTPEQLKEIKDGILKWQTVIVKITNTMVVSTTNNEGSAGSNPGAGTPSMDTNNQSQGTPSTDDTNNQSQGTPSTDDTNNQSQGTPSTDKNNQSQGTTGGSSSPNSGSATGSPSNKPSAGSNPGAGTPSTDTNNQSQGNTNTASSRSETTSQTTEVTVTEVETQTSEQVMTFLMNLEKKCPPKEEYKQFFEKLKSTMAGSAKVSSPKKKGGLFGMIKGAVGKIGDAMQFIRSRIGNKSAEVKKSMETYQAEVIKNMEELNAIYAKIVTQNQSKKGGAMTCTPEQQAEIKTTITKWEQVTTQFVEVAIKSETSTTTSTSSSTGTAQPN

>BnaA09T0042400ZS

MANKNVLALCLMFILVSSVIYEAQGTFLLKMYLRRKFFKKAMQFTPFACKGMTFLLHRLKGGCPATKGFKTFFSLFISYVNFIKTARVSKTTDSQLTTKADGLAKAVSVLTGARKDVSNNFRETILSMGKTLIEQKKAGPQRVTYKQRKVLIVALVQWTKTVVTVVKTAVETAGKTIDTSNLGLDVDVNDLLGGEKDDSPTPTTPTTPATPATPATPTTPTTPATPATPTTPTTPATPATPTPSTPTTPTPTTPTPTTPTPSTPTTPTPSTPTTPTPTTPTTPTPSTPRTSTTPTPSTPTTPTPSTPTTPATPTPSTPTTPTPTTPSTPTTPTGTTTNNAATNTNSQTSQSTTSTASAKQVETQTSQEVMSFISDLEKKYASKTELNTFFETLKSTMTATSKIASTDEKTFVSGVKAAAGTLNEAAETVTEKLGTSAESKQKIESSQQQLMKTFKELEDVKTKIVSESKGKTVSSTQQTELKQTLTKWEQVTTQFVETAASSSSTSSSTQSQQIQQSHQSQQSQQSQKTQQTQQTQQIQQGSILRAQTQT

>BnaA09T0409600ZS

MARVHLLLCFTLLFASVTLFDAASAFLKLKPSLPQIEDPKTVGDVEGYTVQVVMVFVGDLEKECPKTSKFKMFFDKLRGFAKYVCPLKTSGKKDDDDMKAKEAGILKTIASFAIGRIKREIQEEKQEAIETFKFMKSLAGRILGGRKKEEKATTTLTPEQLKEIKDGILKWQTVIVKITNTMVVSTTNTEGSAGSNPGAGTPSTDTNNESQGTPSADKNNKSQGTPSTDTNNESQGTTGGSSSPNSGSATGSPSNKPSAGSNPGAGTPSTDTNNQSQGTKNTASSGSATTSQTTEVTVTEVETQTSEQVMTFLMNLEKKCPPKEEYKQFFEKLKSTMAGSAKVASPKKKGGLFSMIKGAVGKIGDAMQFIRSRIGNKSAEVKKSMETYQTEVIKNMEELNAIYAKIVSQNQSKKGGAMTCTPEQQAEIKTTITKWEQVTTQFVEVAIKSETSTTTSTSTSTSDSRGTAQAN

>BnaC02T0470800ZS

MKKVSLLCLCCITILATSLSFNVVSAQGIAVNAPNTIKDIESYISNRAIGFVLKLEDECPIREKLRSFFEKLKDLLKLESSVTPMIEENEPKTFMSHMKSKADNLLQTMLIIGRGLLSSSVRKEMFKVVKSLTELHAAIGKVIMEKHIKGDGSMSLSLEQKNAVENAVSQWEVTITRIVKIVVEVKSKSSSAALGKESSTTDQNNTSVNETMLESNKGNFESMQENADDVSESNEDSKNDVAMKAKEGNRVEDVKGDVNNEKQLEVQGGNIDSNTNTSLEAKEDINSEVEANTDGSSITKNLEEAKGNGGVLTNDNNLENKGSEVTINDGDHMKANGEETQENNGELVMNENLENNKDNKDVKDNGLVETKKNHETSMEENRKHTQGGTEVSMNGETMHTKNGNVDSNKEKEVEVQGESIGNSTKDVNLENKEDFKGDANSEHKLEVQGGSIGDSQINKNLDTKEDVKSEVDARNDGSFMNEKSEAQGNDEVSTKNTNLENKGSEATSNDEYHTKENGKNTQEKNGELVNDENLENSKDNKEVKDNGLVEANTDHENSTEEKREHIQGGTEVSMNGKMMHTKSGNADSNKENEVEVQGESIGDTTKDVNLENKEDLKRDANSEKTLEVQEGSIGDSHINKNLDTKENVKSEVDARNDGSSMTELEDTKGNGQLFTNDTTLENKESEARTYDEDHTKENGEDTQENNGELVKDANLENNKDNKEVKDDGFVETKTNHENSTEEKREHTQRGTEVSMNGEVMPTKGGHVDSNKESVVKFQGEIIGDSTKDKNLENKDVKSEVETNKNDESSMKEKQGYSHGNDRVSTKDQNLESVGAHEETMNHKTVDAMKNNGDHLKKKREENQQNNKESTKDENSENKEDKKEFKDNGLVETKTSNENSVKENKEEAQGSNEVSMDGQMDSLGANVDSNNKTAVEFKKRHVEDSNKDSKPKVEAIKNEGNSMEGKGEVAEGNDGVSIEDTNLENIKDEKELKNNIPAETKTNKETTMEEKKEKDHVDNDESTYGGNKNESKDDKLDEIKKNKKEHMDKKWREAKGNEKNIMRGHPKEVSIDNKGYKKEIKDGKPLEVKENKENKEETQRSSSGQYTNVDNKEGKDDKSVEVKENKESSKNKIQEKMQGSGRDTTKVDRKEPIEARLENTKKDRSSVDNMDIELQKGSEESMKEEERKEHEKNKSTKIEEEKKKEKEHEEHKSKKKEENKRKEHEKNKLKKNEDEKKKQKEHENTNPKRKKKRKGKNTKKTNPREMKKKKRNRKSTKNTYPKRRKKKKGKSTKKKN

>BnaC02T0471000ZS

MKYAWPVSSTFEATDSRSYMSEMTNMATKVSDAMAVLQAKKLGSGLMKTTLQGYQQEVMKTLTILQSVLTKAVSGQQSQNPGSLTLTLSQQQAIKEITLKWEQVMSQFVRVATESEKQFSMETSTGNGFHMKKSFSSSSSSSSSSSPGFDFNLNSESPEMVDVNS

>BnaC02T0471100ZS

MTRFPLAICLMCIIVASSTVYEAQGGFLLRHYLRKLPRLANEFEPFAFKIMIRFIDNLESLCSSKVEYKEFFSKLKAFLIFINSTAGKSSSSEFESHLKAHSEGLFKAITALGVKASADTSKLIESLMSMGKVMAEYKRSGSLTMTSEQRRVLITSMMKWAQVIGQFVKTVREKTGDGDIDLPSLGIGGGDDDAGNAGGGSSGSGSSSMGTGSAGGGSSGGGASSLGVGSAGGGSSGGGTSSMGAGSAGDGSSGSGSPYPGAGGTGGGSSGSGSSSIGAGNTGMGDGSTGGSGSPSSDSGTPSSETGSPAGGGGGSPGGSPTDSSTAAGGSSMGGGGSSGDTGSTTSAAGGSSGDTGSTTGAAGGSSGDTGSTTGAGGGSSGDTGSTTGAEGGSSSDTGSTTGAAGGSSGDTGSTTGAAGGSSGDTGSTTGAGGGSSGDTGSTTGAGGGSSGDTGSTTGAEGGSSGDTGSTTGAAGGSSGDTGSTMGAAGGSSGDTGSTTGAGGGSSGDTGSTTGAAGGSSGDTGSTTGAGGGSSGDTGSTTGAGGGSSGDTGSTTGAAGGSSGDTRSTTGAEGGNSGDTGSTTGVGGGSSGDTGSTTGAASGSSGDTGSTTGTEGGSSGDTGSTTGAGGGSSVDTQSMTGAGSGSGGGGTSAGGASGSPTDDSSGSGESAGGETSGAGGSSGNTSNMSTEESSSMGGGNSSEDSKIATGGSASAESTSGGASGGSTKTTAAGESSMNNGDSYSDSTGGNTMGSPTGSPSGSGGSSFTGSETGSSSYQVGGASAGGPSGSTTDSSAAGASSKNSGYSAKGSSTTSAQGSTQDGSGLAEGASEGKSFKGKTGSTRYEGSSNYQKTHSKSSDKSSFSHSSEEKNSGNV

>BnaC02T0472000ZS

MANKNVLGLYLMFLVVSSVVYEAQGTFLLKMYLKRKFLRNTRNFTPFACKGMLLLMSRLKRGCPATEGFKTFFSLFTSYVKFIKTAPTTSSTDTQLTTKVDALTNAISVLTGTKSGSNSNFRETMLSMGKTLIEQKRSTSQRMTHQQKKVLVAAMVQWTKTVATLVKTAVETAGKSIDVSNLGLDVDVNSIVGSGSDESPESGSPGSDSPTTTSSGSNTDSGSIIYEDTTGGGSSSPSGSPADSPSSTMTTQSAGSTPGSQSGTTRESPNDTTTESPSGTTTESTSGTTTDSPSGSPTDSPSGTTTESSSGSPTDSPSGATTESPSDTTTDSQSDTTTGSPSGSPTNSPSGATTDSPRGATTDSQSDTTTESPTDTTTGSPSESPTDSPSGATTDSPSGATTDSPSGATTDSPSGATTDSQSDTTTESPTDTTTGSPSGSPTNSPSGATTDSPSGVTTESQSDTNTESPTDTTTGSPSGSPTDSPSGATTDSPSGATTDSPSDTTTGSPSGSPTDSPSGATTDSPSGSPTGSPSGTTTDSSSGSPTDSPSGSPTDSGSGSPTDRPSGSPMDSESSQSSTSSLSTTSAKEVEIQTSKEARSFIHALEKKYAGTVQLDTFFEKLKTSMSASTKISNTDEKRFVSKMSSAVSSVSEAATTVSSKLAKSPEAKSRMESSKEKLMKTYKELEDVNSKIVSENKGKTVSSTQKSELKQILSKWEQVTTQFVENLVSSSSSSSSSSSSSQSQQSQQSQKSHQSQQSQQGSTMKTETN

>BnaC03T0665700ZS

MARVHLLLCFTLLFASVTLLDVASTFLKLKPSLPQIKDPKTVGEVEGYTYVCPLKIFGKKDDADMKAKEAGILKTIASFAIGKSEIQEEKQEAIETFKFMKSLAGRILGGRKKEEKETTTLTPEQLKEIKDGILKWQTVIVKVANTMVVSTTTNNEGSAGSNPGDGTPSTDTNNQSQGTPSTDTNNQSQGTPSTDTNNQSQGAPSTDTNNQSQGTTEGSSSSNSGSATGSPSNKPSAGSNPGAGTPSTDTNNQSQGTTNTASSRSATTSQTTEVTVTEVETQTSEQVMTFLMNLEKKCPPKEEYKQFFEKLKSTMTGSAKVSSPKKKGGLFGMIKGAVEKIGDAMQFIRSRIGNKSAEVKKSMETYQAEVIKNMEELNAIYAKIVTQNQSNKGGAMTCTPEQQAEIKTTITKWEQVTTQFVEVAIKSETSTATSTSTSSSTGTAQAN

>BnaC04T0395000ZS

MSRFQNLLCFTILLATVTFFNVASAHVKIKLALPQTGDPISVGDVEPYTVKIVSTFVADLEKECAKTEKFRHFFEKVNAFSKCVCSVSMDHESHMKAKAGSLFQAISALGSDENGSKGGMVNKLQKEKTEAMETVKMLQSIGEKITGRQNKKTEINGTLKLTTKQQKEIKDGILKWVKVITQIAKQADEISMASETKKESKEENSVGPRILVQTKSSTKSDKNSDKKSDNKKNNNGESKKKKRGGKEGRHGSAKSKGGSNDDKSSKNKRKSSENADKNHQTKYGKVEKKKGGVEQSRNKTAERLRAEASDELD

>BnaC05T0263000ZS

MARVHLLLCFTLLFASVTLFDAASAFVKLKPSLPQIEDPKTVDDVEGYTVQVVMVFVGDLEKECPKTSKFKMFFDKLRGFAKYVCPLKIFGKKDDADMKAKEAGILKTIASFAIGRIKREIQEEKQEAIETFKFMKSLAGRILGGRKKEEKATTTLTPEQLKEIKDGILKWQTVIVKITNTMVVSTTNSEGSAGSNPGVGIPSTDTDNQSQGTPSADTNNESQGTTGGSSSPNSGSATGSPSNKPSAGSNPGAGTPSTDTNNQSQGTPSADTNNESQGTTGSSSSPNSGSATGSPSNKPSAGSNPGAGTPSTDTNNQSQGTTNTASSGSATTSQTTEVTVTEVETQTSEQVMTFLMNLEKKCPPKEEYKQFFEKLKSTMTGSAKVSSPKKKGGLFGMIKGAVGKIGDAMQFIRSRIGNKSAEVKKSMETYQAEVIKNMEELNAIYAKIVSQNQSKKGGAMTCTPEQQAEIKTTITKWEQVTTQFVEVAIKSETSTTTSTSTSASTGTAQAN

>BnaC07T0315800ZS

MAKFPLGICLMFMITTSTIYEVQGHFLLDHYMKKIPKISSEFEPFAFKGILSFIDHLEGLCPLKVEYKEFFTKLKDFMAFINSASGSSAEFHTQLKTKSEELFKAITKMGGTAGASAHTTKLIESLMSMGKTLAEYKRSGSQTLTSEQRTELVTSMSRWAQTIGQFVKSVTETNGGANIDLKSLGCGGATGISTSMETGSTASGSTNMETGSTGGSGSPSGDTGSPASGGSESGSPSNETPAEGGSGSPSGSPSDSSSGAGSTGSETSTEAETTAGSGSGAGAGETPGAAGPSGSPTGSPTESEESSKDESSKDESSKDESSKDESSKDESSKDKSSKGEKDESSKDESSKDESSKDESSKDESSKDESSKDKSSKDDKDESSKDESSKDESSKDESSKDESSKGESSAESGESSKEASGASSTETQSETGAESGSTDGGASAGGPSGEPPGDAGAGAPSGSTSDMSAGGASASGGASGTSTQTSAEGESSMNSGGSSYADTTGGSAAEGSASSPSGSASGSSETSSITGSENSSYQAGGSSAGGPSGSTTAGSAAGESTTEESSEGGSGASENQSVKGKKGTANYEGSSSYKKSQSQSSESAKSSFKHSSEKNAGSS

>BnaC07T0315900ZS

MARISLALCLMLVVTSSVIYEARGHFLLKDYLTTKFPSKSSEFTPYVNTGLTEFLTDLERFCPPTPEFKSFFTEFKSFFSSIETSSSTSQNIDMEKKGDGLFKAVSAITGGAGQKSAEAGSFKSTMISMAKTLVEQKKSTTAITSTEKKTLVTSMVQWTKTIATTVKTACEKKGKKIDINSFGLNVDVNSVMTVSESRQSSSSSSKSSSESSSKSSSYAARAETAASAKAKETTGARAEKGEKKGEKKAEKKGEKKAEKKAEKNEQKAATSSAKTKEATSTKAGPSASTKTGTTFRDTTGGYAGSPRGSPTAAKDKTAGSPRGSPTAAKEKTSVKGTGKAAASVNQQSNAGSAAASTNQQQSNAASSRGSAASTNQESASSKSSSTTSVTEIEKETSQETTSFISGLEKRFAQKAELKPFFEKLKASMTASSRVSSTKSEQEYTNTAKSTTGKLSDAMSFVGSRFSKSAEMKSNIQTTQQQLIKNLQQFQNLNSQIVGEQKVSSTKETEIKKTMSKIEQVTTQFVETAASSSSSSSKQETAASQQSQKKTMASSKKETAASSQQQTQQQTQQQTQQENGMGRLKTN

>BnaC07T0319600ZS

MEHVSSATTATTTSSTTVAELEAETSKEVMEFIMKLEKKCPPKEEYKSFFEKLKATMVASAKVTQEKKKGFFSAAAGKISDAVSFIGSKFTGKSAEVKKSMETYQQEVAKSLQELEAIHKKIIEANQGKVEGSVAVTAEQKTEIKQTITRWETVTTQFVETAIQTEAASNTTVGVDKVKLP

>BnaC09T0027800ZS

MANKNVLALCLMFILVSSVIYEAQGTFLLKMYLRRKFFKKAMQFTPFACKGMTFLLNRLKGGCPATKGFKTFFSLFISYVNFIKTATVSKTTDSQLTTKADGLANAVSVLTGARKDVSKDFRETILSMGKTLIEQKKAGTQPVTYKQRKVLIVALVQWTKTVVTVVKTAVETAGKTIDTSNLGLDVDVNDVIGGEKDDDSPTPTTPTTPATPTTPTTPSTPTTPTTPSTPTTPTTPTTPTTPTTPTPSTPTTPTPTTPTTPTPSTPTTPTPSTPTTPTTPTPSTPRTSTTPTPNTPTTPTPSTPTTPTTPTPSTPTSPTTRTPTTPSTPTTPSTPTGTTTNNAATNTNSQTSQSTTSTASAKQVETQTSQEVMSFISDLEKKYASKTELNTFFETLKSTMTATSKIASTDEKTFVSGVKAAAGTLSEAAETVTEKLGTSAESKQKIESSQQQLMKTFKELEDVKTKIVSESKGKTVSSTQQTELKQTLTKWEQVTTQFVETAASSSSSSSSTQSQQIQQSHQSQQSQQSQKTQQTQQTQQIQQGSILRAQTQT

>Bnascaffold0027T0035400ZS

MAKTSLTIYLSLLVALSTVYETQGTFSLPLYLKNFPKVGHDFESFAYKGMMDFMGDLEGKCPQTTEFKDFFLKLKDYMACYSSTAPGSKDLQVELSIKSETLFRAMSDFSGTKGGTSTRPVYAFGLEDSWTLVDGLLSMGKSLVEMKKSGSKEITFEQRKETIQSMVKWTRGIGLFVKKVSESKGKSIDLSSFGIDYDNNVSSPSERALYETQGTFSLPHYVKDFPKMSKDFEPFAYKGMSGFLGALESKCPATAEFKDLFVTVADYMACFKSGIKVEMQEKSVKLFRAISVLDGTNGGTSVDSWRMVDGMLSMGILVTEMKKNVSQEITFEQRKELIGGMVKWARAIGLLVKTASEKKGKSIDLASFGLIIAPMLLLLSKELAVSYKDKPCSFYFNSNNSTTFDGS

>BniB01g027510

MSRFQHLLCFTIILATVTFFNVASAHVKIKPALPQTGDPIIIGDIEPYTVKVVSTFVADLEKECAKTEKFRYFFEKINAFSKYVCSVSKGKDHESHMKAKAGSLFQAIAALGTDESVSKGGEVNKLQKEKTEAMETVKMLQSIGEKITGGRNNKTEINEILQLTKKQQKEIKDGILKWLNVISLIAEKADEISMKSSSKSETKEEKSVEPRKLVATKSSTKSNKNSDKESDNKKKESDNKKNKKGESKKKKHGGKEGRHGSAKSKGGSDDDKSSKNKRKSSENADKNHRTKYGTVETKKGGVEQSRNKTAERLRAEAADELD

>BniB01g027540

MSRIQNLLCFTILLATVTFFNVASAHVKIKFALPQTGGPISVGDVEPYTVKVVSTFVADLEKECAKTEKFRHFFENVNAFSKCVCSVSKVEDHESHMKAKAGSLFQAISALGSDENGSKGGKVNKLQKEKTEAMETVKILQSIGEKITGGKNNKTEINGTLKVTTKQQKEIKDGILKWLDVITRIAKKADEISMESSSKSVGPRILVETKSSTKSNENSDKGGDNKKKESDNKKKESNNKKNKKGESKKKKNGGKEGRHGSAKSKGGSGDDKSSKNKRKGSKNADKNNQTKYGKVETNKGGVEQPRNKTAERLRAEAADELD

>BniB02g068390

MAKFPLGICLMFMITTSTIYEVQGHFLLDHYLKKIPTISSEFEPFASKGILSFIDHLEGLSPPKEEYKEFFTKLKEFMAFINSASGSSSEFHSQLKTKSEELFKAITKMGGKAGSSAHTSKLIESLVSMGKTFAEYKRSGSQTLTSEQRTELVTSMSKWAQVIGQFVKTATEKSGDGNIDLKSLGCGGGTSGSISSSSTETGSSASGSSSMENALSTGGSGSPSEETGSPSAETGSPASGDGPGDSGSPSDSGSPSNESPAEGGSSSPIGSPSDSSSGPGSTGVESPAEAGSAAGAGSGTGDGETPGAAGPSGSPTEPGESSKDESSKDESSKDEKATESGESSKDASGANSNEESSTETQSETGAESGSTDGGASAGGPSGSPTESSGPGDVGAGGPSGETSGEAGAGGETSGEAGAGGETSGDAGAGGPSGETSGDAGAGGPSGETSGDAGAGGPSGETSGDAGAGGPSGSTSDMSAGGASTSGGASGTSTQTSAEAESTMNSGGSFADTTGGSSGMEGPASSPSGSALGGSSETSSFSEMSSHEAGGSSAGAPSGSTNDGSAEGETSTKGKMSYERSASYRKSHSQTSGHSSFKHSSEEKNTGSS

>BniB02g068400

MARISLGLCLMLVVASSVIYEAQGHFLLKNYLKTQFPSKGNEFTPYVNRGLTELLTDLERFCPPTPEFKGFFTEFKSYMSFIETSSSSSSKNIDMEMTKKADGLFRAMSAISGGAGQKSAEAGSFKSTMISMGKTLVEQKRSTTTITSTEKKTLVTSMVQWTKTIATVVKTASEKKGKKIDINSFGLDVDVKSIVSVSGSSHSSSSSTKTSSESSSKSSSYNARAETGSSAKAKETTEKGEKKDNAEKKPEKKEEKKPEKKEEKKPEKKEEKDSSSAKTKETSSTKAGASGSTKTGSSFKDTTGASAGSPRGSPTAAKDKTSGSPTSDKGRTSEKESGNAGSVKQQSNNAASRGSASSMNQQQSKKSESASSSSTTSVTEIEKETSQETSSFISGLEKRFSQKAELRPFFEKLKASMTASSRVSSTKSAQEFTSTAKSTTGKLSDAMTFVGSRFSKSAEMKSNIETTQEKLMKSLKEFQDLNSRIVGEQKVSTTKETQIKQTMSKIEQVTTQFVETAASSSSQQSQQSQKQRTATSQQQTAASSQQQTAASSQQQTAASSQQQNEQQNGTFLLRHFLRKIPRRSRGLRPFACKGMLKFVNLLEFKSPVKPVYKNLFGNLRSYVGAISSGSSADLKGKAQGVQNALTALGGSSGSSVDTSKVMDVLMTMGKTLTSQTSSSSTEITSVQRKELLTSLVQFARVISQVVVSAASKSGSSIDIKSLGIDGVDANVATGGSTSTSATGSSNTKTGTSTSGNIMTGHGQGYSHTARHHTPARTAGTTARMHCQGHCYGKPRTAAGTATGTVPVPATGTVPGTGMAGGPASSTEGGSSFGRSFSGKTGSTNYAGSLNYQSGHKSSQQTTTSNAGSPGSTVSPGSF

>BniB02g068740

MARVQLLLCFTFLFASVTFMEHVSSATTASSSKSTTVSELEVETSKEVMEFIMKLEKKCPPKEEYKSFFEKLKSTMVGSTKVTLEKKKGFFSAAAGKISDAMSFIGSKLSGKSAEVKKSMENYQQEVTKSLQELETLHKKIIEANQGKADGSATVTAEQKTEIKQTITRWETVTTQFVETAIQTENASNTTVGVDKVKLP

>BniB03g016080

MARVHLLLCFTVLFASVTLFDVASAFLKLKPSLPQIEDPKTVGDVEGYTVKVVMVFVGDLEKECPKTSKFKMFFDKLRGFAKYVCPIKIFGKKDDDDMKAKEAGILKTIASFAIGRIKSEIQEEKQEAIETFKFMKSLAGRILGGRKKEEKETTTLTPEQLKEIKDGILKWQTVIVKITNTMVVSTTTNKEGSDSTTTTTPTGGSSSPNSGSSAGSPSNKPSAGTNSGAGTPSKDTNNQPEDTKNSASSGTGSTSQSTKEVTVTEVETQTSEQVMTFLMNLEKKCPPKEEYKQFFEKLKSTMTGSPKVSSPKKKGGLFTMIKGAVGKIGDAMQFIRSRIGNKSAEVKKSMENYQTEVIKNMEELDAIYTKIVSQNQSKKGGALTCTPEQQAEIKQTITKWEQVTTQFVEVAIKSETTTTSTSSSSSSSTEKAQAN

>BniB04g006810

MKMARFPLAICLMCIIVASSTVYEAQGSFLLRHYLRKLPRMANEFEPFAFKIMLRFIDNLESLCSSKVEYKEFFSKLKAFLIFINSAAGKSSSSEFESQLKAHSEGLFKAITALGVKSSADTSKLIESLMSMGKVMAEYKRSGSLTMTSEQRRVLITSVMKWAQVIGQFVKTVREKTGEGDIDLPSLGIGGVGDDDAGSSGSGSSSMGDGSAGGGSSGGGASSLGAGSAGGGSSGSGTSSMGAGSAGDGSSGSGSPYPGAGGGSSGSGTSSMGGGNTGMGDGSTGGSGSPSSDSGTPSSETGNPADGGSGSPGGSPTDSSTAAGGSSMGGGGSSGATTGAAGGSSGDTGSTTGAGGGSNGDTGSTTGAVGGSSGATGSTTGAAGGSSGDTGSTTGAGGGSSNGGGSVDTQSMTGAGSGSGSGGTSAGGPSGIPTDDGSGSGESAGGETSGAGAGAGAGAGGSSGNTSNMSSKDSKITTGGSASEESTSGGASGGSTDTTATGESSMNNRDSYSDSTGGKTMGSPTGSPSGSTSGSGGSSFTGSETGTSSYQAGGASAGGPSGSITDSSAAGASSMNSGSSKTQTSAQGSTQGGSDLAEGASEGKSFKGKTGSMGYEGSSNYQKAHSKSSDKSSFSHSSEEKSSGNV

>BniB04g006820

QAETPERSEESEKEMQAETPTKSEESEEEMQEEAPARSEESEKEIQAEAPARSEESEKEMQAEAPTKSEESEEEIQAEAPTRSEESGNKIQAETPERSEESEKEMQAETPTKSEESEEEMQEEAPARSEESEKEIQAEAPARSEESGKEMQAETPTRSEESEKEMQAETPAKSDESDKEMQAKVPAMSDESEKEMQTEAPTKSEESKKEMQAEAPARIEKSDKEIQAEVPGRSDESEKEMQAETPIKNDELEKEMQAEAPSKSGESNKEMQANAHTESEESEKEMQAEAQARSEKFEKEIQAEAPARSKESEKELQTKTHIESEESAKKMQAETPTKSEELEKEMQAKAPAKSGETDKEMQANAHTESEESEKEMQAEAQARSEKFDKEIQAEAHTKSAESEKKKQGEAQAGNEDSSMKKHEEIQETNVGDKKDQSVSKEKDSTDSKTTEIKEGSEEDSKNGETVENNGNIAEINKDKEDYMKEDSENSEGAEIDGGKEVPVEEGSKDGQTLENNSSTENSMEEGSKDSKAVEVREGQDNSMEKSNESQEVTMEEGSKDGNTTKLNGSKDNSMEEGTVETSGVKEDSMKDKATEVQGNNNSSTSDTSMATEVQGHNNSSTSDTTTDVKGGSININEDSTKNKTMEAQGGSTGGSTNGTTQETTESNDSMNNQNMQHVEGDNIDVNEDSTKNKTMEAQSGSAGGSTNSTTQETTESNDSMSNQNVQHGGNNANSMNNQTSESSTTEQKVTTDIESSTSKEVSSFISNLENKSPGTQAFQSFFQKLKDYMKYAWPVSSTFEATDLRSYMSEMTNMATKLSDAMSVLQARKLGSGLMKTTLQGYQQEVMKTLTLLQSVLSKAMSGQQSQNSSSLTLTLSQQQAIKEITSKWEHVMSQFVRVATESEKQFSMETSTGNGFHTKKSSSSTSSSSSSSSSSSSPGLDFNLLNGESPGMVDVNN

>BniB04g006830

MKKVSLLCLCCITILATSLSLNVVSAHETAVNAPNTIKDIEPYISNRAIGFVLKLENDCPIREKLRSFFEKLKDLLKLESSVTPMIEDNEPKTFMSHMKSKADDLLQTMLMIGRGLLSSSVRKEMFKVIKSLTELHAAIGKVIMEKHIKGDGSMSLSLEQKNAVENAVSQWEVTITRIVKIVVEVKSESSSTVSGEESSTAEQNSTSVNETMLESNKGNLESMQENGNGINESNEDSKNNVAIEAKEDNRVEDVKGDANNEKQLEVQGGNIDTSLNTKEDVNSEVDAKPDGSSMTKNSEEAQGNDGVSIKDHNLENIGSEAGINDGDHTKDNGEETQENNGELVMNENSENNKDNKEVKNDGLVQTKTNHEISMKEKREHTQERSEVSMNGETMHSKSGGVQGESIGDSTKDVNLENKENLKGDANSKKPLEVQGGSIGDSHIDKNLDTKEDVKSEVDARKEMLSNSEEKSEESEKEMQVKTPTRSEESHKKIKAEVPATSDESEKEMQAETLTKNEELEKKMQAEAPTKSGESDKEMQAKAPSRSDESEKEMQAETPTKSEESEKEMQAEAPTRNEESGNEIQAEAPSRSDESEKEKQAETPTKSEESEKDMQAEAPARSEESEKKIQAEAPTRSEESEKEIQAETPTRSDKSEKEMQAETPAKSEESDKEMQAKAPAMSDESEKEMQAX

>BniB04g045920

MARVHLLLCFAVLFASVTLFDVASAFLKLKPSLPKIEDPKTVGEVEEYTVKVVMVFVGDLEKECPKTSKFKTFFDKLRGFAKYVCPLKIFGKKDDADMKAKEAGILKTIASFAIGRIKSEIQEEKQEAIETFKFMKSLAGRILGGRKKEEKETTTLTPEMVKEIKDGILKWQTVIVKITNTMVVSSTKNNEGSDSTTTTPTTGGDSSSPNSGTSTGGPSNKPSAGSNPGAGTPSKDTNNQPEATTNTASSGSETTSQTKEVAVTEIETQTSEQVMTFLMNLEKKCPPKEEYKQFFEKLKSTMTGSAKASSPKKKGGLFAMIKGAVGKIGDAMQFIRSRIGNKSAEVKKSMETYQSEVIKNMEQLDAIYAKIITQNQSKKGGAMTCTPEQQAEIKQTITKWEQVTTQFVEVAIKSETSTSTSTSTSSSTGTAQAN

>BniB07g036410

MANKNVLALCLLFLVTSSVIYEAQGTFLLKWYLRRKMFKKAMEFTPFACKGMLFLLNRLKEGCPATKGFKTFFSLFTSYVNFIKTAKVSKTTDTELTTKADGLAKATSVLTGAKTVSTDFRETLLSMGKTLIEQKKSGAEPVSRKQRKVLVVALVQWTKTVVTIVQTAVQTAGKTIDTSNLGLDVDVNDVVGDDSDDDDDDSPKKGTPSGSDSASPSSGTPTPTTPTTPTTPTTPSTPTSSTPTPTTPTTPTTPSTPTSSTPTPTTPTTPTTPSTPTGSTPTPTTPSTPTTSTPTPTTPTTPSTPTGSTPTPTTPTTPSTPTRSTPTPSTPTSSTPTPTTPSTPTTPTTSTGGETTSTGSTTNTGSTNTKGAGSTKTTQEATETDTGATKGSPSGTPTDSPSGETPTNTNSQTSESTSSTTSAKQVETQTSQEVMSFISGLEKKYAGKAELNTFFEKLKSTMSATSKIATTDEKTFVSGVKSAAGKLSEAAETVTEKVGKSAESKQKIESSQEQLMKTFKELEDVKTKIVSESKGKTVSSTQQTELKQTLTKWEQVTTQFVETAASSSSASSSSQSSLSQKSQQSQQSHQSHQSQHTQQTQQTQQGSTMKAQTQTN

>Bol012128

MSRFQNLLCFTILLATVTFFNVASAHVKIKLALPQTGDPISVGDVEPYTVKIVSTFVADLEKECAKTEKFRHFFEKVNAFSKCVCSVSMDHESHMKAKAGSLFQAISALGSDENGSKGGMVNKLQKEKTEAMETVKMLQSIGEKITGRQNKKTEINGTLKLTTKQQKEIKDGILKWVKVITQIAKQADEISMASETKKESKEENSVGPRILVQTKSSTKSDKNSDKKSDNKKNNNGESKKKKRGGKEGRHGSAKSKGGSNDDKSSKNKRKSSENADKNHQTKYGKVEKKKGGVEQSRNKTAERLRAEASDELD

>Bol016109

MARVHLLLCFTLLFASVTLLDVASAFLKLKPSLPQIKDPKTVGEVGFAKYVCPLKIFGKKDNADMKAKEAGILKTIASFAIGRIKSEIQEEKQEAIETFKFMKSLAGRILGGRKKEEKETTTLTPEQLKEIKDGILKWQTVIVKVANTMVVSTTTNNEGSAGSNPGAGTPSTDTNNQSQGTPSTDTNNQSQGAPSTDTNNQSQGTTEGSSSPNSGSATGSPSNKPSAGSNPGAGTLSTDTNNQSQGTTNTASSRSATTSQTTEVTVTEVETQTSEQVMTFLMNLEKKCPPKEEYKQFFEKLKSTMAGSAKVSSPKKKGGLFAMIKGAVGKIGDAMQFIRSRIGNKSAEVKKSMETYQAEVIKNMEELNAIYAKIVTQNQSKKGGAMTCTPEQQAEIKTTITKWEQVTTQFVKVAIKSETSTATSTSTSSSTGTAQAN

>Bol032259

MANKNVLALCLMFILVSSVIYEAQGTFLLKMYLRRKFFKKAMQFTPFACKGMTFLLNRLKGGCPATKGFKTFFSLFISYVNFIKTATVSKTTDSQLTTKADGLANAVSVLTGARKDVSKDFRETILSMGKTLIEQKKAGTQPVTYKQRKVLIVALVQWTKTVVTVVKTAVETAGKTIDTSNLGLDVDVNDVIGGEKDDDSPTPTTPTTPATPTTPSTPSTPTTPSTPTTPTTPTPSTPTTPTTPTPSTPTTPTPTTPTTPTPSTPTTPTPSTPTTPTTPTPSTPRTSTTPTPNTPTTPTPSTPTTPTTPTPSTPTSSTTPTPTTPSTPTTPTGTTTNNAATNTNSQTSQSTTSTASAKQVETQTSQEVMSFISDLEKKYASKTELNTFFETLKSTMTATSKIASTDEKTFVSGVKAAAGTLSEAAETVTEKLGTSAESKQKIESSQQQLMKTFKELEDVKTKIVSESKGKTVSSTQQTELKQTLTKWEQVTTQFVETAASSSSSSSSTQSQQIQQSHQSQQSQQSQKTQQTQQTQQIQQGSILRAQTQT

>Bol022560

MNLEKKCPPKEEYKQFFEKLKSTMTGSAKVSSPKKKGGLFGMIKGAVGKIGDAMQFIRSRIGNKSAEVKKSMETYQAEVIKNMEELNAIYAKIVSQNQSKKGGAMTCTPEQQAEIKTTITKWEQVTTQFVEVAIKSETSTTTSTSTSASTGTAQAN

>Bol022559

MARVHLLLCFTLLFASVTLFDAASAFVKLKPSLPQIEDPKTVGDVEGYTVQVVMVFVGDLEKECPKTSKFKMFFDKLRGFAKYVCPLKIFGKKDDADMKAKEAGILKTIASFAIGRIKREIQEEKQEAIETFKFMKSLAGRILGGRKKEEKATTTLTPEQLKEIKDGILKWQTVIVKITNTMVVSTTNSEGSAGSNPGAGIPSTDTDNQSQGTPSADTNNESQGTTGGSSSPNSGSATGSP

>Bol033204

MKKVYLLCLCCITILATILSFNVVSAQGIAVNAPNTIKDIESYISNRAIGFVLKLEDECPIREKLRSFFEKLKDLLKLESSVTPMIEENEPKTFMSHMKSKADNLLQTMLIIGRGLLSSSVRKEMFKVVKSLTELHAAIGKVIMEKHIKGDGSMSLSLEQKNAVENAVSQWEVTITRIVKIVVEVKSKSSSAALGEESSTTDQNNTSVNETMLESNKGNFESMQENADDVSESNEDSKNDVAMKAKEGNRVEDVKGDVNNEKQLEVQGGNIDSNTNTSLEAKEDINSEVEANTDGSSITKNLEEAKGNGGVLTNDNNLENKGSEVTINDGDHMKANGEQTQENNGELVMNENLENNKDNKDVKDDGLVETKKNHETSMEENRKHTQGGTEVSMNGETMHTKNGNVDSNKEKEVEVRGESIGNSTKDVNLENKEDFKGDANSEHKLEVQGGSIGDSQINKNLDTKEDVKSEVDARNDGSFMNEKSEAQGNDEVSTKNTNLENKGSEATSNDEYHTKENGKNTQEKNGELVNDENLENSKDNKEVKDNGLVEANTDHENSTEEKREHIQGGTEVSMNGKMMHTKSGNADSNKENEVEVQGESIGDTTKDVNFENKEDLKRDANSEKTLEVQEGSIGDSHINKNLDTKENVKSEVDARNDGSSMTELEDTKGNGQLFTNDTTLENKESEARTYDEDHTKENGEDTQENNGELVKDANLENNKDNKEVKDDGFVETKTNHENSTEEKREHTQRGTEVSMNGDVMPTKGGHVNSNKESVLKFQGEIIGDSTKDINLENKDVKSEVETNKNDESSMKEKQGYSHGNDRVSTKDQNLESVGAHEETMNHKTVDAMKNNGDHLKKKREENQQNNKESTKDENSENKEDKKEFKDNGLVETKTSNENSVKENKEEAQGSNEVSMDGQMDSLGVNVDSNNKTTVEFKKRHVEDSNKDSKPKVEAIKNEGNSMEGKGEVAEGNDGVSIEDTNLENIKDEKELKNNIPSETKTNKETTMEEKKEKDHVDNDEFTYGGNKNESKDDKLDEIKKNKKEHMDKKWREAKGNEKNIMRGHSKEVSIDNKGYKKEIKDGKPLEVKENKENKEETQRSSSGQYTNVDNKKGKDDKSVEVKENKESSKNRKQEKMQGSGRDTTKVDRKEPIEARLENTKKDRSSVDNMNIELQKGSEESMKEEERKEHEKNKSTKIEEEKKKEKEHEEHKSKKKEEDKRKEHEKNKLTKNEDEKKKQKEHEKHKSKKKEEEKRKEHEENKSKRNEEEKKKQKKKQKKHEEHISKKKEEEKMKEHEENKSKRNEEEKKKQKKHEEHISKKKEKEKRKEQERKKLKNEEDKKKQKEHEEHISKKKEEDKRKEHEKNKSKKNEEEKKKQKYHKEHISKKKEEAKRKENEKNKLKKYEEEKKRQKEHEEQISKKKEEKKRIKHERNKLKNEEEKKKQKEHEEHISKKKEEEKKKEHEKNKTKKNEEKKKMQKEHEEHISKKTEEEKRIEHKEHISKKKKEKERKEHENYKSKKNEEDKKKQKEHKEHLSKKKEEKQRIELEKNKLKKEEEKKKQKEHEEHISKKKEEEKRKEHEKNKSEKNEKEKKKQKEHGEYISRKKEKDKRIEHERNKLKNEEEKKKHKEHEEHKSKKKEEEKKNEHERNKSRKNEEEKKKQKEQEEYLSKKKDEEKRKEHERNKLRKNEEEKKKQKEHEEHISQKKEEDKRKEHEEHTSKKKEEEKRKEHEENKAKKIEEEKRKEHEKNKSKKNEDKKSEHEQYKSKKTHKENESNENEEEKMKKREYEETKSKKNEEQKKREHEKNKSNENEKEKKKEHEENKFKKEEEEKKMKRDYDNNKSKKNKEDGKEKTKENVKMSSDDKSKDHEKEMLVNAQEKSEESEKEMHAETLSRSEESDKEMQADAPTKSEESVKEMQTKTHARSEESEKKIQAEAQARSEEPEKKMQAEAQAKSKESEKETQTKDQAQNEEPEKEMQAKAHIENEESEKEMQAEAHAKSEESEKEMQAEAQAKSAEFEKRKNAKAQAENEDSSIKKQEEIQETNVGEKKDQGDLKEESSADGKTTEIKEGSIEEGSKDGETVENNGVNEEAMEEHSKDGKVVETNGGKEDSMEEGPKDEKLIKDNENTTEISGGKNSTVGDSDDGKIVENNGGKEDSLEEGEDGKPVEMHGGKNSTEEVSKDSTAEINGGKEDSIEESSKDGDIDEINSDKEESMKEGSENGKTADIDGIKEVPIEEGSKDGKTLENNNNSTENSVEEGSKDSKTVEVNEGQDKSMEKGNGSQDVTMEEASKDGNTTELNGSKDNSMEEGSTDGNTVETDGVKEDSMKDKATQVQGNNNSSTSDTSMATEVQGHNSSSTSDTSTDVKGGNIYINEDSTKNKTLEAQGGGAGDSTNGETEETAESNDSMNNQNVQHVEGDNIDINEDSTKNKTMEAQGGGAGDSTNGETEETTESNDTMNNQNVQHGENNANSINNQTSESSSTEKKVTTDIESSTSKEVTSFISNLEHKSPGTQEFQSFFQKLKDYMKYAWPVSSTFEATDSRSYMSEMTNMATKVSDAMAVLQAKKLGSGLMKTTLQGYQQEVMKTLTILQSVLSKAVSGQQSQNPGSLTLTLSQQQAIKEITLKWEQVMSQFVRVATESEKQFSMETSTGNGFHMKKSFSSSSSSSSSSSPGLDFNLNSESPEMVDVNS

>Bol033203

MTRFPLAICLMCIIVASSTVYEAQGGFLLRHYLRKLPRLANEFEPFAFKIMIRFIDNLESLCSSKVEYKEFFSKLKAFLIFINSTAGKSSSSEFESQLKAHSEGLFKAITALGVKASADTSKLIESLMSMGKVMAEYKRSGSLTMTSEQRRVLITSMMKWAQVIGQFVKTVREKTGDGDIDLPSLGIGGGDDDAGNAGGGSSGSGSSSMGAGSAGGGSSGGGASSLGAGSAGGGSSGGGTSSMGAGSAGDGSSGSGFPYPGAGGTGGGSSGSGSSSMGAGNTGMGDGSTGGSGSPSSDSGTPSSETGSPAGGGGGSPGGSPTDSSTAAGGSSMGGGGSSGDTGSTMGAAGGSSGDTGSTTGAAGGSSGDTGSTMGAAGGSSGDTGSTTGAAGGSSGDTGSTTSARGGSSGDTASTTGAGGGSSGDTGSTTGAEGGSSSDTGSTTGAAGGSSGDTGSTMGAAGGSSGDTGSTTGAGGGSSCDTGSPTGAAGGSSGDTGSTTGAGGGSSCDTGSPTGAAGGSSGDTGSTTGAEGGSSGDTGSTTGAGGGSSSDTGSTTGAAGGSSGDTRSTTGAEGGNSGDTGSTTGAASGSSGDTGSTTGTEGGSSGDTGSTTGAGGGSSNGGSSVDTQSMTGAGGGSSNGGSGGGGTSAGGASGSPTDDSSRSGESAGGETSGAGGSSGNTSNMSTEESSSMGGGNSSEDSKIATGGSASAESTSGGASGGSTKTTAAGESSMNNGDSYSDSTGGNTMGSPTGSPSGSGGSSFTGSETGSSSYQAGGASAGGPSGSTTDSSAAGASSKNSGYSAKGSSTTSAQGSTQGGSGLAEGASEGKSFKGKTGSTRYEGSSNYQKTHSKSSDKSSFSHSSEEKSSGNV

>Bol033201

MANKNVLGLYLMFLVVSSVVYEAQGTFLLKMYLKRKFLRNTRNFTPFACKGMLLLMSRLKRGCPATEGFKTFFSLFTSYVKFIKTAPTTSNTDTQLTTKVDALTNAISVLTGTKSGSNSNFRETMLSMGKTLIEQKRSTSQRMTHQQKKVLVAAMVQWTKTVATLVKTAVETAGKSIDVSNLGLDVDVNSIVGSGSDESPESGSPGSDSPTTTSSGSNTDSGSIIYEDTTGGGSSSPSGSPADSPSSTMTTQSAGSTPGSQSGTTTESPNDTTTESPSGTTTESPSGCDSPSGSPTDSPSDTTTESPSGTTTESPSDTTTGIPSGSPTDSPSGATTESPSDTTTDSPSDTTTGSPSGSPTNSPSGATTESPSDTTTGIPSGSPPDSPSGATTDSPSDTTTGSPSGSPTNSPSGATTDSPSGATTDSQSDTTTESPTDTTTGSPSGSPTDSPSGATTDSPSGATTESQSDTNTESPTDTTTGSPSGSPTDSPSGSPTDSPSGATTESPSGAATDSPSDTTTGSPSGSPTDSPSGSPTDSPSGATTDSPSGATTDSLSGTTTESPNGSPTGSPSGTTTDSPSGSPTDSPSGSPMDSESSQSSTSSSSTTSAKEVEIQTSKEARSFIHALEKKYAGTVQLDTFFEKLKTSMSASTKISNTDEKRFVSKMSSAVSAVSEAATTVSSKLAKSPEAKSRMESSKEKLMKTYKELEDVNSKIVSENKGKTVSSTQKSELKQTLSKWEQVTTQFVENLVSSSSSSSSSSSQSQQSQQSQKSHQSQQSQQGSTMKTETN

>Bol042984

MAKFPLGICLMFMITTSTIYEVQGHFLLDHYMKKIPKISSEFEPFAFKGILSFIDHLEGLCPLKVEYKEFFTKLKDFMAFINSASGSSAEFHTQLKTKSEELFKAITKMGGTAGASAHTTKLIESLMSMGKTLAEYKRSGSQTLTSEQRTELVTSMSRWAQTIGQFVKSVTETNGGANIDLKSLGCGGATGISTSMETGSTASGSTNMETGSTGGSGSPSGDTGSPASGGSESGSPSNETPAEGGSGSPSGSPIDSSSGAGSTGSETSTEAETTAGSGSGAGAGETPGAAGPSGSPTGSPTESEESSKDESSKDESSKDESSKDESSKDESSKDESSKDESSKDKSSKDDKDESSKDESSKDESSKDESSKDESSKDESSKDESSKDKSSKDDKDESSKDESSKDKSSKDDKDESSKDESSKDESSKDESSKGESSAESGESSKEASGASSTETQSETGAESGSTDGGASAGGPSGEPSGDAGAGAPSGSTSDMSAGGASASGGASGTSTQTSAEGESSMNSGGSSYADTTGGSAAEGSASSPSGSASGSSETSSTTGSENSSYQAGGSSAGGPSGSTTAGSAAGESTTEESSEGGSGASENQSVKGKKGTASYEGSSSYKKSQSQSSESAKSSFKHSSEKNAGSS

>Bol042985

MARISLALCLMLVVTSSVIYEARGHFLLKDYLTTKFPSKSSEFTPYVNTGLTEFLTDLERFCPPTPEFKSFFTEFKSFFSSIETSSSTSQNIDMEKKGDGLFKAVSAITGGAGQKSAEAGSFKSTMISMAKTLVEQKKSTTAITSTEKKTLVTSMVQWTKTIATTVKTACEKKGKKIDINSFGLNVDVNSVMTVSESRQSSSSSSKSSSESSSKSSSYAARAETAASAKAKETTGASAEKGEKKAEKKAEKAEKKAEKNEQKAATSSAKTKEASSTKAGASSSTKTGTIFRDTTGGSAGSPRGSPTAAKDKTAGSPRGSPTAAKEKTSVKGTGKAAASVNQQSNAGSSRGSAAASTNQQQSNAASSRGSAASTNQESASSKSSSTTSVTEIEKETSQETTSFISGLEKRFAQKAELKPFFEKLKASMTASSRVSSTKSEQEYTNTAKSTTGKLSDAMSFVGSRFSKSAEMKSNIQTTQQQLIKNLQQFQNLNSQIVGEQKVSSTKETEIKKTMSKIEQVTTQFVETAASSSSSSSKQETAASQQSQKKTMASSKKETAASSQQQTQQQTQQENGMGRLKTN

>Bol043019

MEHVSSATTATTTSSTTVAELEAETSKEVMEFIMKLEKKCPPKEEYKSFFEKLKATMVASAKVTQEKKKGFFSAAAGKISDAVSFIGSKFTGKSAEVKKSMETYQQEVAKSLQELEAIHKKIIEANQGKVEGSVAVTAEQKTEIKQTITRWETVTTQFVETAIQTEAASNTTVGVDKVKLP

>Bol016990

MAKTSLTIYLSLLVALSTVYETQGTFSLPLYLKNFPKVGHDFESFAYKGMMDFMGDLEGKCPQTTEFKDFFLKLKDYMACYSSTAPGSKDLQVELSIKSETLFRAMSDFSGTKGGTSEDSWTLVDGLLSMGKSLVEMKKSGSKEITFEQRKEIIQSMVKWTRGIGLFVKKVSESKGQSIDLSSFGIDYDNNVSSPSERALYETQGTFSLPHYVKDFPKMSKDFEPFAYKGMSGFLGALESKCPATAEFKDLFVKVADYMACFKSGIKVEMQEKSVKLFRAISVLDGTNGGTSVSYSPKY

>BraA09003707

MARVHLLLCFTLLFASVTLFDAASAFLKLKPSLPQIEEPKTVGDVEGYTVQVVMVFVGDLEKECPKTSKFKMFFDKLRGFAKYVCPLKISGKKDDDDMKA

KEAGILKTIASFAIGRIKREIQEEKQEAIETFKFMKSLAGRILGGRKKEEKATTTLTPEQLKEIKDGILKWQTVIVKITNTMVVSTTNTEGSAGSNPGAG

TPSTDTNNESQGTPSADKNNKSQGTPSTDTNNESQGTTGGSSSPNSGSATGSPSNKPSTGSNPGAGTPSTDTNNQSQGTKNTASSGSTTTSQTTEVTVTE

VETQTSEQVMTFLMNLEKKCPPKEEYKQFFEKLKSTMAGSAKVASPKKKGGLFSMIKGAVGKIGDAMQFIRSRIGNKSAEVKKSMETYQTEVIKNMEELN

AIYAKIVSQNQSKKGGAMTCTPEQQAEIKTTITKWEQVTTQFVEVAIKSETSTTTSTSTSTSDSRGTAQAN

>BraA02003786

MANKNVLGLCLMFLVVSSVVYEAQGTFLLKMYLKRNFLRNARNFTPFACKGMLLLISRLKRGCPATEGFKTFFSLFTSYVKFIKTASTTSNTDTQLTTKV

DALTNAISVLTGAKSGSNSNFRETMLSMGKTLIEQKRSTSQRMTHQQKKMLVAAMVQWTKTVVTLVKTAVETAGKSIDVSNLGLDVDVNSIVGSGSDESP

ESVTLVKTAVETAGKSIDVSNLGLDVDVNSIVGSGSDESPESGSGSNTNSGSIIYEDTTGGGSGSPSGSPGCSPSSTTTTQSDGSTMGSQSGTATESPSG

TTTESPSGTTTDSPSGSPTDTPSGTTTETPSGTGTGSPSGSPTDSPSGATTDSPSETTTESPSDTTMGSPSGSPTDSPSGETTDSPSETTMGSPSGSPTD

SPSGETTDSPSETTMGSPSGSPTDSPSGATTESQSDTTTESPSDTNTGSPSGSPTDGPSGATTESQSDTTTESPSGSPTDSPSGATTDSPSGTTTDSPSG

ATTDSPSDTTKGSPSGSPTDSPSGETTDSPTGATTESPSGTTTESPNGSPTDSPSGTATDSPSSTTTGGPSGSPTDSPSGSPTDSPSGSPTDSPSGSPTA

SEDATSNTNSQSSQSSTSSSSTTSAKEVEIQTSKEARSFIHGLEKKYAGTVQLDTFFEKLKTSMSASTKISNTDEKRFVSKMSSAVSSVSEAATTVTSKL

AKSPEAKNRMESSQEKLMNTYKELEEVNSKIVSENKGKTVSSAQKSELKQILSKWEQVTTQFVENLVSSSSSSSSSSQSQQSQQSQKSHQSEQSQQGSTM

KTETN

>BraA02003784

MTRFPLAICLMCIIVASSTVYEAQGGFLLRHYLSKLPRLANEFEPFAFKIMIRFIDNLESLCSSKVEYKEFFSKLKAFLIFINSTAGKSSSSEFESQLKA

HSEGLFKAITALGVKASADTSKLIESLMSMGKVMAEYKRSGSLTMTSEQRRVLITSMMKWAQVIGQFVKTVREKTGDGDIDLPSLGIGGGDDDAGSAGGG

SSGSGSSSMGAVSAGGGSSGGGASSLGAGSAGGGSSGGGTSSMGAGSAGDGSSGSGSPYPGAGGTGGGSSGSGSSTMGAGNTGMGDGSTGGSGNPSSDSG

TPSSETGSHAGGGGGSPGGSPTDSSTAGGGSSMGGGGSSSDTGSTTGAGGGSSGDTGSTTGAAGGSSGDAGSITGAAGGSSGDTGSTTGAGGGSSGDTGS

TTGAAGGSSGDTGSTTGAAGGTGAAGGSSGDTGSTTGAAGGSSGDTGSTTGATGGSSGDTGSTTGVGGGSSGDTGSTTGAGGGSSGDTGSTTGATGGSSG

DTGSTMGAEGGSSSDTGSTTGVGGGSSGAGGGSSGDTRSTTGAAGGSSGDTGSTMGAEGGSSGDTGSTTGAGGGSSGDTGSTTGAGGRSSGDTGSTTGAG

GGSSGDTKSTTGAAGGSSGDTGSTTGAGGGSSGDTGSTTGAGGGSGSGGTSAGGASGSPTDDSSGSGESAGGETSGAGGSSGNTSNMSTEESSSMGGGKS

SKDSKIATGGNASEESTSGGASGGSTKTTAAGERSMNNGDSYSDSTGGNTMGSPTGSPSGRGGSSFTGSETGSSSYQAGGASAGGPSGSTTDSSAAGANS

MNSGYSAKGSSTTSAQGSTQGGSGLAEGASEGKSFKGKTGSTRYEGSSNYQKTHSKSLDKSSFSHSSEDKSSGNV

>BraA02003783

MKKVSLLCLCCITILATSLSFNVVSAHEIAVNAPNTIKDIESYISNRAIGFVLKLEDECPIREKLRSFFEKLKDLLKLESSVTPMIEENEPKTFMSHMKS

KADNLLQTMLMIGRGLLSSSVRKEMFKVMKSLTELHAAIGKVITEKHIKGDESMSLSLEQKNAVENAVSQWEVTITRIVEIVVEVKSKSSSAASGEESSN

TGQNSTSVNETTLESNKGNSESMQENSDNVSESNEDSKNDVAMKAKEGNRVEDVKGEVQGENIGDSTKDVNLENKEDFKGDANSEKKLEVQGGNNGDSQI

NKNLDTKEDVKSEVDARNDGSSMTEESETQGNDEVSTKNTNLENKGSEATLNDEYHTKENGKNTQENNAELVNDENLEKNKDNKEVKDNGLVETNTDHEN

STEKKREHIQGGTEVSINEKKMHTNSGNADSNKEKEVEVQGESIGNTTKDLNLENEEDLKGDANSEKPLEVQEGSIGDSHINKNLDTKNGVKSEVDARND

GSSMTESEDTKGNGQFFTKDTTLENKESEARINDEDHTKENGEETKENNGELVKDENLENNKDNKEVKDDGFVETKTNHENSTEEKREHTQGGTEVSMNG

EVMPTKGGNVDSNKESEVEFQGESIGNSTKDINLKDKEDVKSKVETNKNDESSMKEKQRYSQGNDRVSTKDQNLESIGAHKETMNHKTVEAMKNNGDQMK

DKREENQQRESMKDENSENKEDKNELKDNELVETKTSNESSVKENKEEAQGSNEVSMNGQMDSLGANVDSNNKEAVEFKKRHVEDSNKDSKPKVEAIKNE

GNSMEEKGEVAEGNNGVSTEDKNLENIESEQELKNNIPAETKTNKETTMEENKEKDHVNNDESTYDGNKNESKDDKLDKTKKNKKDHMDKKWREAKGNEK

SFMREHQKEVSVDNKGDKKEIKDGKPLEVKENKENKEETQRNSSGQYTNVDNKEGKDDKSKEAKENKESSNNKIQEKVQGSGRDTTKVDRKKDRSSIDNM

DIEVQKGSEESMKEAERKEHEKNKSTKIEKEEKKQKEHEEHKSKKKEEEKRKEHEKNKLKENDDDKKKQKEHSGYKSKKKEKEKRKEHKEHKSKRNEEER

KDHERNKLKNEEEKKKQKEHEEHISKKKEEEKRKEYERNKSKKNEEEKKKQKEHEEHISKKKEEEKRKEHERNKLKNEEDKKKQKEHEERISKKKEDEKR

KEYEKNKSKKNEEDRKKHKEHISKKKAEAKRKEQEKNKSMKSEEEQKRQKEYEEHISKKKEENKRIEHERNKLMNGEEKKKQKEHEEHISKKKEEEKENE

HEKNKSKKNEEEKNKQKEHQEHISRKKEEEKRIEHERNHLKNEEDKKKQKEHEEHISKKKKEEKMKEQENNKSKKNEEEKKKQKEHEEHTSKKNEEKKKK

KKKKEHEKNKSKKNEEEKKKQKEHNEHLSKKKEEEHRIEHERNKLKNEEEKKKQKEHEEHISKNMEEKKRKEHEQNKSKKNEEEKNKQKENDEHMSRKKE

EEKRIEHERNKLKNEEEKKKHKEHEEHKSKKKEEEKKKEHERNKLRKNEEENKKQKEHEKHISQKKEEDKRKEHEEHTSKKKEEEKRKEHEKNKAKKNEE

EKMKEHEKNKSKKNEDKKSEHEQYKSKKTHKENKSNNNEEEETKKGEYEETKSKKNEEQKKREREEEKKKEHEENKFKKEEEEKKMKRDYDNNKSKQNKE

DEKEKTKEDVKMSNDDKSKDHEKEMLANAKEKSEESEKEMQAETLSRSEESDKEMQADAPTKSEESVKEMQTKTHARSEESEKKIQAEAQARSEEHEKNM

QAEAQAKSEVSEKETQTKDQAKNEEPEKEMQANAHTENEESEKEMQAEAHARSEESEKEMQEEAQVKSVESEKRKHTKAQAENEDSSMKKQEEIQETNVG

EKKDQGDLKEEGSADGKTTEIKEGSIEEDSKDGETVENNGVNEEAMEEHSKDGKVVETNGGKEDSMEEGPKDEKLIKDNENTTEISGGKNSTVGDSSSDD

GKIVENNGGKEDSLEEGEDGKTVEMHGGKNSTEEVSKDSTTEINGGKEDSIEEISKDGDIDEINRDKEDSMKEGSENGKTAEIDGRKEVPIEEGSKDGKT

LENKNSTESSVEEGSKDSQTVEVNEGQDKSMEKGNGSQEVTMEEGSKDGNTTELNGSKDNSMEEGSTDGNTVETDGVKEDSMKDKATEVQGNNNSSTSDT

SMATEVQGHNNSSTSDTSTDVKGDSAYINEDSTKNTTLEAQGGSAGGSTNGTIGETTKSNDSMSNQNVQHVEGDSIDINEDSTKNKTMEAQGGGVGDSTN

GEKEETTESNDTMNNQNVQHGENNANSTNNQTSESSSTEKKVTTDIESSTSKEVTSFISNLEHKSPGTQEFQSFFQKLKDYMKYAWPVSSTFEATDSRSY

MSEMTNMATKVSDAMAVLQAKKLGSGLMKTTLQGYQQEVMKTLTILQSVLSKAVSGQQSQNSGSLTLTLSQQQAIKEITLKWEQVMSQFVRVATESEKQF

SMETSTGNGFHMKKSFSSSSSSSSSSSSSPGLDFNLNSESPEMVDVNS

>BraA04001304

MSRFQNLLCFTILLATVTFFNVASAHVKIKLALPQTGDPISVGDVEPYTVKIVSTFVADLEKECAKTEKFRHFFEKVNAFSKCVCSVSMDHESHMKAKAG

SLFQAISALGSDENGSKGGMVNKLQKEKTEAMETVKMLQSIGKEGRQGSAKSKGGSNDDKSSKNKGKSSENAKNQQSKYGKVEKKKGGVDQSRNKTAERL

RAEAADELD

>BraA06004112

MEFIMKLEKKCPPKEEYKSFFEKLKATMVASAKVTQEKKKGFFSTAAGKISDAVSFIGSKFTGKSPEVKKSMETYQQEVAKSLQELEAIHKKIIEANQGK

VEGSVAVTAEQKTEIKQTITRWETVTTQFVETAIQTEAASNTTVGVDKVKLP

>BraA06004146

MAKFPLGICLMFMITTSTIYEVQGHFLLDHYMKKIPKISSEFEPFAFKGILSFIDHLEGLCPLKVEYKEFFTKLKDFMAFINSASGSSAEFHTQLKTKSE

ELFKAITKMGGTAGASAHTTKLIESLVSMGKTLAEYKRSGSQTLTSEQRTELVTSMSRWAQTIGQFVKSVTETNGGGNIDLKSLGCGGATGISTSMETGS

TATGSTSMETGSTGGSGSPSGDTGSPSAETGSPAGSESGSPSNETPVEGGSGSPSGSPSDSSSGAGSTGGSESSAEAESTAGAGSGAGAGETPGAAGPSG

SPTDSPTESEESSKDESSKDESSKDESSKDESSKDESSKDESSKDESSKDESSKDESSKDENSKDESSKDESSKDESSKDESSKDESLQG

>BraA06004150

MARISLALCLMLVVTSSVIYEARGHFLLKDYLKTKFPSKSSEFTPYVNRGLTEFLTDLERFCPPTPEFKSFFTEFKSFFSSIETSSSTSQNVDVEKKGDG

LFKAVSAITGGAGQQSAEAGSFKSTMISMAKTLVEQKKSTTTITSTEKKTLVTSMVQWTKTIATTVKTACEKKGKKIDINSFGLNVDVNSVMTVSESRQS

SSSSSKSSHESSSKSSTYAARAETAASAKAKETSAKTKEASSTKAGAAFRDTTGGSAGSPRGSPTAGKDKTAGSPRGSPTAAKEKTSVKGTGNAAGSVNQ

QQSNAGSSRGSGSAAASTNQQQSNAASRGSAASTNQESKKTASSKSSSTTSVTEIEKETSQETMSFISGLEKRFAQKAELKPFFEKLKASMTASSRVSST

KSEQEYTNTAKSTTGKLSDAMTFVGSRFSKSAEMKSTIQTTQQQLIKNLQQFQNLNSQIVGEQKVSSTKETEIKKTMSKIEQVTTQFVETAASSSSSSSK

QETAASQQSQKQTMASSKKETAASSQQQTQQQTQQQTQQQTQQENGMGRLKTN

>BraA06004151

MAKFPLGICLMFMITTSTIYEVQGHFLLDHYMKKIPKISSEFEPFAFKGILSFIDHLEGLCPLKVEYKEFFTKLKDFMAFINSASGSSAEFHTQLKTKSE

ELFKAITKMGGTAGASAHTTKLIESLVSMGKTLAEYKRSGSQTLTSEQRTELVTSMSRWAQTIGQFVKSVTETNGGGNIDLKSLGCGGATGISTSMETGS

TATGSTSMETGSTGGSGSPSGDTGSPSAETGSPAGSESGSPSNETPVEGGSGSPSGSPSDSSSGAGSTGGSESSAEAESTAGAGSGAGAGETPGAAGPSG

SPTDSPTESEESSKDESSKDESSKDESSKDESSKDESSKDESSKDESSKDDSSKDESSKDENSKDESSKDESSKDESSKDESSKDESSKDESSKDESSKD

ESSKDESSKDESSKDESSKDESSKDESSKDESSKDESSKGESSAESGESSKEASGGSSTETQSETGAESGSTAGGPSGEPSGDAGAGAPSGSTSDMSAGG

ASASGGASETSTQTSAEGESSMNSGGSYADTTGGSAEGSASSPSGSASGSSETSSITGSENSSYQAGGSSAGGPSGSTTEQSSEAGAAGSTEGGSGASVN

QSVKGKVGATSYEGSSSYKKSHSQTSESGQSSFKTEKKAGSS

>BraA08002716

MARVHLLLCFTLVFASVTLLDVTSAFLKLKPSLPQIEDPKTVGDVEGYTVNVVMVFVGDLEKECPKTSKFKMFFDKLRGFAKYVCPLKIFGKKDDTDMKA

KEAGILKSIASFAIGRIKSEIQEEKQEAIETFKFMKSLAGRILGGRKKEEKETTTLTPEQLKEIKDGILKWQTVIVKITNTMVVSTTNNEGSAGSNPGAG

TPSMDTNNQSQGTPSTDDTNNQSQGTPSTDKNNQSQAGTPSTDTNNQSQGNTNTASSRSETTSQTTEVTVTEVETQTSEQVMTFLMNLEKKCPPKEEYKQ

FFEKLKSTMTGSAKVSSPKKKGGLFGMIKGAVGKIGDAMQFIRSRIGNKSAEVKKSMETYQAEVIKNMEELNAIYAKIVTQNQSKKGGAMTCTPEQQAEI

KTTITKWEQVTTQFVEVAIKSETSTTTSTSSSTGTAQPN

>BraA09000381

MANKNVLALCLMFILVSSVIYEAQGTFLLKMYLRRKFFKKAMQFTPFACKGMTFLLHRLKGGCPATKGFKTFFSLFISYVNFIKTARVSKTTDSQLTTKA

DGLAKAVSVLTGARKDVSNNFRETILSMGKTLIEQKKAGPQRVTYKQRKVLIVALVQWTKTVVTVVKTAVETAGKTIDTSNLGLDVDVNDLLGGEKDDSP

TPTTPTTPATPATPATPTTPTTPATPATPTTPTTPATPATPTPSTPTTPTPTTPTPSTPTTPTPSTPTTPTPTTPTTPTPSTPRTSTTPTPSTPTTPTPS

TPTTPATPTPSTPTTPTPTTPSTPTTPTGTTTNNAATNTNSQTSQSTTSTASAKQVETQTSQEVMSFISDLEKKYASKTELNTFFETLKSTMTATSKIAS

TDEKTFVSGVKAAAGTLNEAAETVTEKLGTSAESKQKIESSQQQLMKTFKELEDVKTKIVSESKGKTVSSTQQTELKQTLTKWEQVTTQFVETAASSSST

SSSTQSQQIQQSHQSQQSQQSQKTQQTQQTQQIQQGSILRAQTQT

>Csa02g069680

MARISLAICLTLLVTLSTVYETQGTFSLPHYLEKFPKVGKDFEAFANKGMSDFLGDLEGMCPKTAEFKNLFATLKDYMASFGSGSSKDIKMELSEKSEKLFRAMSVFDTSKGGTSDDSWNLVDGLLSMGKGLMEMKKSGSQELNFEQRKELIVSMVEWTRGIGLFVKAASESKGQSIDLSSFGIDYDNSVESPIKRAMYETQGTFSLPHYLENLPKKAQDFEPFAYNGMSHFIDSLESKCPATTEFKDFFVKLEDYMAIFKSASSGSKDFKVDMSIKSQRLFKAMSVLTGTQGSGISVDSWRMLDGLLSMGKFLVEMKKHGSNEITFEQRTEMVGSMVQWARAIGLFARVASEKKGKTIDLSPFGIFHARNYGKGNFKTSSEL

>Csa04g017990

MARFPIEICLMLILVASSTIYEAQGTFLLRHYMRKFPKMSQDFEPFAYKGMLSFVDNLESMCPLKGEYKDFFSKLKAFISFINTAKGSSSEFQSQMKSQSEGLFKAISALGVKGGSSADTSKLIESLMSMGKTFAEFKRSGATTMTSEQRRELVISMSKWAQVIGQFVKTVGEKSGDGGNIDLSSILGIGGSGGDNGSPSSDTSFPSTDAGTPTNSGSYPDSTGDSGTSAGGPSGSTTDSGDGSMGDTGSGSTAGGPSGSTTDSLMGAGGGAAADGENGRTAAGGENGGAAAGDESGGAAADGESREAAAGVESGGAAAGGENGGAAADDESGGAAADGESGEAAAGVESGRAAAGGENGGAAADGESGEAAADGESGGAAADGESGEAAAGGSTQRTESGGANSMGGATQRTESGGSAAVGGESETESSMIGGGAYIDSTGGSPASSPSAGGPSGSTTKNSMEGVAGGSATVTSYQAANYQKTHSKSSGKSSFSHSLEEKNSGGTNADS

>Csa04g018010

MAKNLLAICLVFMVASSVVYEVQGTFLLKLYLRTRFPRRCMDFAPFAAKGMLMLLSNLQDGCPATREFKEFFSRFKSYISFIISASSSTKNIDTEIDGRCELLAKAMSALSGSTSSKTSSDLKMTMLSMGKTLVEQKRQGSTILSFQQKKELLKAFVDWIRLVFTFVKSVAEQKGKTIDESSYGLDVDVNSSIGSGSDSDDDSSDSEGSSPQAGTPTPTTPSTPSTPSTPTPTTPTPSTETGSSPQGETPSPTTPTPTTPTPNTETGSSPQGETPAPTTPTPTTPTPSTETGSSPQGETPTPTTPTPSTPSTPTPSTPTPTPATPTPTTPTPSTSDSPSGKTSEKGSESSSGGASTKEESNSQSGTPTGTPSGSTPTDSETSAQGSATSQKKESSSVGDSSQTNEESNTGKSNSQSGTPTGTPSGSTPTDSETSAQGSATSQKKESSSVGDSSQTNEESNTGSNSSEKKESKSQSSSTTTSVKEVESQTSSEVMSFISNLEKKYSHNAELKVFFDKLKTSMSASSKLSTSNAQEFVSGMKSAASKLSEAMMFVCSRFSQSQETKSNMETSQQQVLKTLKELQDINSQIVSGKKVTSTQKTELKQTITNWEQVTTQFVETAASSKSSSSSSSSSSHGSVKIAQTN

>Csa04g018020

MVFIRSRVESKSAEVKKSMETYQGEVMKTLQELETIRSQIVSQNKGKKEGSLTCTPAQQMQIKQTITKWEQVTTQSVETATQTQTSSTSSTGKMVAN

>Csa04g018030

MARVQLFLCFTILLASVALLDVVSAHLKLKPSLPQIEDPKTLKDVEPYTVKVVMVFVSDLEKECPKTSKFKVFFEKLRAYAKYVCPIKRKDQVDYDRDLKAKAGGLVQAISSFAIGTIKEEIQEEKMEVINTFKYMRFVATKILGSRKKEESEESMKLSAEQQKEIKEGILRWETIIARITNTMITSTKDSSSSETSTTEKGDSSDKSKSSGSEGKGSSEDTQESKSNSGSPSGSPSSSPSGSSGKETDSKANKTTDEKSSAASGKETDSKDTKNTDEKSSATSGKETSTKDTKINGESSSAASGKETSSTDTKANGESSASENTETSQSGTVNVEQVEAETSKQVSSFIMNLEKKCPQKEEYKAFFEQLKGTMIAPVKEGKDLFTRIKSAAGKVSGAMAFIRSRVESKSAEVKQSMETYQGEVMKTLQELETIHSQIVSQNKGKKEGSLTCTPAQQMQIKQTITKWEQVTTQFVETATQTQTSSTSSTGKMVAN

>Csa04g018040

MARVSLGICLMLVVASSVIYEAQGTFLLKLYLRKNFPKQCNEFTPYANKGMLSFVTNLEGNCPATAEFKNFFTQFKSYMSFIETAWASSKNVDTEMTTKCNGLFKAMSAISSGKGEKSADAGGLKATMLAMGKTLVEQKKNTKIMTLTQKKELIGSMVKWTNMLATFVKSASEKKGKSIDIGSYGLDVDVNDSSIVGNTESSSASSTKAGSVSTKTKESSSGASSAASKTKESSGASSGGANKDTTGKNSGSPSETPNAKPSKGSESATEDANAKGSISGKSSFSANSATKTTNEKRSKSSSQSSFKSSSTISVKQVESETSKEVMSFITQLEKKYSAKTELKVFFEKLKASMQASSSIASKTSKDYVSATAAATGKLSEAMALVGSKNVKSAKMKSNMETSKDEMMKCLKQIQDINSKIVSGKTASSTQQSELKQTITKWEKVTTQFVETAASSSSSSSLSSSATQQQGSARMVENN

>Csa04g018050

MLATFVKSASEKKGKSIDIGSYGLDVDVNDSSIVGNTESSSASSTKAGSVSTKTKESSSGASSAESKTKESSGASSGGANKDTTGKNSGSPSETPKAKPSKGSESATEDANAKGSISGKSSFSANSATKTTSEKRSKSSSQSSFKSSSTISVKQVESETSKEVMSFITQLEKKYSAKTELKVFFEKLKASMQASSSIASKTSKDYVSATAAATGKLSEAMALVGSKNVKSAKMKSNMETSKDEMMKCLKQIQDINSKIVSGKTASSTQQSELKQTITKWEKVTTQFVETAASSSSSSSLSSSATQQQGSARMVENN

>Csa04g018280

MARVQLFLCFTILFATASLLDLASAHLNYKPSLPQLEDPKTVKDVEPYTVKVVMVFVADLEKECPKNNKFKVFFEKLRGFAKYVCPIKRRDQVDYDRDLKAKAGGIFKAISSFAIGKIREEIQEEKMEAINTFKFMKSVAAKIMGGRRQDESEDTMKLTAEQQKEIKEGILKWETIITRITNTMVMSSSNSSSSEESNVEKEDSSSNSKSSGKESSTSAKGSSEETKDSNSNSGSPSGTGSPSSSPSDASGKETSTKDTKTKGESSSDASGKETSSKDTKPAGETSSDDASGKGTSSKDTKNTEEESSATSGKETSTKDTKTKAESSSNASGKETSTKDTKANGESSASEKTETSQSSTVNVEQVEAETSKQVSSFIMNLEKKCPQKEEYKAFFEQLKGTMIAPVKEGKDLFTRIKSAAGKVSGAMAFIRSRIESKSAEVKQSMETYQGEVMKTLQELETIHSQIVSQNKGKKEGSLTCTPAQQMQIKQTITKWEQVTTQFVETATQSETKTSSSASSKMVAN

>Csa04g018640

MRKVLLLFLCVIVVTNSSLNVVLAHEMVVTPPNTIKDIEPYISNRALGFVLKLENNCPIREQLRSFFEKLKDLLKLESSVTPLIENNESDTFKFDLKSKSENLLQTMFMLGRGLLSSSVRKEIFEVMKSLTELHAAIGRVIIEKHIKGDDDSMSLSVEQKNAVENSITEWEQTITRIVKIVVEVKSKGSTEAAVHESNTSEHKNVSMDSNMVDTNGEDSESTQEKAYGIKRSYGGDVSMETQEGNKGEDVKEGNFVSKNDETKVIKDENANSNIEKEIEGQGENLGDSTNDKNLETKEDVKSEVQAKNDGSSVTENLEEAQKNNRGSTMDMNLENKGSGDEAKDNKMVNATTNDEDQKIEETQENNGELEKDKNLENNVGNKILKGNGSTKKNTTNEISIEEKREETQKSHEISMKEKQEETQRSHEIYMNKETIKDKNKNLNKEKGTNFQGESIEDLTKGTISNDKKNVTSEVNSNGTNESYQEDQGNAGVSTKDKNLGADGEKKYDKSVEVMKNEDYTKKQREENQGNNEGSMKNENLENKEDKKESKDDQPIETKTNNETSKEEKDEHRQGGHDISMNGKIMENKSGNAYSNKEKEVHVGDSTNGNNIESKDDAKLEAELNKNNGGSRKEKGEEGQGTNEGSLNNKNLETQVSQTDLKDDKSVADKENLRSSMKENQEEAQRNERDKKEASDDKSAEIKGKNKESSKNKSIKTNENKKNNREDFVHGKKKESKTLETGEKKEFKDVKLVKAKETKEISMKENREKSQGSSSGGLSKEDNKEEKKESKNYQTGKKKANKEKQESAQENTRVHTKIDNEKDSKNLKDDKSVETKANKENFVKKKQEQAQRNDIGSTKVKDFSSEDARLKKNTKEDKGSSNDIDIDVKNRSGESTKHKKEKKKDIKKEEKKDTTTDVKSKDKRKDNHEHEKSRKSILKKQKEDKKEKKESGDNILKNKEKKGYEDNKLKIKDESKTENTNSKDYKKKEENKKEKQDFESNILKKQNEDIKEKGEFGDVKSKNKEKKEWDKKEKKEHMNNRSKIKEENKQKKTNSKDYKSKRKEEDKNKKINFEHKNKEKKDSKDHKSKEKKEEKQEKKEFKDRKANKNEVEKKEKKESENHKSKKKEKENNKYENTLKEKHEANKKEKNEYEEKKSMIKEKKEFKDHNMKKNEEKKIVTKXSMEKTFKANHTDDKSQEDYKTNETNGGKEVNMEEGSKDDKKVKANGGKEDSMEEKSKDRGTNSMKNTSEDSNIVDTSGDKKXEEEVSRRESKKKEKKNSIHNQQKKKEQDKKESNNKITKKNEEDRKKKTIFEAKNKKREENEEKKEESQDNKSKHSKKHIGNSKQGGFQSNTEYSMKEKEDKKESKNFESRETEKQKKNHGQEKKVGIQVSNKDSEKNKKKTKEDNKVSKHGKSEKYEKEMEATTQRDNKKAKKEMQADDQVKGEESLEYKKGKSKEDNEDKVGKEKNEHFKKKSGKMIEDKETLDKKIEDTKSKKKQEDSKASSMKKHEGVQEANKESNISEKKLQNESSDDASKENDEALMKQKKIDEGDTNIKKSKGKTKETNRDLVNGTSIEVRGENKESRDGKRIESYQSKNNSIEEVSNDGKKVEIKGGKESSLKEGSKDSIRGKEENSKADKKNEHNKAKRDFNERGSKDGNTTEINEGKEDSMNKDSKNGMLVETSGGKNHREEGSVDDKTTNINGNKKDLKDGNIVEISIGTNSTYESSKGGSTVKTNGGKDVSMEKTFKANHIDDKGQEDYKTNETNGGKEVNMEEGSKDDKKVKANGGKEDSMEEKSKDRGTNSMKNTSEDSNIVDTSGDKKDFIKENSEDAKRAETNGGKNVSVEENSKDGNINEVHKGQEDSTNKGFKEGKSDEINGGKEVTMEEGSNDDKTTGIIGGKDNSMEEKSKDSATENLNGATNLTADGKIVEANGRYXKQGGFQSNTEYSMKEKEDKKESKNFESRETEKQKKNHGQEKKVGIQVSNKDSEKNKKKTKEDNKVSKHGKSEKYEKEMEATTQRDNKKAKKEMQADDQVKGEESLEYKKGKSKEDNEDKVGKEKNEHFKKKSGKMIEDKETLDKKIEDTKSKKKQEDSKASSMKKHEGVQEANKESNISEKKLQNESSDDASKENDEALMKQKKIDEGDTNIKKSKGKTKETNRDLVNGTSIEVRGENKESRDGKRIESYQSKNNSIEEVSNDGKKVEIKGGKESSLKEGSKDSIRVSSTLKAKDSSSYMSEMVNMATKLSDAMAVLQAKKSGSGQMKTTLQGYQEEVMKTLNILQSVMTKAISEKQTTSGGLFTLTLSQQEAIKEILLKWEQVMSQFVKVATESEKQFSIEISTGNGYHMKKSSNSSSSSSSISSSTSSSNLDFQVNGETLKGLDMDG

>Csa06g010040

MVVAPPNTVKDIEPYISNRALGFVLKLENNCPIREQLRSFFEKLKDLLKLESSITPMIENNESDTFKFDLKSKSENLLQTMFMLGRGLLSSSVRKEIFEVMKSLTELHAAIGRVIIEKHIKGDDDSMSLSVEQKNAVENSITEWEQTITRIVKIIVEVKSKGSTEAAVHERNTSEHNNVSMDSNMVETNGEDSESTQEKAYGIKRSYGGDVSMETQEGNKGEDVKEGNLVSKNDETKDIKGENVKSNIEKEIEGQGENLGDSTNDKNLETKEDVKSEVHAKNDGSSVTENLEEAQKNNGGSTMDMNLENKGSREEAKDNKMVNATTNDEDQKIEETQENNGELEKDKNLENNVGNKILKGNGSTKKNTTNEISIEEKREETQRSHEISMEEKQEETQRSHEIYMNKETTKDKNKNLNKEKGTNVQGESIGDLTKGTISNDNKNVKSEVNSNGTDESYQEDQGNAGLSTKDKNLGADGEKKYDKSVEVMKNEDYTKNQREENQGNNEGSMKNENLENKEHKKESMDDRPIEAKTNNETSKEEKDEHRQGGHDISMNGKIMENKSGNAYSNKENKVHVGDSTNGNNIESKDDAKLEAELNKNNGGSRKEKGEEGQGTNEGSLNNKNLETQVSQTDLMDDKSVADKENLRSSIKENQEEAQRNKRDKKEANNDKSVEIKGKKKESSKNKSIKTNENKKNNREDFVHGNNKESKTVETGERKESKDAKLVKAKENKEISMKENREKSQGSSSGGLSTEDNKEEKKESKNYQSGKKKANKEKQESAQENTRVHTKIDNKKDSKNLKDDKSVEAKTNKENFVKKKQEQAQRNDIGSTKVKDFSSEDARPKKNTKEDKGSSNDIDIDVKKRSGESTKHKKEKKKDIKKEEKKDTTDVKSKEKRKNNHENEKSRKSILKKQKKDKKEKKESGDNILKNKEKKGYEDNKLKIKDESKTENTNSKDYKKKEENKKEKQDFESNILKKQNEDIKEKGEFGDVKSKNKEKKEWDKKEKKENMNNRSKIKEENTQKKTNSKDYKPKRKEEDKNKKINFEHKNKEKKDSKNRKSKEKIEEKQEKKEFKDRKANKNEVEKKEKKESENHKLKKKEKENNKYENTLKEKHEANKKEKNEYEEKKSMIKEKKEFKDHNMKKNEEKKKETNEHEEKKSKKKLEDKREKKKSQDGKSLKNEDIKKKKKENKNIEDKSVTTGDKNSESKKKEKKNSIHNQQKEKEQDKKESNNKITKKNEKDRKKKTIFEAKNKEREENEEKKEESQDNKSKHSKKHIGNSKQGGFQSNTEYSMKENKDKKESKNFESRETEKQKKNHGQEKKVGIQVSNKDSEKNKKKTKEDNKVSKHGKSEKYEKEMEATTQRDNKNAKKEMQADDQVKGEESLEYKKGNSKEDNEDKVGKEKNEHFKKKSGKIIEDNKALDKKIENKKSQKKQEDSRAFSMKKHEGVQEANKESNISEKKYQNESSDDASKENDEALMKQKQIDEGHTNIEKSKGKTKETNRDLVNGTSIEVREENKESRDGKRIESYQNKNNSIEEVSNDGKKVEIKGGKESSLKEGSKDSIRGKEENSKAHKKNEHNKAKRDFNERGSKDGNTTEINEGKEDSMNKDSKNGMLVETSGGKNHREEGSVDDKTTNINGNKKDLKDDNIVEISRGTNSTYESSKGGRTVETNGGKDVSMEKTFKANHTDDKSQEDYKTNETNGGKEVNMEEGSKDDKKVKANGGKEDSMEEKSKDGGTNSTENTSEDSKIVHTNDVKEDSENGNVNETNDNNIALRAETSGDKKNFIKENSEDAKRAETNGGKNVSVEENSKDGNINKVHKGQEDSTNKGFKEGKSDEINGGKEVTMEESSNDDKTTGIIGGKDNSMEEKSKDSATENLNGATNLTADGKIVEANGVKEDSKNGNINETSVKSKESKGDKVDMSGDSMKNKTIEAQESSNGDSVNGVEEENKENNDGGSNNNFMNNQTIEHGVNSTTKQELRTDIETNTSKEVTNFISNLEKKSPGTQEYQSFFQKLKDYMKYLCPVSSTLKAKDSSSYMSEMVNMATKLSDAMAVLQAKKSGSGQMKTTLQGYQQEVMKTLNILQSVMTKAISEKQTTSGGLFTLTLSQQEAIKEIVLKWEQVMSQFVKVATESEKQFSIEISTGNGYHMKKSSNSSSSSSLISSSTSSSNLDFKVNGETLKGLDMDG

>Csa06g010050

MARFHIAIRLMLILVASSTIYEAQGTFLLRHYMRKFPKMSQDFEPFAYKGMLSFVDNLESMCPLKGEYKDFFSKLKAFISFINTAKGSSSEFQSQMKSQSEGLFKAISALGVKGGSSADTSKLIESLMSMGKTFAEFKRSGATTMTSEQRRELVISMSKWAQVIGQFVKTVGEKSGDGGNIDLSSILGIGGSGGDNGSPSSDTSFPSTDAGTPTNSGSYPDSTGDSGTSAGGPSGSTTDSGDGSMGDTGSGSTAGGPSGSTTDSLMGAGGGAAADGENGRTAAGGENGGAAAGDESGGAAADGESREAAAGVESGGAAAGGENGGAAADGESGGAAADGESGEAAANGESGGAAADGESGEAAAGGSTQPSSTIYEAQGTFLLRHYMRKFPKMSQDFEPFAYKGMLSFVDNLESMCPLKGEYKDFFSKLKAFISFINTAKGSSSEFQSQMKSQSEGLFKAISALGVKGGSSADTSKLIESLMSMGKTFAEFKRSGATTMTSEQRRELVISMSKWAQVIGQFVKTVGEKSGDGGNIDLSSILGIGGSGGDNGSPSSDTSFPSTDAGTPTNSGSYPDSTGDSGTSAGGPSGSTTDSGDGSMGDTGSGSTAGGPSGSTTDSLMGAGGGAAADGENGRTAAGGENGGAAAGDESGGAAADGESREAAAGVESGGAAAGGENGGAAADGESGGAAADGESGEAAAGGSTQRTESGGANSMGGATQRTESGGSAAVGGESETESSMIGGGAYIDSTGGSPASSPSAGGPSGSTTKNSMEGVAGGSVTVTNTQY

>Csa06g010060

MAKNLLAICLVVMVASSVVYEVQGTFLLKLYVRRMIPRRCMDFAPFAGKGMLMLLSNLQDGCPATREFKEFFSRFKSYISFIISASSSTKNIDTEIDGRCELLAKAMSALSGSTSSKTSSDLKITMLSMGKTLVEQKRQGSTIMSFQQKKELVKAFVDWTRLVIIFVKSVAEQKGKSIDESSYGLDVDINSSIGSGSDSDDDSSDSEGFSPQGGTPTTPTPSTETGSSPQGETPTPTTPTPTTPTPSTPTPTTPTPSTPTPSTPTPTPSTPTPTTPTPTPSTETGSSPQGETPAPTTPTPTPTTPTPTTLTPNTPTPTPATPTSTTPTPTTPTPSTSDSPSGKTSEKESESSSGGASTKKESNSQSTGSSGNTDKDTTTSGSPSGTPTGTPSGSTPTDSETSAQGSATSQKKESNSGGDSSQTNEESNTGGDSSEKKESKSQSSSTTTSVKEVESQTSSEVMSFITNLEKKYTGNVELKVFFDKLKTSMSASSKISTSNAQEFVSGMKSAASKLAEAMMFVSSRFSKSQETKSSMETSQQEVLKTLKELQDINSQIVSGKKVTSTQKTELKQTITKWEQVTTQFVETAASSKSSSSSSSSHGSAKIAQTN

>Csa06g010070

MARVQLFLCFTILLASVALLDVVSAHLKLKPSLPQIEDPKTLKDVEPYTVKVVMVFVSDLEKECPKTSKFKVFFEKLRAYAKYVCPIKRKDQVDYDRDLKAKAGGLVQAISSFAIGKIKEEIQEEKMEVINTFKYMRFVATKILGSRKKEESEESMKLSAEQQKEIKEGILRWETIIARITNTMITSTKDSSSSETSTTEKGDSSDKSKSSGSEGKGSSEDTQESKSNSGSPSGSPSSSPSGSSGKETDSKANKTTDEKSSAASGKETDSKDTKNTDEKSSATSGKETSTKDTKINGESSSAASGKETSSTDTKANGESSASENTETSQSGTVNVEQVEAETSKQVSSFIMNLEKKCPQKEEYKAFFEQLKGTMIAPVKEGKDLFTRIKSAAGKVSGAMAFIRSRVESKSAEVKQSMETYQGEVMKTLQELETIHSQIVSQNKGKKEGSLTCTPAQQMQIKQTITKWEQVTTQFVETATQTETTSSSSTSSTGKMVAN

>Csa06g010080

MARVSLGICLMLVVASSVIYEAQGTFLLKLYLRKNFPKQCNEFTPYANKGMLSFVTNLEGNCPATAEFKNFFTQFKSYMSFIETASASSKNVDTEMTTKCNGLFKAMSAISSGKGEKSADAGGLKATMLAMGKTLVEQKKNTKIMTLTQKKELIGSMVKWTNMLATFVKSASEKKGKSIDIGSYGLDVDVNDSSIVGNTESSSASSTKAGSVSTKTKESSSGASSAASKTKESSGASSGGANKDTTGKNSGSPSETPNAKPSKGSESATEDANAKGSISGKSSFSANSATKTTNEKRSKSSSQSSFKSSSTISVKQVESETSKEVMSFITQLEKKYSAKTELKVFFEKLKASMQASSSIASKTSKDYVSATAAATGKLSEAMALVGSKNVKSAKMKSNMETSKDEMMKCLKQIQDINSKIVSGKTASSTQQSELKQTITKWEKVTTQFVETAASSSSSSSLSSSATQQQGSARMVENN

>Csa06g010090

MLRFVTLLQSLCPLKPRYTSFFGNLKSYMNFINSASGSANYDSDLKGKAQGLYSSISALSGKGGASADSSKVMETLMSMGKTLGHQQESSPTMSLGERKELIMSMAKWTQTIGQFVVSPAAQNGKTIDISSLGLDIDASATATGESTTNTETSTTTDTGTPSGGNTATGTGSTAGGTTTTGTGTTAVGSPTSSCTTSKCSNGDGSSFKAAVNIQHGGKASSQSQQTTSAQQDVTSAGSS

>Csa06g011330

MARVQLLLCFTILFATASLLDLASAHLKYKPSLPQLEDPKTVKDVEPYTVKVVMVFVADLEKECPNNNKFKAFFEKLRGFAKQDESEDTMKLTAEQQKEIKEGILKWETIITRITNTMVMSSSNSSSSEESNVEKEDSSSNSKSSVEAETSKQVSSFIMNLEKKCPQKEEYKAFFEQLKGTMIAPVKEGKDLFTRIKSAAGKVSGAMAFIRSRIESKSAEVKQSMETYQGEVMKTLQELETIHSQIVSQNKGKTEGSLTCTPAQQMQIKQTITKWEQVTTQFVETAAQSETKTSSSTSSSASGKMVAN

>Csa09841s010

MGKTFAEFKRSGATTMTSEQRRELVTSMSKWAQVIGQFVKTVGEKSGDGGNIDLSSILGIGGSGGDNGSPSSDTSFPSTDAGTPTNSGSYPDSTGDSGTSAGGPSGSTTDSG

>Csa09g020090

MVKDIEPYISNRALGFVLKLENNCPIREQLRSFFEKLKDLLKLESSVTPLIENNESDAFKFDLKSKSENLLQTMFMIGRGLLSSSVRKEIFEVMKSLTELHAAIGRVIIEKHIKGDDESMSLSVEQKNAVENSITEWEQTITRIVKIVVEVKSKGSTEHTVQESNTSEHNNVSMDSNMVETNGEDSESTQEKAYGIKGSYGGDVSMETQEGNKGEYVKEGNLVSNNDETKDIKGENADSNIENEIEGQGKNLGDSTNYKNLETKEDVKSEVEAKNDGSSMTEKLEEAKKNNGGSTMDMNLEDKGSGEEAKDNKMVNATTNDEDQKIEETQENNGELEKDKNLENNIETTKDKNRDLNKEKGTNVQGESIGDLSKGTISNDNKNVKSEVNSNGTYEKYQEAQGNDGVSTKDKNLGADGEKKYDKSVEVMKNGDYTKKQRGENQENNGGSMKNENLENKEDKKESIDDQSIEAKTNNETSKEEKGEHTQGGHDISMNGKIMENKSGNAYSNKEKEVHVGDSTNGNKMESKDDAKLEVELKKNDGGSRKEKGEEGQGKNEGSLENKNLKTHVSQTELKDDKSVADKENLRSSMKENQEEAERNERDKKEANDDKSVEIKGKKKESIKTNENKTNHRKDFVHGNKKESKMVETGEKKESNEANLVEAKENKEISMEENREKSQGSSSGGLSKEDNKEEKKESKNYQSGKKNANKEKQESAQENTRVHTKIDNKKDSKDLKDNKSVEAKANKEIFVKKKQEQAQRNDIGSTKVKGISSEDARLKKNTKEDKGSSNDIDINVKKRSRESTQHKKEKKKDIKKEEKKDTTDAKSKEKIKDNHENEKSRKSILKKQKEDKKERKESRDNILKNKEKKGYEDNKLKIKDESKTENTNSKDYKKKEEDKKEKQDFESNILKKQNEDNKEKRDFGDVKSKNKEKKEWDRKEKKEYMNNRSKIKEENKQKKTNSKDYKSKRKEEHKSKKMNFEHKNKEKRDSKDRKSKEKKEEKQEKIKFKDRKANKNEVEKKEKKESENHKSKKKEKENNKYDNTLKEKHEANKKEKNEYEEKKSMIKVKKEFEDHNMKKNEEKKKETKEHEDKKSKKKREDKREKKKSQDGKSLKNEDVKKKKKENKNIEDNSVTTDVKNSESKKKEQKKKESNNKITKKNVEDRKKKTIFEENISNTKAKNKKREENEEKKEESQDNKSKHSKKHIGNSKQGGFQSNAEYSMKEKEDKEESKDLESRETEKQENNHGQEKKVGVQVSTKNSKKNKKKTKEDDKVSKHGKSENYEKKMEATTQKDNKKAKNEMQADDQVKSEESLEYKKGKSKKDNEDKVGKEKNEHFEKKSGKIIEDKKTLDKKIEDTKSKKKQEDSKASSMKKHEGVQEANVESNISEKKYQNESSDDTSKVNDEALMKQKQIDEGHTNIEKSKGKTKETNRDLVNGTSIKVRGENKESRDGKTIESNQSKNNDGKKVEIKGGKESPLKEGSKDIIRGKEENSKVDKKNEHNKTKRDFNERGSKDGNTTEFNEGKEDSMNKDSENGKLVETSGDKNHKEEGSIDDKTTNINGKKKDNIVEISRDTNSTYESSKGGSTVETNGGKYVSMENFFKANHTDDEGQKDYKTNETNGGKEVTMEEGSKDEKKFKANGGKEDSMEEKSKDGMTTNLNGGTNSTENTSKDSKIVDTNDVKEDSENGNINEANDNNIAGTSGEKKNSIKENSEDAKRAETNGGKNVSVEENSKDGNINEVHKGQEDSTNKGFKESKSDEITGGKQVTMEEGSNDDKTTEIIGGKNKSMEEKSKDSETESLNGATNSTVDGSADGKIVEANGVKEDSKNGNINETSGTSKESKGDKVDINGGSIENKTIEAQESSNGDSMNGVEEENKENNDGGSNTKFMNNQTIEHGVNSTTKQELRTDIETNTSKEVTNFISNLEKKSPGTQEYQSFFQKLKDYMKYLCPVSSTLKAKDSSSYMSEMVNMATKLSDAMAVLQAKKSGSGQMKTTLQGYQQEVMKTLNILQSVMTKAISEKQTTSDDSFTLTLSQQEAIKEIVLKWEQVMSQFVKVATESEKQFSVEISTGNGYHMKKSSNSSSSSSSISSSTSSSNLDFKVNGETLKGLDMDG

>Csa09g020100

MAKNLLAICLVFMVASSVVYEVQGTFLLKLYLRRRFPKRCMDFAPFAAKGMLLLLSNLQDGCPATREFKEFFSRFKSYISFIISASSSTKNIDTEIDGRCELLAKAMSALSGSTSSKTSSDLKMTMLSMGKTLVEQKRQGSTIMSFQQKKELFKAFVGWVRLVFTFVKSVAEQKGKSIDESSYGLDVDVNSSIGSGSDSDDDSSDSEGSSPQAGTPTPSTETGSSPQGETPSPTTPTPTTPTPSTENGSSPQGETPATTTHTPSTETGSSPQGETPTPTTPTPSTPATPTPATPTPSTPSTPTPSTPATPTPSTPSTPTPTPSTPTPATPTPTTPTPSTSDSPSGKTSEKGSESSSGGASTKEESNSQSGTPTGTPSGSTPTDSETSAQGSATSQNKEPNSVGDSSQTNEESNTGSDSSEKNESKSQSSSTTTSVKEVESQTSSEVMSFISNLEKKYSGNAELKVFFDKLKTSMSASSKLSTSNAQEFVSGMKSAASKLSEAMMFVCSRFSQSQETKSNMETSQQEVLKTLKELQDINSQIVSGKKVTSTQKTELKQTITKWEQVTTQFVETAASSKSSSSSSSSHGSVKIAQTN

>Csa09g021110

MARVQLFLCFTILLASVALLDVVSAHLKLKPSLPQIEDPKTLKDVEPYTVKVVMVFVSDLEKECPKTSKFKVFFEKLRAYAKYVCPIKRKDQVDYDRDLKAKAGGLISIGIDLLIVLCKEYFSVRTNIDCNNHSHAWSEQFISPAIAPHLISSFCGKLENPHQI

>Csa09g021120

MARVQLFLCFTILLASVALLDVVSAHLKLKPSLPQIEDPKTLKDVEPYTVKVVMVFVSDLEKECPKTSKFKVFFEKLRAYAKYVCPIKRKDQVDYDRDLKAKAGGLVQAISSFAIGTIKEEIQEEKMEVINTFKYMRFVATKILGSRKKEESEESMKLSAEQQKEIKEGILRWETIIARITNTMITSTTDSSSSETSTTEKGGSSNKSKSSGSESSGSEGKGSSEETKDSKSNSGSPSGSPSSSPSGSSGKETNSKDTKTKGESSSDASGKETNSKDTKNTDEKSSATKSGKETSTKDTKTKGEISSDASGKETSTKDAKATGEISASETTETSQSSTVNVEQVEAETSKQVSSFIMNLEKKCPQKEEYKAFFEQLKGTMIAPVKEGKDLFTRIKSAAGKVSGAMAFIRSRVESKSAEVKQSMETYQGEVMKTLQELETIHSQIVTQNKGKKEGSLTCTPAQQMQIKQTITKWEQVTTQFVETATQSETTSSSSTSSTGKMVAN

>Csa09g021130

MARVSLGICLMLVVASSVIYEAEGTFLLKLYIRKNFPKQCNEFTPYANKGMLSFVTNLEGNCPATAEFKNFFTQFKSYMSFIETASASSKNVDTEMTTKCNGLFKAMSAISSGKGEKSADAGGLKATMLAMGKTLVEQKKNTKIMTLKQKKELIVSMVKWTNMLATFVKSASEKKGKSIDIGSYGLDVDVNDSSIVGNTESSSASSTKAGSVSTKTKESSSGGSSAASKTKESSGGSSSGANKDTTGKDSGSPSETPTAKPSDSESAGGKSALKGSESATEDANAKGSISGKSSYKSSKTSSERRSKSSSQSSYKSSSSISVKQVESETSKEVMSFITQLEKKYSAKTELKVFFEKLKASMQASSSIASKTSKDYVSATAAATGKLSEAMALVGSKNVKSAKMKSNMETSKDEMMKCLKQIQDINSKIVSGKTASSTQQSELKQTITKWEKVTTQFVETAASSSSSSSSSSSASQQQGSARMVENN

>Csa09g021140

MARISLALGLLLLVALSEVYEVQGTFLLRHYLRKLPRRNRDFRPFACKGMLRFVALLQSMCPLKPQYTSFFGNLKSYMNFINSASGSENYDSELKGKAQGLYSSISALSGKGGASADSSKVMDTLMSMGKTLGHQQESSSTMSLGERKELILSMAKWAQTIGQFVVSAAAQSGKKIDISSLGLDIDASATATGDSTTTTENSTTTGTGTTAGGTTTTGTGTTTVGSPTSSCTTSKCSNGGGSSFKAAVNVQHGGKASSQSQQNTSTQQDATAAGSS

>Csa09g021430

MARVQLWLCITILFATVSLLDLASAHLKYKPSLPQLEDPKTVKDVEPYTVKVVMVFVADLEKECPKNNKFKTFFEKLRGFAKYVCPIKRKDQFMKSVAAKIMGGRRQEESEDTMKLTAEQQKEIKEGILKWETIITRITNTMVMSSSNSSSSEESNVENEDSSSNSKSSGKESSTSAKGSSEETKDSKSNSGSPSGTGSPSSSPSDASGKETSTKDTKTKGESSSDESGKETNSKDTKIKGESSSDATGKETNSKDTKTKGEISSDASGKETSTKDAKATGEISASETTETSQSSTVNVEQVEAETSKQVSSFIMNLEKKCPQKEEYKAFFEQLKGTMIAPVKEGKDLFTRIKSAAGKVSGAMAFIRSRVESKSAEVKQSMETYQGEVMKTLQELETIHSQIVSQNKGKTEGSLTCTPAQQMQIKQTITKWEQVTTQFVETASQSETKTSSSASSSASGKMVAN

>Csa10g047890

MEQKNQVSTYKTEAMNSVKLLQSIGEKISSGRNNKPEKLTVKQQKEIKDGILKWIQVITRIAKTGEEINSKASSKSQTRQDSKGEEESTTQTQTHTKRRSQREKAQITALPRGSRVTK

>Csa11g055950

MPRFQLLFRFTILAATITFFNVASAHVKIKPVLPQIQDPMTVKDVESYTIKVVTNFLADLEKECPKTEKFKAFFEKLKAYSKYVCPVSKARGYESDMKAKAGSLFEAISALSSVENRSRGGRVNMSLQREKTEAMNTVKLLQSIGEKISSGRNNKPEKLTVEQQKEIKDGILKWIQVITRIAKTGEEINSKASSKSQTRQDSKGEEESSTQTQTHTKRRSQREKAQITALPRGSRVTKVGRNNDISKRRKPKRYTNIRERQQ

>Csa11g097570

MARISLAICLTLLVTLSTVYETQGTFSLPHYLEKFPKVGKDFEAFANKGMSDFLGDLEGMCPKTAEFKNLFATLKDYMASFGSGSSKDIKMELSEKSEKLFRAMSVFDTSKGGTSDDSWNLVDGLLSMGKGLMEMKKSGSQELNFEQRKELIVSMVEWTRGIGLFVKAASESKGQSIDLSSFGIDYDNSVESPIKRAMYETQGTFSLPHYLENLPKKAQDFEPFAYNGMSHFIDSLESKCPATTEFKDFFVKLEDYMAIFKSASSGSKDFKVDMSIKSQRLFKAMSVLTGTQGSGISVDSWRMLDGLLSMGKFLVEMKKHGSNEITFEQRTEMVGSMVQWARAIGLFARVASEKKGKTIDLSPFGIFHARNYGKGNFKTSSEL

>Csa12g081480

MPRFQLLFSFTILAATITFFNVASAHVKIKPVLPQIQDPTTVKDVESYTIKVVTNFLADLEKECPKTEKFKAFFEKLKAYSKYVCPVSKARGYESDMKAKAGSLFEAISALSSVENRSRGGQVNMSLQREKTEAMNTVKLLQSIGEKISSGRNNKPEKLTVEQQKEIKDGILKWIQVITRIAKTGEEINSEASSKSQTRQDNKGEEESSTQTQTHTKRRRQREKAQITALPRGSRVTK

>Csa14272s010

MKLSAEQQKEIKEGILRWETIIARITNTMITSTTDSSSSETSTTEKGGSSNKSKSSGSESSGSEGKGSSEETKDSKSNSGSPSGSPSSS

>Csa18g035100

MARISLAICLTLLVTLSTVYETQGTFSLPHYLEKFPKVGKDFEAFANKGMSDFLGDLEGMCPKTAEFKNLFATLKDYMASFGSGSSKDIKMELSEKSEKLFRAMSVFDTSKGGTSDDSWNLVDGLLSMGKGLMEMKKSGSQELNFEQRKELIVSMVEWTRGIGLFVKAASESKGQSIDLSSFGIDYDNSVESPIKRAMYETQGTFSLPHYLENLPKKAQDFEPFAYNGMSHFIDSLESKCPATTEFKDFFVKLEDYMAIFKSASSGSKDFKVDMSIKSQRLFKAMSVLTGTQGSGISVDSWRMLDGLLSMGKFLVEMKKHGSNEITFEQRTEMVGSMVQWARAIGLFARVASEKKGKTIDLSPFGIFHARNYGKGNFKTSSEL

>Cagra.0535s0009

MRKVSLFFFCCIVATNLISLNVVSSHEMVDVTAPNMVKDIEPYISNRALGFVLKLENNCPIKEQLGSFFDKLKDLLKLESSVTPLIENNEPKTFEFDLKSKSETLLQTMFNLGKGLLSSSVRKEMFEVMKSLTQLHAAIGRVIVEKHISGDDESMSLSAEQKNAVESSITEWEQTISRIVKIVVEVKSKGSTDVSLEENSNSGHNNVSMDSNMANTNGEDSETSQGKEDGIQGSFGGNVSMETQEGNKGEDDKEGNLVSENSGIKDIKGENGDSNIEAKVEGQGGNLGDSRVGKNLETNEDVKSGIEAKNVGSSMNENLEEANKTNGVSTMDMNLENKGSDEEGKDDNMVNATTNDEDHMKEKREETQENNGELAKDENLENNIGNKELKGNGSIESKTNNENSMEEKREEIQRSHEISMEHKREETQRSHEISMEQKREETQRSHEISMEQKREEAQRSHEISMNKETTKGENTDLNKENEKNVQGESTREMNKGENANKTGKIIKDKNALNKEIEDTQSKEKHKDKKEALMKKNEGVQEEANVTSNISGKKDQNESTHDTSKDINKNDEFLRKQKEVVEGHVNIGDSDKKASEKKIDDTKSKEKHKDKEEASMKKSEGVQEANTTSNISGKKDQNESTDDTSKIINKNETLKKQKQVDEGQVNIGDSDKNTKGETKENNGDLVNGTSIEVSGGSKESEDGKNNETNQTKNNSMKEVSSDGKKVETKGGKEGSIEESSKDNIGGKEENSKDDKKVEHNEAKRDFKEGGSKDGNATKINEGYKDDSKNKDSKESSEEESLKDAKIVETSGSKNHTEIGSKDDNTTNINGGKKDLKDGKLVETSKGTNSTNESSRDGNTMENSGVKLDSMEKNSSDSNIAETKGGKEDSIKESSEVGKRNETNGSKDVSVQENSKEGNVEEVQKGQEDSTKEGFKEGKEDQGNGGKEVTMEEGSKDDKQIEIGKEDLTDKLSKNGTTTNLNGGKNSTENASEDSKTVETNGAKEDSENGNIHETSNSTISGTNGQKEESIKESSKDGKGTETNGDKDVSMKENSKDGNIEVNKGQEESTKKGFTEGKVDETNGDKEVNMEEGSKDDKTVDINGGKEDSTEEKTKDGMTTSLNGSTNSTENGSEDSKTVETNGVKEDSANGNINEINGTSMESKGDKVDMNEDSMKNKTMEAQESSNGDSMNGKVEEEDKENKDVMNNQNLQDGGSNTTFMNNQTTGNGTTSTTKQEIRTNIETSTSKEVTSFITSLEKKSPDTQEFQSFFEKLKDYMKYLCPDSSTFEAKDSSSYMSEMISMATKLSDAMAVLQAKKSGSGEMKTTLQGYQQEVMKTLNILQSVMSKAISEKQSTEGGSLTLTVSQQQAIKEIVLKWEQVMSQFVKIATESEKQFSVEISSGNGYHMKKSSNSSSSSSSISSSSTHSNMDLKVDGETLKGLDMNV

>Cagra.22421s0001

TPSVDALTKKESNSQTGTSSNTKETNTGSSGSTLTDTTGSTSGSPSGNPTPTPSSSTPASGKPSEKESPSTTLGSPSGSTTPTPSSSTPADGKTPTQKKEESYSGIASGEGSEEESNSGGATSQKKESTSQSSSVKELESQTSSEVMSFISNLEKKHKDNAELKVFFDKLKTSMSASSKLSGSNTKDIVSGMKSATSKLSEAMMFVRSRFSKSEETKSSMESSQQEVLKTLKELQDINSQIVSGKKVTSTQQTELKQTITKWEQVTTQFVETAASSSSSSSSSQTSAKMAQQN

>Cagra.3344s0003

MARISLVLGLLLLVALSEVYEVQGTFLLRHYLRKMSRRNRDFRPFACRGMLKFVNLLELRCPLKPRYKSFFGNLRSYVKFINSASGSKNYDSELKGKAQGLLSAISSMNGKSGSSDDSNKVMETLLSMGKTLGHQQESTTTMSLTQRKELIMSMAQWAKTIGQFVVTTAAKSGSKIDMSSIGLDGIDSSETIESQDSTTSTENTSTGGTTKTQGTGTTTGGDSTTTGSAPSEDTPTTDTPTTDSPTSGCTSPKNDCPNGGTTIKGSVNFQHSGKATQSRQTISSQTQQGVTTAGSN

>Cagra.3344s0004

MSFIETASTSTKNIDVEMTSKCDGLFKAMSAMSSGKGTKSADAGGLKATMLSMGKTLVEQKKNTKIMTLKQKKELIISMVKWTKMLATFVKSASEKKGKTINIASYGLDVDVNDSSIVGTTESRTSSSTKAGAVSTGSSGSVSTKTKVSSNGGSSGGANKDTKAKNSGSPSGSPKAKPSDSGSAGGKATLKGSASAKENTNTAGGLSGKSSFSTNTASQRSSERKSQSSRQSSFKSSYKISVKQVESETSKEVMSFIMQLEKKYSARAEFKIFFEKLKASMQASSSIASKTSKDYVSATSAATSKLTEAMALVGSKNVKSAKMKSNMETSKDEMIKCLKQIQDINSKILSGKTVSSTQQSELKQTITKWEKVTTQFVETAASSSSSSSSSSSASQQQGSARMIKTN

>Cagra.4554s0002

MARVQLLVCFTILFASVTLLDLASAHLKFKPSLPQLEDPKTVKDVEPYTVKVMMVFVADLEKECPKTSKFKAFFGKLRAFAKYVCPIKRRDQVDYDRDMKAKAGGIFKTISSFAIGKIREEIQEEKQEAIDTFRFMKSVAAKIMGGRRKDESEDTMKLTVEQQKEIKEGILKWETVITKITNTMVMSSSNSSSSVSSNVEKDESTNKNNKSSSEETKVSKSNSGTPSTGTDSKDTKPTEEKSPAANAKEPSSKDAKPAEESSSPAASGKETSSKETSSKETKATGEKSSSGSVETSQSSSGVNVEEVEAETSKQVSVFIMNLEKKCPQKEEYKAFFEQLKGTMIAPPKERKGLFSRLKTAAGKVSGAMAFIRSRIGNKSAEVKKSMEAYQGEVMKTLQELETIHSQIVSQNKGKKSLTCTPAQQVQIKQTITKWEQVTTQFVEVATKSETQSSSSSTSSSKKMVAN

>Cagra.5499s0001

MARFPIAGCLMLILVASSTIYEAQGTFLLRHYMRKFPKLSQDFEPFAYKGMLSFVDNLESMCPLNGEYKDFFSKLKSFMSFINTAQGSSSDFQSQMKSQSESLFKAVSALGVKAGSSADTSKLIESLMSMGQTFSEFKRSGSTMMTNEQRTELVKSMAKWAQVIGQFVKNVGDKSGDGNIDLASILGGGSPLDGSGSPSSDTSSPSGDAGTPTDGGSYGDSTGDSGSSAGGPSGSVTDLGDGSTAGGPSGSTTDGSSGGDSSMGAGSGAAGDTGSTSGGDAAGGDSAAGGAAPGGDAPGGAAPGGESGGAAAGGESSGAATGAESAGATGAESATGGDSSGAAAGGDTSGAAA

>Cagra.5905s0009

MPKFQLLLCFTILVATITFFNVASAHVTIKPVLPQIQDPTTVKDVESYTIKVVTNFLVDLEKECPKTEKFKVFFETFKAYSKYVCPVSKARGYESDMKAKASSLFKAMSALNSVENRSREGQVNMSLDREKVEVMKTVKLLQSIGEKIASGENNKLTVEQQKEIKDGILKWIQVVTRIAKIGEEIDSKDSSKPQTRQDGSNEEKSSTQTQTKRRSLRARAQITALPRGSRRTKVGNKDDTLRRRKSKRHTNIKSSKNNT

>Carub.0005s0732

MRKVSLFLFCCIVATYLLSLNVVSSHEMVDVTAPNMVKDIEPYISNRALGFVLKLEKNCPIKEQLGSFFDKLKDLLKLESSVTPLIENNEPKTFEFDLKSKSETLLQTMFNLGKGLLSSSVRKEMFEVMKSLTQLHAAIGRVIVEKHISGDDESMSLSAEQKNAVESSITEWEQTISRIVKIVVEVKSKGSTDVSLEENSNSGHNNVSMDSNMANTNGEDSETSQGKEDGIQGSFGGNVSMETQEGNKGEDDKVGNLVSENSGIKDIKGENGDSNIEAEVEGQGGNLGDSSVGKNLETNEDVKSGIEAKNVGSSMNENLEEANKTNGVSTMDMNLENKGSDEEGKDDNMVNATTNDEDHMKEKREETQENNGELAKDENLENNIGNKELKGNGSIESKTNNENSMEEKREEIQRSHEISMEHKREETQRSHEISMEQKREETQRSHEISMNKETTKGENTDLNKENEKNVQGESTREMNKGENANVNKENESKVQGESTRDMTKGENANVNKENESKVQGESTREMTKGENANVNKENESKVQGESTRDLTKGENTDFNKDNEIKVQGENTGDLTNGTISNNNEDVKSEVDSNGSVGSSTKEKNQEAQGNDIVSTKDKNLENSGVDKETKHDKSVEVTTNDGGHTKEKRKRNGESMKNENLENIEHQKELKNDGLVTAKTNNETSKEEKGEQMQRGHDISINDKKMENKSGNAYVNKENEAQVGDSTSDNNMESKDDAKLEVDFKKNGGASKKEKREEVQGSNESSLENKNLETQKDQTDLKEDSSIEDKRNVRSSTKENQEEAQRNGRDKKEVNDDKSTEAKRKRKESKSTKNSENKVNEIENKESKKVKEGEKKESKDAKLVEAKENKEISMKEKREKSQGSTSEGLNKVDNKEEKKESKDYQSRKNKDNTESSVKEQQQKAQENGGVHTMIGNKQDNNDLKDGKSVEAKTNKESIMKKDREQAQRNDIDTMLKKTTKEDKSSADTTDANSKEKGKDKHENKEFRGSHLKKQKEDKKEKKESGDSKSKNKEKNRYKDHKVKIKDESKTKNTNSKEYELKKKEEDKKEKINSRDKKKENKDSEHRKSKEKKEEKQEKKEFRDHKDNKKETKKKEKKESEHHKSKKKEKENNKYDDTRKEKNEDKRVMKKVENKKEKKEHEDKKSMMKEKDKKMEESKEAQSKKFKKEKKEFEDNKLKKNEDEKKETKEHEDMKSKKRKEDKKVNKKSQDKKKKKENKDSSSKINVADKNSESKKIEEDKKEKTISKDIQQKNKEQEKKHSNDKITKKYEEGRKKKAMSKEKNKEQEKKQSNEKITKKNEEDRKKKAMSKEKNKEQEKKQSNDKITKKYEEGRKKKAMSKEKNKEQEKKQSNEKITKKNEEDRKKKAMSKENKSKTKGENKKPKENEDKKKESKEDNSKQGGVQATTQIEGGEGKNEMKTDAQVKREESLKDKKGDNKEDHDEKVDKENDEHYKKKTGKIIKDKDASDKEIEDTKSEEKHKDKKEASMKKNEGVQEANVTSNISGKKDQNESTHDTSKDINKNDEFLRKQKQVDEGHVNIGDSDKKASEKKIDDTKSKEKHKDKEEASMKKSEGVQEANTTSNISGKKDQNESTDDTSKNINKNDETLKKQKQVDEGQVNIGDSDKNTKGETKENNGDLVNGTSIEVSGGSKESEDGKNNETNQTKNNSMKEVSSDGKKVETKGGKEGSIEESSKDNIGGKEENSKDDKKVEHNEAKRDFKEGGSKDGNATKINEGYKEDSKNKDSKESSEEESLKDAKIVETSGSKNRTEKGSKDDNTTNINGGKNDLKDGKLVETSKGTNSTNESSRDGNTMENSGVKLDSMEKNSSDSNIAETNGGKEDSVKESSEVGKRNETNGSKDVSVQENSKEGNVEEVQKGQEDSTKEGFKEGKEDQGNGGKEVTMEESSKDDKQIEIGKEDLTDKISKNGTTTNLNGGKISTENASEDSKTVETNGAKEDSENGNIHETSNSTIAGTNGQKEESIKESSKDGKGTETNGDKDVSMKENSKDGNIEVNKGQEESTKKGFKEGKVDETNGDKEVNMEEGSKDDKTVDINGGKEDSTEEKTKDGMTTSLNGSTNSTENGSEDSKTVETNGVKEDSANGNINEINGTSMESKGDKVDMNEDSMKNKTMEAQESSNGDLKNGKVEEENKESKDVMNNQNLQDGGSNTTFMNNQTTGNGTTSTTKQEIRTNIETSTSKEVTSFITSLEKKSPDTQEFQSFFEKLKDYMKYLCPDSSTFEAKDSSSYMSEMISMATKLSDAMAVLQAKKSGSGEMKTTLQGYQQEVMKTLNILQSVMSKAISEKQSTEGGSLTLTVSQQQAIKEIVLKWEQVMSQFVKIATESEKQFSVEISSGNGYHMKKSSNSSSSSSSISSSSTHSNMDLKVDGETLKGLDMNV

>Carub.0005s0733

MARFPIAGCLMLILVASSTIYEAQGTFLLRHYMRKFPKLSQDFEPFAYKGMLSFVDNLESMCPLNGEYKDFFSKLKSFMSFINTAQGSSSDFQSQMKSQSESLFKAVSALGVKAGSSADTSKLIESLMSMGQTFSEFKRSGSTMMTNEQRTELVKSMAKWAQVIGQFVKNVGDKSGDGNIDLASILGGGSPLDGSGSPSSDTSSPSGDAGTPTDGGSYGDSTGDSGSSAGGPSGSVTDLGDGSTAGGPSGSTTDGSSGGDSSMGAGSGAAGDTGSTSGGDAAGGDSAAGGAAPGGESGGAAAGGESSGAATGAESAGATGAESATGGDSSGAAAGGDTSGAAAGGDSSGAAAGGDTSGAAAGGDSSGAAAGGDTSGASAGGDSSGATAGGAAAGGESGGAAAGGGDAASGASGESSGDASGAAAGGASGAASGDTSGASAGGASGAASGDASGAAAGGASGATSGDASSGDASSAASGDASAASGDASGAAAGGTSGAASGDASGAASGGASGASSGDASGAAAGDTSGAASGGASDAASGSSSGAGAAGATQTTETGEASSGESSSAASGSTETGSSMTGGAYTDSTGGSPASSPSAGGPSGSETSMEGAAAGETSGASAGSVSYQAANYEKKHSTSSGKSSFSHASIKKSSGTANADS

>Carub.0005s0734

MTKKLLVLCLVLMFASSVVYEVQGTFLLKLYLRRKFFPRRCIDFTPFAAKGMLMLIKNLQGGCPATREFKEFFTRFKSYISFISSVSSSSKNVDTEMDGRCELLTKALSVLTGSKSSQTSELKSTMLSMGKTLVEQKRQGSKIMTLKQRKELVVAMVKWTRLVITIVKSVAEQAGKSIDESSYGLDVDVNSSVRKRTDNDDDDDDDDDDDDDDDKETSPKGRSPPQNESPPQSESPPQGGSSPQSESSPQGGSPPQGESPPQGGSSPQSEYPPQKGSPPQGGSSPQNEYPPQGGSPPQSESPPQSGSPPQSESPPQGGSPPQGGSPPQGGSPPQSGSPPQGESPPQGGSLPQGGSPPQGGSPPQGGSPPQGGSPPQGGSPPQGGSPPQSGSPPLDGSLPQGGYVTPSVDALTKKESNSQTGTSSNTKETNTGSSGSTLTDTTGSTSGSPSGNPTPTPSSSTPASGKPSEKESPSTTLGSPSGSTTPTPSSSTPADGKTPTQKKEESYSGIASGEGSEEESNSGGATSQKKESTSQSSSVKELESQTSSEVMSFISNLEKKHKDNAELKVFFDKLKTSMSASSKLSGSNTKDIVSGMKSATSKLSEAMMFVRSRFSKSEETKSSMESSQQEVLKTLKELQDINSQIVSGKKVTSTQQTKLKQTITKWEQVTTQFVETAASSSSSSSSSQTSAKMAQQN

>Carub.0005s0735

MARVQLFLCFTILFASVTLLDVVSAHLKLKPSLPDQIEAPKTVKDVEPYTVKVVMVFVSDLEKECPKTSKFKAFFAKLRAYAKYVCPIKRKDQVDYDRDMKAKAGGIFQAISSFAIGRIKKEIQEEKQEVIDTFKFMRFVAAKILGSRKKDENEDTMKLTAEQQKEIKEGIIRWETIITRITNTMVMSTKNSSSSETSTTEKGDSSEKSKSSSKDTNSGSPSTGSPSGPKSSDSSEKEAKSKDTKTTGEKSSKDAKNTEDNSSQDTKNTEENSSKDTKNTKENSSKDAKNTEENSSTTSGKETSSKDTKKSAGESSVSQSSAVNVEEVEAETSKQVSTFIMNLEKKCPQKEEYKAFFEQLKGTMVAPPKERKGLFSRLKSAAGKVSGAMAFIRSRIGSKSAEVKKSMEAYQGEVMKTLQELETIHSQIVSQNKGKKEGSLTCTPAQQTQIKQTITKWEQVTTQFVETAVQSETQSSSSTSSSVGKMTSN

>Carub.0005s0736

MARVQLFLCFTILFASVTLLDVVSAHLKLKPSLPQIEAPKTVKDVEPYTVKVVMVFVSDLEKECPKTSKFKAFFAKLRAYAKYVCPIKRKDQVDYDRDMKAKAGGIFQAISSFAIGRIKKEIQEEKQEVIDTFKFMRFVAAKILGSRKKDENEDTMKLTAEQQKEIKEGIIRWETIITRITNTMVMSTKNSSSSETSTTEKGDSSEKSKSSSKDTNSGSPSTGSPSGPKSSDSSEKEAKSKDTKTTGEKSSKDXXXXXXXXXXXXXXXXXXXXXXXXXXXXXXXXXXXXXXXXXXXXXXXXXXXXXXXXXXXXXXXXXXXXXXXXEVEAETSKQVSTFIMNLEKKCPQKEEYKAFFEQLKGTMVAPPKERKGLFSRLKSAAGKVSGAMAFIRSRIGSKSAEVKKSMEAYQGEVMKTLQELETIHSQIVSQNKGKKEGSLTCTPAQQTQIKQTITKWEQVTTQFVETAVQSETQSSSSTSSSVGKMTSN

>Carub.0005s0737

MAKISLGICLMLVIASSVIYEAQGTFLLKLYLKKNFPRKCNEFTPFANKGMLTFVTNLEDNCPATAEFKNFFTQFKSYMSFIETASTSTKNIDAEMTSKCDGLFKAMSAMSSGKGTKSADAGGLKATMLSMGKTLVEQKKNTKIMTLKQKKELIISMVKWTKMLATFVKSASEKKGKTINIASYGLDVDVNDSSIVGTTESRTSSSTKAGAVSTGSSGSVSTKTKVSSNGGSSGGANKDTKAKNSGSPSGSPKAKPSDSGSAGGKATLKGSASAKENTNAAGGLSGKSSFSTNTASQRSSERKSQSSRQSSFKSSYKISVKQVESETSKEVMSFIMQLEKKYSARAEFKIFFEKLKASMQASSSIASKTSKDYVSATSAATSKLTEAMALVGSKNVKSAKMKSNMETSKDEMIKCLKQIQDINSKILSGKTVSSTQQSELKQTITKWEKVTTQFVETAASSSSSSSSSSSASQQQGSARMIKTN

>Carub.0005s0738

MARISLVLGLLLLVALSEVYEVQGTFLLRHYLRKMSRRNRDFRPFACRGMLKFVNLLELRCPLKPRYKSFFGNLRSYVKFINSASGSKNYDSELKGKAQGLLSAISSMNGKSGSSDDSNKVMETLLSMGKTLGHQQESTTTMSLTQRKELIMSMAQWAKTIGQFVVTTAAKSGSKIDMSSIGLDGIDSSETTESQDSTTSTENTSTGGTTKTQGTSTTTGGDSTTTGSAPSEDTPTTDTPTTDSPTSGCTSPKNDCPNGGTTIKGSVNFQHSGKATQSRQTISSQTQQGVTTAGSN

>Carub.0005s0757

MARVQLLVCFTILFASVTLLDLASAHLKFKPSLPQLEDPKTVKDVEPYTVKVMMVFVADLEKECPKTSKFKAFFGKLRAFAKYVCPIKRRDQVDYDRDMKAKAGGIFKTISSFAIGKIREEIQEEKQEAIDTFRFMKSVAAKIMGGRRKDESEDTMKLTVEQQKEIKEGILKWETVITKITNTMVMSSSNSSSSVSSNVEKDETTNKNNKSSSEETKVSKSNSGSPSTGTDSKDTKPTEEKSPAANAKEPSSKDAKPTEESSSPAASGKETSSKETSSKETKATGEKSASGSVETSQSSSGVNVEEVEAETSKQVSVFIMNLEKKCPQKEEYKAFFEQLKGTMIAPPKERKGLFSRLKSAAGKVSGAMAFIRSRIGNKSAEVKKSMEAYQGEVMKTLQELETIHSQIVSQNKGKKSLTCTPAQQVQIQQTITKWEQVTTQFVEVATKSETQSSSSSTSSSKKMVAN

>Carub.0007s3585

MPKFQLLLCFTILAVTITFFNVASAHVTIKPVLPQIQDPTTVKDVEPYTIKVVTNFLVDLEKECPKTEKFKVFFETFKAYSKYVCPVSKARGYESDMKAKASSLFKAMSALNSVENRSRGGQVNMSLDREKVEVMKTVKLLQSIGEKIASGRNNKLTVEQQKEIKDGILKWIQVVTRIAKIGEEIDSKDSSKPQTSQDGSNEEKSSTQTQTKRRSLRARAQITALPRGSRRTKVGNKDDTLRRRKSKRHTNIKSSKNNT

>CARHR151780

MAKFPLAICLVFMLVASSTIYEAQGTFLLQHYMRKFPKMSQDFEPFAYKGMISFVDNLQNMCPMKGEYQEFFSQLKTFMSFINTASGPSSEFQSQMKSQCENLFKAISALGVKGGSSADTSKLIDNLMSMGKTFNEFKRSGSTTMTSEQRREMVTSMAKWAQGIGQFVENVNEKSGDGANIDLSSLIGSVNGGSSGFGTSSSPTGNGNTGDSPSSTGESGSPAESGSSSSNSGSPSVSPTDSGSPTDTDTGSPADSGNPSSSSGSPFGSPADSGSPTDSGSPADNGSPADSGILTHSGSPADSGRPADSGSPADSGSPADSGSPNSSSGSPSGSPSNSGSPADSGSPAESGSPTDNGSSNSSSGSPSGSPADSGSPTESGSPSSNSGSPSGSPSDIGSPTESGSPSSSSGSPSGSPWDSGNPSDSGTPSSTSDFPSGSPTDNESPSDTGSPSDSGSLSSGSGSPSAESPSSGSGSPSDDSGSPADSGNSNDNRTYNDSTGDSGSPSPDSGSPSAETGSPSGSPDSGSPSAETGSPSGSPDSGSPSTETGSPSESPTDSSKGEDSSSNESAGGRETTSSEGAAGGQIKSVEGGENSGSGGAAGGESSAGGGQTTSSKSSVKGTTNASLSKQEMFLKKKTEEDRLMARGGEYSDSTGGGGPASSTTVVSEASSCKGSAEEVSGGSGASGGQSCKGKGGSVGYQGAASYHKRHSISSEKASYHHISQQKTSGNANVNS

>CARHR151800

KKSMENYQGEVVKCMEELDAIYSKIVTQNKGKSEGSLTCTAEQQKEIQVTITKWEQVTTQFVETAVQTETQTSSSSTSSSSSIGKQIQEN

>CARHR151820

MARISLGICLMFLITSSVIYEAQGHFLLKHFLRKRFPENTNEFTPFANKGMLMFVTDLESNCPPTTEFKTFFTQYKSYMTFIESASTSSKNVDAEMTKKCDGLFKAMSALSAGKGAKSADAGSMKTTILSMGKTLLALKKKAVVLTLTQKKELVLSMVKWTRMLATFLKSASEKKGKTINIASYGLDVDVNDKSIIGESTETTSSTKTESTTKDANSESPKGKVSDAMAFIRTRIAGKSAEAKKSMENYQGEVVKCMEELDAIYSKIVTQNKGKSEGSLTCTAEQQKEIQVTITKWEQVTTQFVETAVQTETQTSSSSTSSSSSIGKQ

>CARHR152080

MAKIQLLVCFTILFASVTLLDVVSAHIKIKQTLPEIEDPKTVKDVEPYTVKVVMVFVSDLEKECPKTNKFKTFVEKLRGYAKYVCPIKNKDEADYDNDMKAKAGGLLKTISSFAVGKIREEIQEEKQEAINTFKFLRFVAAKIVGGGKNDENKESMTLTSEQQKEIKEGILRWQTIMTKITNTMVVSTSSSSPKEDSTSGGESPNGKSEKSGGSSFKDTTGSGSNMGAPSGSTSDSPSGSPSTSFKDTTGGVMISPSGSMTIPSGSPSNSPSGSDSISPTQSGSDEASQNTTMTVTEVEAETTKEVMTFIMSLEKKCPQKEEYKSFFEKLKSVMTAPPKAAAKGIKGFLSGVKSAGGKLSETMTFVRARIGSKSAEVKKSLEDYQGEVMKTIQELEAIHSQIVKQNKDKKDDGALIVTTEQRTEIKKTITKWEQVTTQFIETVIQSETQTSSSPTLGNAIGKFDVQTSA

>CARHR262310

MPRFQLLFCFAILVVTINLFNLATAHVKIKPALPQIEDPKTVKDVESYTIKVVMSFLVNLEKECPKTEKFKIFFEKLKAYSKYVCPVSKARGYESDMKAKAGSLFEAMSALSSCKNRSKGGLVNKSLQREQIEAMNTMKLLQSIGEKIAGGRNKKQEINVTMKLTVEQQKEIKYGILKWLHVITRIAKTTEEIISKSSSKFQTMRERNEDKSLTKTKTKSRQRDSQKARIHVLPRGSRESKKNTNIKGESRRCKTVVKSKNA

>Thhalv10009470m

MVIISDLEKECPKTNIFKFFFEKLRTYAKYVCPVKKNDQKDYDRDMKAKPYPLLLLEGQYSNHKNKGVQSTTTNSSSCEDSTSGKRSSSNRSSSGSSFKDTTGSSSYFGSPKGTGTSFTDTIGGNSISPNESPTKQPIFKLHELCFKESSVAVSQNSAIKVMEIEAKTSIESYDIRYLDKKCPQKEEYKVFFEKLKGTMTGSSKVSNENKKGFFSRKISAAGKLSDAMAFMRSRIGSKSADVLKNQGKKGGSLTCKPRHTKKRDLSRLSPTQFVETAIQSESSSTSSSSSFSSNLQGKLKVKAN

>Thhalv10004155m

MARIQLLVCFTILLASVTLLDVVSAHLKLKPALPQIEDPKTVKDVEPYTVKVVMVFVSDLEKECPKTNKFKAYFEKLRAFAKYVCPIAKKDQKDYDRDMKAKAGSLFQAISSFAIGKIKEEIMEEKLEAVNTFKWMKSLAGKIMGARKKEESEESMKLTAEQQKEIKEGILKWETVIARITNTMLQSTGSSSASGGDSTSGKETSSGSGKSSGSGSSSSGKGSSSSGSGTSYKDTTGSSSDMGSSSGSPATSPSGSTDIASGGSGTSYADTTGVSSISPAGSPANSPAGSTTSNSQGSTVAEVEAETSKEVMQFIMNLEKKCPPKEEFKSFFEKLKGTMMASAKVTSEKKNDFFSGIKSAAGKVSDAMSFMGSRLGSKSSEVKKSMESYQGEVTKALHELDAIHKKILSQSQGKKDGTVTVTETQRTEIKQTITKWETVTTQFVETAMKSESTTNSPAGIDKIKLN

>Thhalv10003856m

MAKNILAICLMFLVASSVIYEAQGSFLLKMYLKRKLFPRRCVDFTPYAYKGMMLLVGNLEGGCPATREFKQFFSTFKSYMAYINSASASSKSVDVELTTKCDLLAKAMSALSGAQSVSSDFKLTMLSMGKTLIEQKRKGTVIMTLQQKKELVKSMVSWTRMLLTFVKTASEKRGKSIDVASYGVDVDVNSIVGSTESDSSSANTGSRNSDSSSTGSGSTSSGSATSSRSGSSSSSGNAAYTGSGSTYKDTTGGGSGSPSGSPTGSPSSSTSATDETSGSTSGSGSASGSENASGSGSTSGSGSASGSGSGSASYKTKQSASGSSASGSSKSGSVNSYSDATGATSGSPSGSPTGSPSGSTSASGENSVKGSASGSESASGSGSASGSGSASGSGSASGSGSASSKTSSESKGTSSSASSSSMSAKQVESETSKEVMSFISGLEKKYAGKAELKVFFDKLKTSMSASSRLSATDAKDFVSGMRSAAGKLSEAMAFVRSRFSKSEEMKSTMETYQEQVMKTLKELQDINSKIVSQSQGKTVTSTQKTELKQTITRWEQVTTQFVETAASSSSSSSSSSSSSSSQQKGSMNAQSSMKSQSLMNAQAS

>Thhalv10003975m

MARISLGICLMMLVVASSVIYEAQGSFLLKHYLSKNFPRNCNEFTPYAKTGMLTFVTNLESSCPATAEFKEFFAKFKAYMNFIATAPATSKNIDVEITTQCEGLFKAMSALSGAAAGKSGDAESFKVTMISMGKTLMEQKKNAMMMTLAQKKRLVMSMVRWTKMIATFVKTASEKKGKTIDLKSYELDVDVNDKSILGASASASGSTETKAGAASGGSTEKSSSSTKTGSSASTKTGSGSTYKDTTGGYSASPSGSPTSSPSGSGKTFSKASAGASGSASSTKKESSTGSAGDSSTKKESTAGSAGDSSTKKESSTGSASSSSSTKKESSTGSASSSSSTKKESTTGSTSVKQVETETSKEVMSFIMELEKKYSGKAELKVFFEYLKASMAASATISTKTAKDYIALTRSATGKLSQAMATMGSRAASKSAKMKSNIETAQEKMIRTIKELQDLNSKIVSEKKVTATQQSELKQTLTKWEQVTTQFVETCASSSSSSSSSSSSSSSSQKSSGSSQESSASSQQGRARMVKID

>Thhalv10005404m

MKRVRLLFLCCITILATSLSQNLVSAHEMDVSSPNTIKDIEPYISNRAIGFVVKLENNCPIREQLRSFFDKLKDLLKLESSVTPLIENNEPKTFKFDLKSKADTLFQTMITLGRGLLSSSVRKEMFQVMKSLTELHAAISRVILEKHIKGDGSMSLSLDQKNAVENAVSQWEVTITRIVKIVVEIKSGGSSVASGEESSTTEQNSASMNGTMVQTNGGNSESMQENLDGVKGSNVDSRNDVEGGSESENVDSNNEKQVEVQGRSIGNSTIEKNLESKEDANSEVEAKNEGVSTMDSNLENKGSGEESSKDADDTKEKREGSQENNEESAKDENLMKNEDNKELKNVGSVETKTNHEVSMEEKREHTQGSTEVSMNGETMDTKGGNADSNEEKKNDFQGVSTGNSTEDKNLENKEDVKSEVKDDENEKNLAKEKQEEAQVNDGNSTKDQNMENAHEETKDGQSVEAMENNGDHMKENTEEVQGNNGESVNDDNVENKQELKENGSVEARTHNETSMEEKKQGAQGEERNVDSNKENGADVQGEHSENSSKDTNLDSKEEAKSEVEARENGGSSIKEKGEDQAQENGGGGEKELKDNKSVETQQNADFSTGEKKEEGQGNNGESTKVETKGGKNESKDDKSSKDKEKARSSMKEKGEKAQRNSGESKDDNSVKAKENEGSSKEENQEKVQGNKKESKKVDNKGYKKELKDDKSLQDQDNKESFMKENREETQRSNNGDHSKIDDKEGKKDSKDKKSFETKENNESSMKGKQQESQGKGGEYTKVENKEGYQQEVMKTLTILQSVMSKAVSEQQSQNDGSLTLTSSQQQAIKDIILKWEQVMSQFVKVATESEKQFSMETSTGHSYHIKSSTSSSTSTSSSNSGPGLDFKPNDESSKMVDDLM

>Thhalv10004661m

MARTSSVCLLLLVALSAVYEVQGTFLLRHYLRKIPRRSGDIQPFACQGMLKFVDVLELKCPLKPEYKSFFGKLRSYMSFISSASGSKNFDANLKGQAQGLQSAMSALGGKGASSADTSKVLDVLMTMGRTLSQQKSSGSTEMSFAQRKELIIDMVQWARTIGQFVVGAASKSGSSIDMSSLGIDGVDANADAAAGGSPSSGVSPSTGGAGGSTDTSSGIGATGSTSTSSGTGATGSASAGGNYTAGGSASSNTQSPSGASAPTGSSTQGGSSFGRSFSGKTGSASYEGSVNYQSGQKASQQSTSGR

>Iin01354

MEVVSAHLKLKPSLPQIEAPKTVGEIEPYTVKVVMVFVSDLEKECPKTSKFKAFFEKLRAYAKYVCPIGRKDEKDYDRDMKAKAGSLLQAISSFAVGKKIREEIREEKEEAIQTFKFLKSVAARILGGRKKEESEESMKLTAEQQKEIQEGILKWETVIAKITNTMVETTTTTTTTTTTSGEESTSGKESQTGGNSKSSGSGSSSSGSGVTFKDTTGSGSNSGSPSGSPTNSPSGSTYAAAGGSGSSANSPSGSTYAAASGSGSPANSPSASTYAAAGGSGMAEVEAETSEEVMTFIMNLEKKCPPKEEYKSFFEKLKGTMIASVKVTAEKKKGFFAGVKSAAGKVSDAMSFMGSRIGSKSSEVKKSMENYHAEVTKALQELDAIHSKIISQNQGKKDGSVTVTVEQRTEIKQTITKWETVTTQFVETCMNSEASSNSTVGIDKVKLT

>Iin01394

MLVVASSVIYEAQGTFLLKKYLRSRFPSKCNEFTPYANQGMIMFVTNLEGSCPATPEFKSFFTQFKSYMTFIDTASASKNMDVEMTTKCDGLFKAMSALNGGAVGKSADAGSFKATMLSMGKILVEQKKNTMMMTIAQKKTLVMAMVKWTKMIATLVKTASEKKGKTIDIAAYGLDVDVNDKSIISVTGSTESSSTKTETKTRTEGSTKKEASSTKTGEASATKAGGSGSTATKSGSGTTFKDTTGGNAGSPSGSPTSSPSATGKTSEKAKGNAAASGSTTSTTQKQSSSGSTSVKQVETQTSKEVMSFIMELEKKYSAKAELKPFFEKLKASMTASATLSSKSQQDYVARTKAATGGLSEAMRFVGATKSAKMKSNMETCQEQLMRTLKELQDINSKIVVAKTVTSTQQTEIKQKITKWEQLTTQFVQTAASSASSTSQQSQQSQQQSSTSSHTQSNVGMVKNN

>Iin01395

MYLKRKFFPRRCRDFTPFACKGMLKLMGALQGGCPATREFKQFFGLFKSYIQFILSITSSSKSIDTEMSTKCDGLAKAMALLSGVKGGVDASSDFKETMFSMGKVLVEQKTSGSIFMRRAVRRELIVSMVKWTRTIVTLVSAAAEKRGKTIDVSSLGLDVDVNSSVGSDDSDESDDESPSSGSPSPKTESQNSGSPSDENESSPSPKTESQNSGSPSNEDESSSSGSATSSRSGSNSRAASGSSNKDTSDGGSGSPSGTPSDSSSPSVPSPSVPSPSVPSDSGETAVKGTASGSGEATTKTKKSATGSTKTKNVNEYSDESGATSGSSTDSPSPSVPSPSVPSPSVPSPSVPSDSGETSVQGTASGSGEASTKTKSEGTSSNSATTSVKQVEKETSKEVMSFISSLEQKYAGKAELKVFFDKLKSSMSASSKISTTDSKDFISGMKSAAVKLSEAMVFVRSKFSKSEEMKTNMATYQAQLMKTLKELQEINSEIVTQSKGKTVTSSQQTELKQTITKWEQVTTQFVETAASSSSQSSSSQSSYQSQQGSSMMNSQAS

>Iin01396

MLSFVDNLESLCPLKGEYKEFFTKLKAFMAFINSASGSSSEFQSQLKAQSEELFKAISALGGKAGSSADTSKLIESLMSMGKTLAEFKRSGSQTMTSEQKRELITSMAKWAQVIGQFVKTVGEKSGDGNIDLASLGFGGGGNNANAGSAGGGSTGMGDGSSGGMGDGSIGGSGSPSDSGSPSSRMGSPSGETDSPATGAGSGDNPNDSGSPNLDSGSPSSETGSPTDGGSGSPSGSPSDSSDDVEGTAGAGSGLTNGGTPGAGGPSGTPTDSSSSGSGAGGGVGGGAGSGDGAYGAGGGPSSGGPSGTPIVSSSSGSGAGGGVGGGAGSGDGADGGAGAGAGGGPSSGGPSGTPTDSTSSGAGGSAGGGAGGGAGAGAGPSSGGPSGAPTDSSDSSSGAAGGAAGGAIGGASGGAAGGASGGAVGGASGGAGSGTDGGGGGPSGNTDDMSTGANSMGGKMSYEDSKMAVSGNANEESTTGGAGGGSTYNSAVGGGSMNGGGVYSDSTGGSTMGSPVGSPSSSISGNGESSYSASGSGSSSYQAGGGIAGGPSGSTSDNSVKGESMSGGYSAQGSTTTQTNTKGSSDGSSFAAGGGSGSYEGGSGGASEGKTFKGKIGSVSYEGSVNYKKTHSQSSGQSSVNHSSEEKNSR

>Iin01397

MKTVSLLFLCCISILATSLSLDVVSGHEMDVIPPNTIKDIEPYISNRAIGFVSNLENNCPIREKLRSFFDKLKDLLKLESSVTPLIDNNEPETFKLHLKSKADNLLQTVMMIGRGLLSSSVRKEMFEVMKSLTQLHAAIGKVIMEEHIKGDGSMSLSLGQKNAVENAVSQWEVTITRIVKIVVQVKSEGSSAASGEESTTTEHNSTVNASVNGTMVETNGGNSEPMQGNLDGVKESNEDSVNHVVMEAKEDSNNEKQLEGQGGSIGDSTIGNNNLENKGNTTDGNFMTEKSEEVQGNEGVSPKDNNLENKGTEATINDGDHNKENGEETQENNGELLKDENLENKGSEATTNDGDHKKENEEEAQENNEELVKDENLENNKDNKELKDEGLVETKTNHEISMEEKREHTQGGTEVSINGETMHTKDGNADSNKEKEVELQGESTGDSTKDINLENKEDVKPEEEVNKTDESSTMKKQEDAQGNSGVSTKDQNLENMGAHGETVDHKPVEAMENNGDQVKEKREETQSNNGETVKDEKQEDGQGNDGVSTKDQNLENKGAHEEHIDHKPAEAMENNGDHVEEKTEETQGNNGETVKDENLEKKEDKNKLKDNGSVEAKKSNESSMEEKWEEAQGSNEVSMEGQTEENIGGTADSNKEKGVEVKEGHVEDSTKENDGSSTKEKGEEAQGNNEVLQEDKNLENIGGEHEMKDNMSVESKTKTNNETSSEDNESLEAKENNETSMKEKQQDAQGKGGEYTKVENKEGKDDKSAEAKESSTKKKEEKAQGSGRDTTKVEKNESKDAKSMETTEKERGSAENMGTKVQKGSGDSTQYNKDESRRKNKDTVDEKV

>Iin10907

MARVHLLLCFTLLFASVTILDVVSAHLSYKPALPQIEDPQTVNDVEGYTVKVVMVFVSDLEKECPKTSKFKFFFEKLRAYAKYVCPIKRNDQKDYDLDMKAKAGGLLKAISSFAIGKIKEEIQEEKMEAIQTFKWMKSVALRIMGGRKKDEGEANMKLTAEQQKEIKEGILKWETVITRITNTMVQSTTTSSSSSESSSSEKESSGSKGSFKDTTGSSSKSGSPSGESPSSSPTTKSSSSESSTSEKESSGSKGSFKDTTGSYSKSGSPSGESPSSSPSGSNSPVESGTKTSAKDTSGGKSTVSSGSPTNSPSEDSKKSTTSESVVSASESKAMTVTEVETQTSEQVMAFIMNLEKKCPQKEEYKLFFENLKGTMTTGSSKASSPKRKGLFSRIKSAAGKISEGMALIRSRIGTKSAEVKKSMENYQGEVMKTIQELDAIHSQIVSQQQSKKGGALTCTAAQQAEIKQTIVRWEQVTTQFVETAIQSESSSSSSSSSSSSEKVQAN

>Iin20226

MSRFQTLLCVTILLATVTFFNGASAHIKIKPALPQIGDPTTVNDVESYTVKVVSTFVAELEKECPKTEKFKHFFEKVNAYSKYVCAVSNVKGSESEMKAKAGSLFQAVSDLGSCKNASKGGKMNKSLKKEKSEAMDTVKMLQSIGEKIGGGKNNKTEINGTKKLTVEQQKEIKDGMLKWLQVITRIAKTTEETTLQYSSKTQTSRESKEEKSSTRTETKTSSRAAKAGNNDDVSKSDKKSKKKSDNDKKKKGDSKKDSKSTKEDGKGSGSKEGKSSKSKGESSEKADKNHKVSYGKIEPKKAGVEPARNKTEERIRAEMADELD

>LA_C42497_1

MARISLVISLMFLIASSVIYEAEGFFLKRFLRKAFPKKTDECTAFANKGMILFVTDLERSCPPTTEFKTFFTQYKSYMSFIESASASSKDVDAEMTSKCDELYKSMSKLSASKGAKSADAGSMKVTILSMGKTLLAQKKKAVVMTLEQKKEFALAMVKWTRTLATFLKSASEKKGKTINISTFGLDVDVNDKSIIGEGTESSSESTTKTGANSESTTKSGAKSEITQSKSTSTSTSLTVDEMEAETSKNVMAFIMKLEKKCPQKEEYKGFFEKLKATMTFSGKASPKKRKGLFSRIKSAAGKVSDAMAFIRARIGGKTAAAKKSLENYQGEVVKTMEELDAIYTKIVSQNKGKQEGSVALTAEQQKEIK

>LA_scaffold1003_15

MARISLSICLILVVASSVIYESIGHPQDQAQAQSQSQGQAQSQAQGQAQSQAQRQSQGQRQAQGQFEGSQSHSHSQTQTSSSKSSYKMSYSASVQQVETQTSNQAMSFISQLEKKYATKSEIKTFIDKLKSSVQTCSNIGNKNVKDYVSASKAATVKLDDAMSLLSKGYVKSAKMKSNLEQCQGEVMKNFKQLSDINSQIASTKKATSAQQEELKNTVKNMEKATTHFCEMVATPESSTGKGGVGIKSKMTN

>LA_scaffold1003_16

MARISLSICLMLIVTSNVIYESKSHGQVQNQMQSQSTRNMAYSASVQQVETQTSNQAMSFISQLEKKYEANSELKAFFQKLKSSTQTCSSIGTKTAKDYVSASKAATDKLSAAMSTVGKGCDSSPKLKTNLETCKGEMINTFKQLQTINSQIAISKKVTTTQQTELKQTIVKMEKVTIQFVQIVATSSSSSSMSSSQQNQQFLGSKARMVQKN

>LA_scaffold2543_3

MARINLLLCFILVFSSVTLLDIVSAHLKTNPNDMPQMEAPKTVKDIEPYTVKLVTVFVSDLEKECPKTNKFKAYFEKLRAYAKFVCPVKGKEEADYDKDMKAKAGGVFQTISSFADGKSKDKIQEEKKEAVETFKYLRFLGTKILGEDKKEENKEAKQLTPEQQKEIKEGILRWKIIITKITNTMVVSTTNSSSSGEGSKGQEKSPDDKNKSSGGNSTNKGAPSGSPSSSPSGSKGQEKSPDDKNKSSGGNSTNKGAPSGSPSGSKGQEKSPDDKNKNSGGNSTNKGAPSGSPSSSPSGSKGQEKSPDDKNKSSGGNSTNKGAPSGSPSSSPSGSKKDNGVMISPTGSMTIPETKKNSTKEGSQNGTMTVKEVEAQTSKEVLGFIMNLEKKCPQKEEYKSFFEKLKATMIAPPKIAANGIKGFLEGFKSAGGKVSEAMTFMRSRLGSKSAEVKQSLEGYQGEVMKTLQELEKIHIQIEKDNKGKKPEELTVTTQQRTEIKQTITKWEQVTTQFVETALKSVNDTSSDPDTNLSKAIGKINLPHAS

>LA_scaffold2994_1

MAFIINLEKKCPQKEEYKGFFEKLKATMTFSGKASPKKRKGLFSRIKSAAGKVSDAMAFIRARIGGKTAAAKKSMENYQGEVVKTMEELDAIYTKIVSQNKGKQEGAVTLTAEQQKEIKVTITKWEKVTTQFVETAVQNEEQSSSSSSSSSSSSSSQGKLEKN

>LA_scaffold330_3

MTKFSLVLCLMLLVALSTVYETQGTFSLPHYLENFPKQSKDFVAFADKGMSDFMGELEGKCPNSAEFKDFFVVLKDYMTCFDSTSKKDIQVEMALKSEKLFKAMSLLDGKNGIAEDSWRLVNGMLSLGKHLVEMKKSSSMEITFDQRKELIGSMVKWTRAVGLFVKTASENRGHPIDLSTFGIDYDYNASSSSSKRTLYETQGTFSLPIYMKNFPKISKDFESFAYK

>evm.model.scaffold165.149

MARISLAVCVMLLVALSGVYETQGTFSLPHYLENFPKVCKDFDAFASKGMSDFMSDLESKCPKTAEFTDFFANLKDYMAYFSSTSPESKDNMFEMSKSSDKLFRTMSKMEGSGITADSWRLVDSLLSLGKNLVEMKKCGSKELTFEDRKELIGNMVKWTRAIGLFVKTASEKKGQSIDLSVYGVDYDFSSSSSSKRTLYETQETFSLPLYLKNFPKMGKDFEPFAYKGMSDFLGSLESKCPATTEFKDFFAKLEDFMTCFKSASPGSKDMKFEMAWKSDMLLRAVSPLDGTKGGTSVDFWRMLDGLLSMGKVLVDMKKHSSEEITFEPRKECFGAMIKWARSIGLFVKAASERNGKSIDLSSFGIDYVSGNDQGNPKEKDSPASKPKNEL

>evm.model.scaffold19.222

MAKILLGICLMLVVASSVIYEAQGHFMLKDYLEKKFPSNCNEFAPFANKGMLELATDLQGSCPPTAEFKTFFTQFKLYVTFLESASATSQNIDAEMTTKCDELFKSMAPLCSGKGVKSTDAGAVKVTMLAMGKTVIAEKKNRKPVTSEHKKQLATDMSKWTNTFGSFVKTASERKGKPLDIATYGLNGNANAGGAASGGKTESGSSASTKTESGSSTSTKTESKSASAKSGSGFKDTTGPTLGSPSGSPKASPSGSSPKASPSGSSSAGGSETSTKGNASAGGKASGKTMESKESKKSQSKTSSESSSKNSSSSSTYASPKQVQEETSKEVMTFIMELEKKNSAKSELKIFFEKLKATMKTACATTAAQNPKEYAFATRSASDKLSEAMTLVGSRNQESKVRRFRSLISQFTD

>evm.model.scaffold19.225

MARVQLLLCFTLIFASVTLLDVVSAHIKLKPGLPNIEPPLTVDDVEPYSVKVVTVYVSDLEKECPKTSKFKSFFERLRGFAKYIVPIRRKGQEPDYDSDMKAKAGSLFQAISSFAIGKIKEEIIEEKNDAIKTFKFMKSIAGKIVGGGQKSEASGKLTTEQQTEIKEGILKWETVITRITNTMVMSEEKSSSSSEASATTKEESSSSGGKASEESSSTKEGSSSKSEESKSEGSKSKESKSEGSKKESSSSKSEGSKGTAAFDDTTGSASNSGSPSESPSSSPSGSSSKTESKKESASGKKTEASGSKKTEANGSESTEASKESSGSESTEDSSGSTSSKNVETSSKKSVTVQQLEAESSKEIMVFIMNLEKKCPQKEEYKSFFEKLKGTMTGSAAVSKKKGIFSGIKSAAGKLSDAMVFIRSRIGSKSAEVKKNMETYQAQIIKTMQELDTIHSQILSENKGKSEATLTCTPEQQTKIKQTITRWEQVTTQFVEVAVKSESSSSSSSSSSSSSSSGSASGSLRGQAMAN

>evm.model.scaffold19.227

MAKNFLAICLMFMVVSSVVYEVQGHFLLKLYLRRRFPRRCMDFAPFAFKGMMMLLSNLQGACPGTRDFGEFFSRCKSFISFVSSASATSINIIGEMSGRCELLAQSMSKMSGGKSLDVENVKDTMMSMGKTLIEQKKQGSKIMTYQQKKELVVAMVRWTRILVIFVKNAAEQSGKLIDIGSFXXXXXXXXXXXXXXXXXXXXXSQSQTGSNSGSGSQTSQSSGQGQDTFTGSGSQTSQSNGQGSQVSQFNGQGSQSSQSNGQGSQSSQSNGQGSQTSQSSNQGSHGSQSSGQGSQTSQSRGQGSQGSQSSGQGSQTSQSRSQGAVSGSGSKDTQSSSQDAVSGSGSEDTQSSSQGAVSGSGSEDTQSSSQGAFSGSGSEDTQSSNQGAVSGSSAKDSQSNSQGAVSRSGSKDTQSSSQGAVSGSSAKDSQSNSQGAVNGSDSKDSQYMSQKAVSESGSKDSQTSTNKGSKATGNQSETQGSSATGSQSETQGSKATESQSETQGSKATGSQSETKGSSDTGSQSETQGSSAIGSQSESGSASAQKETKSESSSNDESLKAVEKETSSEMMSFISNLEKKYPGNTELKGFFDKLKGTVSTSSKISSSSTAKDFFSGMKSAAGKIAEAMMSVKAKFTKSEESKSTMENCQQQMMSTIQQLEALKSQMASDNTVTTTQKTVLKQTVTKWEQVTTQFVETAASSSSSSSSSSSSSSGQQHQSRSVAMLSSN

>evm.model.scaffold19.228

MTKTSLLFLCCITIVATSLNVVLAHENPPNTVKDVEPYIANRAIGFVSQLENNCPVKDKLSSFFNKLKDLLKLESSVTPLLEDNEPKTFNFDLKSKSDGLLETMFAIGKGLLSSSVRNEIYEGIKSITELHAAIARVIEEEHIQGSETTSLSVNQKNAVENAVSKWEVSITRIVKVVVQVTSSSSSESATTEETNTTGQNNESMDGNMKETNAENSESTQENVNSNNENEVGAQGGSVGDLTNKNDGSSMNEISEEAQGNDGDSTVDNNLKNNGNEDKVLENTTQNSQNNTRENKEETNKNNETSMEEKQEETQGGNVDSSKEKEVDVQGESTGNSTDVTNLENKEDANLGVEANVNDESSTKKTQEEANANKEETQGTNGEAQANNGESEKKTEAEALTNNAESEKKTEAEAQTNNEESQKKTEEEAQANSGESEKKKEAEAHANSGGSEKKTQRESQENSRESEKKTEAEAHENSEESKKKMEAQTHASNEELEKKTKEEAQANNEESEKKIEAEAQKNSGELEKKIQGETQVNNGESEKKIQGEAQVNNGETEKKTQGEAQANNGETEKKPQGEAQANKGESEAQVNNGESEKKTQGEAQVNNGESEKKTKVEAQANNGESEKKTEAEAKENNKELKKKTEEDAQKNNGESEKKIEAEAQVKIGESEKKIEAEVQANSGESKKKKEGKAQKNSGQSEKKTEAEIQESNVESGKKTEAETQANNGELEKKTEKEAQAHNGQSEKKIEVGTQESNAESGKKTKSKAQGNSGESEKKTQAEDQVNSGESEKKTQGEAQANIGESKKKIEAEAQANNGESEKKTEGEAEANNGDKSITTKTNKESSKEEEQNDNQGKSGESKEVDTKEKQQENGGVSTKIENKEGMKDSKDDKSLQKSKEKSKGSDKNKSEDAMSKKITKKDKSSAENMNIDVQKRSGESVQHNKEEKIEKGDGKSKKGEEKKKSDDSNMKKKKEGENEKNDSDNSNLKKQEDKKEKSKSQNRKKEDKNVKKHLGEHESKKNEEEKERKEESKDNEMKEKEDSKKEKKKCNGKEDEKKKSEDGKSMEKEEEKKKKEEEKKKDEEKKKKEDEKKQSKERKLLKKQEEKKKKEEEKKKEEEEKKKKEEEKKKEEEKKKCEDGKSGKKEKEDKYEDGQSMKKEEKKKESESNISKKNEEDKNQNNKSKDVELKNSEEQHKGQEKEVGVKQDSKKRKDKSKEEIARKGQTYGGEFRKKVHGQGQANGGDSDKKVQGEAQGNGGESEKKVQGETQANGGESEKKVQGEAQANGGESEKKVEGEGQENGGESKRKVHGETQANGGESEKKVQGETQTNGGESEKKLQGEAHSNGGDSEKKVQGEAQANGGETEKKVQGGGQSKGGESDKKVQGEAQANGGESEKKIKGEAHANNGEYEKKKEAEAHANSGESEKKTEVDAQANIGESEKKIKAGVQANNGESKKKTKGEAQVKNGESEKKTQGESEGNKGKSEKKKQGDTQANSSETEKKTEAEAQINSGESEKKMEAEAHANSEESEKKREANAHGNSEESEKKTEAKVQAESGESDKKTQGEAQVNSGESEKKTQGEAQENNGESEKKTQGEAQANNGVSEKKIEEEAHVNSGESEKKIKAEAQSNSGESEKKTEYEAQPNTGESEKKMEAEVQANNGESEKKMEAEAQSNNGGSETKIEAEAPTNKGESEKKTEAEAPVNNGKTEKKTEVEAPANNGESAKKTEAEAPANNGEAEKKVEAEVPVNNGESEKKMEAEAPANNGEIKKKVESEAHVNNGETEKKTKAEAPAKNGESEKKTEAEAPVNNGETEKKTEAEAPANNGESANKTEAEVPVNNGESEKKMEAEAPANNGEIKKKVESEAPVNNGETEKKTKAEALAKNGESEKKTEAEAPVNNGETEKKTEAEAPANIGESEKKTEVDARVNNGEAEKKVEGEVPVSNGESEKEIEAEAPANNGEIKKKMEAEAPVNNGESEKKTEAEASVNNGESEKKTEAEAPTNNGELEKKIKEATKESNNSMENQNMQGGGSNTNLTNNEKTKETMNSTTKRNVTTDIESNISKEVTSFISNLEKKSPGTPEFQSFFKKLEDCMTYLLPTTSTFESKDSNSYMSEMINVASQLSDSMAVLQAKKSGSQQVKTTLQGYQQEVMKTVTTLQSVMKIAHQSKNGGSLTLTLSQQETIKQIVSKWEQVMSQFVNVACEGEKQFSHSSSSNSSSSTSSSSGSNSSLESNSGGSSNLGSNLNGETPSLDSNLGGKIPSLDSNLGGQSSSLNSNLGGQSSSLESNLDSGSSSLESDLDGEI

>evm.model.scaffold309.328

MVKVPRHHNLAVKVPKHHNLVVKVPRHHNLAVMVPRAHNLAVKIPKHHNPVVKVLLVEVVPRHHNLAVKVLLAEALLRTHNPSTKIMLAVLRTHNPAATALKLQEVKVKHKALKLPEIKVKQKALKLPEVEVIHRALKLPEVKVKHKALKLPEVKVKHKVLKLPEVKVKHKVPEVKVKHKARKVPEVKVKHKALKVPEFKVKHKVLVLPEVKVRAEALPHRKIQRVKVHPKYPGNTELKGFFDKLKGSVSTSSKLSSSSTTKDFFSCMKSAAGKIAEAMTSVKAKLTKSEDKKSTMENCQQQMMGTIQQLQALKSQMASSNTVTSTQKTVLKQTVTKWEQVTTQFVETAASSSSSSSSSFSSGQQHQSRSSAMLLSN

>evm.model.scaffold309.330

MTNPPLLFLCCITIVATSLNVVLAHENPPNTIKDVEPYIANGAIGFVSQLENNFPVKDKLNSFFDKLKDLLKLESSVTPLIENNEPKSFNFDLKSKSDGLLETMFAIGKGLLSSSVRNEIYEGIKSITELHAAIARVIKEEHIQGSETTSLSVNQKNAVENAVSKWEVTITRIVKVVVQVTTSSSSESSTTGETNTTGQNNESMDGAQGGSVGDLTNKNDGSSMNENLEEAQGNSGVTTMDNNMENNGNEDKVLENTTQNSQNNAMENKEETKKRNETSMEEKQEETQGSNVDSNDKEVEVQGGNNGNSTDVTDLENKEEANSRVEANVNDQSSTKKTQGEANEKKEETQGTNEEVANGEAQAPNGESEKKTQGKTEAEAPTNGGELEKKTEVEVQAKIGESEKKIQGEAQLNTEESEKKTQGEAQANSGESEKKTEAEAHANRGESEKKTQAGAHENRIEFEKKIEAEAQENKKKTQEETQAQSGESEKKTEEETQANGGESEKKTEAEAPTNSGESEKKTEAEVQAKTGESEKRTQGEAQLNTGESEKKTQGEAQANSGDSEKKIEAEAHANSGESDGESEKKIEAEVQANSGESEKETEASAQGNSEESEKKTQGEAQANNGDKSVTAKTDKESSKEEGQRDNQGKSEEFIDVKTNENQQENGGGSTKIENTEGEQKPKKNEEEKERNEESKGNEMKKKEYGKTEKKDSGEHMSKKKEDDHNMTEKCDGKEDEKKKSEDGKSMEKKEEKKKKKEAKKKKEEEKKISKERKSLKKQEEKKKKEEEKKKKIKRKKRRKSQKMTNQEESARKGKTNGGESEKKVHGEGQANGRESEKKVQGEAQENGGESEKKTQGEAQTNGGESEKKVQGETQANGGKSEKKTEAEAQVNNGESQKKTEAEAQGNSGESKKKTQGEAQPNSGESENKTQGKVHANKGESEKKTEAEAEDNSGVSENKTQGEAQGNSGESEKKTQGEAQGNSGESEQKTQGEAQGNSGESEQKTQGEAQGNSGESEKKTQGEAQDNSGESENKTQGEAQRNNEESEKKTQGEAQANNGESEKKTEVEAHVNSGASEKKTEAKAQANNGVSEKKTEADVQANSGESEKKRKGEVQENNGDSEKKRQGKAQAGSGESEKKTQGEAQANNGESEKKTEAQAQTNKAESDKKTEAELKQIMEAEVQVNNGESEKKTKAEAQTNSGESKKKTAAEVQANNGEFEKKTKAEAQAKSGESEKKTDAEALTNSGESENKTDEEAKANSGEYEKKTEAEAQANNEESEKKTEAATQVNSGESENKTEEEAQANNEESEKKMEKKEAEAQANNGESEKKTEAEAQANNGESEKKTEAEAPANNRETEMKTEAEARANNGESEKKTEVEAQVNNGETEKKTEAEAQANNGELEKKIEAEAQAKTEESEKKVQEEAQAQGKNEESTKDKKTEDSTKESSEDVKTAEVNRGKNSTEESFIDGNKTEETMKESNNYMENQNMQGGGSNTNSINNATTEETMNSTTKQNVTTDIESNTSKEVTSFISNLEKKSPGTSEFQSFFQKLKTCMNYLLPTTSTFESKVSTSYMSEMINVASQLSDSMAVLQAKKSGPQQVKTTLQGYQQEVMKTLTTLQSVMKVAHQSKNGGSLTLTLSQQEAIKQIVSKWEQVMSQFVNVACEGEKQFSHSSSSNSSSSTSSNSCSGSNSNLESNSGGSSNLGSNLNGETQSLDSNFGGQLPSLDSNLGRQSSSLDSNFGGQSSSMDSNLGGQSSSLESNLNGGSSSLESDFDGEI

>evm.model.scaffold448.20

MAKYLSGICLMLVVASSVIYEAQGHFMLKNYLENKFPSKCNEFTSFAKNGMLELATDLQGSCPPTAEFQTFFTQFKSYVTFVASASASSQNIDAQMTTKCDELFKSMAPLCSGKGVKSTDAGAVKATMLLMGKTLVAEKKNPKPVTPQHKKELATDISKWTNTFASFVKTASERKGRPLDIATYGLDGNANAGGAASVGKTQSGSSASTKTESKSSSAKSGSGFTDTTGPTLSSPSGSSKASPSGSSSAGGSETSTKGRASGGGKASGNSMESKESQSKTSSESSSKNLSSSSSTSASPKQVQEETSKEVMAFIMELEKKHSSKSELKIFFEKLKATMKTACATTAAKKPKEYVSATKSASDKLSEAMTLVGSRNQESKMKSNMDTSKNELLNSVKQIQDMTSQMSSEKTVTSTQKTEMKQTVTKMEQATTKFVEAASSSSSSSSSSSSSSSSSSSSQQQQGSAGMVKKE

>evm.model.scaffold448.21

MPRVQLLLCFTLIFASVTLLDVVSAHLKIKPSLPNIEPPQTIDDVEPYCVKVVTVYVSDLEKECPKTSKFKSFFEKLRGFAKYVVPIKRKGQEPNYDSDMKAKAGSLFQAISSFAIGKIKEEIIEEKNDAINTFKFMKSIAGRIMGRGQNSEASGKLTTEQQTEIKEGILKWETVITRITNTMVISEEKSSSSSEASTSTKEESSSSGGKASEESSSKKEGSSSKSEESKSEGSKKEGSKSEGSKSSPSESPSSSPSGSSSKTESKKESASGKKIEANGSKKTKASGSESAEASNESSGSESTKASSESTSSKSVETSSKKSVTVQQVEAESSKEIMVFIMNLEKKCPQKEEYKSFFEKLKGTMTGSATVTKKRGGIFSGIKSAAGKLSDAMAFIRSRIGSKSAEVKKSMETYQAQIIKTMEELDTIHSQILSENKGKSKATLTCTPEQQTKIKQTITRWEQVTTQFVEVAVQSESSSSSSSSSSSSSSSSGSASGSLRGQAMAN

>evm.model.scaffold891.60

MLVVASSVIYEAQGHFVLQSYLQNNFPSKCNEFAPFANKGMLELATDLQGSCPPTAEFQTFFTQFKSYVTFLESASAASQNIDAQMSTKCDELFKSMVPLCSGKGVKSTEAGAVKATMLAMGKIVIAEKKNPKPVTPQHKNELAKDMSKWTNTFGSFVKTASERKGKPLDIASYGLKANSNDKSAAAASTKTESKSASTKASPSGSSPKASPSGSSSAKETSTKGNVSAGGKASGKATESKESKESQSKTSSKSSSKQSSSSSTSASPKQVQEETSKEVMAFIMELEKKHSAKSELKIFFENLKATMKTACATTAAKKPKEYASATRSASDKLSEAMTLVGSRSQESKMKSNMDTSKNELLNSVKQIQDITSKMSSEETVTSTQKTEMKQTVTKMEQATTKFVETASSSSSSSSSSSSSSSSSSSSQQQQGSAGMVKKQ

>evm.model.scaffold891.62

MERVQLLLCCTLLFASVTLLDVVSAHLNIKPSLPNIEPPQTVDDVEPYCVKVVTVYVSDLEKECPKTSKFKSFFEKLREFAKYVVPIRKKGQEPDYDSQMKAKAGSLFQEISSFAIGRIKEEIIDEKNDAIKTFKFMKSVAGRIMGRGQKSEASGKLTTEQQTEIKEGILKWETVITRITNTMVISEEKSSSSSEASATTKEESSSSGGKASEESSSKKEGSSSKSEESKSEGSKKEGSKSEESKSEGSKKEGSKSEESKSEGSKKEGSKSEESKSEGSKKEGSKSEESKSEGSKKEGSKSEESKSEGSKKEGSKSEESKSEGSKKEGSKSEESKSEGYKKEGSKSEESKSEGSKKEGSKSEGSKKDSSSSKSEGSKGTAAFDDTTGSASNSGSPSESPSSSPSGSSSKTESKKESASGKKTEASGSKKTEASGSDSAEASKESSGSESTEASSGSTSSKSVETSSKKSLTVQQVEAESSKEIMVFIMNLEKKCPQKEEYKSFFEKLKGTMTGSSTVTKKRGGIFSGVKAAAGKLSDAMAFIRSRIGSKSAEVKNNMETYQAQIIKTMEELDTIHSQILSENKGKSEAMTCTPEQQTKIKQTITRWEQVTTQFVEVAVKSESSSSSSSSSSSSSGSASGSLRGQAMEN

>evm.model.scaffold891.63

MAKNFLAICLMFMVVSSVVYEVKGHFLLKLYLRRRFPRRCMDFAPFAFKGMMLLLSNLQDACPGTRDFGDFFSRCKSFISFVSSASATSANIIGEMSGRCQLLAQSMSKMSGGKSLDVGNVKETMMSMGQTLIEQKRQGSKIMTYQQKKELVVAMVRWTRILVIFVKNAAEQSGKLIDVGSFGXXXXXXXXXXXXXXXXXXXXXXXXXQSQTGSNSGSGSQTSQSSGQGQGTFSGSGSQTSQSNGQGSQTSQSSGQGSHASQSMGQGSQTSQFSGQGSQGSQSTGQGSQTSQSSGQGVASGSGSQITQSSSQGADSGSGSKDTQSSSQGAVSGSGSQDTQSSSQGVVSGSGSQNTQSNSQDVVTGSGSKDTQSSSQGSVSGSGSKDTQSSSQGAVSGSGSKDSQSMSQKAVSESGSKDSQSSTDQGSKEIGSQSETQGSSATGSQSETQSSSATGSQTESGSASAQKETKGESSSSESLKAVEKETSSEMMSFISNLEKKYPGNTELKGFFDKLKGTVSTSSKVSSSSTAKDFFSGMKSAAGKIAEAMVSVNAKLTKSEESKSTMENCQQQMRSTIQQLQALKSQMASSNTVTSTQKTVLKQTVTKWEQVTTQFVETAASPSSSSSSSSSSSSGQQHQSRSAAILSSN

>evm.model.scaffold891.64

MSQDFEPYAFKGMLSFVDNLESSCPAKTEYKEFFLKLKAFMGFINSASGSSSSLLQVQSQMKSHSEELHKAISALGVKGVSPAETSKLIESLMSMGKTFVEFKRSGSETMSPEQRREMIIAMIRWAQVIAQFVKNVGEKNGDNIKFDLSSLVNDADGGSIGGGSPTSGGPSSESGSPSTNIGSPNDSGSPSGSLAGENGSGGDMTGTKSTSGGGAASGGNASDGGATSGESASSTGATGGGDSSSGDATNGGSATDGESANGGGATGGESASSGGTAGDGDASNEGAANGGSTTGGESASGGGASDGENSSGGGAXXXXXXXXXXXXXXXXXXXXXXXXXXXXXXXXXXXXXXXXXXXXXXXXXXXXXXXXXXXXXXSRTDIGGSDASGGSAGGGKSSSGDAAGGSSTSGVSDSSSGNANSGEAASGANNEASSMAGEGSYSDTTGMGANGSPAGSPSEEPSGSTPENSSGSRSFKGRIGNINYQGTTLHILLSDKPLLGVMVTQAFLAASRRDPLPATLPLGFSGIHIFRWGFPVETFISIFHLLPRESLCLEPTSPPSCMRLQDFHGVTSL

>evm.model.scaffold891.65

MSEMINVATKLSDSMAVLQATKVKTTLQGYQQEVMKTLTTLQSVMKIAHQSKTGGSLTLTLSQQETIRQIVAKWEQVMSQFVNVACEGEKKFHTLLAPTQALARVQIRIWDQILVEVRI

>rna-MERR_LOCUS39158

MAKNTLAICLMFLVASSVVYEAQGTFLLKMYLKRKLFPRRCVDFTPFACKGMLMLASNLEGGCPATREFKNFFVQFKSYMSFISSVQVSSSKSIDVDLTSRCELLSKAMTLLNGAQSSSDDFKMTMLSMGKTMVEQKKRGSMIMTISQRKELVISMVKWTRLVATLMKTAAEKKGKSIDISSYGLDVDVNASVGSSSEASSSSQTESQNTGSQNTGSQNTGSQNTGSSSTSQGSSGSATSSRQGSQSSTNGGAASYSGGGSGSTYQDTTGGSGSGSPSGSSPSGSPTPSGSSPSGSPTGSPSGSASDSGSASASGSASASGETSVKGSASKTKESKSESKGAASSESKQSSSGSSSQSGGGTTYSDTTGATSGSPSGSPTESPSGSPSGSPSGSASLSGETSVKGSAESKSAASSESKGAASSESKGAAETESKGAASTESKGAAETESKGAAVTESQGAASSTTSSSSSSTSSVKQVETETSKEVMSFISGLEQKYASKAELKVFFEKLKTSMSASSKISTTDTNSFLSGMRSAVGTLSQAMGSVRSKATKEESKSSMQSSEQQVIKTLKELEDVNSKIVSQSQGKTVSSTQQTELKQTLTKFEQVTTQYVETAASSSSSSSSSQQGMAMKSQSQAS

>rna-MERR_LOCUS39156

MSRTTSAVCLLLLVALSAVYEVQGTFLLRHYLRKFPRRSRDFSPFACKGMLTFVKLLELKCPLKPEYRSFFGRLRSYMNFISSSSGSQNFDVELKSQAEGLNSAMSALGGGSSADNSNVLDTLTSMGKTLSAQTRSDSTTEMSLSQRKELIMSMAKWAGVIGKFVSTAASKSGTSIDISSLGIDGIDANMGSPSADAGGSPTSPTTGTGASPTSPTTGTGGYPTSPTTGAGGSTYTGSGSGTGGSNTQVGATASGGSPYNGGYNTQVEGTARGETTSSGGTGMGGGAQTAINGGGGVYDKLIRTIDDCVWHVDSSFHGRFTIDFEFLELKISPLNGGEASPVWPNDTTMQSISTQTTLKCLSRMLEESILSDVTIPNPHSRRYALRSQRHPLG

>rna-MERR_LOCUS49592

MARAHLLLCFTILFASVTLLDVVSSTLNFKPALPQIADPQTVAEVEPYTIKVVMVFVGDLEKQCPKTSKFKMFFEKLRGFAKYVCPIRRLREKNYETDMKAKAGGLFQAISSFAIGKIRAEIQEEKNEAIANYRFMKTLAGKILGGRKKDETEDTMKLTAEQQKEIKEGILKWETVIAKVTTTMVQSTTTSSSASEGSASAKEVSAGGKQDSASGSSYKDTTGSSSSSGSPSESPSGSKSSGSSSDESISKETGSKGSSSKGSSSEESSSKETGSKGSSSKGSSSEESSSKETGSKGSSSKGSSSKVEAKAGGGASFEDSTGGSPSGSPSNSPSETSTSTKSSGKVEASESASGGASASSGGSASVSSQKGEISITEVETQTSEQVMTFLMGLEKKCPQKEEYKSFFEKLKGTMTTTVRVKRGFFSGLRAAVGKVGDAMAFMRSRIGTRSAEMKTKLETCQAEVVKAMEELDAIHAQIVSQNKGKKGGAMTCTAAQQAEIKVKITKWEQVTTQFVEATMESEASSSASASSSSSSGKLTSGGVKPF

>rna-MERR_LOCUS39157

MARISLGICLMFLVASSVMYEAQGHFLLKNYLRSNFPRTCNEFTPYANKGMITFATDLEVGCPQTVEFKEFFTQFKSYMTFIETSSTTSTNINAKMTTKCDGLFKAMSALNGGAAAKSADAGKFKATMLSMGQTLVQQKKSTVVMSLTQKKSLVTAMVQWTKMIATFVKTASETKGKTIDIATYGLDVDVNDQAIMGSTTSTQTRSSSSTKAASSSSTKTQSESSTKIGAVAASGEKASAKTKEASGGAASKKTENASSNKSGSGSSKSKETKTGSGSSKSKETKTGSGATFKDTTGSGSPSGSPTSSPSGETSVKGKSSSKGSASSSASASASSEKKEAKAEKKEAKAEKKEAKAEKKEAKAAKKEAKAEKKEAKAGSASSEKKETKAGSASSTKAGSASSEKKETKAGSASSTKAGSASSEKKEVKAQGASGSASSTKAGSASSEKKEVKAQGASGSASSEKKEVKAQGASGSASSTKTGSASSTMSGSTATASSSTSVKQVETETSKEVMSFVMGLEKKYSSQSELKLFFEKLKASMTASSTISSKTEQDYVSTTRAASTKLTEAMALVSSRMSKSAKMKSNMETSQDQLLQSLKQLQDINSKIVTAKTVTSAQQTEIKQTITKMEQVTSQYVETASSSSSSSSSSSSSSQTQQGSMGMVKNN

>rna-MERR_LOCUS39160

MLSFVDNLESKCPLNGAYKEFFTKLKAFMAFINSASGSSSEFQSQLKAQSEGLFKAISALGLKGVSSADTSKLIDSLMSMGKTFAEYKRSGSQTMTSEQRTELVTSMAKWAQVIGQFVKTVSEKNGGGLNIDLPSLLGGGIGGGSSGSDSSIGAGSTGMGDGSSSGSGSPSFGDSTGDSGIPSGSPSAETGSPADSGSSIPSGSPSDISAMGGGSTDGDASSGMASGEASSAAGPSGSPTDSSDSGAADGGASDAGAADGGASDAGDASGGAYDAGAAGGGASDAGAAGGGASDAGDAGGGASDAGAAGPESSGAGGGAETSGPAGAETSGTTGAGAEASGDAGAESSGAAGGGASDAGAAGGGASDAGAAGGGASDSGAGGGAESSGAGGGAETSGTTGAGAESSGAAGGGASDVGAAGPESSGAAGGEASGAGGGAETSGPAGAETSGSTGAEASGAAGAGGPSGSTTDMSTGGESSTGSESSTSGSQGDTMAAGGGSAYSDSTGGTPMESPAGSPSNEVGASAGGPSESTASGESSMSGVSGGGTQTDSQESSSSEGGSVSAGGKSFKGKIGSVSYEGSVYEKTHSKSSGKSSFSHSSDTNSNTSS

>rna-MERR_LOCUS39161

MKGGSLLSLCCITILATSLSLNVVSGQEEAAPNTIKDIEPYISHRAIGFVLKLENNCPIREQLRSFFDKLKDLLKFESSVTPLIENNEPKTFSFDLKSKAENLFQTVTMLKRGLMSSSVRKEMLEVMKSLTELHAAIKRVIIEKHIKGDGSTSLSSEQKIAVENAVSQWEVTITRIVKIVVEVKTGRSSESSGEESSTTEQNSASVNGTMAESNGENSESTQEKNVGVEGNSLDSRNDVAMEEAKEGDKVVDAKGGAQGNDGVSMKDNNLENKGSEEASKDDKTVEGVTNDGDDTKEKTGENQENNGELVNGENLENKEETKTNHEISMEEKREHRQEGTEVSKNGETMGTNGGNGDAKENEVESQGDSTKDTNLENKGDVKPEVEADKNVESSTKEKQGEAQGNEDIEMDDKPEEATTNNEDETKKKTEETQENNGESVKDENIENKEDQQLKEEGSVEAQANNETSMEGKREENQGGNEVAVNGTETNENKGGNTESSKEKGEEVQGEHVGDLTKDTNLGSKEDANSEVEAKENGSSMKDKEEEAQGNNEVSTNDNNLENKEGEKELKEDESVEGKTNKESSTEEKQEEGQGNNGESQVVETKGGMTESKEDKSVKDEENENVRSSVKEKEDEAQRNGGDKKESKDDKSVESEENRESKDDKSAEANANDSFVEENQEEVQGNNKESKKVEDKGDKKEFKDNKSVEVKDNKESSTKKKEESQGSGRDTTKVEKKRSKDAKSKETTTEKDSGSAENMGIEVQNGSGESEQYKKDEKKDTSDGNSKKENNKKEKKKSEDHNSTKKKKETKEKKESENSKSKKKEEEKKQKKEHGDNKSKIKEEDEKEETDSKDYNSMRKKEDISEKKNSDDKRSKKNEEDKNEKRASEERKSKKREEKRKKKDSKNQKSKKKKEERQEKKQKKEERKEKKESEDNKSGKKEKKEKKEERKVKKESEDNKLGKKESEDSKSMKQEEEKKEKKEERKEKKESEDNKSGKKESEDSKSMKQEEEKKDSKHNKQKKTEKENKEKKKSEDSEKSKKNKEDKKGKKEHEESKLKKNEENKKKEQEENKSKKKKEEQREEKESEKGKSVKNEKDEKEKREAEDNKSNKEEEEKKEKSKSEDHMSKKIRKNKRKEDEKQKKESKDNRSKKKKESKKEKSKYEDHMSKKTGKNEKEEDEKEKRDSEDTKSKEKDEDTKEASKSENHMSKKTEKNKKEKKEEKEKKSEAKKSKRKEKEKEKRESQDKKMKKREEDKKEKVVSQDNKSKKRKDKEGSQSNKSKKNEETRRENKESEDEKSKNTKKQGGDATTELGLQSNTKDAESKEAEKEQENSEQQKQVGDQVNTQDSRKDKEKTKEDGKSKDMQSEAHAKSEESEKELEAKAQATSDNSEKEKQTETEIKSVESVEDNKGESMKDKKAEDTESKTKKEENKDSSTKKQEDIQETNMGEKKDQNGSTAEGSKDSKTTETKEGSNEEVSNDDKTMVNDGGQNTEKDSKDGKEVEVNGGNADSMEEGSKDGKEGSMEEVSDDGKTTEISGGNNSTEETSKDGKTTEINGGKEDSKDGKTVEISGDKEESSKEATENGKIVETGGGKEVSKEEGSMDGKTGENNKGKEESVEEGSKDSNNIEVNKGQEESMEKGDGKTDEINGGKEVTMEEGSKDGKTVEINGGKNSTEEVSKDGKSDETSEGKEAPTEEGSKDDKMVETQGGKDNSIEEGSKDGETNEISGDKNSTEEGSKDGKSDETNEGKEAPTEEGSKDDKIIETQGGKDNSTSTGIKEDKIDINKDSTKNETADAQGGGSGDSMNGKTEETNESSESINNKKIQNGGSTENSTDNQIAGGAVNSTTTTQQNMTTNIESTTSKEVTSFISNLEQKSPGTQEFQSFFQKLKDYMKYACPISSTFETKDSKSYMSTMINMATKLSDAMAVLQAKKIGSGLMKTTLQGYQQEVMKTLTMLQSVMGKSVSEQQSQNDGSLTLTLSQQQAIKEIVMKWEKVMSQFVKIATESEKQFSVETSTGSGFHIKKTSSSSSTSSSNPGSDLKLNDESAKMVGVNG

>Rs024930

MARVHLLLCLALLFAFVTLFDVASAFLKLKPSLPQIEDPKTVGDVEEYTVQVVMVFVGDLEKECPKTSKFKAFFDKLRGFAKYVCPLKIFGKKDDADMKAKEAGILKTIASFAIGKIKREIQEEKQEAIETFKFMKSLAGRILGGRKKEEKATTALTAEQLKEIKDGILKWQTTIVKITNTMVVSTTTSESSETTTGSSESTTGGSSGPNAGSSTGSPSNKPEAGSKPGAGTPSNKPEAGSNPGAGTPSEDTDNESEDTTNTASTGTETGGSKGPNSGSSTGSPSNKP

>Rs024920

MARVHLLLCFALLFASVTLFDVASAFLKLKPSLPQIEDPKTVGDVEEYTVKVVMVFVGDLEKECPKTSKFKAFFDKLRGFARYVCPLKIFGKKDDADMKAKEAGILKTIASFAIGKIKREIQEEKQEAIETFKFMKSLAGRILGGRKKEEKATTALTAEQLKEIKDGILKWQTTIVKITNTMVVSTSTSESSETTTGGSESTTGGSSGPNAGSSTESPSNKPEAGSNPGAGTPSNKPEAGSNPGAGTPSNKPEAGSNPGAGTPSEDTDNESEDTTNTASTGTETGGSTGPNSGSSTGSPSKKPAAGSNPGAGTPSQDTNNESEDTTNAASTSTATQSTSQTKEVTVTEVETQTSQQVMTFIMNLEKKSPQKEEYKQFFEKLKSTMTGKVSSPKKKGGLFAMIKGAVGKIGDAMQFIRSRLANKSAEVKKSMETYQAEVIKNMEELDAIYAKIVAENQNKKGGAMTCTPEQQAQIKTTITKWEQVTTQFVEVAIKSETSTSTSTSSSTGTAQAN

>Rs036520

MSRFQDLLCFTILLATVTFFNVALAHVKIKLALPQTGGPISVGDVEPYTVKVVSTFVADLEKECAKTKKVRHFFEKVNALSECVCSVSKVKDRESHMKAKAGSLFQAISALGSDENGFKGGKVNKLQKEKTEAMETVKMLQSIGEKITGGKSNGTLKLTTKQQKEIKDGILEWLNVINRISKKADEISMKSSSISETKKESKEEKSVGTRILAEKKSSTKSNKNSDKGRDNKKKGSDNKKNKKGESKKKKHGGQEGHHGSAKSKGGSDDDKSSKNKRRSSENSDKNHQTKYGKVEKKKGGVEQSRNKTAERLRAEAADELD

>Rs174090

MARVQLLLCFTFLLASVTFMEHVSSATTASSSSTTTVAQLETETSKEVMEFIMKLEKKCPPKEEYKSFFEKLKSTMVASTKVTTEKKKGFFSAAAGKISDAVSFIGSKFTGKSAEVKKSMETYQQEVAKALQELEAIHKKIIEANQGKVEGSVAVTAEQKTEIKQTITRWETVTTQFVETAIQTEAASNTTVGVDKVKLP

>Rs174190

MARISLGLCLMLVVTSSVIYEAQGHFLLKNYLTTQFPSKSNEFAPYAKKGLTEFLTDLERICPPTPEFKGFFTECRSYMSSIQTSSSSSSSSSSSSQNMDGLSKAVSAISGGAGQQSEEKAEKKEEKAEKKEAKKEEQKEKSGSSAKTKEASSTKAGASSSTKAGASSSTKAGVTFDDTTGASAGSPTGSPTSGEDRTSGSPEASPTSAKGKSSKKESGKAAASSSKQESNAGSAASSMNQQQSKSEKSESSSSSATTSVTEIEKETSQETSSFISGLEKRFAQKQELKPFFEKLKASMTASSRVSSTKSAQEYSSTARSTTGKLSDAMTYVGSRFSKSEEMKSTIKTTQEELMKNIKEFQDINSQIVGEQKVSSTKETQIKQTMSKIEKVTTQFVETAASSSSASSSSTSSQQQNAGSSQQQNAASSQKHTAASSQKQTASSQQETAASSQQENSMGRLKTN

>Rs174200

MAKFPLGICLMFMITTSTIYEVQGHFLLDHYLTKIPKLSSEFEPFAFKGINSFIDHLEGLCPLKADYKEFFTKLKDFMAFINSASGSSSEFHSQLKTKSEELFKAITKMGGKAGPSAHTSKLIECLMSMGKTLAEYKRSGSQTLTSEQKTELVSSMSKWAQVIGQFVKSVTEKSGGGNIDLKSLGCGGGTSVNVASSMRAAGSTGSSEASSSMQGESSAEAGSTAEAGSTAGAGSGAGDGETPGAAGPSGSPTDVSSAESGESSKDESSKDERSKDESSKDKSSKDESSNKDESSKDEKDESSKDEKDEKDGSSKDEKDESSKDESSKDESSKDEKDESSKDKSSKDESSKDESSKGESSKDESSKEESSKGESSKDGSSTGSEESSKGASGASSNEESSTETQSETGAESGASAGAPSGGPTSGDAGAGGPSGSSSDMSAGGATGSTSGSGSATSTQTSAEGESAMNSGASFSDTTGGSGTEAPASSPSGSASASGSSEASSLSGDSSLQAGSSSTGAASGSATDVSAQGESSMKGSIATQKSSASYKKSHKESSDQSSFKHSSQEIKAGSSSN

>Rs188390

MSRFQNLLCFTILLATVTFFNVASAHVKIKLALPQTGGPINVGDVELYTVKVVSTFVADLEKECAKTEKVRHFFEKVNEFSKCVCSVSKVKDHESHMKAKAGSLFQAISALGSDENGSKGGKVNKLQKEKTEAMETVKMLQSIGEKITGGKNNETEMKETLKLTTKQQKEIKDGILEWLNVINRIAKKADEISMKSSSISETKKESKEEKSVGPRILAETKSSTKSNKNSDKGSDNKKNKKGESKKKKKNGGKEGRDGSAKSKRGSDDNKNSKNKRKSSDNANKNQQTKYGKVEKKKGGVEQSRNKTAERIRAEEADELD

>Rs317160

MANKNVLALCLIFLVVSSVVYEAQGHFLLKMYLKRKFLGKAGDFTPFACKGMLLLMSRLQRGCPATEGFKTFFSLFKSYVKFIKTASTTSESTDTQLTTKVDALTNAISVLTGAKSGSNSNFRETMLSMGKTLMEQKRSSSERMTLQQKKVLVVAMVQWAKTVVTLVKTAVETAGKSIDVSNLGLDVDVNAVVGSGSNESPQSGSPGSNSDSATTSGSQNTDSTSTGSNTNSESPSETTTESPSESSTDSPSGTTTESPSENPTDSPSGTTTGSPSESPTDSPSGTTTESPSETTTESPSESSTDSPSGTTTESPSENPTDSPSGTTTDSSSGTTTDSPSGSPTDSPSGSTTDSPSGSPMDSNSGTTTDSLTGTNTESPSGSPADSSSGTTTGSPSDTTTDSPSGTTTDSPSGSPTDIPSGSFTDSTGITTDSPSGSPTDSPSESPTDSPSDSKSASGENSAKEVEIQTSKEARSFIHALEKKYAGTVELDTFFEKLKTSMSASTKISNTDEKRFVSKMNSAVSSVSEAASTVSSKLAKSPEAKSRMKSSKEQLMKTFKELEDLNSKIVSENKGKTVSSTQHSELKQALSKWEQVTTQFVENLTSSSSSSSSSSSSQSQKSQQSKQSHESQQSQQGSTMKTQTN

>Rs317140

MARFPLAICLMCIIVASSTVYEAQGGFLLRHYLRKLPRLANEFEPFAFKIMLTFIDNLESLCSSKVEYKEFFSKLKAFLIFINSAAGKSSSSEFESQLKAHSEGLFKAITALGVKASADTSKLIESLMSMGKVMAEYKRSGSLTMTSEQRRVLITSVMKWAQVIGQFVKTVREKTGEGDIDLPSLGIGGGDDDDAGSAGGGSSGSGSSSMGAGSAGGGSSGGGASSLGAGSAGGGSSSDTGSTAGAAGGSSGDTGSTAGAAGGSSGDTGSTAGAAGGSSGDTGSTASVGGGSSGDTGSTAGAAGGSSGDTGSTAGAGGGSSGDTGSTAGAAGGSSGDTGSTAGGSSGDTGSTAGAGGGSSVEAQSMTGAGSGSGGGGTSAGGSSGSAGGSSGNTSTMSTEESSSMSGGKSSKDSKIATGGSASEESTSGGASGGSTHTTATGESSMNNGDSYSDSTGGNTMGSPTGSPSGSTSGSGGSSFTGSETGSSSYQAGGTSAGGPSGSKTDSSAAGASSMNSGYSAKGTSRTQTSASYQKTHSKSSDKSSFSHSSEEKSSGNV

>Rs317130

MAVNAPNKIKDIEPYISNRAIGFVLKLEDECPIREKLSSFFEKLKDLLKLESSVTSMIEDNEPKTFISHMKSKADNLLQTMLMIGRGLLSSSVRKEMFKVIKSLTELHAAIGKVITEKHIKGDGTMSLSLEQKNAVENAVSQWEVTITRIVKIVVEAKSTSSSGTSGEESSTTEKNSTSVNKSMLESNKRNSGSMQENSNGVNESNEDSRNDVAMEAKEGNRVEDVKGDANNEKQLEVQGDNTDSNIDTNLETKGDVNSGIEAKTDESSMTKNPEEAQGNNGVPKKDNNFENKGSDATINDGDRTEENEEETQENNGEQVMNENLENNKDDREVKDDGLVQTKTNHEISMEKNREHTQGRTEVSMNGETMHTKSGNDVSNKEKEVEVQGESIGDSTKDVNLDNKEDLKRGGPQLEVPGGSIGDSQINKNLDTKEDKSEVGAKNDGSSMTEKSETQRNDKESTKNTNLENEGSEAATNDGYHTKENGDATQENNEELVKDANLENNKNNKEVKDDELVETNTDHENSTEEKREHTQGGTNVSMNGEMMHTKSGNADSNKEKEVEVQGENIGDSTKDVNLENKEDLKGDANSEKPLEVQRGNTGDSHTDKNLDTKEDVKSEVDARNDGSSMTENSEGIQVNNDVFTKDTNLKNKESGARTNDEDHTKDNGEETPENNGENVKDVNLENNKDNKEVKDNGLVETSTNHENSSEEKREHTQGGTDVSMNGEVMHAKGENVDSNKENEIEFPGESIGNSTKDINLENKEDVKSEVETNKNDESFMKEKQRNSQGNDRISTKDQNVESIGAPEEITNHKTVETMKNNGDHVKENREENNGESMKDADLENNEDKKELKDNGIVETKTSNESSVKEKQEEAQGSNEVSMNGQMDYLGENVDSNNKKVVEFRKGHAEDSNKDSNSKAEAIRNDGNSTKGKGEEAEGKNGVSTEDKKLENIKGEQELNNNIPVETKTDKETTSEEEKEKNHVNNDKSTYGADNKESKDVKLEEARENKKENTDNKWGEAKENEESFMGEHQKEVSVDKKGDNKEIIDEKSLESKENKKKFMQENQEETPRSSTGDITKVNNKEDKKKSKDIGSTEANENNESTMKESHQDSHGKGDEYTDLDSKEGKDDGSIETTENKESSTNKMQEQVQGSSRDTIKVHKNEPIEARPIESIEKDVQKGGGDPMQYKKDKNKNTSDVNSEKIDKEKKRYGNSKPKKKQKKENEDHKSKKIEENKKKIYNKSISNNNKEEKKKQKEHKEHKSKKEEEEKRKENERNKLKNEEERKNKKEDEVRISKKKKEEKRREHEKNISKKNEEEKRKQKEQEEHISKKKEEEKREEHKRIKLKIEEEKKKQKEHGEHIFKKKKEERRKEHERNKLKKKEEAKKKHKGYEEHITKKKEEEEKRKEHDKNKLKNEEEKKKRKEHQEHKSKKKEEEKMKKHERNKLKNKEEKKKQKEDKEQMSKKKDIEKRREHERNKSKKDEEEKKRQKEYKEHISKKKEEKKTKENERNKLKNKEEKKKQKEHGEHISKQKEEERRKEHKRNKSKKKEEDKKKHEEHITKKEEEEEKRKKHEKNKLKNEEDKRKQKEHISKKKEEEKKKEHKKNKLKNEEEKPKHKDHEEHISKMKDEEKRKEHERNKSKKNEEEKKKQKEYEEHISKKKEEEKKREEHERNKLKNEEEKKKQKEHEEHISKKKEDKKRQEHEKNKSKKNEEEKKKKKEYEEHITKKKEEEKRKEHEKKKLKNEEDKKKQKDHEKHISEMKEDEKKKEHERNKSKKNEEEKTKISSDDKSKNQKTEMLTNAEKKGEKKEKELQAETPVQSEESEKEMQAEAPTQSEESEKETKAEAPAQSEESEKKMQAEVQEQNEESEKEMQAEAPVQSKESEKEMQTEAPAQSEESEKEMQAEAPAQSEKSEKEMQAEAPAQSEESEKEMQTDVQEHNEESEKEMQAEAPTQSEESEKEMQTEVQEHNEKSEKEMQTKDQENNEESKKEMQTKAHTENEESEKEMQAEAQARSEESKKEMQAEAHARNEKFKKETQAKSAESEEKKQSMSEEKNEDSSMKKQEEIQETDVGEQKDQTVAKDEDSTNSKTTEINEGSEESSKDGNTTEVNGSKDNSMEEGTVETSGVKEDSMKDKATEVQGNNKSSTSDTSMATEVQGHNNSSTSNTTMDVKGGNIDINEDSAKNKTTEAQGGGAGDSTNGETEETTETNDSMNNQNMQHVEGDNIDINEDSTKNNTMEAQGGSSGDSTNGETEETTESNDSMNNQNVQHGENNANSMNNQTAESSTTEQQVTSDIESSTSKEVTSFISNLEHKSPVTQEFQSFFQKLKDYMKYAWPVSSTFEATDSRSYMSEMTNMATKVSDAMAVLQAKKLGSGLMKTTLQGYQQEVMKTLTILQSVISKAVSGQRSQNSGSLTLTLSQQQAIKEITSKWEQVMTQFVRVATESEKQFSMDISTGNGFHMKKSFSSSSSSSSSSSPGLDFNLNGENRIW

>Rs420610

MARVHILLCFTLLFAYVTFFDVASAFLKLKPSLPQIEDPKTVGDVEEYTVQVVMVFVGDLEKECPKTSKFKMFFDKLRGFAKYVCPLKIFGKKDDADMKTKEAGILKSIASFAIGRIKSEIQEEKQEAIENFKFMKSLAGRILGGRKKEEKATTALTPEQLKEIKDGILKWQTTIVKITNTMVVSTTTSESSESTTGGSEASTTGGSTGSPSNKPEAGSNKPEAGSNKPEAGSNPGGGTPSQDTNNESEDTTNTASTATETGGSTGPNSGRSTGSPSNKPSAGSDSGAGTPSTDTNNQSQASANSASSASSSQTTEITVTEVETQTSEQVMTFLMNLEKKSPQKEEYKQFFEKLKSTMTGKVSSPKKKGGLFSMIKGAVGKIGDAMQFIRSRIGNKSAEVKKSMETYQAEVIKSMQELDAIYAKIVSQNQSKKGGALTCTPEQQAEIKTTITKWEQVTTQFVEVAIKSETSTTSSSSSSTSTGTAQAN

>Rs491740

MANNKSVLALCLLFLVASSVIYEAQGTFLLKMYLRRKFFKRAMQFTPFACKGMIFMLHRLQGGCPATKGFKTFFSLFISYVNFIKTATVSKTTNTELTTRCDGLAKAISVLTGANKVSTDFRETMLSMGKTLIEQKKAGAVPVTPQQRKMLIVSLVQWTRTVVTVVKTAVETAGKSIDTSNLGLDVDVNDIIGGQTPTTPTTPTTPTTPTTPTTPTTPTTPSTPTTPTTPSTPTSSTPTPTTPTTPSTPTSSTPTPTTPTTPTTPSTPTSSTPTPTTPSTPTSSTPTPTTPSTPSTPSTPSTPSTPTTPTSSTPSAGSTTTQSATSSTASAKQVETQTSQEVMSFISELEKKYAGKAELNTFFQKLKSSMSATSKIATTDEKTFESGVKTAAGTLNEAAESVTEKLGTAETKQKIESSQQQLMQTFEELKEVNSKIVSESKGQTVSSTQQSELKQTLTKWEQVTTQFVETAVSSSSASASSSTQSQKSQQSQQSLQSQQSQQSLQSVQTQQGSTLKAQTQTN

>c0001_02342

MVFVSDLEKECPKTSKFKLFFEKLRAYAKYVCPIKRNDQKDYDLDMKAKAGGLFQAISSFAIGKIKEEIQEEKMEAINTFRWMKSVAGKIMGGRKKDEDEAAMKLTAEQQKEIKEGILKWETVIARITNTMVTGTTTTSSSGENSTSTKEGGKGSGSGGSFKDTTGSGSNSGGPSGSPSGGPSGSGSDVAGKAETSFKDTTGGKSIGSSESPTGSPYGSSKGSSSSGSVATGEGKAVTVTEVEAETSKQVMVFIMNLEKKCPQKEEYKAFIEKLKGTMTTSSKVSSEKRKGLFSRIKSAAGKLSDTMAFLRSRIGGKSAEVKKSMEAYQAEIMKTMQELDAIHSQIVSQNQGKKGGSLTCSAAQQAEIKQTITKWEKVTTQFVETAIQSEAIQSESSTSSASSSSASLGKVMPN

>c0003_00304

MTRISLALCLMLLFAIGDVHETQAQATFSLLDYLNLFPKLGKDFEPYASKGLLDFASVLEGKSPATVEFKTFFKKLKDYISNCFKPASSGSIDIQGEISEKSEQLFLSISALNGTKDGTSFDSWRLIEGLVSMDKVSVEMKKGTSNEISSQQWDRLYGSMSEWIVRIGLFAKSTSEVNGRLIDLSQFDINSIDPTYVSLSKEVGNKTQEPFSLPLYLRNFPKMGKDFESFAYKGMLDFVHELEVKCLANAEFKDFFAKLNDYMAGFKTVSPESYSIETNNITTQAMKLFIASSALNGTKVGTSDPWRLVDGLVSMGEVLVKMKKSGSNEITFEQRRELTWSLVKWARAISQFVKTASEKKGVTMDLSSFENYYNSYVLVPPKGATGRGNPDGGNKGSNSTKP

>c0003_01187

MARVQLLLCFTILFASVTYMEVVSAHLNLKPALPQMEDPKTVKDVEPYTVKVVMVFVSDLEKECPKTNKFKAFFDKLRAYAKYVCPIAKKDQKDYDRDMKAKAGGLLQAISSFAVGKIKEEIQEEKMEAINTFKWMKSVAAKIISGRKKEEGEETMKLTAEQQKEIKEGILKWETVITRITNTMVQGTTTNSSSGGDSTSGKESKTGGGKGSGGGGSGFGSGTSFKDTTGSSSNLGSPSGSPSGSPSGSPSGSPAGSSSGSTAAAAGGSGSSFKDTTGGSSITPAAGPGPSAGPTGSGSGSTVSGGQGGATTVAEVEAEASKEVMTFILNLEKKCPPKEEYKSFFEKLKGTMTASVKVTAETKKGFFAGIKSAAGKVSDAMSFMGSRIGGKSSEVKKSMENYHAEVMKALKELETIHGKIISQNQGKKDGSVAVTAEQRTEIKQTITKWEQVTTQFVETAMKGEASSNTTIGIDKVKLN

>c0003_01222

MARTSSAVCLLLLVAFSAVYEVQGTFLLRHYLRKFPRRNRDFRPFACRGMLKFVDLLEIKCPFKPEYKNFFGKLRTYTAFLNSASGSKNFDADLKGKAEGLQTAMSALGGKGGSSPDTGNVMDAVMSMGKTLNTQTSGGSTEMTLAQRKELIISMVKWARVIGQFVVSTASKSGGSINISSLGIDEDSPASPSTGTGSGTTTGGSISGGTGTTGGGSVTGNTGTTAGGSVTGNTGTTAGGSVSGGTGTGTNTGSGMAGGPASSTNGGSATGGSSFGRSFSGKTGSTNYEGSVNYQAGQTQQTATGGS

>c0003_01223

MARISLGICLMLVVVASSVIYEAQGTFLLKKYLRSKFPRKCNEFTPYANKGMVVFVGDLEGGCPATAEFKTFFTQLKSYMTFIDTASASSKNIDAEMTTKCDGLFKAMSAMNGGAVGKSADAGNFKATMISMGKTLVEQKKNTMMMTLAQKKTLITAMVKWTKMIATSVKTASEKKGKTIDITSYGLDVDLNDKSIISVSGGTQKGSSSTKTETSSSTKSGSFAAGGGVSSKTKETGGGGTEKGSTSTKKEDASSTKAGGGGSASAKGGSGGTFKDTSGGSSGGPTGSPSGGPSVSGKASAKGGASAGGSATSMKKESKGAGSSKSSGSSGTSVKQVETETSKEAMSFIMELEKKYAGKAELKPFFEKLKASMTASAMISSKTGQDYDSVTRSATGKLSEAMTFVGSRFGKSAKMKGNMETCQDQLMRTLKELQDINGKIVNAKTVTSTQQTEIKQKITKWEEITTQFVETASSSSSSSSQQQGNMRMVKNN

>c0003_01224

MAKNVLALCLMFLVASSVIYEVQGTFLLRMYLRRKLFPRRCVDFTPFACKGILMLAGTLQGGCPATREFKQFFSTFKSYIAFIGSVSASSRNIDSEMTVKCDGLAKAMSVLSAVKGGASMSSDFKETMLSMGKTLVEQKRSGSKIMTLTQRKELLVAMVKWTKMLATFVKSASETRGKSIDISNLGLDVDVNSSVGGGSEGGGGTGTGSSSSTKTGGQNGGSSSTDNGSAISGSASGSATSSRSGSAASSGSGGTYKDTTGGGSGGPSGSPSGSPTGGPSGSTPSASGAASAKGGASAGGGAIATGNASSKTKQTTTGSSTTGSTKTGSANSYTDTTGATTGGPSGSPTGGPSGSTPSGSTPSVSGETSVKGGASAGGGASTKTNTGGSATTSGGSSTTTTSVKQVESDTSKEVMAFISGLEKKYAGKAELKVFFDNLKTSMSASSKISTTDTKDFLSGMKSAAGKLSEAMLFVRSKFGKSAETKTGMETCQEQFMKTLKELQDINSKIVTQSQGQMVSSTQQTQLKQTITRWEQVTTQFVETAASSSSSSSQQGMTMNTQAS

>c0003_01226

MKTVSLLFLCCITILATSLSLNVVSAHEAVVNTPNTINDIEPYISNRAIGFVSNLENNCPVREKLGSFFDKLKDLLKLESSVTPFIENNEPKTFKFDLKSKADNLLQTVMMLGRGLFSSSVRKEMFEVMKALTELHAAIGRVIMEEHIKGDGSMSLSLEQKNAVENAVSQWEVTIGRIVKIVVQVKSEGSGAVSGEEGSTTEDNSASVNGSMVETNGGNSESMQENVDGIKEGNEDSRNDVAMEAKEGDKVEDVKEANEDSNTEKPVEVQGGSIGDSTVDKNLDTNEDVKSEVETKTDGSSMTEKPEEAQGNNAVSTKDNNLENKGSEATTNDGDHTKENEEKTQETNGELVKDENLEHNKDNKDLKDGALVETNTNHEISTEEKREHTQGGTEVSTNGETMHTEGGNADSNKEVAFQGESTGNSTKDINLENKEDVKSEVEANKTDESSTKEKQEDAQGNNGVSTKDHNLENMGAHEDTMDHKSVEAMKNNVDHMKEKREETQGNNGESVKDENLENKEDKKELKDDESVEAKTGNENSMEQKREEPQGSNEVSMNGQTVENLGGNADTNKEKGIEVKEGHVKDSTKDANSEVEAEKNDGSSTKENGEEAQGNSGISTEDKNIENIGGEQEMKEKMSVETKTNKENSSEVKKEEGHGNNEGSTEEEIKGGKKESKDDQLSKDKENVGSSMNEKGEEAQTNGGDKKESKDDKSEEAKENEESSNQEEVQGNNNNFKNTDNKGDKKVIMDDKSMEAKENKESSMMENREETQRGNSGDLGKIDNKEGKKESKDNESVEAQANTEKKESQGNVGESTKVENKEGDDDKSVDAKENKESSKKKKREKAQGGGGDTTKVENNESKDANLVETTQQNKGSAENMDVEVQKEMGESMQYKKEENKDTLDGNSKKKDKDTKEKKVAEERELKKKQEYRKEKKESENSKLNNTEEKKEKKESGKTKSKKKDEEKKDNKEFDEGKSEKKDEKEKEKKEYGDNTLKIKEEDGKEKTNSKDHNSKKKEEEKREKKEYEISKSKEKKEENNRSEKKKEDNTGKKESMDNTSMKKKEEKEEKKENINEEGKKEKKDSQENKTKKKEDGKKGNKEKKDFELKKNEDEKNKKKEHEDNKSKKKEEEKKKKKEHEDNKSKNKEECEKGKKEHAPPAAEVSPPAPEVSPPAPEPEPELSVELPLGPPAEDPPPALPLPIPAALSVPPLELPPPMELSPAPVELSVGLPLGPAAPEAPPLAIPLPASAVLPVSPELPPPMELAPDEAELSLGLPLGLPLPVSPAGEPLSEPGLPLSVGLSPELSLVAGLPVPAEGEPLSELGLPLPPMLPSPIPELPDPVEEELPPEFPPPKPRDERSILPSPLFSPTVFTN

>c0016_00209

MPRLQNLSCFVILLVTITFFNVALAHVKIKPSLPKIGDPTTVNDVESYTVKLVSTFVAELEKECPKTEKFKLFFEKLNAYSKYVCPVSKARGHESDMKAKAGGLYQAISALGSGKNESKQGKANKGLQQGKTEAMNTVKMLQSVGEKIAGGKNNKTENKGTEKLTVEQQKEINDGILKWLQVITQIAKTAEEEIGSKSSSKTQTTREGKEEKSSTQKENKKSGQKDSEMAQITALPRGGRTSKAGNKNDISKDARNSAKEGDDNKNKKGESKKNKGAKGGSQGSANNKPTTEDGKGKGSNEGKGRRGKGESREKADKSPPTYYGKIEPAKGGARNKTEERLRAEMADELD

>c0858_00002

MKKKEYEDNKSKKNEEEKRKKKEHEDNKSKKNEEGKKEKKEHEDRKLKKNEEEKKKKKKEHGDNKSKKNEEGEKGKKQHEDRKSKQNEEDKMKKKEHEDGKSKKNEEEKMKKKEHEDNKSKKHEEEKRKKKEHEDNKSKQRLMQRVRNLKEKYKLRFKQEMRKPRKKCKQRLMQRVRNLKKKYKKRLKQRVKNLKKKCKQRLTQSMEAKKNPWMVTETQVNGNNSTSDTSTEDIGPKPDINENSTKNKTLEAEGGGNGNTTNGKTEETTEGNDSMNNQNVQNGGGNANSTNNQTTGEAVNSTTEQKVTTDIEGSTSKEVTSFISGLEKKSPGTQEFQGFFQKLKDYMKYACPVSPTFEAKDSQSYMSEMINMATKLSDAMAVLQAKKIGSGQMKTTLQGYQQEVMKTLTILQSVMGKAVSEQQGQNDGSLTLTLSQQQAIKEIILKWEQVMSQFVKVATEGEKQFSMEISSGNGYHIEKSSGSGTGSSSSTSTDLDSKLDGESSKMVDVNG

>SI_scaffold1551_21

MSRVQSLLCFTILLATATFFNVASAHVKIKPALPQIEDPTTVNDVETYTVKVVSTFVADLEKECPKTEKFKVFFEKLNAYTKYVCPVSKVKDNESDMKAKAGSLFQAIADLGSAKNGSKGGTGNKSLQQEKTEAMNTVKMLQSMGGKIAGGENSKTETVKLTVEQQKEIKDGILKWLQVITKIAKTTEEISLKYSSKTHTKRESKEEKSSTQTQTKTSSRAAKAGNKDDLSESAKNSAKESDDDKNKKGESKKKNDGKGESKESANDSTSGDKKEESKKKNGGKGENKDSADDSTSGDKKEDSKKKNGGKGESKHSADDSKSRDKKEASKKKNGGKGGDQDSANDSTSGDESSSKSKGDAKEKGSSKSKGETSEKADKKNHQISYGKIEPKKDGAEQSRIKKEERLKAEMADELD

>SI_scaffold2787_1

MARVHLLLYFTLLFASVTLLEVVSAHLKITPSLPQIEDPKTVNDVEGYTVKVVMVFVSDLEKECPKTSKFKLFFEKLRAYAKYVCPIKRNDQKDYDRDMKAKAGSLFQAISSFAIGKIKEEIQEEKMEAINTFRWMRSVAGKIMGGRKKDENETSMTLTAEQQKEIKEGILKWEAVIAKMTNTMILSTTNSSSSEDSTSEKGSSNASSETSGTGSSSITTTGSSFKDTTGSGSNSGSPSGSPSSSPSGSSSPVEDGAGTSFKDTTGSKSITSSGSPTKSPSEDSMNFASSGSTEASQNKSMTVTEVEAETSKQVMTFIMNLEKKCPQKEEYKQFFENLKGTMTTSSKVSSEKRKGLFSRIKSAAGKLSDAMAFIRSRIGSRSAEVKKSMETYQGEVMKALEELDAIHSKIVSQNQSKKGGSLTCSKTQQAQIKQTITRWEQVTTQFVETAIQSESSSTSSSSSSSMAKVKEN

>SI_scaffold27_215

MARFPLAICLMFIIVATSTVYEAQGGFLLRHYLRKFPRASSEIEPFAFKGMLSFVDNLESLCPLKGEYKEFFTKLKAFMAFINSASGSSSDFQSQLKAQSEGLYKAISALGGKVGSSADTSKLIESLMSMGKTLAEYKRSGSQTMTSEQRTQLITSMAQWAQVIGQFVKTVGEKSGDGTIDLSSLGFGGGGDDANAGSSGSGSSGAGSTGMGDGSTGGSGSPSDGGSPSDGGSPSSDSGSGMGDGSTGGSGSPSDGGSPSDDSGSGMGDGSTGGSGSPSDGGSPSFPDNSGSPDSDIGSPSSDTGSPVDGGSSANSPTSSTGGGSGSPSGSPSGSSDSSGANSMGGESSEDAEGTAGAGSAMANGGTSGAAGPSGSPTDSMGGGSTSSTESTTGAGGENSNSGSSVNTQSVTDVGSGSADGGASAGGPNGGPTDSSGSGSSGSSSSGSGSSGESSTETGSANGETSSSGGETSGAGAGAGAGTGGPSGSTSEMSTEGASSMGGGSASGGSTTGGASGGTSYSDSTGGNTMESPAGSPSSSASGSAESSYSGSGSGSSSYQGGGAAAGGPSGSTTAGSAAGYSAEGSATTQTNSQGSSAGNSFAAGGGSGSAEGGSGASEGKSFKGQMGSVSYEGSASANYEKMHSKSSGSQKSFSHSSEEKNSGNV

>SI_scaffold27_216

MAKNVLAICLMFLVASSVIYEVQGSFLLKMYVKRKFFPRRCMDFTPFACKGLLMFIGTLKAGCPATREFKEFFTKFTSYIEFIRSSSASSNNIDMEMTTKCDGLAKAMSLLTAVKGGASSSSSESSDFKDNMLSMGKILIQQKRSGSMIISLAQKRELVRSMVSWTKMIATLVKSAAEQRGKSIDISNLGLDVDVNSSVGGESGESTETDSSNTNTGSQNSGSTSNDSGSSASGSATSSRSGSSSKSGSSSGSGSSYKDTTGGSSGSPSGSPTGSPSGSTPSSSTPSDSTPSDSTPSSSTPSASTSTGSTPSSPTPSGSTPSGSTPSSSTPSSSTPSGSTPSGSTPSSSTPSSSTPSGSTPSGSTPSSSTPSASGETSVQGSTSGSSDSSTTNKQSATEKSKTSSTSGNASSYKDTTGTGATTGSPSGSPTGSPSAGSGSRSGETSVKGSASGSEGATSKTSSESSSTTSVKQIESETSKEVMEFISNLEKKYAGKAELKVFFDSLKTSMSASSRLSTGDSKDFISGMKSAAGKVYEAMSFVRSKFSKSEETKSSMETSQEQFMKTLKELQDLNSQIVSQSQGKTVTSTQQTELKKTITRWERVTTQFVETAASSSSSSSSSSSKSQQSSTMNGQAS

>SI_scaffold27_217

MARISLGICLILVVASSVIYEAQGTFLLKKYLRSKFPRKCNEFTPYANKGMIMLVTNLESGCPATAEFKTFFTQLKSYMEFIDTSSASSKDMDVEMTTKCDGLLKAMSALNGGAAGKSADAGTFKATMISMGKTLVEQKKNTMMMTLAQKKSLVMAMVKWTKTIATIVKTASEKKGKTIDITSYGLDVDANDKSIISVSGSSESSSSSTKTESGSSTKTESGSSTKSGTVAGSGTSGGSTEKGSSSTKTEDASSKKADGSGSTKSGSGSGSSFKDTTGGKAGSPSGSPTSSPSATGKTSEKGSASASGSGTTMKTQSSTGSTSVKKVESETSKEVMSFIMELEKKHAAKAELKPFFETLKSSMTASAMTSSKNEQEYSSTARSATGKLSETMAFVGSRFSKSAKMKSNMETCQDQLLKTLKELQDINSQIVSAKTVTSTQQTELEQTITKWEKFTTQFVEAATSSSSHSSSSSQQSQQSQHSQEQRSSSSQQQSSMGMVKNN

>SI_scaffold27_218

MRKLPRRSRDFRPFACRGMLKFVNVLEGKCPLKPEYKNLFGNLRSYMSLLSSASGSKNFDADLKGKAEGVQSAMSALGGKGGSSADTSNVMDVLMSMGKTLSTQTSSESTEMTMEQRKELVMSMAKWARVIGQFVASAASKSGSSIDISSLGVDGIDANVATEGAASPSSGGSAGSASGSYMSGKTGSTNFEGSVNYQAGGKSSQQTTTGSS

>SI_scaffold27_256

MARVQLLLCFAFLFASVTFMEVVTAHLKIKPSLPQIEDPKTVNDVEGYTVKVVMVFVDDLEKECPKTNKFKAFFERLRAYAKYVCPIAKKDEKDYDRDMKAKAGSLLQAISSFAVGKIKEEIREEIREEKEEAIQTFRWMKSVAAKILGGRKKEESEETMKLTAEQQKEIKEGILKWETVITRITNTMVQTTTTNTSEGEGSTSGKGSPSGSDSGSASKSSGSGSSSFSSGASFKDTTGSGSNSGSPSGSPASSPSASTDAAASGSKSGSSFKDTTGASAMSPTAGPTSSGSSESASGSQSSATTVSEVEAETTEEVKSFLMNLQKKCPPKEEYKSFFEKVMGTMTASAKVTAEKKKGFFAGLKSAGGKISDAMSFMGSRIGTKSSEVKKSMENYHAEVMKALNELETIHSQIISQNQGKKDGSVKVTAEQRTVIKQTITKWEQVTTQFVETAMKSEASSNTTVGIDKVKLT

>Thlar.0042s0043

MARVQLLLCFTILIASVTLLDVVSAHLKIKPSLPQIEDPKTVKDVEPYTVKVVMVFVSDLEKECPKNNKFKAFFEKLRAYAKYVCPIGNRANQEYEKDMEAKAGGLFKSIASFAYGKIKQEIKEEIQEEKQEAIETFRWMKSLAGRILGGRKKEESPETMKLTAEQQKQIKEGILKWETVIAKIANTMVQTTTTTTTTTTSSSSSSGEASTGAKESQGSKSSGAGNSYTDTTGSGSGSGSGSGSGSGELSTGGKGSQGSKGSGSGASYKDTTGSGSGSTMGSPSGSPSGSPKTSPSGSTYDAESGSGSGTSFKDTTGGSPMSPSGSSMSPSGSPKNSISSDESSTYRTTGSTTSASGESVSASQSVTVQEVEAETSQEVMTFIMNLEKKCPQKEEYKSFFEKLKGTMTASAKVTIEKKKGFFSGLKSAAGKISDAMSFMRARIGGKSAEVKKSMEAYHAEVVKAVHELDAIHSKILAQNQGKKEITVTAAQRTEIKQCITKWETVTTQFVETAVKSETSTNSSAGLDKTKLT

>Thlar.0042s0093

MARTSSAVCLLLLVALSAVYEVQGTFLFRHYMRKFPRRSRDFGPFACNGMLKFVDVLELKCPLKPEYKNFFGKLRSYMGFINSASGSATFDTDLKGKAEGLHTAMSALGGKGGSSADTSNVINVLLSMGKTLSAQKRSGSTEMSFSQRKELIMSMAKWARVISQFVASAASKSGTSIDVSSLGIDGVDASVAIEAGGSPTSGGSPSTAGTGGYGTGASGPTTSSSTQSTNSGSGTTYEGSVNYQSGEKSSQQTVTGSY

>Thlar.0042s0094

MARISLGICLMVLVVASSVIYEAQGTFLLKKYLRNNFPRKCNEFTPYANKGMILLVTNLESGCPATAEFKNFFSQFKSYMSFIETASASSKNIDVEITTKCDGLFKAMSVLNGGAGGKSADAGGLKVALISMGKTLVEQKKSAVVMTVAQKKRLIVAMVKWTKMVATFVKTASEKKGKTIDIKTYELDVDVNDKSIIGVSASAGTTEKSSSSSSSASGGNTKTENTPSGNTKSENASKTKESKSEKKSSSGGDAKTGAAGGATTYKDTTGAYSGSPSGSPTGSPSGSAYAGGETAVKGSSSGSGDSSTKTKSESKGAAAGETKTTSGSSYKTKTESKGAAAGETKTTSGSSTTTVKQVESDTSKEVMSFIMGLEKKYAGKAELKVFFEKLKASMTSVATISSKTAKDYVSTARSATGKLAETMTFVGSKFSKSAKMKSHMETCQDQMLKALKDLEAINSKIVTEKTVTTTQQTEIKQTITKWEKVTTQFVEGAASSSSSSSSSSSSSSSSGMIKNN

>Thlar.0042s0095

MAKNILAICLMFLVASSVVYEVQGTFLLKMYLRRKFIPRRCVDFAPFATKGILMLMSHLEGGCPATREFKQFFSTFKSYMSFISSASTSKTTDVQMSTRCDALAKAMSLLTGSKSQSSSSDFKVTMLSMGKTFMEQKSRGSARMTTAQRIELVSAMVKWTRMLATLVKTAAEKNGKSLDISSYGLDVDVNASVGSESETTETGTPSNQNSGASPTPTTETPVSSTPSDSKNTGYSGSGSGSGTATSSGSGNTGYTASGSGSGSSTSSGSGNAGYSGSGSGSTYTDSSGGSGSGSPSGSPTGSPSGSAVASGYNSKQSGTANGSGSGSPSGSGSGSASSYKSKQSETTANAGSENTYKDTTGATKGSPSGSPTGSPSTSTYAGGETPVKGGSSASGETTVKGGSSGTGGSSSKTKSESKGAAAGETKTTSGSSSKTKSESEGAAAGETKTTSGSSTTTVKQVESETSKEVMSFISGLEKKYAGKAELKVFFEKLKTSMSATSKLSSTDEKNFLSGMKSAVGKISEAMVSVRSKFSKSSEVKTTMETYQQQVMKCLQELQEINSEIVSQGETVTSTQQTELKQTITKWEQVTTQFVETAASSSSSSSSSSSSSKFQQNSAANMNMNMNTQAS

>Thlar.0042s0096

MARFPSAICLMFIIVASSTIYEAQGSFLLNHYLNKLPSSSNEFEPFAFKVMLRFLDNLESKCPLKREYKEFFTKLKAFMAFINSASGSSSEFQSKLKEQSQGLLQAISALGIKGGSSADSSKLIESLMSMGKTLAEYKRSGSQTMTSEQRSELITSMAKWAQVIGKFVKTVGEKSGDGINIDLSSLGVGSGGNDANAEGTGMGDGSAGDGGSPSSGSPSADTESPSDSGIASGGAPAGGPSGSTADNSGSGENSGASDAENGTSGGASGAENETTGGAGGGTGSSGGAGDGTGTAGGAGGGTATTGGAGDGTATTGDAGGGTGSSGGAGDGTATTGGAGDGTATTAAAGDGTTTTAGAGDGAGSSGGAGGGTGSSGGAGDGTATTGGAVDGTATTAAAGDGTTTTAGAGDGTGSSGGAGDGTSTTAAAGDGTATTGGAGDGTGTSGGAGDGSETTDGAGDETGTSGGGAGDGSETTDGAGDETGTSGGGAGGETGTSGDAGGETGTSSSAGGKTGTSGGTGGDKTEASGSAGGEASSGDETKTSSGSSDKTETSGSAGGETKTSDGANGDKTETTDSTGGGTGTPAGGGDEETKTSGGAGGSSDGKTSSNMGGEMSYEESKTAVGGSGSGGSGSTTDSSMTGENSMSFYSDSTGGTPMESPTGSPSGGGSSFSGSSSYKASEGKSFNYAKTHSKSSDKSSFSHSSEDKLNS

>Thlar.0042s0097

MKGVSLLFLCCITILATSLSLNVVSAHEMVENPPNTVKDIEPYISNRAIGFVLKLENNCPIREQLRSFFEKLKDLLKLESSVTPLIENNEPKTFKFDLKSKADNLLQTVFTLGRGLLSSSVRKEMFQVVKSLTELHAAIGKVITEKHIKGDGSVSLSSEQQTAVENAVSQWEVTITRIVKIVVEVKSGGSSETTSGQESSTAEHNSASLNSTTVENSGGNTTESMQQNVDGVKGENHVDSNNEKQEEVQGDSTINNNMETKEDVNSEEIEAKNDASSKTEKPKEAQGNDGVLTKDGNLENKGSEEESKNDEVVEPTTNNANEANQTREETQGNNGELGKNENSENVEGDKTSKDDGSVETKTNHEYSREEKHEHTQGGTEVSTNGETEDTKGGNADSGEEKKEAEIQGENSGDTTKDTNLENKEDVKPEVKANENVESSTKEKQEEAHGNSGVSTEDQNLENKGAHEETNGDSKSVEAMTNNGDHMKEKREETQENNGESVKDGNLDDKKESKEDESVGAKTSNETTMEEKQDKAQGGDEVSVNGEMEEKNGGNVDSNKENGVEVQGEHVEESTKDTNMESKEEAKSEVEAKENDGSSTKEKGEEAQGNNEVSTEDKNLENIGGEKELKNKTSMEETQNESQGSNEVSTEVETKGGEKESKDGKSSKDNQTVESSTKEKGDEAPINGGDKKESEDDKLVEAKENKESSMKENREETQGSNSGDNKEGKKETKDNESVEAGESNESSMQEKQQESQGKGEEYTKVENSEGKKDAKDDKSVEANANKESSKKDENNGEKRDIIDGKSKKKEKHKKKKKESENSKSKKNEEDGKQKKESENSISKENKEDEKQNKESENSKSKKKEKDEKEKKDLEHNKSKKKDEEKKEKKEHGDNKSKKKEEDNNQNKELEDNKSKKNEEGKKEKKEHGDSKSKIKEDKKEKTNSKDHNLKKKEEDKKEKKMSKDNKSEKNEQGKKEKKEHEDGDSKIKIKEDSKEKKKSKDHNSKKKEEDKNEKKESKDNKSKKNEEDKKEKKEAQDRKSKKKEEERKTEFENGKSKKEEKNETEDNKSKKNGEVQKENKKDNKSMKNEEEKKQKKDYEDNKLKKKEEDKKEKKKSEGGKNSKKHEEDKNGKKENKDHKLKKHEEEKKKKKEHENNKSKKKKEDVKMEKESEKGKSMKKEEGKKEKKEHADDKSKIKVEDTKEKEELEDNTSKKKEEDEKKKTKSEDHMSKKTAENKKEKKDNKENKASEDKKSKRKEEKEDSRDIKMKKKEEDKNKKTHSKDEKSKKKEQGEKEKKESHDIKMKKKEEDKKKKTNSEDNKSKKKEEGEKEKKESQDNNKSKKKEEMEKKNKESEEDKSKNTRKDGGDATKQVGFQSNTEDSMKNKEEEEESKDAKSKETEKQQKSSGQEKQVGVQVNAQDSRKDKKKTTEDAKVSKDGKSKDHEKEMQAEAHAKSEEAENELQAKAEEVNEKSLENQKGEGKEDDEVDKKAAEDVKSIGKEEDNEDSSMKKQEEAQETNVESNMGEKKDQNISIDDKSKETKESDENSAKEKEIDEGKSLNTGDSKEETKESNGDLVNGETEPIEGENEDSKDGKSVETKEGKNDSMEEGSKDGKAVEIKGGKEGSVESSTDIGKTVENNGDKEDSEDGRAVEVNGGNVDSNEEGPKDGKEDSMEKDSKDGRTSEINGGKEGSMNESSKDGKTTEISGGKNSTEEASKDGKATEIDGGKEDSTEKGSKDGKITETSEGKNSTEEISKDGKTTEINGGKEDSTKEGSENSKTDEISGDKNTTEEISKDGKTEETNGVKEDSKDGNIVETNGGQEDSMKDKVTEIQGNDNNGGKEDSEDGKAVEINGGNVDSKEEGSKDGKEDSTEESSKDGKTEEINGVKEDSKDGNIVETNGGKEDSMKDKVTEIQENDNNSTTNTSMEAKEDNLDINKDSTKNKTEEAQGGNTEESTNGKTEETTERNDSINNQKTESEGSSANSTNNQTTGETVNSQKTTTDIESSTSKEVTSFISNLEKKSPGTQEFQSFFQRLKDYMKYACPVSSTFEAKDSRSYMSEMINMATKLSDAMAVLQAKKTGQGLMKTTLQGYQQQVMKTLTLLQSVMSKAVSERQSQNDVSLTLTLSQQQSIKEIIMKWEQVMSQFVKVATESEKQFSMETSTVNSYHMTKSSSSSSN
