# Supplementary material for: Lineage-specific gene duplication and expansion of DUF1216 gene family in Brassicaceae
Source: PLoS One. 2024 Apr 16;19(4):e0302292. doi: 10.1371/journal.pone.0302292 (PMC11020792; doi:10.1371/journal.pone.0302292)

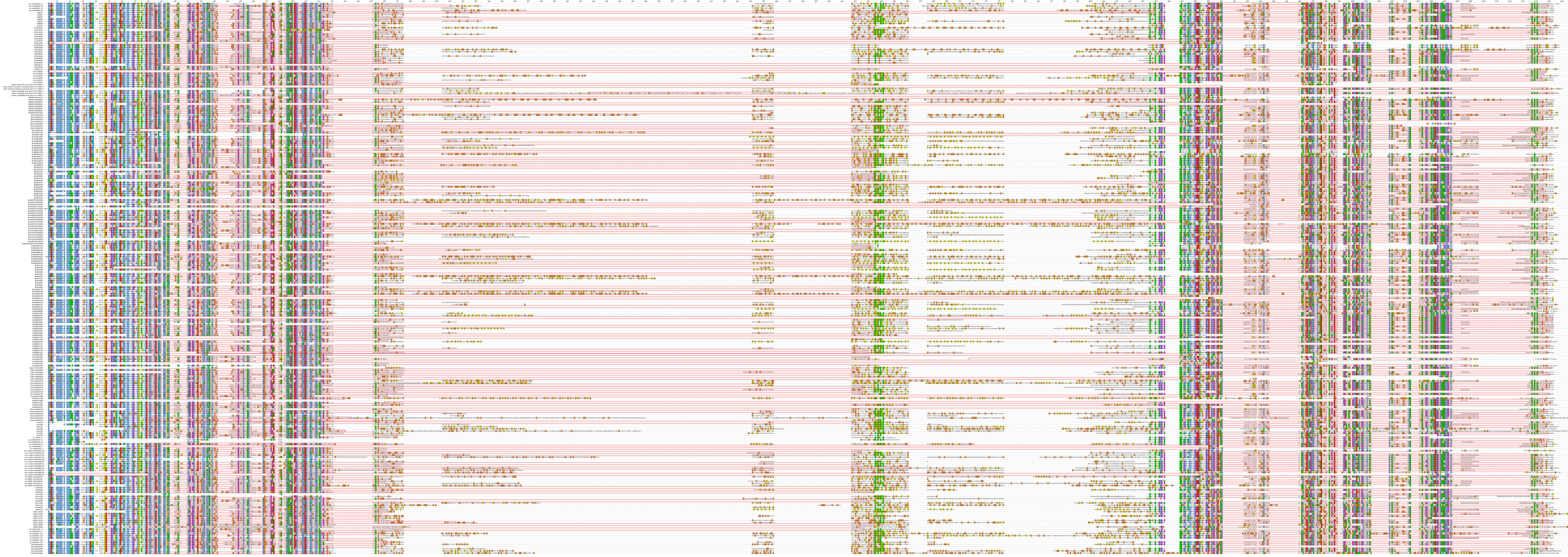

Supplement: S1 Fig — DUF1216 domains are framed with red lines. (PDF) [file pone.0302292.s001.pdf]
