# Supplementary material for: Lineage-specific gene duplication and expansion of DUF1216 gene family in Brassicaceae
Source: PLoS One. 2024 Apr 16;19(4):e0302292. doi: 10.1371/journal.pone.0302292 (PMC11020792; doi:10.1371/journal.pone.0302292)
Supplement: S2 Fig — (PDF) [file pone.0302292.s002.pdf]

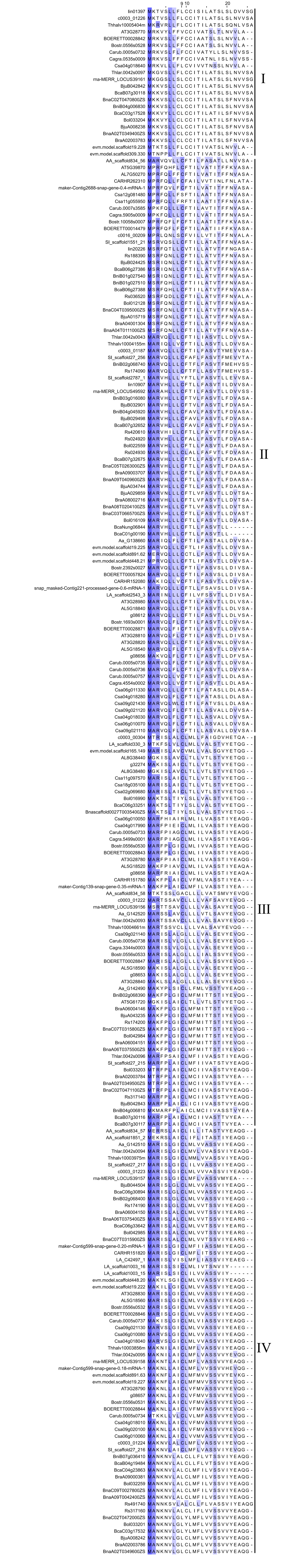

9 10
20
I
II
III
IV
