# Supplementary material for: Lineage-specific gene duplication and expansion of DUF1216 gene family in Brassicaceae
Source: PLoS One. 2024 Apr 16;19(4):e0302292. doi: 10.1371/journal.pone.0302292 (PMC11020792; doi:10.1371/journal.pone.0302292)
Supplement: Attachment — Submitted filename: Pone-D-24-02626 Review file.docx [file pone.0302292.s012.docx]

**Lineage-specific gene duplication and expansion of DUF1216 gene family in Brassicaceae**

| **Sections** | **Comments** |
| --- | --- |
| **Abstract** | - I suggest authors to check the English by a native English speaker - Italicize the word “*Brassicaceae*” througout the manuscript - Avoid starting senetence with a mathematical figure (see line 36) - Check for space (see lin3 36) |
| **Introduction** | - All scientific names should be italicized for an example see line 74. - The section of introduction is underdeveloped. Also, it misses out on several important references like authors gene duplication is the main way to produce new gene in Brassica, such statements need to be cited. The authors must mention about other gene families (at least two) which experienced gene duplication events. - How do DUF1216 modulate gene duplication? |
| **Material and Methods** | - To analyze phylogeny, on what basis different listed plants were selected. - In the methodology section, references are not cited for some context for the protocols used for experimentation by the author. Please cite appropriate references along with the text to acknowledge the contribution of other researchers and to justify the authenticity of the protocols used. For example, cite for HMMER, IQ-TREE. - There is tense inconsistency in methodology. Kindly check and rectify as it a grammatical error which must be rectified for uniform reading of manuscript. |
| **Results** | - Authors must add more clarity about the Ka/Ks related results obtained. What can be deduced if a gene family experience purifying selection pressure. - It is suggested that qRT-PCR will add more meaning in your expression experiment work. |
| **Discussion** | - What is the contribution of this research in field of science and to the society? |
| **Suggestion** | You can add citation from these articles just to add general importance of brassica.   - Farooq, O., Ali, M., Sarwar, N., Mazhar Iqbal, M., Naz, T., Asghar, M., & Ehsan, F. (2021). Foliar applied brassica water extract improves the seedling development of wheat and chickpea. Asian Journal of Agriculture and Biology, 1. - Ahmad, N., Fazli Ahad, R., Iqbal, T., Khan, N., Nauman, M., & Hameed, F. (2020). Genetic analysis of biochemical traits in F3 populations of rapeseed (Brassica napus L.). Asian Journal of Agriculture and Biology, 8(4).   Following reference should be cited to add more information about gene family duplication events in brassica.   - Lv, X., Wei, F., Lian, B., Yin, G., Sun, M., Chen, P., ... & Wei, H. (2022). A Comprehensive Analysis of the *DUF4228* Gene Family in *Gossypium* Reveals the Role of *GhDUF4228*-67 in Salt Tolerance. International Journal of Molecular Sciences, 23(21), 13542. |
